# Organic Carbon Monoxide Prodrugs Activated by Endogenous Reactive Oxygen Species for Targeted Delivery

**DOI:** 10.1021/jacs.5c05952

**Published:** 2025-07-03

**Authors:** Inga Cernauskiene, Claudio D. Navo, Carlos Labão-Almeida, Rupert S. J. Proctor, Bengt H. Gless, Wei Ting Khaw, Cong Tang, M. Milagros Muriel-Olaya, Gonzalo Jiménez-Osés, Gonçalo J. L. Bernardes

**Affiliations:** † Yusuf Hamied Department of Chemistry, 2152 University of Cambridge, Lensfield Road, Cambridge CB2 1EW, U.K.; ‡ 73038 Center for Cooperative Research in Biosciences (CIC bioGUNE), Basque Research and Technology Alliance (BRTA), Building 800, Derio 48160, Spain; § GIMM - Gulbenkian Institute for Molecular Medicine, Avenida Prof. Egas Moniz, Lisboa 1649-028, Portugal; ∥ Xi’an Fengcheng Hospital, No.9 Fengcheng Third Road, Xi’an, Shaanxi 710018, China; ⊥ Ikerbasque, Basque Foundation for Science, Bilbao 48013, Spain; # Translational Chemical Biology Group, Spanish National Cancer Research Centre (CNIO), C/Melchor Fernández Almagro, 3., Madrid 28029, Spain

## Abstract

Carbon monoxide (CO) has demonstrated therapeutic benefits in reactive oxygen species (ROS)-rich environments, such as inflammation and cancer. However, the targeted delivery of CO remains challenging, limiting its clinical application and necessitating the development of improved CO-prodrugs. Herein, we report a radical-activated, metal-free, CO-prodrug designed to address delivery limitations and avoid metal-associated toxicity. This tertiary aldehyde-based prodrug is stable under physiological conditions and, upon activation by a radical trigger, releases CO, 2-ethyl-1-butene, and a nontoxic thiol carrier. The stability of the CO-prodrug building block allows for its incorporation into synthetic peptides via solid-phase peptide synthesis and site-specific bioconjugation to therapeutic antibodies. We synthesized trastuzumab conjugates with a CO-prodrug-to-antibody ratio of 23 and demonstrated efficient, tumor-specific CO release in HER2-high-expressing cells. These findings open new avenues for investigating the therapeutic effects of CO. We anticipate that our metal-free CO-prodrug strategy will be broadly applicable to a wide range of synthetic peptide- and protein-based therapeutics.

## Graphical abstract

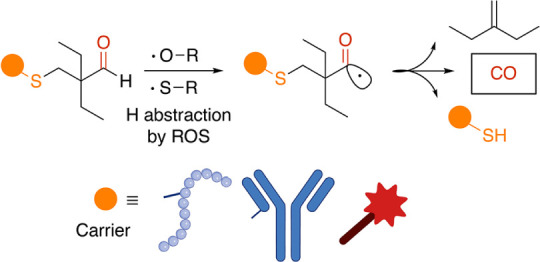

## Introduction

Carbon monoxide (CO) is produced in the body through the breakdown of heme by oxygenase enzymes. Since this discovery, CO has been recognized as a gasotransmitter alongside nitric oxide (NO) and hydrogen sulfide (H_2_S).^1,2^ Beyond established physiological roles, CO has shown therapeutic potential in many pathological and clinical conditions,^3^ including bacterial infections,^4,5^ inflammatory diseases,^6–9^ organ transplantation,^10^ and cancer.^11–16^ The safety and benefits of low-dose inhaled CO have been supported by multiple clinical trials.^2,17^

The broad range of therapeutic applications has initiated the development of targeted ways to deliver CO for medical use. The first reported carbon monoxide releasing molecules (CORMs) were metal-based (Ru, Fe, Mn, Co, Re, and Mo), where CO release was triggered by the solvent, nucleophiles, enzymes, or light.^7,18–23^ CO delivery and accumulation in tumors was achieved using metal-CORMs complexed with nonspecific histidine residues of bovine serum albumin (BSA), facilitating biodistribution and increasing half-life.^24,25^ Organic carbonyl-based CO-prodrugs were reported later to be capable of releasing CO when triggered by light, mild physiological conditions (pH, redox potential), or bioorthogonal chemistry.^26–30^

The therapeutic potential of CO highlights its importance in reactive oxygen species (ROS)-elevated conditions.^31,32^ Hence, it is highly desirable to develop CO-prodrugs that release CO upon ROS triggering. A few examples of CO-prodrugs activated by endogenous ROS have been recently reported in the literature, including metal-based nanoparticles^33–35^ and organic carbonyl prodrugs.^36–42^ However, most of these CO-prodrugs have low aqueous solubility, and the byproducts are undefined or have unclear physiological relevance. One notable example of ROS-triggered CO release is CO caged in a cyclopentadienone moiety like **1a-3**, which is formed spontaneously from norborn-2-en-7-ones **1a-2**, where the former is a result of spontaneous beta-elimination following sulfur or selenium oxidation by endogenous ROS in **1a-1** (Figure 1a).^43,44^ The phenyl groups can be exchanged to enhance water solubility.^45^

**Figure 1:**
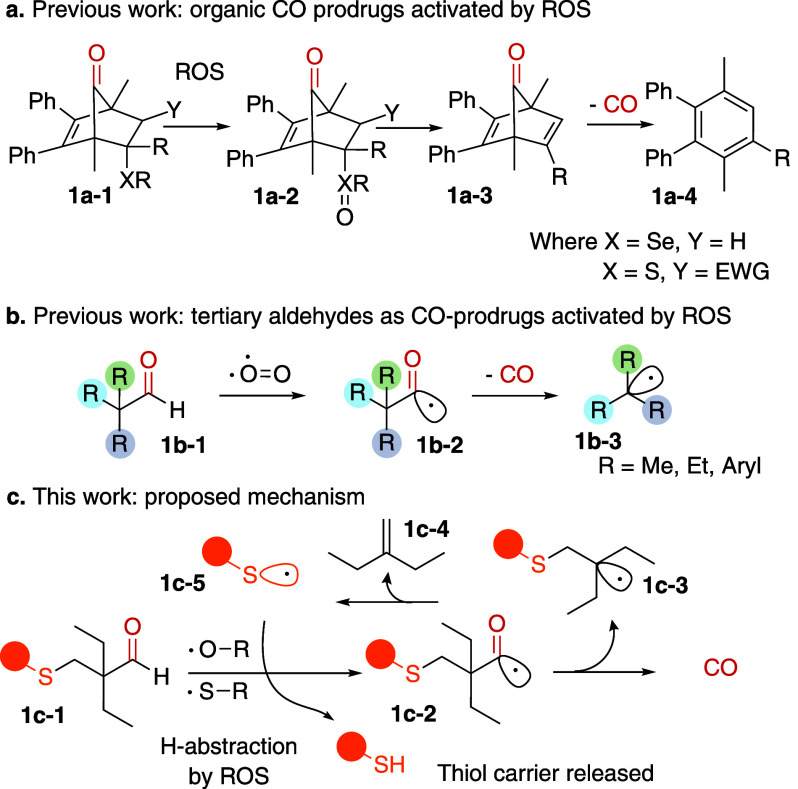
**a, b.** Selected examples of ROS-activated CO-prodrugs described in previous literature. EWG, electron-withdrawing group. **c.** Overview of this work. After initial hydrogen abstraction and CO release, the resultant stabilized radical **1c-3** would undergo a *retro*-thiol-ene uncaging reaction to release **1c-4** and a thiol carrier **1c-5**.

Another ROS-triggered CO release mechanism has been reported using tertiary aldehydes such as 4-ethyl-4-formyl-hexanenitrile and 2-methyl-2-phenylpropanal **1b-1** (Figure 1b).^46^ CO release was observed in the presence of oxygen; however, it was acknowledged that the release from these compounds is slow, and under mild physiological conditions, significant release occurred only at very high concentrations, when microdroplets formed, yielding high local radical concentrations. The reaction proceeds via an initial hydrogen abstraction from aldehyde **1b-1**, resulting in the corresponding acyl radical **1b-2**, which subsequently undergoes decarbonylation to form the tertiary radical **1b-3** along with CO release. This process is endothermic with enthalpic activation barriers that limit the rate of CO liberation under physiological conditions.^47^ However, the release of CO as a gas introduces a significant entropic benefit, which can shift the overall Gibbs free energy (Δ*G*) toward a more favorable, slightly exergonic process. These compounds have demonstrated therapeutic benefits in treating rheumatoid arthritis in mice.^48,49^

We envisioned that the reactivity of such acyl radicals toward CO release could be improved by introducing a reactive thioether moiety at the γ-position (**1c-1**, Figure 1c), in such a way that the tertiary radical formed upon CO release **1c-3** would undergo a subsequent *retro-*thiol-ene uncaging to release 2-ethyl-1-butene **1c-4** and a thiol **1c-5**, where the thiol can be a drug or a carrier. Similar types of thiol-enes were used in thiol uncaging reactions before.^50^ Interestingly, for this mechanism, the H-abstraction could be initiated either by a reactive oxygen species in the cell or possibly proceed via a radical chain, with the released thiol abstracting the aldehyde hydrogen. We hypothesized that the steric bulk provided by ethyl groups should protect the aldehyde from undesired nucleophilic attacks, while radical stabilization, alongside entropy gain, should drive the decarbonylation.

## Results and Discussion

### Synthesis

We have developed a synthetic route to these CO-prodrugs and applied it to a few thiol carriers (Figure 2). Briefly, the three-step synthesis starts with a single tosylation of diol **1**, followed by Swern oxidation of the resulting alcohol to an aldehyde, and an S_N_2-type reaction to substitute tosyl with the desired thiols **4a-d**. Several thioether compounds were synthesized using small bioactive molecules such as *N*-acetylcysteine (NAC) **4a**, its methyl ester (NAC-Me) **4b**, 7-mercapto-4-methylcoumarin **4c**, and mercaptopurine **4d** (Figure 2). Given the poor aqueous solubility of aromatic-thiol-containing CO-prodrugs, we focused on the nontoxic cysteine-bearing **4a-b**, due to their good water solubility, the versatility of cysteine as a building block, and shown CO/NAC synergistic evidence of anti-inflammatory effects in the literature.^51^

**Figure 2:**
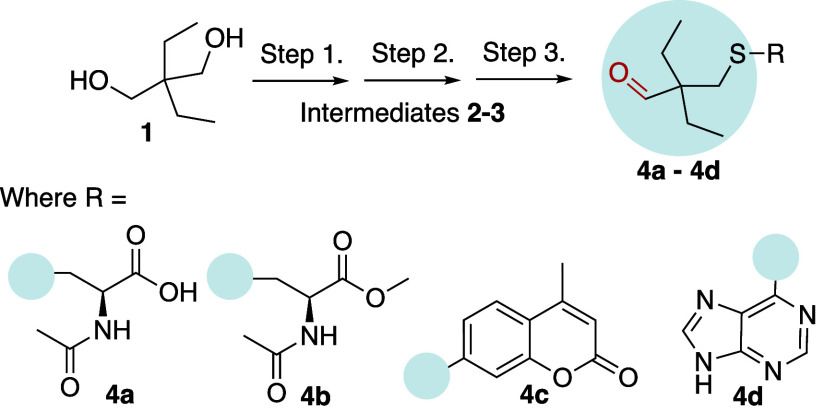
Schematic synthesis of aldehyde prodrugs **4** include single tosylation (Step 1), Swern oxidation (Step 2), and S_N_2 substitution of tosyl to desired thiol (R-SH, Step 3). Example final compounds synthesized are **4a-4d**.

### Verification of ROS-Triggered CO Release

Having established an efficient route to CO-releasing building blocks (**4a-d**), we investigated the proposed radical-triggered release of CO. First, we were able to detect sufficient CO release using a CO-monitor from CO-carrier molecules **4a** and **4b** when exposing them to radical triggers, such as hydroxyl, *tert*-butoxyl, and peroxyl radicals (Figure S1). Further, we confirmed the release of CO from **4a** using a previously described two-compartment myoglobin assay (Figure S2).^52^

Due to the high reactivity of hydroxyl radicals, the quantitative determination of the reaction kinetics can become very complex.^40^ Hence, we were interested in following the CO release in physiological conditions qualitatively. We applied a Fenton-like reaction using Mn^2+^ as a catalyst to allow for constant exogenously triggered radical generation in a physiological-like medium (pH 8.0, Figure 3a).^53,54^ This allowed us to monitor aldehyde **4a** conversion by ^1^H NMR and infer that the rates of **4a** conversion and CO evolution (measured by CO-monitoring) are proportional and depend solely on the radical-like ROS generation (Figures 3a and S3). Identifying all of the products formed in this reaction was difficult due to competing reaction paths involving excess hydrogen peroxide (H_2_O_2_) and hydroxyl radicals. Thus, we confirmed the formation of NAC-products and 2-ethyl-1-butene **1c-4** under milder radical-generating conditions (i.e., using the radical initiator azobis­(isobutyronitrile), AIBN) by ^1^H NMR, diffusion-ordered ^1^H NMR spectroscopy (DOSY), GC-MS, and LC-MS (Figures 3b and S4–S7).

**Figure 3:**
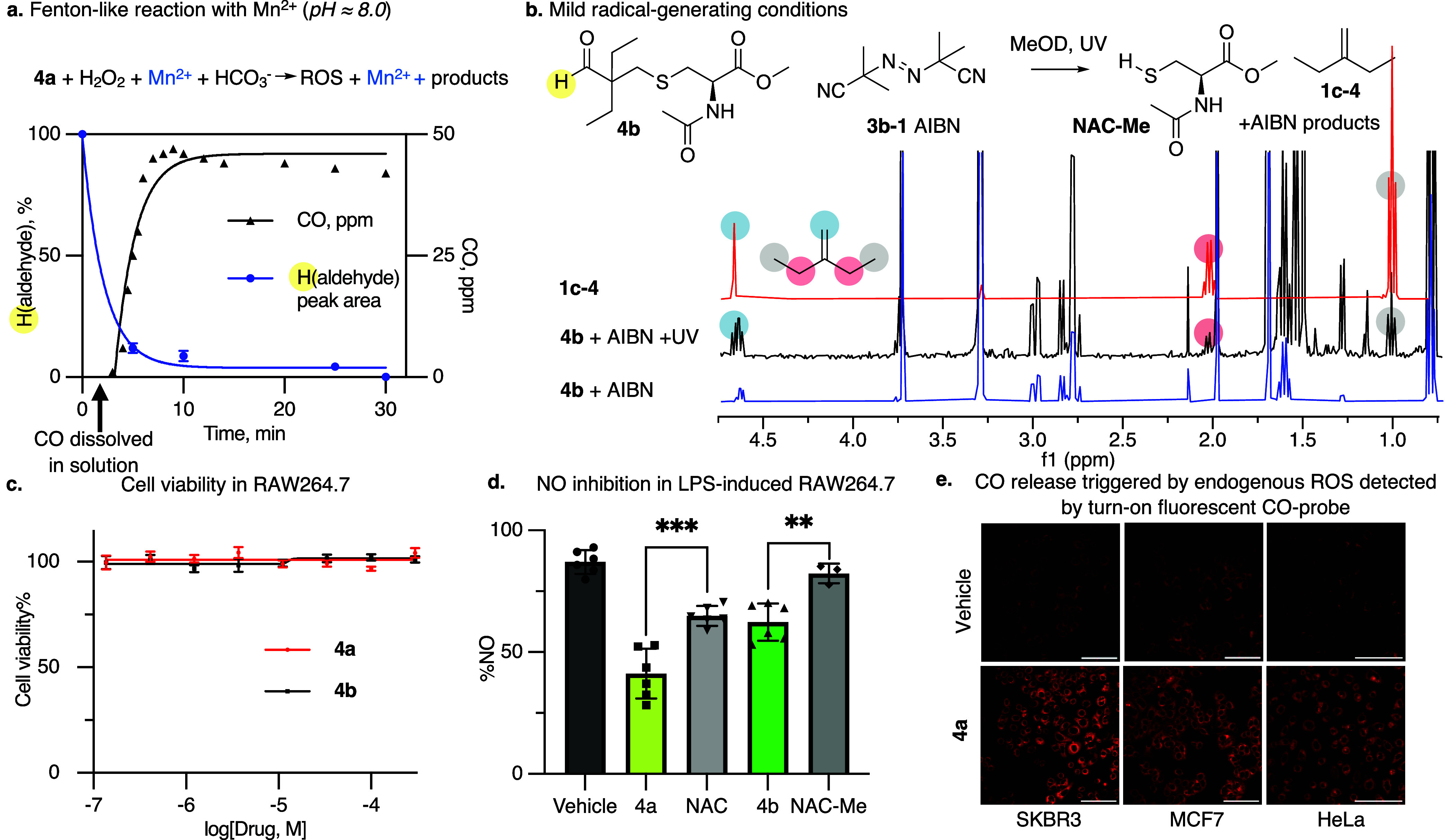
**a.** Aldehyde **4a** conversion monitoring by ^1^H NMR in Mn^2+^ catalyzed Fenton-like conditions infer that the rates of **4a** conversion and CO evolution (CO-monitor response) are proportional and depend solely on the ROS generation. **b.** ^1^H NMR spectra of **4b** in the presence of a radical starter azobis­(isobutyronitrile) (AIBN) with or without UV treatment confirmed the radical-dependent formation of 2-ethyl-1-butene **1c-4**. **c.** Cytotoxicity studies of CO-prodrugs **4a-b** in RAW264.7 shown no toxicity up to 300 μM. **d.** Effect of **4a-b** and their *N*-acetylcysteine (**NAC**) counterparts at 100 μM on the inhibition of NO production (% control) in lipopolysaccharide (LPS)-induced RAW264.7 cells. Statistically significant differences found using unpaired *t* test and marked as *** (*p* ≤ 0.0005), ** (*p* ≤ 0.005), and dots represent different independent experiments **e.** Confocal microscopy images for cellular endogenous ROS-triggered CO release in untreated (vehicle control, top panel) and treated SKBR3, MCF7, or HeLa cells (50 μM **4a**, bottom panel). After an initial 30 min pretreatment with 5 μM 1-Ac CO probe, **4a**/vehicle was added, and after 30 min of incubation cells were fixed, and images were acquired. Increase in fluorescence represents the turn-on response of the 1-Ac CO probe (λ_ex_ = 561 nm, λ_em_ = 570–620 nm). Scale bar represents 100 μm.

Our next goal was to assess the chemical stability of the CO-carrier molecules **4a** and **4b** under physiological conditions in the absence of radicals. We incubated **4a** and **4b** in phosphate-buffered saline (PBS, pH 7.4) and human blood serum at 37 °C and did not observe significant decomposition after 48 h (Figure S8).

### CO Release in Cellular Models

Next, we evaluated cytotoxicity and whether CO can be released in cells from **4a-b.** Compounds **4a-b** showed no toxicity up to 300 μM in RAW264.7 (Figure 3c), HeLa, and HEK293T cell lines (Figure S9), while the anti-inflammatory effects of **4a** and **4b** in lipopolysaccharide (LPS)-induced RAW264.7 were enhanced compared to NAC alone (Figures 3d and S10). The release of endogenous ROS-triggered CO in cells was confirmed by detecting increased turn-on fluorescence of the CO-probe 1-Ac^55^ in three different ROS-rich cancer cell lines (SKBR3, MCF7, HeLa; Figures 3e and S11–S13), while CO release in the low-ROS macrophage cell line RAW264.7 was detected only when ROS-enhancing pro-inflammatory factors were introduced (Figure S14).

### *In Vivo* Study

Many studies have shown that low-dose CO has a therapeutic effect in cancer, with immunostimulatory potential.^14–16^ Given that CO-prodrug **4a** carries ROS-quencher NAC, we explored whether similar effects could be observed in animal models. Treatment with **4a** effectively attenuated tumor growth and prolonged survival in the MC-38 syngeneic model of colon carcinoma in C57BL/6 mice, relative to **NAC** or vehicle controls (Figure 4). Based on mouse weight measurements, the treatments were well tolerated (Figure S15), further highlighting the potential of this CO-releasing system.

**Figure 4:**
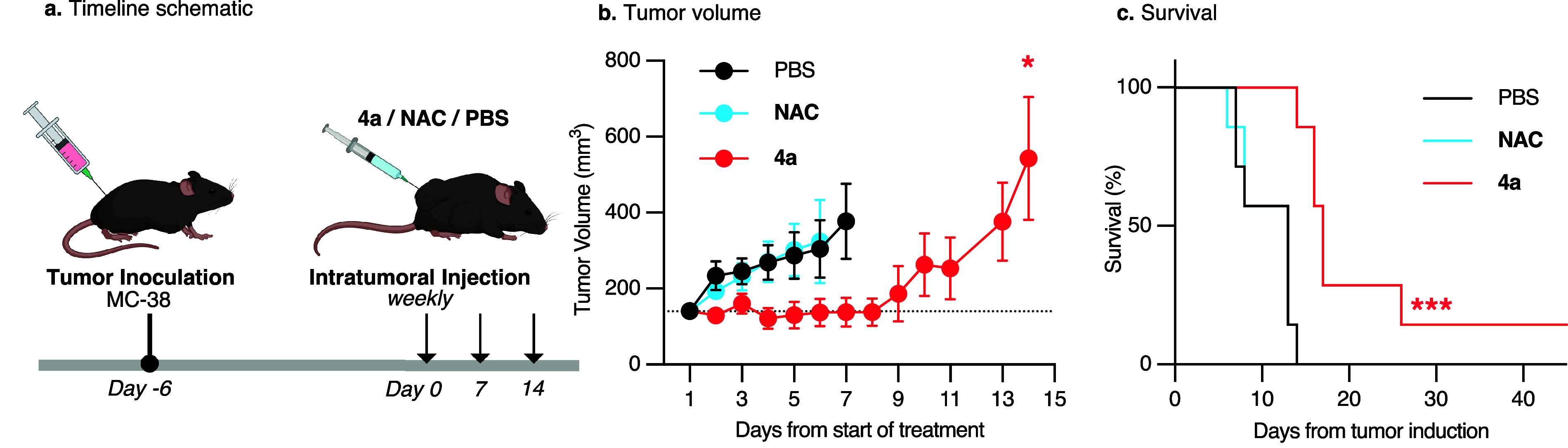
**a.** Representation of the timeline of MC-38 tumor cell inoculation and CO-prodrug therapy. **b.** Tumor growth curve. Data are represented as mean ± SEM (*n* = 7). A two-way ANOVA indicates a significant effect of time versus treatment. **c.** Overall survival over time (*n* = 7). A log-rank test indicates a statistically significant difference between the **4a** treatment and both the **NAC** and PBS controls. Statistically significant differences are marked as *** (*p* ≤ 0.0005), * (*p* ≤ 0.05).

### Mechanistic Study Using Quantum Mechanical Calculations

Once confirmed that aldehydes **4a** and **4b** can release CO in mild, physiological, and in-cell conditions, we evaluated the proposed reaction mechanism using quantum mechanics (Figures 5 and S16–S26). Starting from aldehyde **A**, which bears a methylsulfane group as an abbreviated model of the cysteine moiety, hydrogen abstraction by a hydroxyl radical (HO·), a likely ROS generated during the reaction, has a relatively low activation energy (Δ*G* ^‡^_TS1_ ∼ 7 kcal mol^–1^). This step is quite exergonic (Δ*G* ∼ −30 kcal mol^–1^) due to the formation of a water molecule and the intrinsic stability of radical aldehyde **B**, as the unpaired electron is delocalized along the carbonyl group and the adjacent C–C bond. The subsequent decarbonylation reaction has a slightly higher activation energy (Δ*G* ^‡^_TS2_ ∼ 9 kcal mol^–1^), albeit relatively low as well, and leads to carbon-centered radical **C**. As previously reported in the literature,^47^ this process is intrinsically endothermic (Δ*H* = +8.1 kcal mol^–1^), with the release of CO as a gas making it an entropically favorable and therefore a slightly exergonic process (Δ*G* = −4.6 kcal mol^–1^). This is because radical **C**, although stabilized by hyperconjugation with the surrounding alkyl groups, is highly localized, in contrast to the delocalized (i.e., stabilized) character of acyl radical **B** (Figure 5c). Finally, the *retro*-thiol-ene reaction has a slightly lower activation energy (Δ*G* ^‡^_TS3_ ∼ 8 kcal mol^–1^) generating the corresponding thiol-radical (**MeS·**) and 2-ethyl-1-butene (**D**) as reaction products. Therefore, calculations suggest that the reaction is perfectly feasible under mild conditions due to the low activation energies of all the involved steps (Δ*G* ^‡^ ∼ 7–9 kcal mol^–1^); also, it corroborates the initial hypothesis that the final *retro*-thiol-ene step makes the radical process even more thermodynamically favored, i.e., more exergonic, even before the thiyl-radical recombination/annihilation expected to take place at the end of the global process.

**Figure 5:**
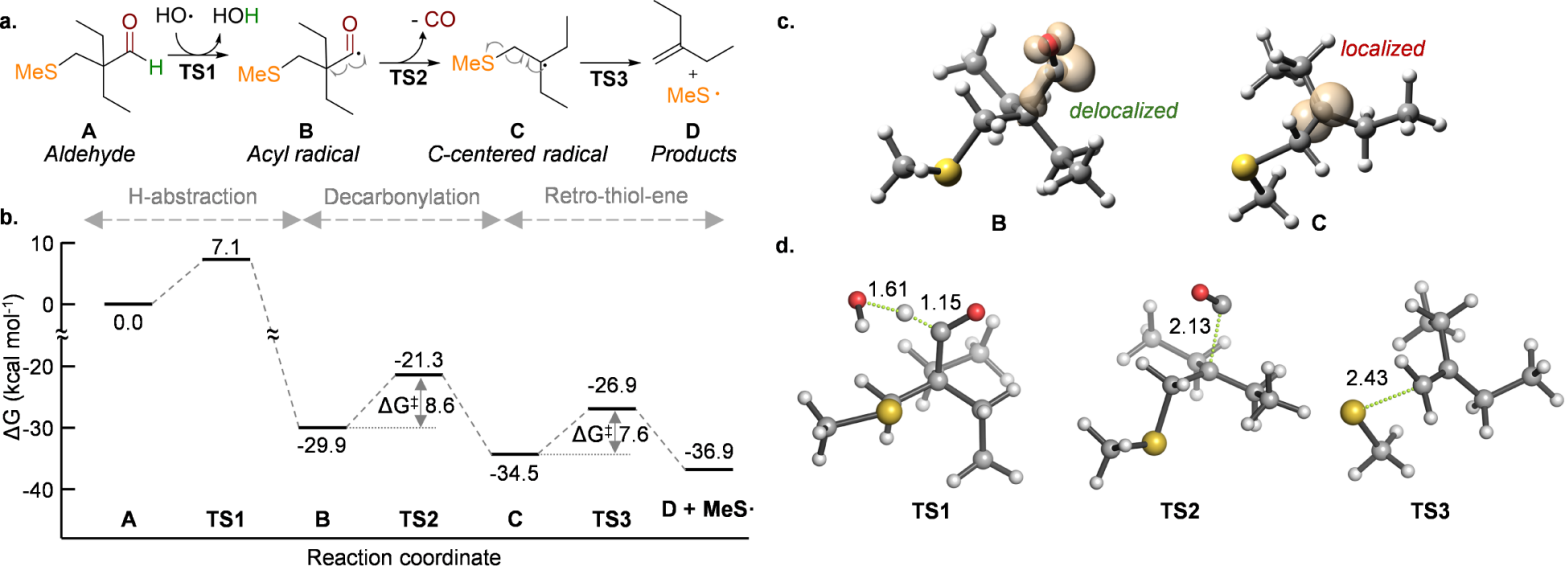
**a.** Whole radical reaction mechanism and **b.** minimum energy pathway calculated with PCM­(H_2_O)/M06-2*X*/6-311+G­(2d,p) for the H-abstraction, decarbonylation, and *retro-*thiol-ene reactions from tertiary aldehyde A. **c.** Lowest-energy structures and spin density plots of radicals B and C calculated at the same level of theory. **d.** Lowest-energy transition structures for the H-abstraction (TS1), decarbonylation (TS2), and *retro*-thiol-ene (TS3) reactions. Breaking bonds are represented with green dotted lines. Distances are given in angstrom.

### Exploration of CO-Prodrug Alternatives

We also explored additional CO-prodrug alternatives. We predicted that an analogue-bearing methoxy groups instead of ethyl groups at the quaternary carbon would have a lower activation energy for the decarbonylation step (Figures S21 and S24); however, any attempts to synthesize it resulted in unfruitful perhaps due to its higher reactivity. We were able to synthesize a less bulky analogue-bearing methyl instead of ethyl groups, although it showed higher cytotoxicity. Finally, a selenocysteine analogue, in principle a better leaving group, turned out to be unstable to oxidation, showing low reaction yields and no improvement over **4a** (Figure S27).

Thioether oxidation to sulfoxide by H_2_O_2_ proceeds slowly under near-physiological conditions but is markedly accelerated in the presence of other more reactive ROS.^56^ Further calculations revealed very similar reaction profiles for sulfinyl and sulfonyl derivatives of aldehyde **A**, suggesting a highly consistent mechanism irrespective of the oxidation state of sulfur under high ROS conditions (Figures S22–S25). Finally, competing H-abstraction reactions were discarded due to the calculated instability of α-S-alkyl radicals compared with acyl radical **B** (Figure S26).

### CO-Prodrug in Solid-Phase Peptide Synthesis

Having established *N*-acetylcysteine CO-prodrugs **4a-b** as a viable construct for activation in ROS-rich cellular environments found in cancers, we focused on the conjugation of CO-prodrugs to a tumor-targeting antibody as a carrier for tumoral delivery. Since a single CO molecule per high-molecular-weight protein conjugate would likely be insufficient for therapeutic efficacy, we explored strategies to increase CO loading. We proposed that the cysteine scaffold may facilitate the preparation of payloads for protein conjugation that contain multiple CO-releasing units using solid-phase peptide synthesis (SPPS). We followed a synthetic approach similar to that shown in Figure 1 (Scheme S8) to yield Fmoc-protected building block **4e** for use in SPPS (Figure 6a). We demonstrated the compatibility of SPPS with this unprotected aldehyde by synthesizing a peptide containing both an aldehyde and reactive residues such as lysine and tyrosine (Figure S28). Next, we proceeded with the synthesis of peptide **5** using SPPS, incorporating three CO-prodrugs within a single peptide via glycine spacers. A maleimide bioconjugation handle was introduced in the final coupling step (Figure 6a). The methionine-bearing negative control **7** was chosen due to the thioether functionality in methionine (Met), which lacks CO-releasing properties.

**Figure 6:**
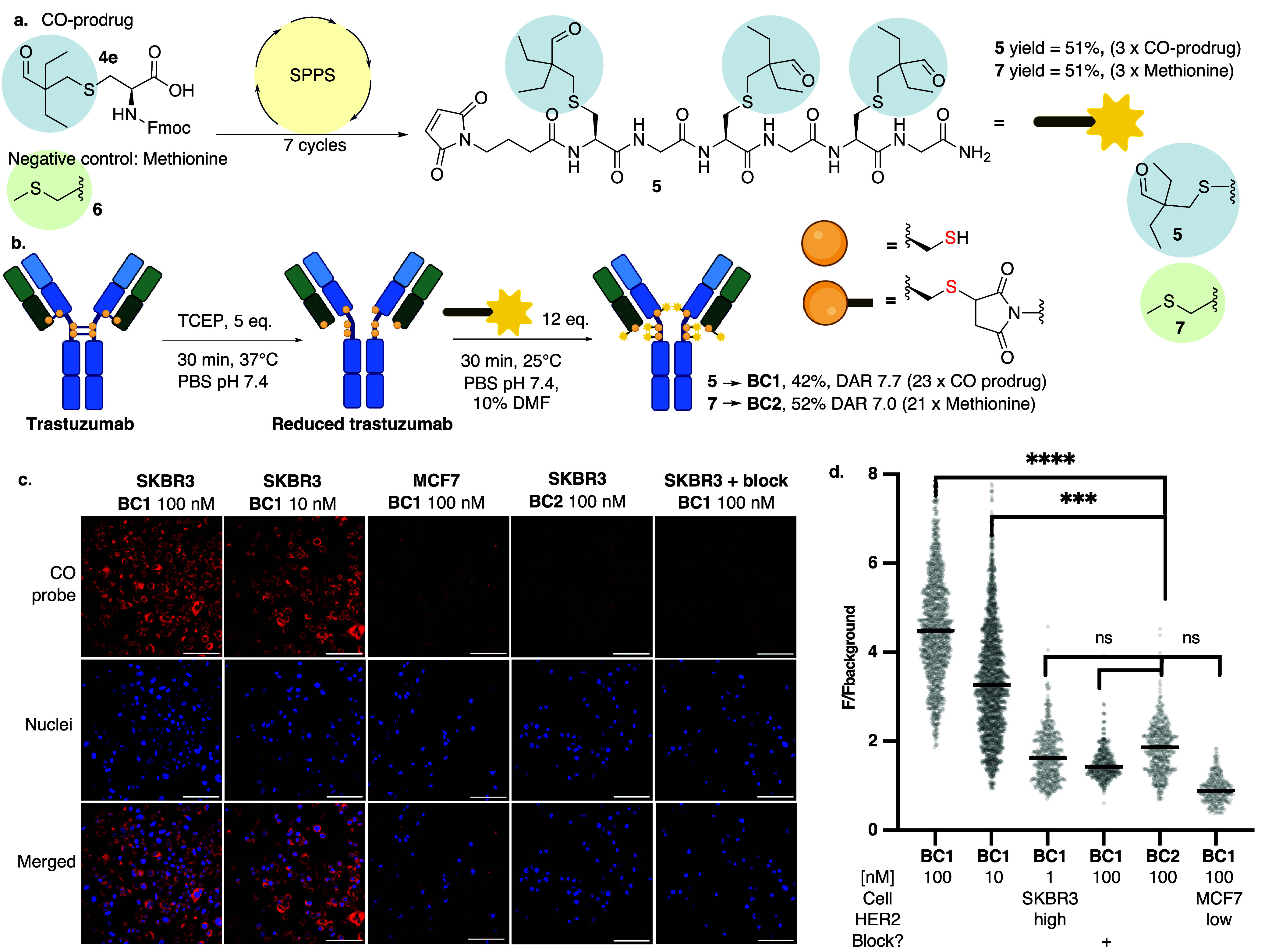
**a.** Schematic representation of the synthesis of a maleimide bioconjugation handle containing CO-prodrug **5** and negative control **7** using solid-phase peptide synthesis (SPPS). **b.** Schematic representation of the bioconjugation of trastuzumab using interchain disulfides. **c.** Confocal microscopy images for cellular CO release in SKBR3 (HER2-high) or MCF7 (HER2-low) cells treated with **BC1** (trastuzumab carrying CO-prodrugs) or **BC2** (negative control). After an initial 30 min treatment with a 5 μM 1-Ac CO probe, **BC1** or **BC2** was added. Following 2 h incubation, cells were fixed and imaged using DAPI for nuclei (blue) and 1-Ac turn-on CO probe (red, λ_ex_ = 561 nm, λ_em_ = 570–620 nm) for intracellular CO release. White bar represents 100 μm. **d.** Fluorescence quantification in images in **c.** Turn-on 1-Ac CO probe fluorescence was significant when HER2-high SKBR3 cells were treated with 100 and 10 nM while no significant turn-on fluorescence was observed in HER2-low MCF7 cells. Pretreatment of SKBR3 cells with nonfluorescent trastuzumab (i.e., receptor blockade) abolished the fluorescence response following 100 nM **BC1** incubation. Statistically significant differences found after comparing whole populations using unpaired *t* test and marked as **** (*p* ≤ 0.00005), *** (*p* ≤ 0.0005), and ns (*p* > 0.05). TCEP, tris­(2-carboxyethyl)­phosphine; PBS, phosphate-buffered saline; and Met, methionine.

### Bioconjugation of CO-Prodrug to Therapeutic Antibodies

In the next step, we successfully conjugated maleimide-bearing payloads **5** and **7** to trastuzumab, a clinically used internalizing human monoclonal antibody that targets human epidermal growth factor receptor 2 (HER2), which is overexpressed in certain types of cancer. The resulting conjugate **BC1** had an average drug-to-antibody ratio (DAR) of 7.7, corresponding to 23 CO-prodrug molecules per antibody, in an overall yield of 46% (Figures 6b and S29). Importantly, the bioconjugate retained its binding affinity for HER2 (Figure S30). Stability studies confirmed that the conjugate remained intact for at least 48 h under physiological conditions (pH 7.4), as verified by LC-MS analysis. No CO release or intramolecular aldehyde condensation was observed during this period (Figure S31).

### Targeted CO Release in a Cellular Model

Lastly, we demonstrated that carbon monoxide can be selectively released in HER2-expressing cells using SKBR3 and MCF7 as high- and low-HER2 cell lines, respectively, in combination with a 1-Ac turn-on CO probe.^55^ To determine receptor saturation levels, we first assessed HER2 binding under identical conditions (2 h, 100–0.1 nM) using a trastuzumab–Alexa Fluor 488 conjugate. The results indicated that HER2 receptors approach saturation at approximately 10 nM within 2 h (Figures S32 and S33). We then performed imaging studies using the 1-Ac CO probe. A significant increase in turn-on fluorescence (*p* ≤ 0.0005) was observed in **BC1**-treated cells at both 100 and 10 nM concentrations, whereas a low fluorescence signal was detected under negative control conditions, including treatment of methionine-bearing **BC2**, **BC1** treatment after HER2 receptor blockade, and experiments using the HER2-low MCF7 cell line (Figures 6c,d and S34).

Although the full potential of this system can only be validated through *in vivo* studies using humanized mouse models, careful selection and characterization of the overexpressed receptor are essential first steps, given the complex and context-dependent biological roles of CO, particularly in oncology. The availability of both HER2-high and HER2-low cell lines, combined with the established clinical use of high-DAR trastuzumab conjugates (i.e., Enhertu DAR7.7),^57^ enabled us to deliver CO to the cells at concentrations within the reported detection limits of the turn-on fluorescent probe selected (limit of 50 nM for 1-Ac).^55^ To the best of our knowledge, this represents the first example of an organic CO-prodrug site-specific bioconjugation to a specific protein carrier.

## Conclusion

In summary, we successfully demonstrated the use of thiol-ene-caged tertiary aldehydes for CO release *in vitro*. The CO-prodrugs readily prepared from commercial starting materials are water-soluble, nontoxic, and stable in human serum. These metal-free CO-prodrugs selectively release CO in response to both exogenous and endogenous radical-like ROS. These prodrugs alone effectively prolonged the survival of the tumor-bearing mice. Furthermore, since the rate of the release depends solely on ROS levels, our CO-prodrugs are well-suited for conjugation to tumor-targeting, long half-life protein carriers, such as trastuzumab.

To the best of our knowledge, this represents the first example of a CO-prodrug conjugated to proteins, with a proof of concept for receptor-targeted delivery demonstrated in HER2-positive cell lines. The CO-prodrugs described herein hold significant potential as tools for future studies exploring therapeutic applications of CO.

## Supplementary Material



## Notes

Additional figures, detailed methods and characterization, and additional data (PDF)

## Abbreviations

CO: carbon monoxide
CORM: carbon monoxide releasing molecules
HER2: human epidermal growth factor receptor 2
NAC: *N*-acetylcysteine
ROS: reactive oxygen species
SPPS: solid-phase peptide synthesis
