# Supplementary material for: Organic Carbon Monoxide Prodrugs Activated by Endogenous Reactive Oxygen Species for Targeted Delivery
Source: J Am Chem Soc. 2025 Jul 3;147(28):24691–8. doi: 10.1021/jacs.5c05952 (PMC12272688; doi:10.1021/jacs.5c05952)
Supplement: Supplementary file 1 [file ja5c05952_si_001.pdf]

Supporting Information

Organic Carbon Monoxide Prodrugs Activated by Endogenous Reactive Oxygen Species for Targeted Delivery

Inga Cernauskiene,[1] Claudio D. Navo,[2] Carlos Labão-Almeida,[3] Rupert S. J. Proctor, [1] Bengt H. Gless,[1] Wei Ting Khaw,[1] Cong Tang,[3,4] M. Milagros Muriel-Olaya,[2] Gonzalo Jiménez-Osés,[2,5] Gonçalo J. L. Bernardes[1,3,6*]

[1] Yusuf Hamied Department of Chemistry, University of Cambridge, Lensfield Road, Cambridge, CB2 1EW, UK.
[2] Center for Cooperative Research in Biosciences (CIC bioGUNE), Basque Research and Technology Alliance (BRTA), Derio 48160, Spain.
[3] GIMM - Gulbenkian Institute for Molecular Medicine; Avenida Prof. Egas Moniz, 1649-028, Lisboa, Portugal.
[4] Xi'an Fengcheng Hospital, No.9 Fengcheng Third Road, Xi'an, Shaanxi, China
[5] Ikerbasque, Basque Foundation for Science, 48013 Bilbao, Spain.
[6] Translational Chemical Biology Group, Spanish National Cancer Research Centre 26 (CNIO), Madrid 28029, Spain

*Correspondence should be addressed to G.J.L.B.:
Tel. +44 1223 336305; Email: gb453@cam.ac.uk

Contents

1 Supplementary Figures *5*

Figure S1. Verification of CO release from 4a using a CO-meter 5

Figure S2. Verification of CO release from 4a by myoglobin assay 6

Figure S3. Decomposition of 4a in Fenton-like conditions by ^1H NMR 7

Figure S4. Decomposition of 4b in mild radical conditions by ^1H NMR 8

Figure S5. Decomposition of 4b in mild radical conditions ^1H NMR DOSY 9

Figure S6. Decomposition of 4b in mild radical conditions by UPLC-MS........ 10

Figure S7. 2-ethyl-1-butene detection in decomposition products of 4b by GC-MS........ 11

Figure S8. Stability of 4a-b in PBS (pH 7.4) and human serum 12

Figure S9. Cell viability assay for compounds 4a and 4b for RAW264.7, HeLa and HEK293T cells 13

Figure S10. TNF-α and iNOS suppression by 4a and 4b in LPS-induced RAW264.7 14

Figure S11 CO release in SKBR3 triggered by endogenous ROS 15

Figure S12 CO release in HeLa triggered by endogenous ROS........ 16

Figure S13 CO release in MCF7 triggered by endogenous ROS........ 17

Figure S14 CO release in live RAW264.7 imaged by COP-1 under ROS induction by lipopolysaccharides (LPS) 18

Figure S15 Weight variation in tumor-bearing mice........ 19

Figure S16 QM study: Labelling of ethyl substituent compounds for calculations 20

Figure S17 QM study: Labelling of methoxy substituent compounds for calculations........ 20

Figure S18 QM study: Labelling of sulfinyl compounds for calculations........ 21

Figure S19 QM study: Labelling of sulfonyl compounds for calculations........ 21

Figure S20 QM study: Comparison of geometries and spin densities of intermediate radicals and transition states 22

Figure S21 QM study: Minimum-energy pathway for the methoxy-substituted compounds 23

Figure S22 QM study: Minimum-energy pathway for sulfoxide derivatives 24

Figure S23 QM study: Minimum-energy pathway for sulfone derivatives........ 24

Figure S24 QM study: Intrinsic Reaction Coordinates for thioether derivatives 26

Figure S25 QM study: Intrinsic Reaction Coordinates for sulfoxide and sulfone derivatives........ 27

Figure S26 QM study: instability of α-S-alkyl radicals........ 28

Figure S27 Alternative building blocks synthesised 29

Figure S28 Aldehyde stability towards intramolecular biological nucleophiles – intramolecular reaction 30

Figure S29 Peptide Synthesis and Bioconjugation to Trastuzumab 31

Figure S30 BC1 binding to the receptor 32

Figure S31 Stability of BC1 in PBS 33

Figure S32 Bioconjugate control – Alexa Fluor™ 488 C_5 maleimide 34

Figure S33 Receptor saturation 35

Figure S34 CO release from Bioconjugates – extended figure 37

2 Experimental procedures 38

2.1 General experimental 38

2.2 Characterisation 39

2.3 Chemical synthesis 41

2.3.1 Synthesis of the main compounds 41
2.3.2 Synthesis of the supporting compounds 46

2.4 Peptide synthesis 49

2.4.1 General peptide synthesis protocol 49
2.4.2 Peptide synthesis and characterisation 49

2.5 Verification of CO release vitro, product detection and stability 52

2.5.1 House-hold CO-meter assay 52
2.5.2 Myoglobin assay 52
2.5.3 CO release in Fenton-like conditions 52
2.5.4 CO release in mild oxidising conditions 53
2.5.5. Stability assays 54

2.6 Quantum mechanical calculations. 55

2.7 Bioconjugation reactions to IgG (immunoglobulin G) antibodies 57

2.7.1 **BC1** - Trastuzumab CO conjugate 57
2.7.2 **BC3** - Trastuzumab LC-V205C- Alexa Fluor 488 57
2.7.3 Bioconjugate stability 57
2.7.4 Biolayer interferometry (BLI) 58

2.8 Cell experiments 59

2.8.1 Cell viability assays 59
2.8.2 LPS stimulation of RAW264.7 cells and nitrite quantification by Greiss assay 59
2.8.3 LPS stimulation of RAW264.7 cells and TNF-α quantification by Western blot 59
2.8.4 Cell imaging 60
2.8.5 Flow cytometry 62

2. 9 Animal studies 63

3 ^{1}H and ^{13}C NMR spectra 64

3.1 Spectra of the main molecules 64

2.2 Spectra of the Supporting Information (SI) molecules 71

4 Protein LC-MS spectra ***79***
Unconjugated Trastuzumab 79
BC1 Trastuzumab-**5** conjugate 80
BC2 Trastuzumab-**7** conjugate 81
Unconjugated Trastuzumab-LC-V205C 82
BC3 Trastuzumab-LC-V205C AlexaFluor 488 nm 83
5 Uncropped gels ***84***
6 Additional Computational Data ***86***
7 References ***93***

1 Supplementary Figures

Figure S1. Verification of CO release from **4a** using a CO-meter

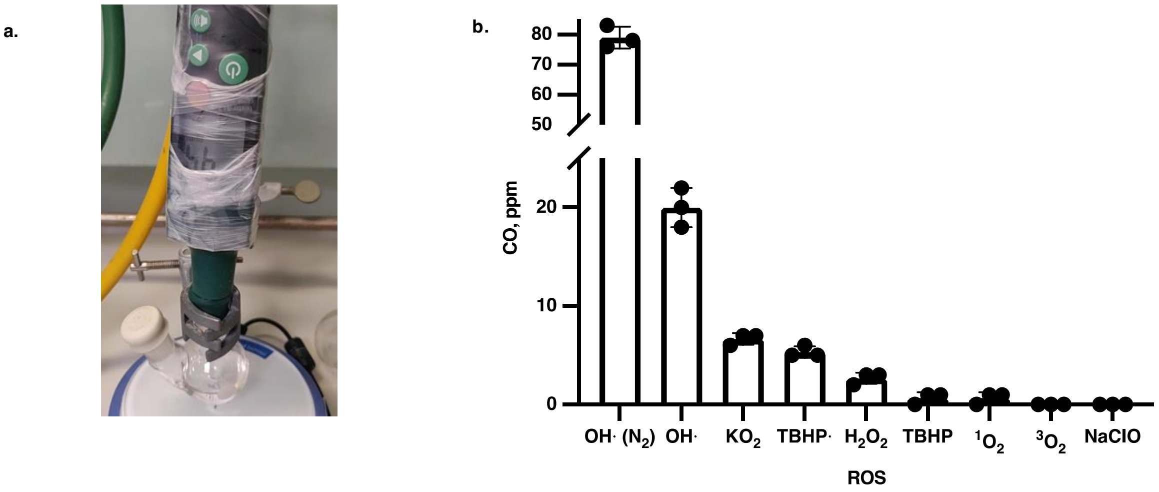

Figure S- 1. ***a.*** *A household CO meter was attached to a two-necked flask to provide a rough estimation what specifically triggers CO release from compound* ***4a*** *in water at ambient temperature under non-inert conditions.* ***b.*** *The CO meter detected CO release from* ***4a*** *only after radical-like reactive oxygen species (ROS) were introduced. When the Fenton reaction (producing OH•) was performed under oxygen-free conditions, the CO yield was approximately four times higher, suggesting that the initial experiment lacked pH control (due to $Fe(OH)_3$ formation) and that ROS were quenched by atmospheric oxygen. This experiment illustrates that radical species can be quenched by air dioxygen, and that CO monitor readings provide only rough estimates. Accurate carbon monoxide detection requires more sensitive instruments, such as gas chromatography with a reducing compound photometer or a thermal conductivity detector, which were not available onsite. The experiment was performed in triplicate.*

Figure S2. Verification of CO release from **4a** by myoglobin assay

a.	Reagent	[Stock]	V	[Final]
Outer vial, 1mL	Myoglobin in PBS	1.1 mg/mL	900 μL	1 mg/mL (60 μM)
	Sodium dithionite in PBS	10 mg/mL	100 μL	1 mg/mL
Inner vial, 1 mL	CO-prodrug in MeCN	20 mM	100 μL	2 mM
	MQ water	-	780 μL	-
	H_2O_2 in water	100 mM	100 μL	10 mM
	$FeSO_4$ in MQ-water (or MQ-only –ve control)	100 mM	20 μL	2 mM

b.

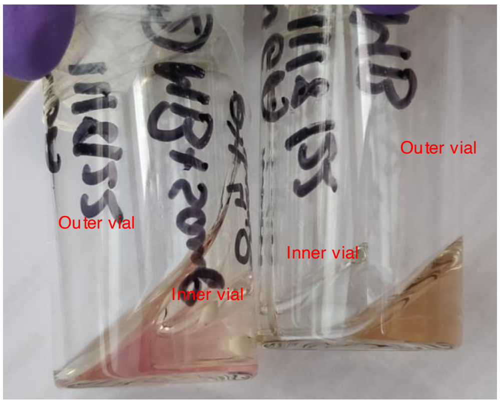

c. d.

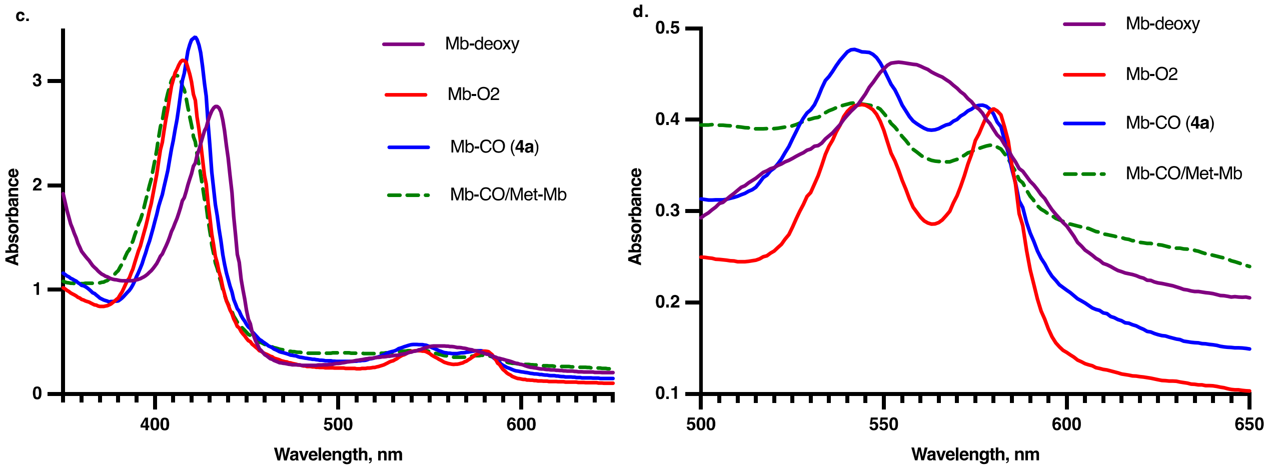

*Figure S- 2 Verification of CO release from **4a** using myoglobin (Mb) assay. **a.** Experimental conditions. **b.** The setup included two glass vials. **c.** Myoglobin bound to CO released from **4a** is shown in blue; myoglobin bound to oxygen (O_2) is shown in red; and the negative control (deoxy-myoglobin) is shown in purple. Note: After prolonged incubation, myoglobin is oxidized to met-myoglobin (dotted line). **d.** Enlarged spectra of myoglobin in the 500–650 nm region. Deoxy-myoglobin typically exhibits a single absorption maximum around 560 nm, while CO-myoglobin displays two maxima in this region. A similar response was observed for compound **4b** (data not shown). V = volume.*

Figure S3. Decomposition of **4a** in Fenton-like conditions by ^1H NMR

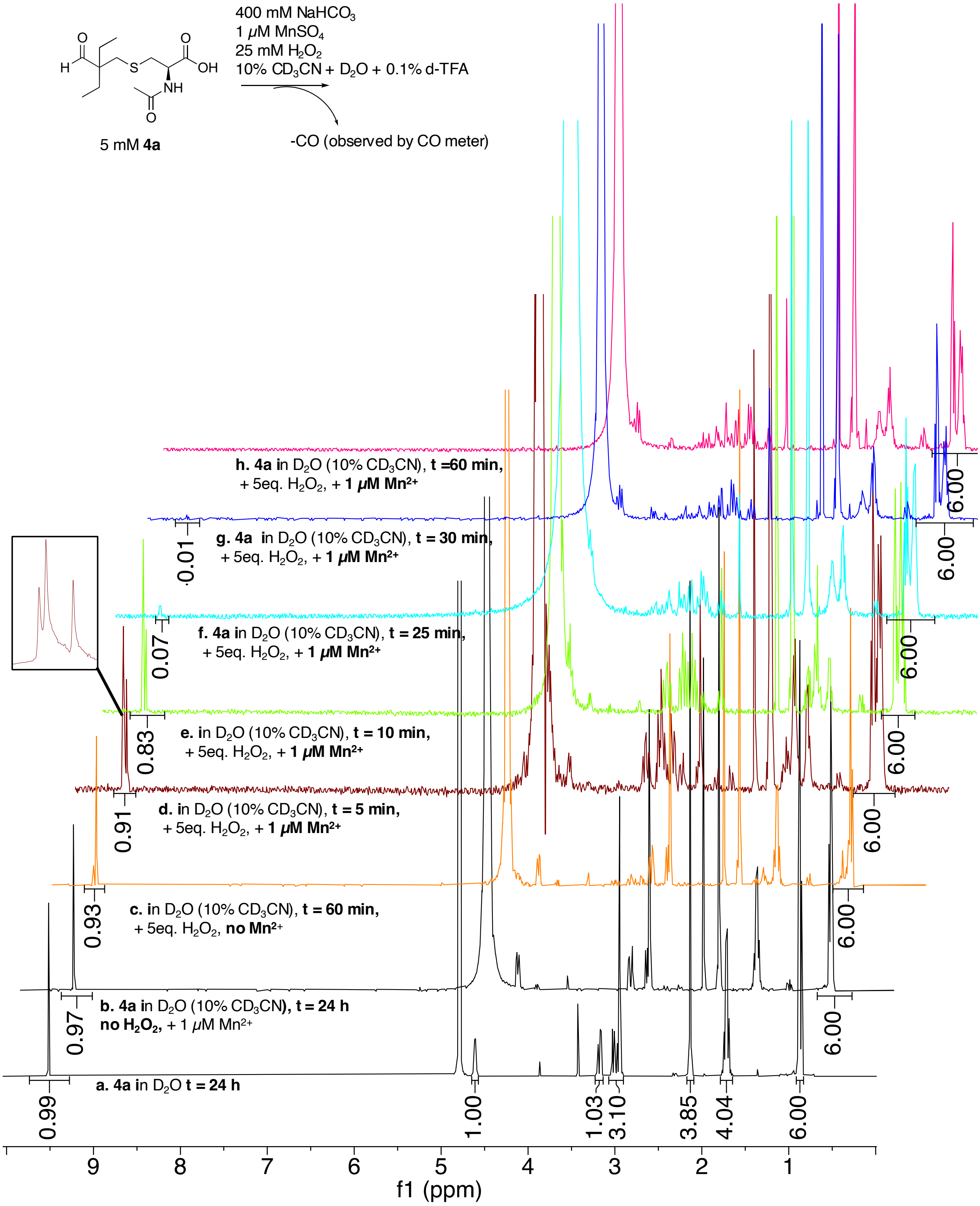

*Figure S- 3 CO prodrug **4a** is stable in D_2O or in D_2O when Mn^{2+} is present (**a-b**), however, sulfur is oxidized to sulfoxide and sulfone in the presence of H_2O_2 hence three aldehyde-H signals are observed after H_2O_2 is added (**c.** confirmed by LC-MS of mass addition +16, +32, not shown). When both H_2O_2 and Mn^{2+} are added, this results in Fenton—like reaction generating radical species capable of abstracting the aldehydic hydrogen and thereby triggering CO release. Different timepoints of this reaction are shown in **d-h**. All three aldehyde signals at 9.46 ppm disappears in Fenton-like reaction, suggesting products of sulfur oxidation also undergo decarbonylation. Isolation of pure oxidised sulfur products by prep-HPLC was unsuccessful.*

Figure S4. Decomposition of **4b** in mild radical conditions by ^{1}H NMR

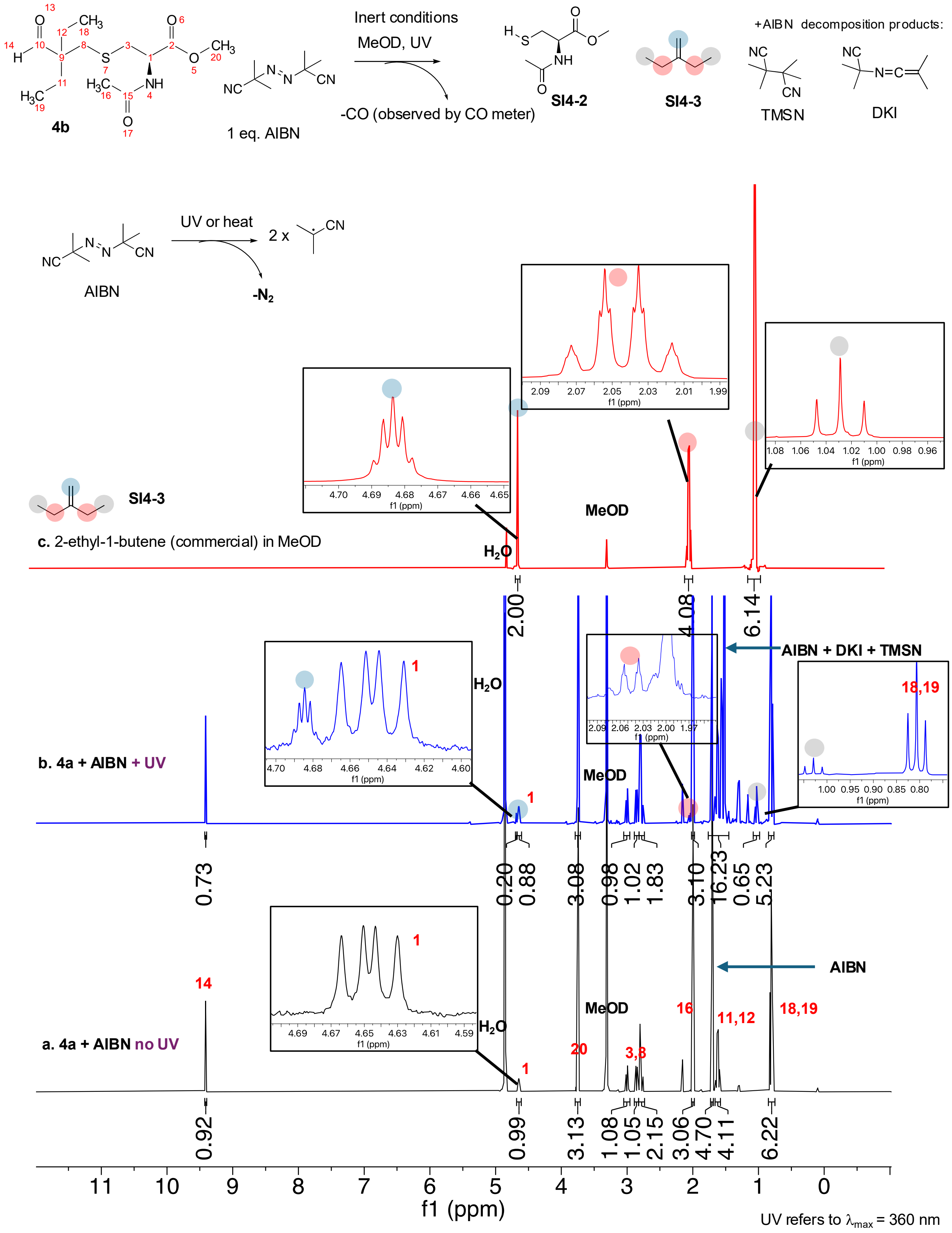

*Figure S- 4 Extension of the figure 3b from the main text. When **4b** CO prodrug (**a.**) is exposed to radical species at mild oxidising conditions the formation of **SI4-3** 2-ethyl-1-butene (**c., 1c-4** in the main text) is observed by ^{1}H NMR (**b.**). Azobis(isobutyronitrile) AIBN **SI4-1** is a known radical initiator (UV or temperature). Red numbers represent ^{1}H assignments of **4b**, while colourful spheres represent ^{1}H signals of **SI4-3** (2-ethyl-1-butene).*

*Formation of AIBN decomposition products **SI4-4** Tetramethylsuccinonitrile TMSN and **SI4-5** dimethyl-N-(2-cyano-2-propyl)ketenimine **DKI** is also observed in **b.** (assigned from the literature Nat. Commun. 5, 4662 (2014)). Signals for commercial 2-ethyl-1-butene (**SI4-3**) in **c.** ^{1}H NMR (400 MHz, MeOD) δ 4.68 (p, J = 1.2 Hz, 2H), 2.04 (qt, J = 7.5, 1.2 Hz, 4H), 1.03 (t, J = 7.5 Hz, 6H). Measured multiplicity for signals of interest (2-ethyl-1-butene) in spectrum **b.** (^{1}H NMR (400 MHz, MeOD) δ 4.68 (p, J = 1.2 Hz), 2.04 (qt, J = 7.5, 1.1 Hz), 1.03 (t, J = 7.5 Hz). The peak area could not be measured accurately due to the overlap of the signals.*

Figure S5. Decomposition of **4b** in mild radical conditions ^{1}H NMR DOSY

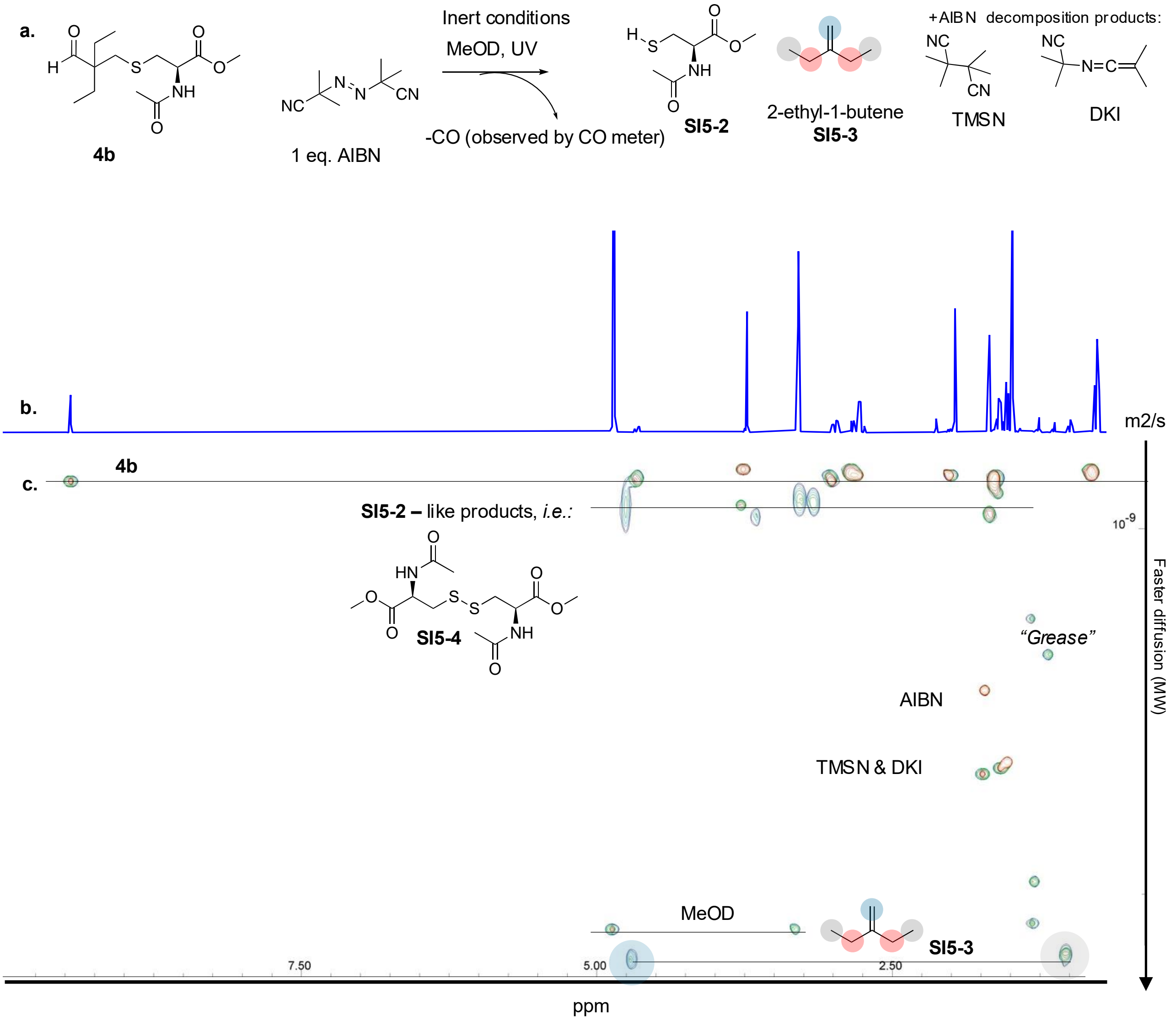

*Figure S- 5 ^{1}H NMR DOSY experiment of the sample described in **Figure S-4**. **a**. Proposed reaction scheme, **b.** ^{1}H NMR spectrum when **4b** is exposed to conditions as in **a.**, **c.** DOSY experiment, where signals can be diffentiated by representative molecular weight, i.e. small molecules diffuse faster. From bottom to top: **SI5-3**, 2-ethyl-1-butene is observed as a very fast-diffusing species, while **AIBN** and its decomposition products (**TMSN, DKI**) result in slower diffusion. These are followed by **N-acetylcysteine** (**SI5-2**)-like products (which show no saturated carbon peaks in the 0.8 ppm region, but may also include disulfide species like **SI5-4**), while unreacted **4b** is the slowest-diffusing molecule.*

Figure S6. Decomposition of **4b** in mild radical conditions by UPLC-MS

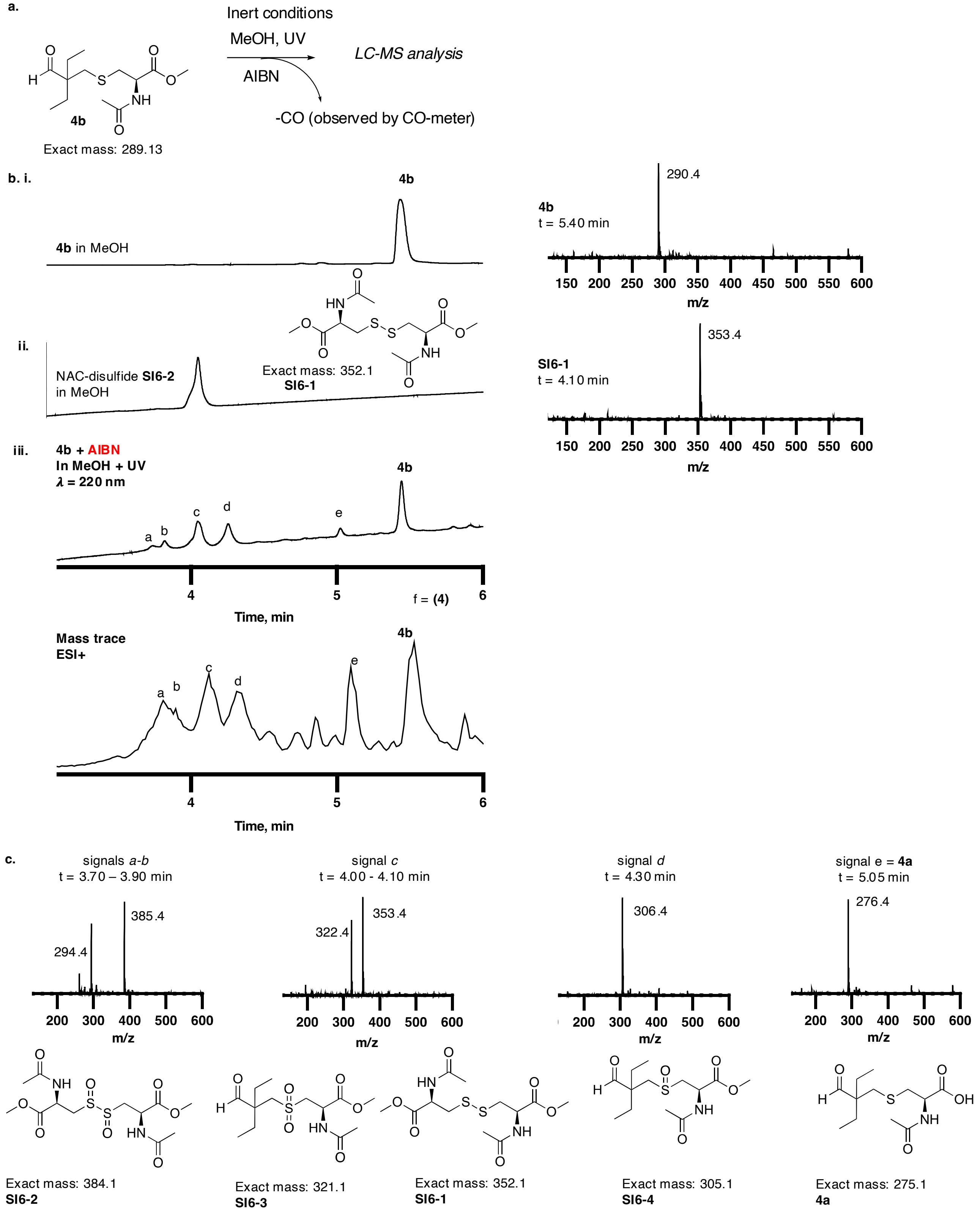

Figure S- 6 UPLC-MS analysis of the sample described in ***Figure S-4. a****. Proposed reaction scheme,* ***b.i.*** *UV traces of* ***4b*** *alone (pure) (left) with combined mass spectrum (right),* ***b.ii.*** *UV trace of N-acetylcysteine methyl ester disulfide* ***SI6-1*** *(left) with combined mass trace (right),* ***b.iii.*** *UV trace when* ***4b*** *was exposed to conditions described in* ***a.*** *(top), with combined ESI+ mass trace of sample (bottom).* ***c.*** *combined mass signals a-f from* ***b.iii.*** *with the proposed corresponding stuctures. Assigments has been made by combination of the methods, including analysis of ^{1}H NMR (Figures S-4 to S-6).*

Figure S7. 2-ethyl-1-butene detection in decomposition products of **4b** by GC-MS

a.

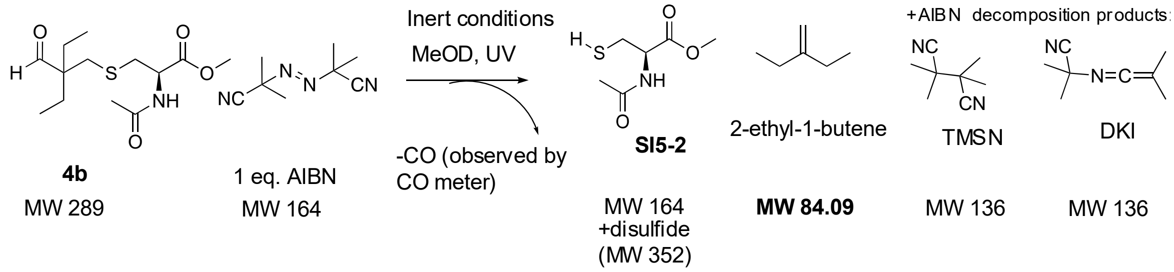

b. i. 2-ethyl-1-butene (standard)

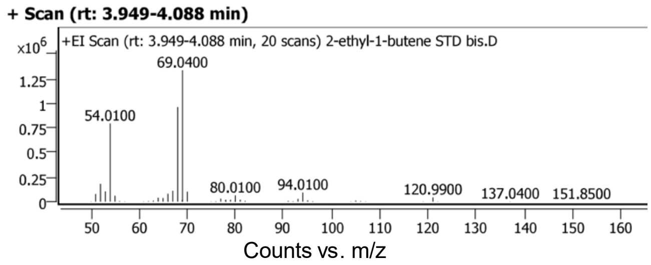

ii.

m/z<br>3.949-4.099 min	Abundance, %
54.01	59.5
55.02	4.80
69.04	100
70.03	7.8
84.04	0.11

c. i. Sample from **a.** MeO**D**

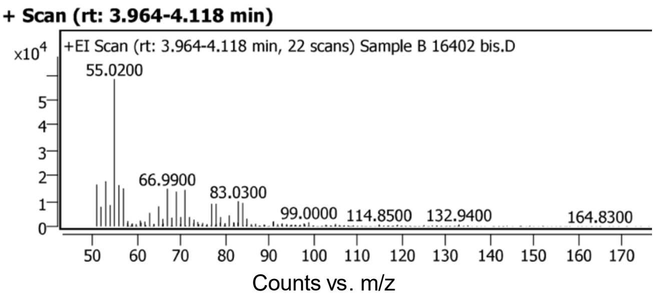

ii.

m/z<br>3.964-4.118 min	Abundance, %
54.01	14.5
55.02 (D)	100
69.04	23.7
70.05 (D)	24.7
83.99	15.9
84.99 (D)	5.3

*Figure S- 7 GC-MS experiment of the sample described in Figure S-4. **a.** Proposed reaction scheme. **b.** GC-MS of 2-ethyl-1-butene standard with ionization pattern (**i.**) and list of detected peaks (**ii.**). **c.i.** GC-MS trace from the sample described in **a.**, at t = 4 min, suggested the presence of 2-ethyl-1-butene as one of the decomposition products of **4b**. **ii.** The use of deuterated methanol as a solvent resulted in an intensification of +1 mass signals relative to the predicted mass.*

Figure S8. Stability of **4a-b** in PBS (pH 7.4) and human serum

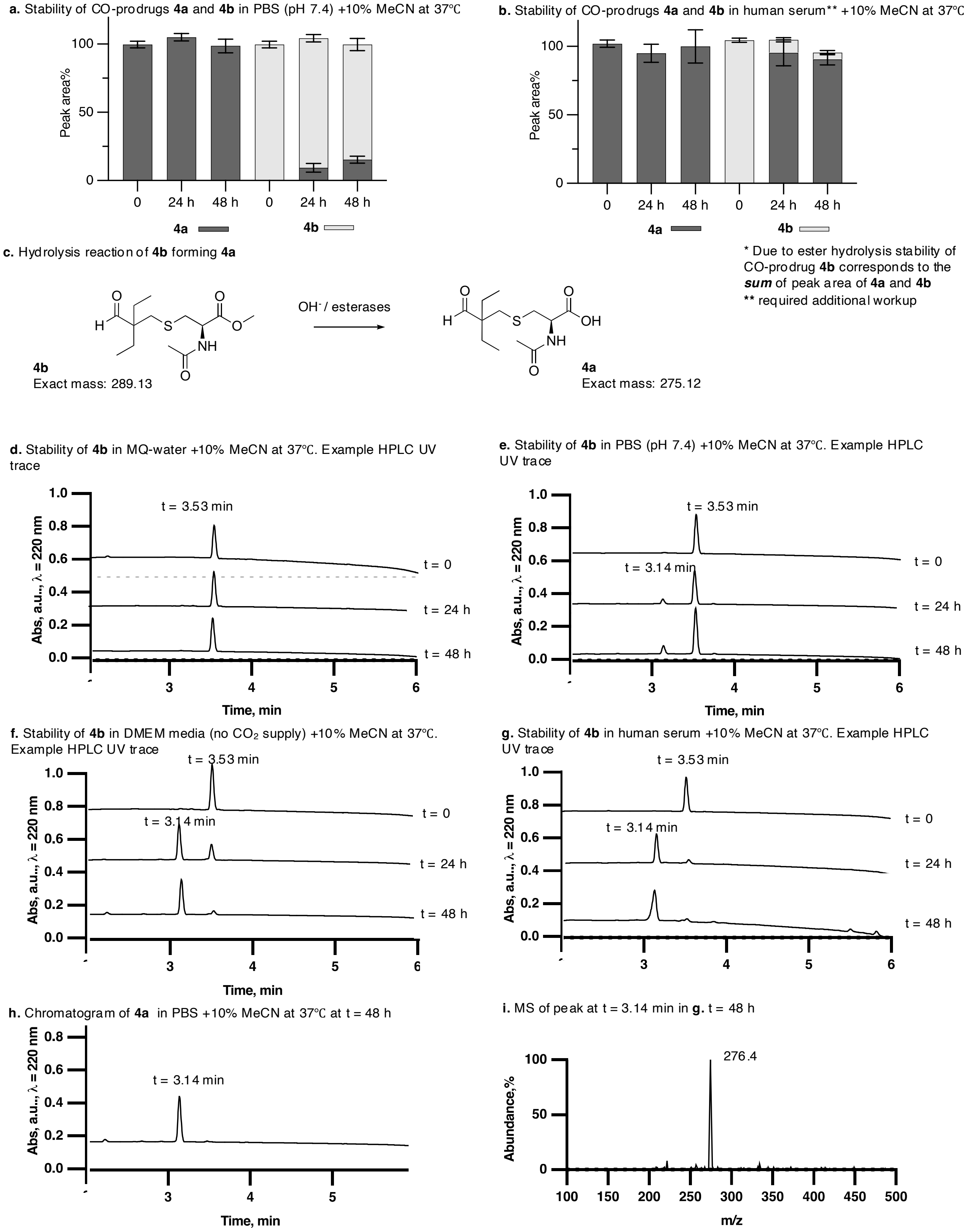

*Figure S- 8 Stability of CO-prodrugs **4a** and **4b** in PBS (pH 7.4) and human serum (**a.**, **b.**) analysed by UPLC-MS. CO-prodrug is stable in PBS and human serum over 48 hours. However, in basic conditions, or where esterase enzymes may be present, **4b** hydrolyses to **4a** (**c.**). The assay suggests that CO is not released, and the aldehyde is not oxidised. Example traces of **4b** and **4a** stability in different media is presented in figures **d.** - **i.***

Figure S9. Cell viability assay for compounds **4a** and **4b** for RAW264.7, HeLa and HEK293T cells

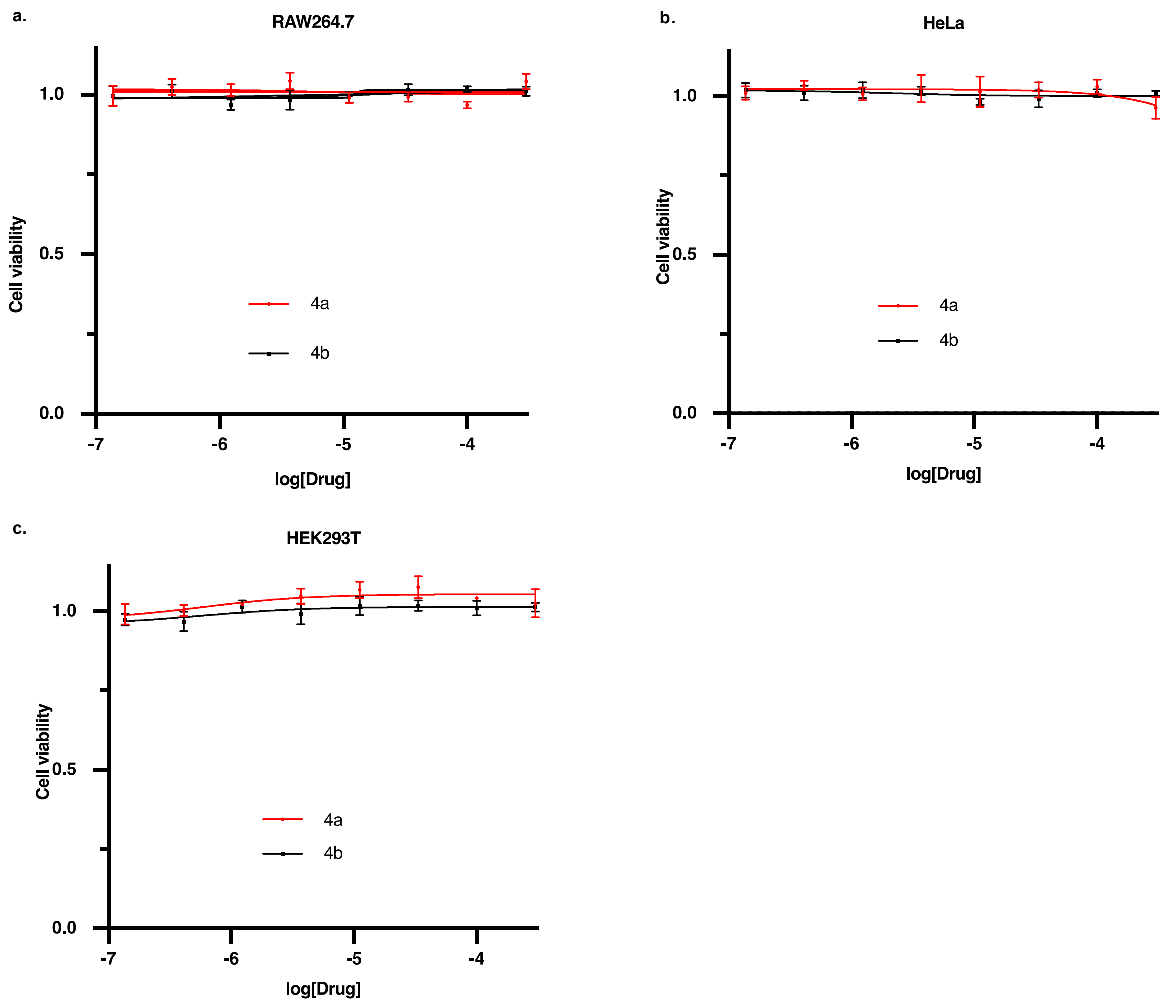

Figure S- 9 CO-prodrugs ***4a*** *and* ***4b*** *were completely non-toxic to RAW264.7, HeLa, HEK293T cell lines up to 300 μM tested, as represented by viability assays in* ***a., b.,*** *and* ***c.****, respectively. Error bars represent ± standard deviation (n = 3). The experiment was repeated three times for each drug and cell line using CellTiter blue™ assay. Drug concentration is in M (mol L^{-1}), log (300 μM) = -3.52, 1.0 represent 100% viability of the controls. Each experiment had a cytotoxic drug control (digitonin or doxorubicin).*

Figure S10. TNF-α and iNOS suppression by **4a** and **4b** in LPS-induced RAW264.7

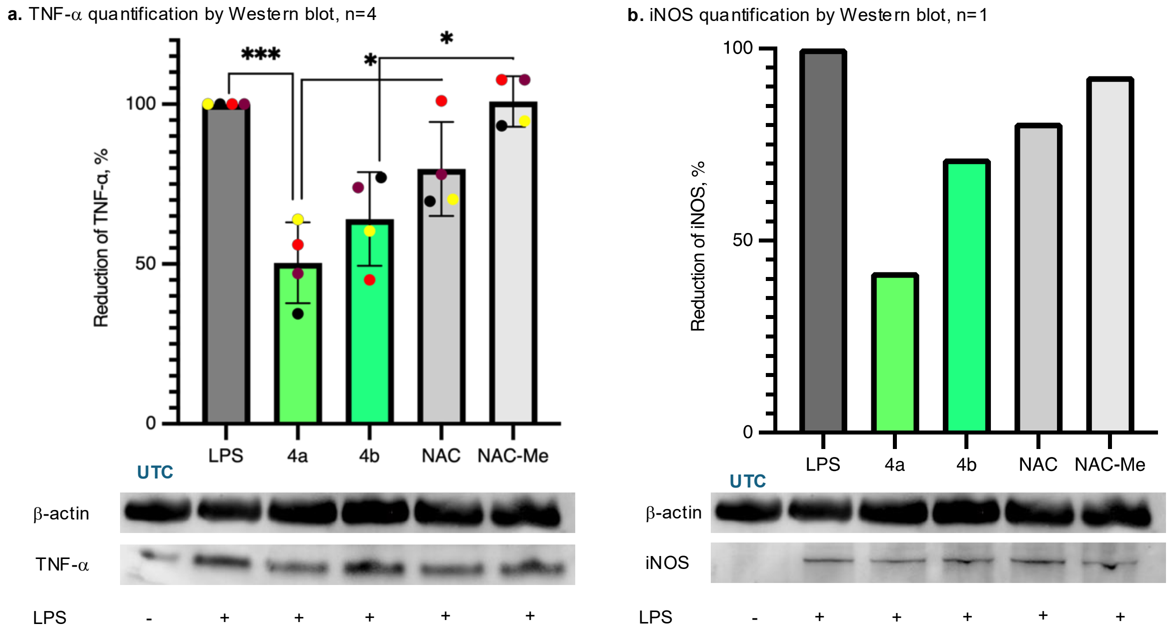

Figure S- 10 Compounds ***4a*** *and* ***4b*** *were shown to be effective in suppressing TNF-α (****a.****) and iNOS (****b.****) expression in LPS-induced (100 ng/mL, 16 hours) RAW264.7 macrophages, as assessed by Western blot analysis and subsequent image quantification. Final expression values were calculated by normalizing TNF-α and iNOS band intensities to β-actin using ImageJ software (gel quantification tool). Coloured dots in* ***a.*** *represent different gel replicates (black dots represent quantification of the shown gel). Statistically significant differences found using unpaired t-test and marked as ***($P \leq 0.0005$), *($P \leq 0.05$). The experimental setup should be further optimized by adjusting LPS concentration and induction time, and by employing more sensitive detection methods such as ELISA to measure TNF-α levels in the supernatant rather than the cell lysate. UTC – untreated control (no LPS induction), LPS – lipopolysaccharide treated (vehicle). TNF-α – Tumor necrosis factor alpha, iNOS – inducible nitric oxide synthase.*

Figure S11 CO release in SKBR3 triggered by endogenous ROS

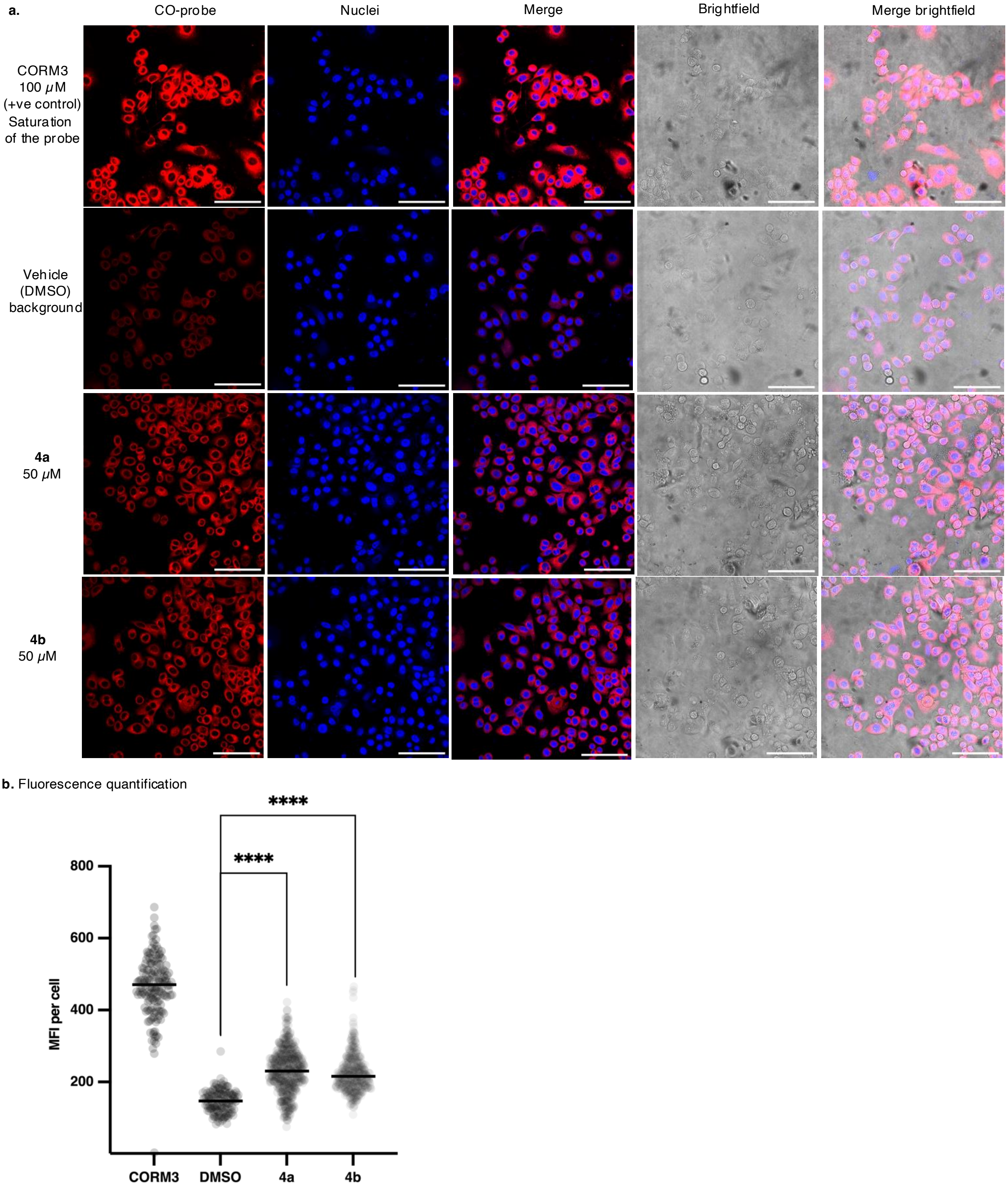

*Figure S- 11 Confocal microscopy images of SKBR3 (breast cancer) cells for cellular CO release in treated SKBR3 cells (50 μM of **4a** or **4b**) and untreated (control) using 1-Ac CO probe. After an initial 30 min of cell pre-treatment with CO probe (1-Ac, 5 μM) the cells were washed and prodrugs **4a** or **4b** or controls (vehicle – negative control, CORM3 100 μM – positive control, oversaturation of the probe to regulate the laser intensity) were added for 30 min. After the incubation, cells were washed, fixed and imaged by confocal microscope using DAPI for nulei (blue) and 561 nm channel (red, λ_{ex} = 561 nm, λ_{em} = 570 – 620 nm) for endogenous CO (CO probe turn-on response). White scale bar represents 100 μm. **b.** turn-on fluorescence quantification (mean fluorescence intensity) suggested significant CO detected from both **4a** and **4b**. Each dot represents fluorescence of a single cell; data sets combine two separate biological replicates. Statistically significant differences found after comparing whole populations (vehicle vs. treated) using unpaired t-test and marked as *****

Figure S12 CO release in HeLa triggered by endogenous ROS

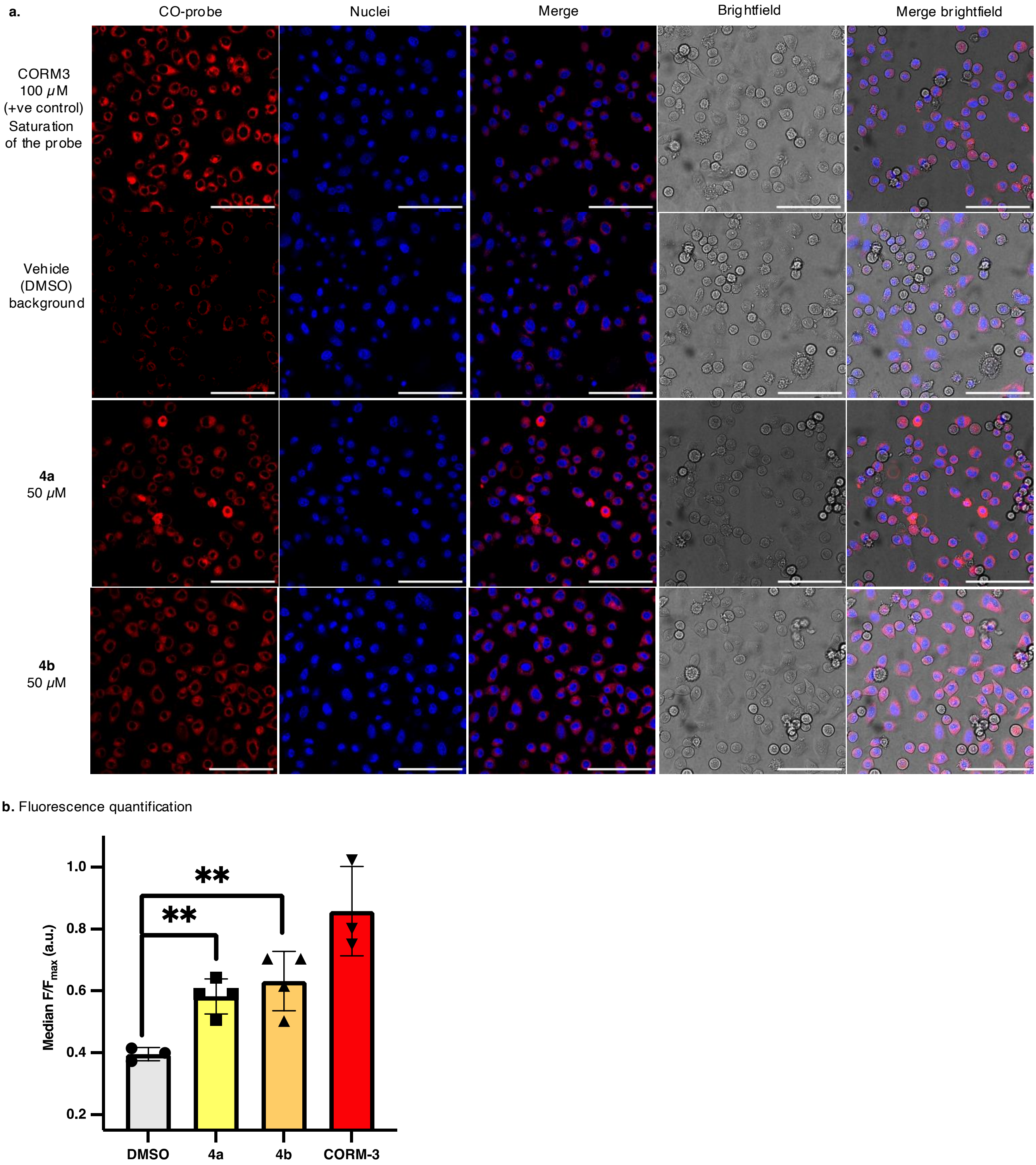

*Figure S- 12 Confocal microscopy images of HeLa (cervical cancer) cells for cellular CO release in treated HeLa cells (50 μM of **4a** or **4b**) and untreated (control) using 1-Ac CO probe. After an initial 30 min of cell pre-treatment with CO probe (1-Ac, 5 μM) the cells were washed and prodrugs **4a** or **4b** or controls (vehicle – negative control, CORM3 100 μM – positive control) were added for 30 min. After the incubation, cells were washed, fixed and imaged by confocal microscope using DAPI for nulei (blue) and 561 nm channel (red, λ_{ex} = 561 nm, λ_{em} = 570 – 620 nm) for endogenous CO (CO probe turn-on response). White scale bar represents 100 μm. **b.** turn-on fluorescence quantification (per image) suggested significant CO detected from both **4a** and **4b**. Error bars represent standard deviation within separate biological replicates. Statistically significant differences found after unpaired student t-test and marked as ** (P<0.005).*

Figure S13 CO release in MCF7 triggered by endogenous ROS

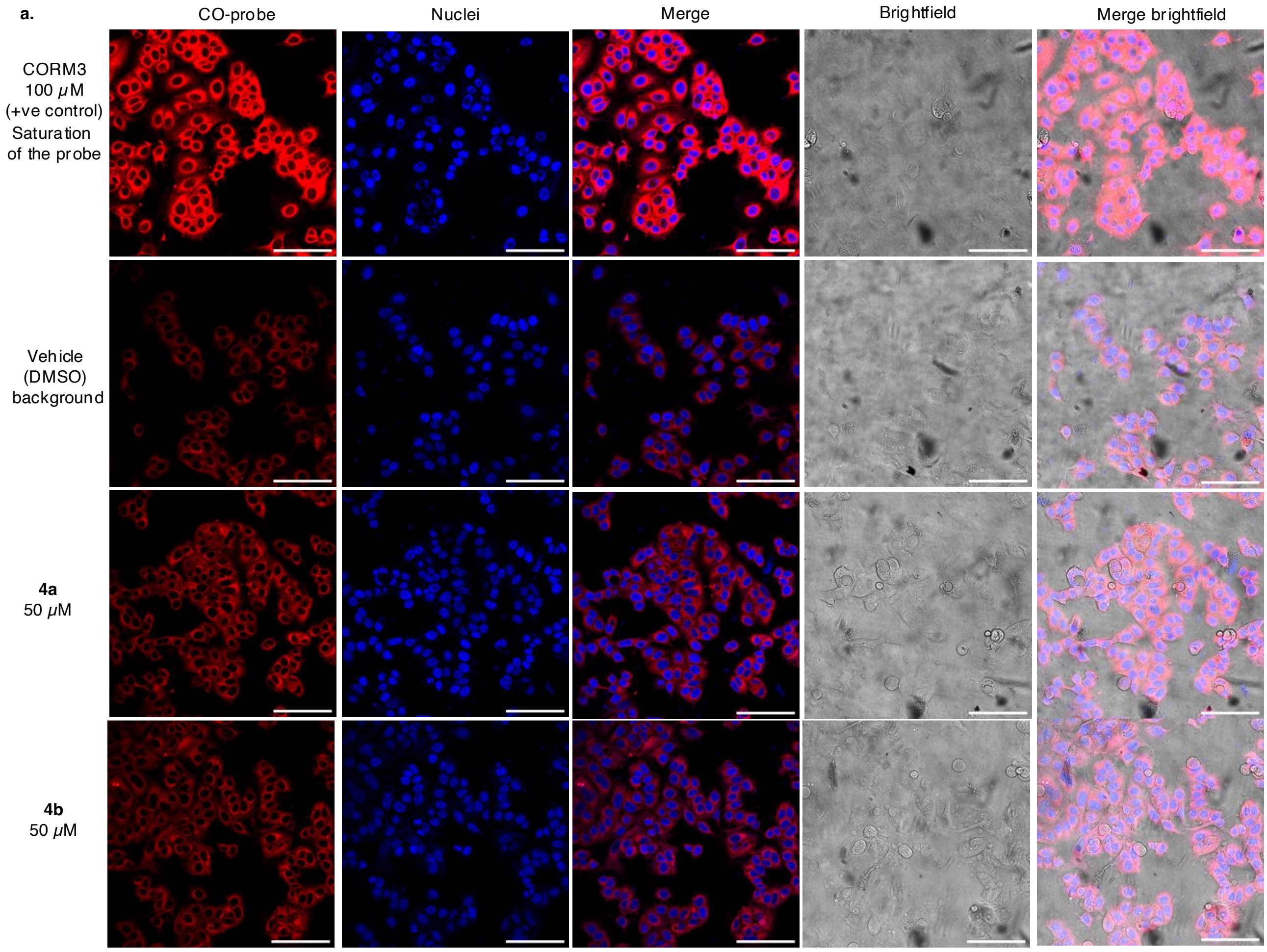

a. Fluorescence quantification

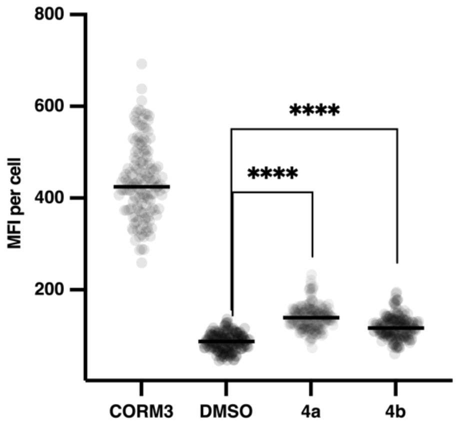

*Figure S- 13 Confocal microscopy images of MCF7 (breast cancer) cells for cellular CO release in treated MCF7 cells (50 μM of **4a** or **4b**) and untreated (control) using 1-Ac CO probe. After an initial 30 min of cell pre-treatment with CO probe (1-Ac, 5 μM) the cells were washed and prodrugs **4a** or **4b** or controls (vehicle – negative control, CORM3 100 μM – positive control) were added for 30 min. After the incubation, cells were washed, fixed and imaged by confocal microscope using DAPI for nulei (blue) and 561 nm channel (red, λ_{ex} = 561 nm, λ_{em} = 570 – 620 nm) for endogenous CO (CO probe turn-on response). White bar represents 100 μm. **b.** turn-on fluorescence quantification (mean fluorescence intensity) suggested significant CO detected from both **4a** and **4b**. Each dot represents the fluorescence of a single cell, data sets combine two separate biological replicates. Statistically significant differences found after comparing whole populations (vehicle vs. treated) using unpaired t-test and marked as **** (P<0.00005).*

Figure S14 CO release in **live** RAW264.7 imaged by **COP-1** under ROS induction by lipopolysaccharides (LPS)

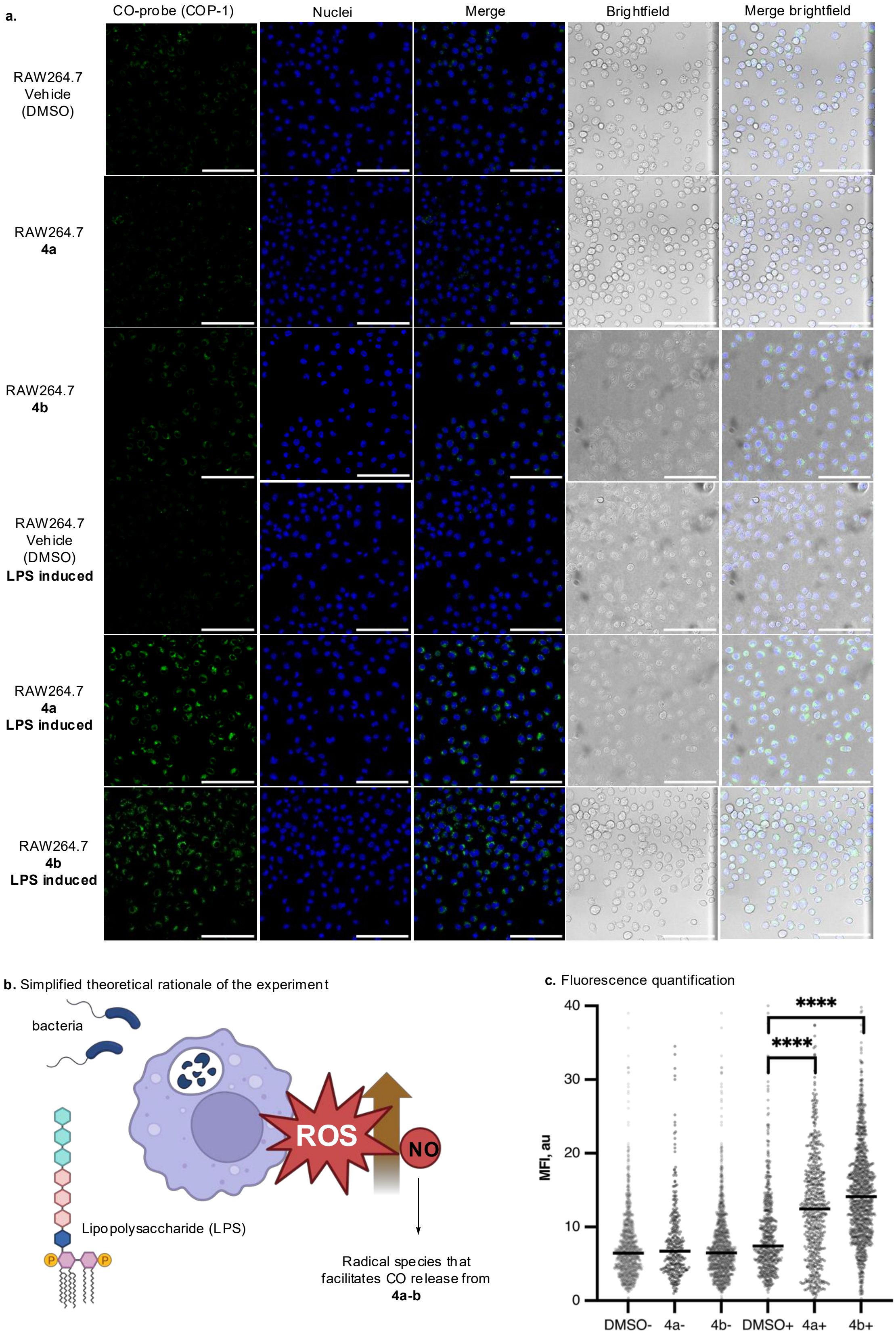

Figure S- 14 ***a.*** *Confocal microscopy images of RAW264.7 (murine macrophages) cells for cellular CO release in treated RAW264.7 cells (50 µM of* ***4a*** *or* ***4b****) and untreated (control) using COP-1 CO probe where the production of reactive*

*oxygen species was induced using LPS (Lipopolysaccharides) or not. After an initial 30 min of cell pre-treatment with LPS (0.5 μg/mL), cells were pre-treated with CO probe (COP-1, 1 μM) and then the cells were washed and prodrugs **4a** or **4b** or controls (vehicle – negative control) were added for 30 min. After the incubation, cells were imaged live (5% CO_2, 37ºC) by confocal microscope using Hoechst 33342 for nulei (blue) and 488 nm channel (green, λ_{ex} = 488 nm, λ_{em} = 500 – 650 nm) for endogenous CO (CO probe turn-on response). White scale bar represents 100 μm. **b.** Rationale of the experiment – LPS mimic bacterial infection, hence, the concentration of ROS increases to mimic the fighting of the resultant infection **c.** turn-on fluorescence quantification (mean fluorescence intensity) suggested significant CO detected from both **4a** and **4b** only when cells were briefly induced with LPS (+). Prolonged LPS exposure itself may lead to endogenous CO increase, hence, for this experiment, the induction with LPS was very brief (30 min in low concentration 0.5 μg/mL) so it did not elicit significant endogenous CO production. Each dot represents fluorescence of a single cell, data sets combine two separate experiments. Statistically significant differences found after comparing whole populations (vehicle vs. treated) using unpaired t-test and marked as **** (P<0.00005). (+) indicates LPS induction, (-) indicates LPS-untreated control.*

N.B. In this experiment, different CO-probe was used (COP-1, green vs. 1-Ac, red). This is because this was one of the initial experiments using first reported probe (COP-1. For later experiments (Figures S11-S13) COP-1 was swapped for 1-Ac since 1-Ac was less toxic to cells and had higher fluorescence yield. This allowed for a higher-dose treatment of the probe and allowed for cell fixation.

Figure S15 Weight variation in tumor-bearing mice

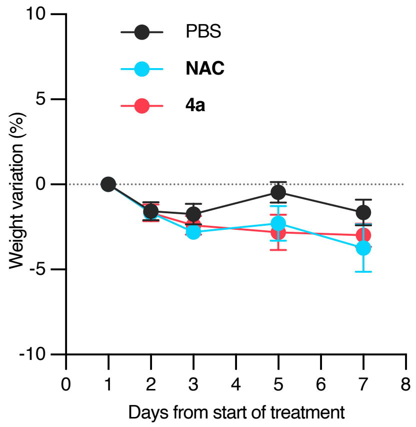

Figure S- 15 Weight variation in tumor-bearing mice indicating that all treatments were well-tolerated. Data are represented as mean±s.e.m. (n=7).

Figure S16 QM study: Labelling of ethyl substituent compounds for calculations

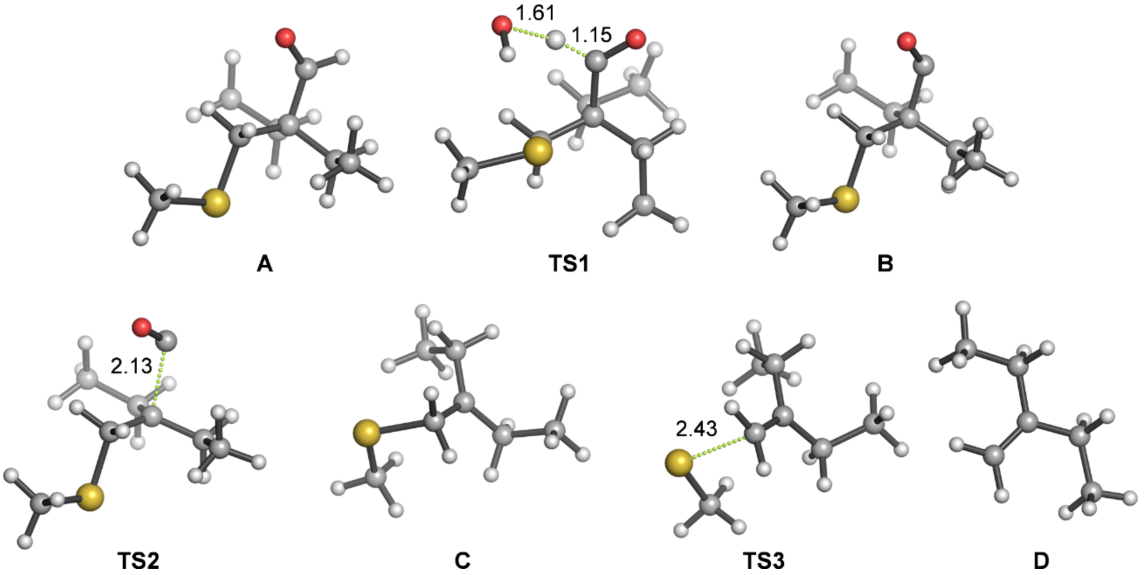

Figure S- 16 Lowest-energy structures of the ethyl-substituted reactants, intermediates, products and transition structures for the H-abstraction (TS1), decarbonylation (TS2) and retro-thiol-ene (TS3) reactions calculated with PCM(H_2O)/M06-2X/6-311+G(2d,p). Breaking bonds are represented with green dotted lines. Distances are given in angstrom.

Figure S17 QM study: Labelling of methoxy substituent compounds for calculations

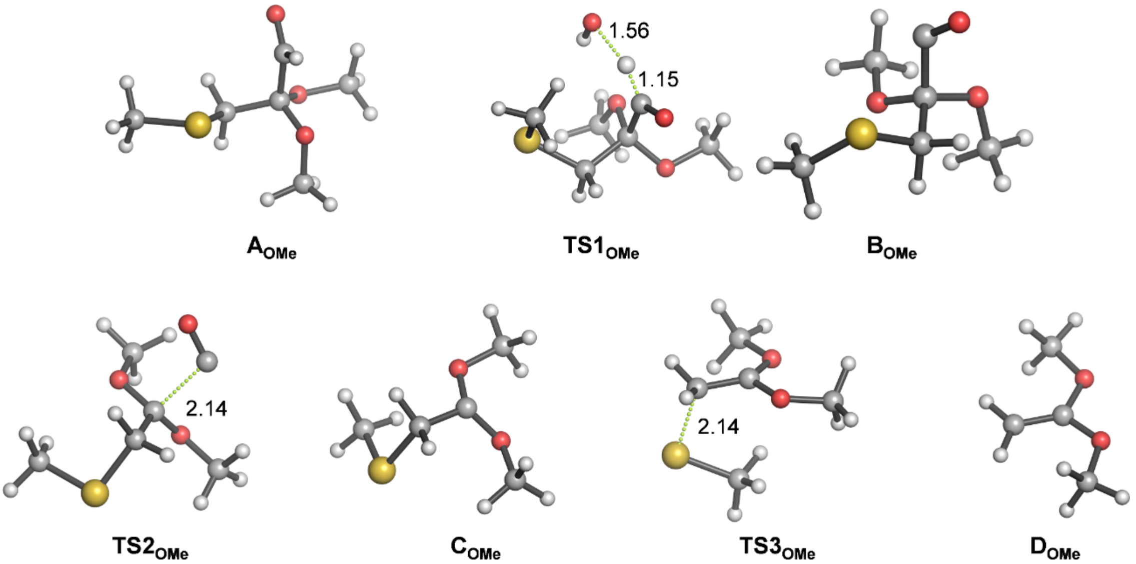

Figure S- 17 Lowest-energy structures of the methoxy-substituted reactants, intermediates, products and transition structures for the H-abstraction (TS1), decarbonylation (TS2) and retro-thiol-ene (TS3) reactions calculated with PCM(H_2O)/M06-2X/6-311+G(2d,p). Breaking bonds are represented with green dotted lines. Distances are given in angstrom.

Figure S18 QM study: Labelling of sulfinyl compounds for calculations

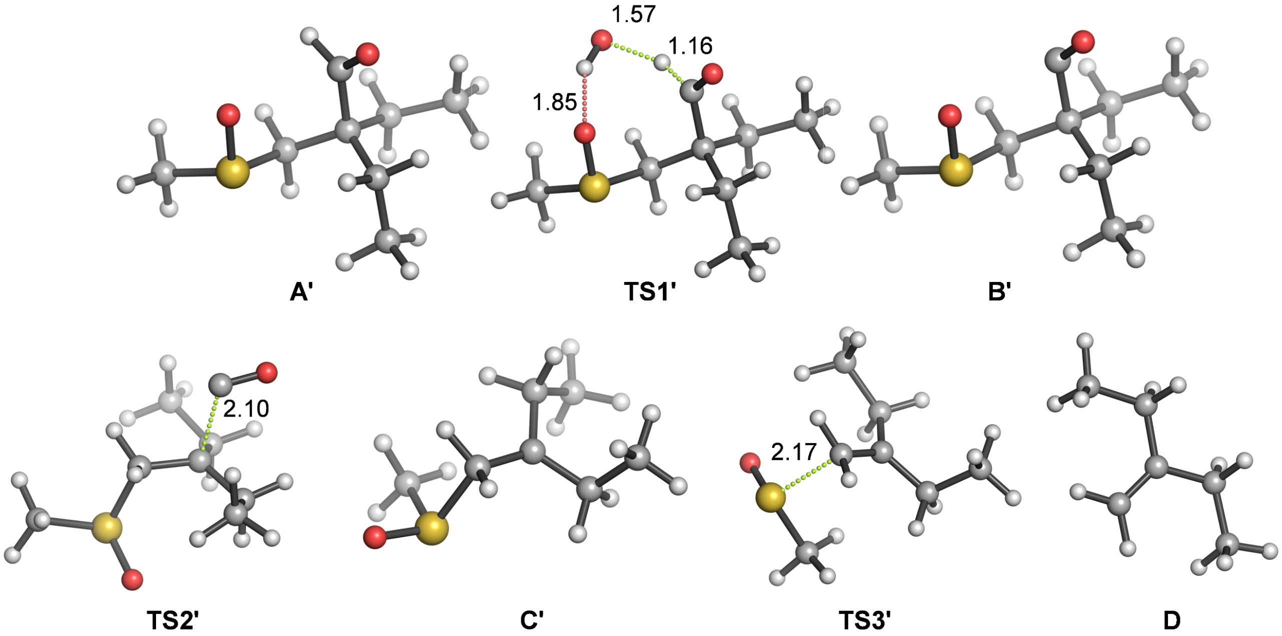

Figure S- 18 Lowest-energy structures of the sulfinyl-substituted reactants, intermediates, products and transition structures for the H-abstraction (TS1), decarbonylation (TS2) and retro-sulfoxide-ene (TS3) reactions calculated with PCM(H_2O)/M06-2X/6-311+G(2d,p). Breaking bonds are represented with green dotted lines. Distances are given in angstrom.

Figure S19 QM study: Labelling of sulfonyl compounds for calculations

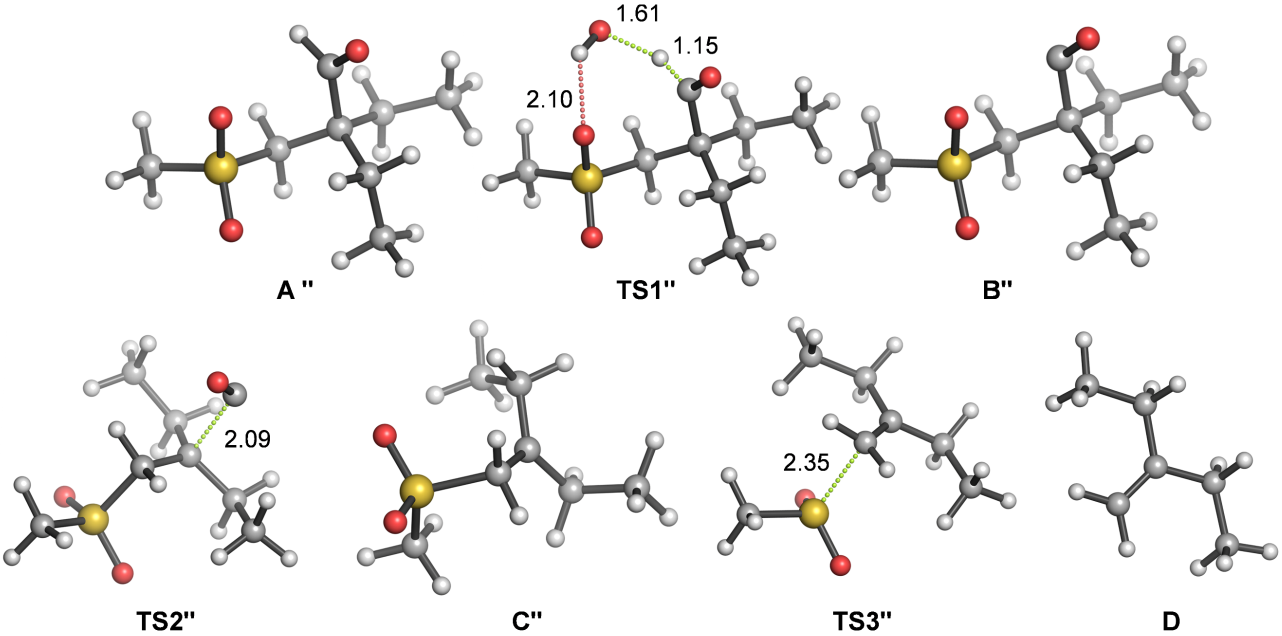

Figure S- 19 Lowest-energy structures of the sulfonyl-substituted reactants, intermediates, products and transition structures for the H-abstraction (TS1), decarbonylation (TS2) and retro-sulfone-ene (TS3) reactions calculated with PCM(H_2O)/M06-2X/6-311+G(2d,p). Breaking bonds are represented with green dotted lines. Distances are given in angstrom.

Figure S20 QM study: Comparison of geometries and spin densities of intermediate radicals and transition states

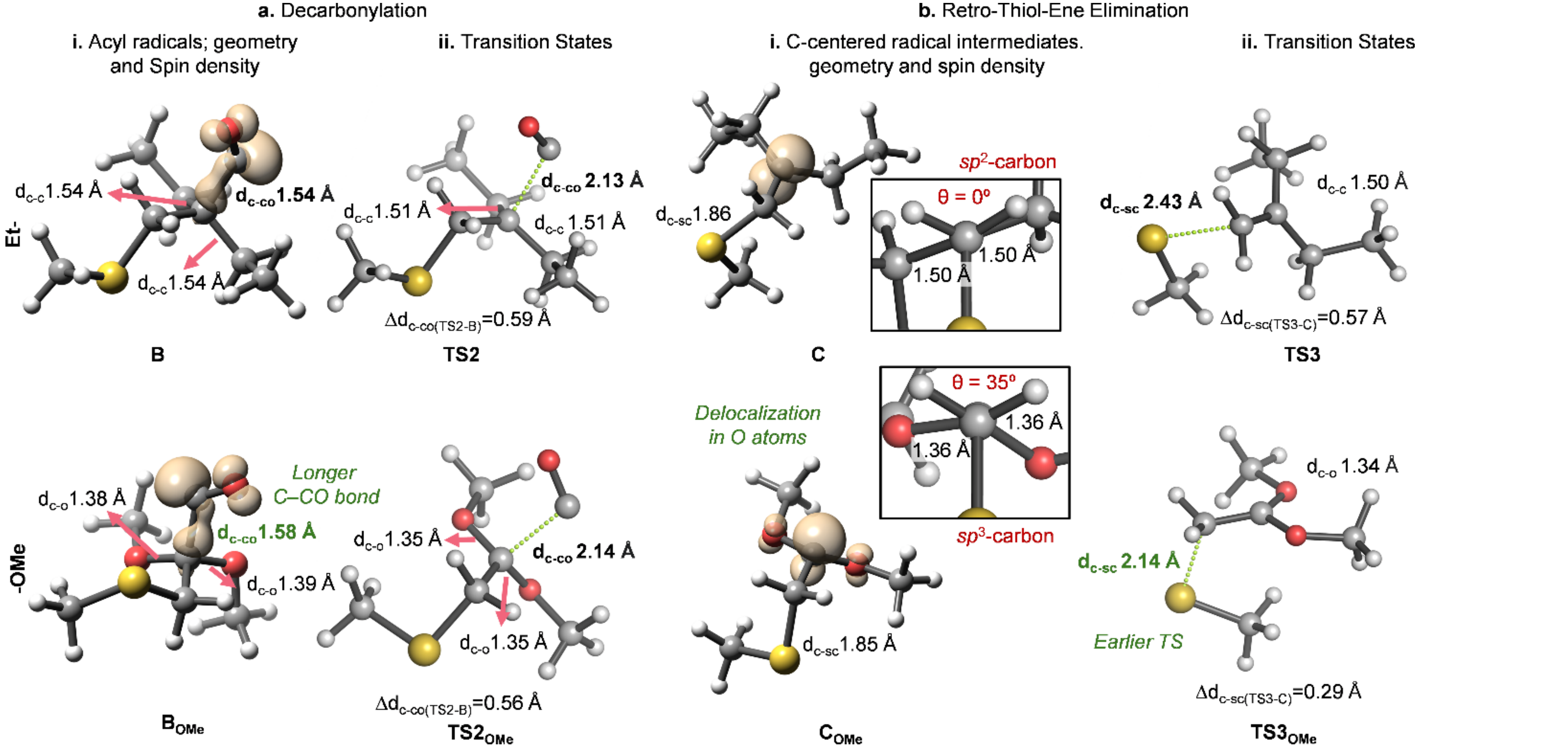

Figure S- 20 ***a.i.*** *The unpaired electron in* ***B*** *and* $\boldsymbol{B_{OMe}}$ *is delocalized along the CO moiety and the labile C-C bond. The C-CO bond distance in* $\boldsymbol{B_{OMe}}$ *is slightly longer than in* ***B****, reflecting its pre-distorted nature (i.e. predisposition to CO release), in agreement to the lower activation barrier for this step.* ***a.ii.*** *Due to the shortening of the C-CO bond,* $\boldsymbol{B_{OMe}}$ *has a smaller difference in distance with respect to* $\boldsymbol{TS2_{OMe}}$*, which corresponds to a lower activation energy in line with Hammond's postulate.* ***b.i.*** *Pyramidalization angle (θ, i.e. the* sp^3*-character of the tertiary radical) is remarkably larger in* C_{OMe}*, which would translate into instability. However, the spin density is delocalized on the adjacent oxygen atoms, compensating such destabilization. Conversely, C displays a complete* sp^2*-character, translating into a larger stability due to increased hyperconjugation, although compensated by a lack of delocalization, therefore resulting in a increase in the relative energy.* ***b.ii.*** *The breaking C–S bond is quite shorter in* $\boldsymbol{TS3_{OMe}}$ *(i.e. early TS) compared to* ***TS3****, which corresponds to a lower activation energy, in agreement to Hammond's postulate.*

Figure S21 QM study: Minimum-energy pathway for the methoxy-substituted compounds

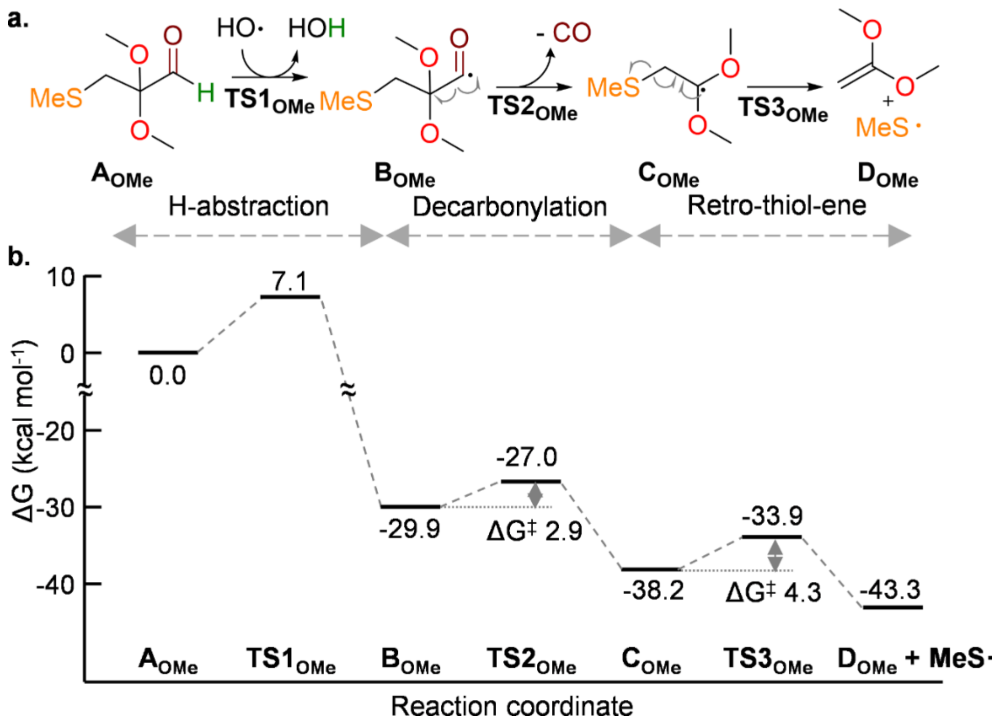

*Figure S- 21 **a.** Mechanism and **b.** minimum energy pathway calculated with PCM(H_2O)/M06-2X/6-311+G(2d,p) for the H-abstraction, decarbonylation and retro-thiol-ene reactions of methoxy-substituted aldehyde.*

Figure S22 QM study: Minimum-energy pathway for sulfoxide derivatives

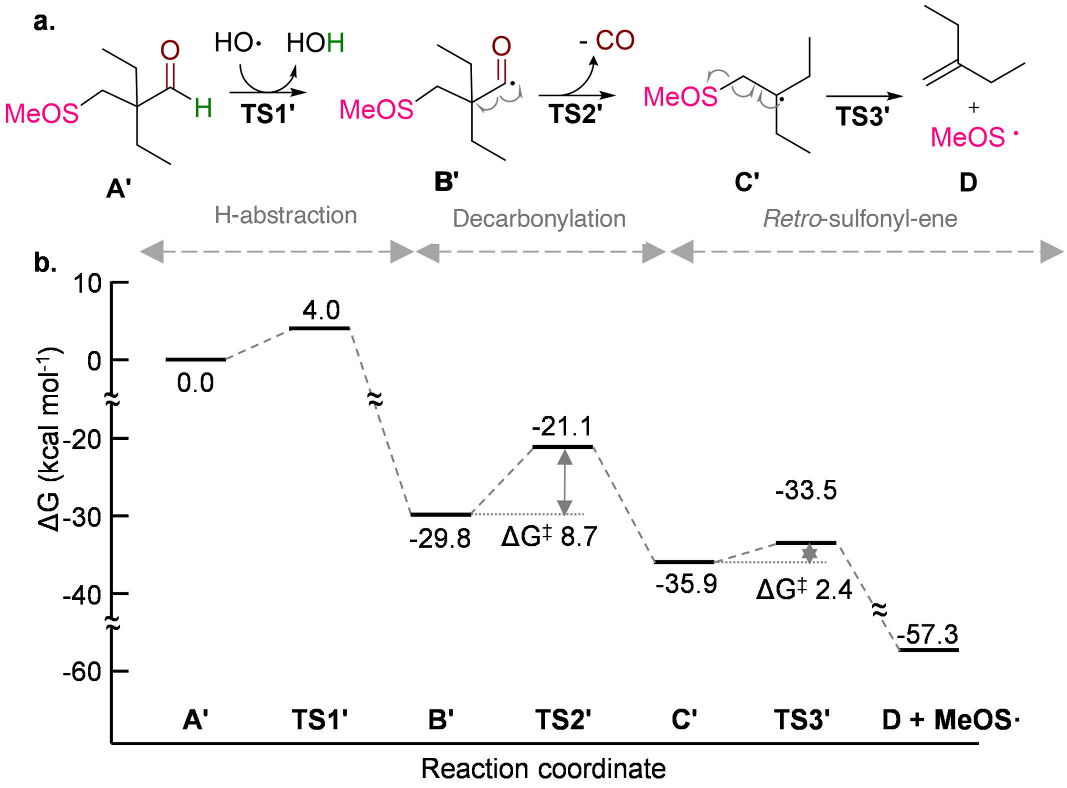

*Figure S- 22 **a.** Radical reaction mechanism and **b.** minimum energy pathway calculated with PCM(H_2O)/M06-2X/6-311+G(2d,p) for the decarbonylation and retro-sulfoxide-ene reactions from tertiary sulfinyl aldehyde A'.*

Figure S23 QM study: Minimum-energy pathway for sulfone derivatives

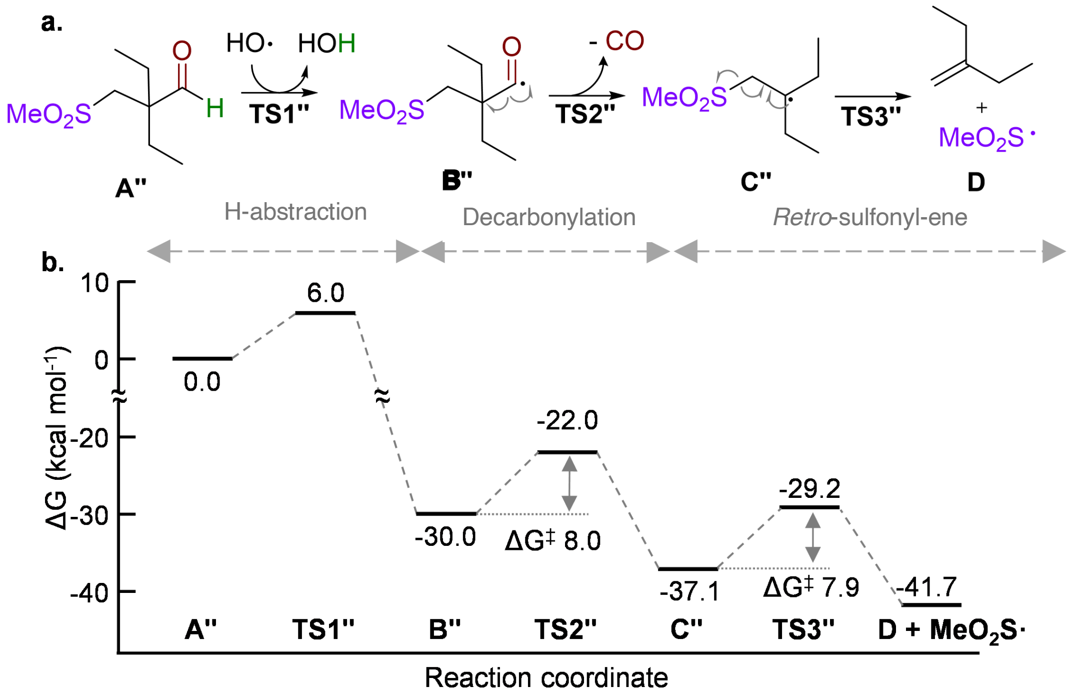

Figure S- 23 ***a.*** *Radical reaction mechanism and* ***b.*** *minimum energy pathway calculated with PCM(H_2O)/M06-2X/6-311+G(2d,p) for the decarbonylation and retro-sulfone-ene reactions from tertiary sulfonyl aldehyde A".*

Figure S24 QM study: Intrinsic Reaction Coordinates for thioether derivatives

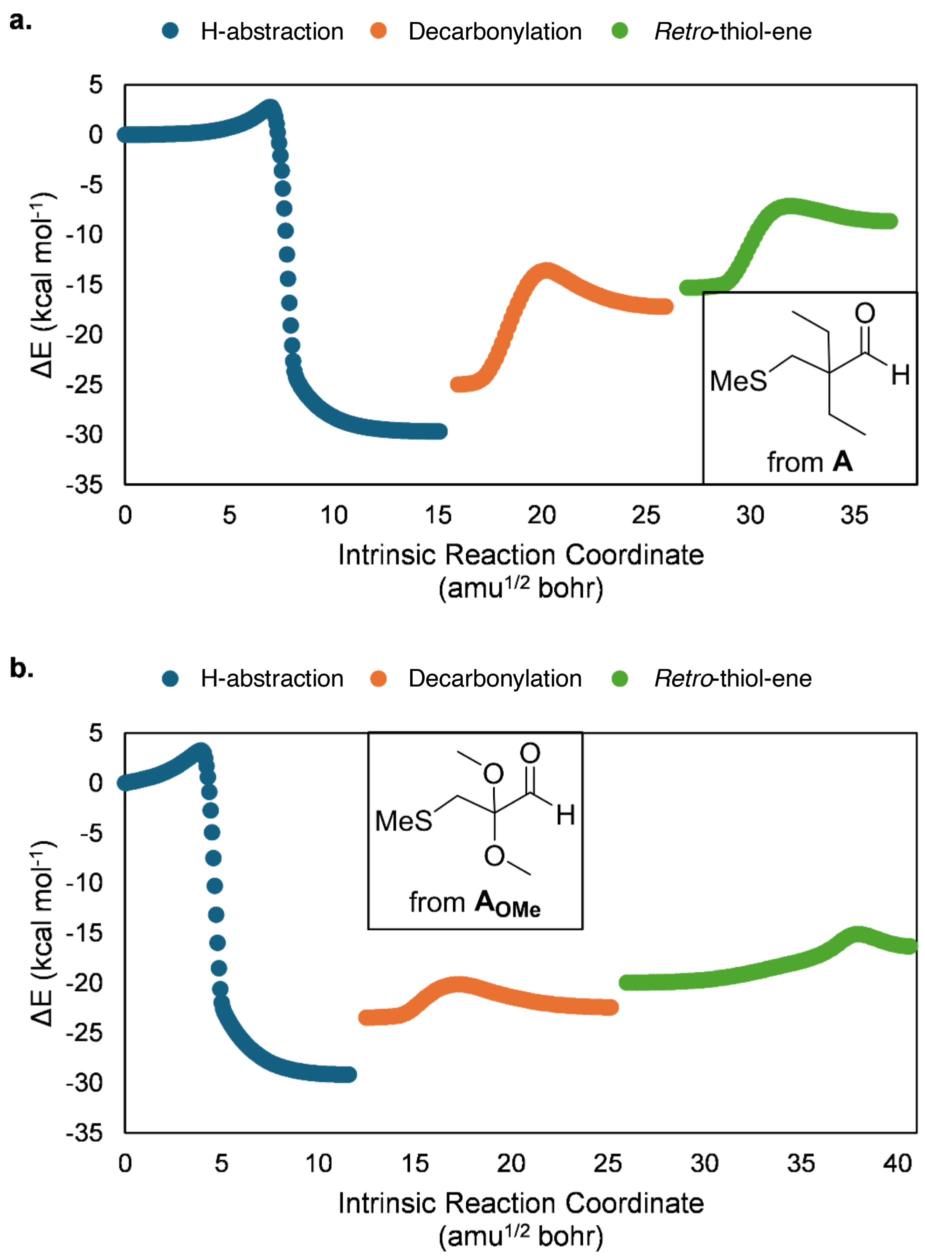

*Figure S- 24 Intrinsic reaction coordinates (IRC) calculated with PCM(H_2O)/M06-2X/6-311+G(2d,p) from the lowest-energy transition structures for the H-abstraction (blue), decarbonylation (orange) and retro-thiol-ene (green) reactions of ethyl (**a**) methoxy (**b**) substituent aldehydes. The electronic energy of the first point for each IRC was arbitrarily set to 0 kcal mol^{-1}.*

Figure S25 QM study: Intrinsic Reaction Coordinates for sulfoxide and sulfone derivatives

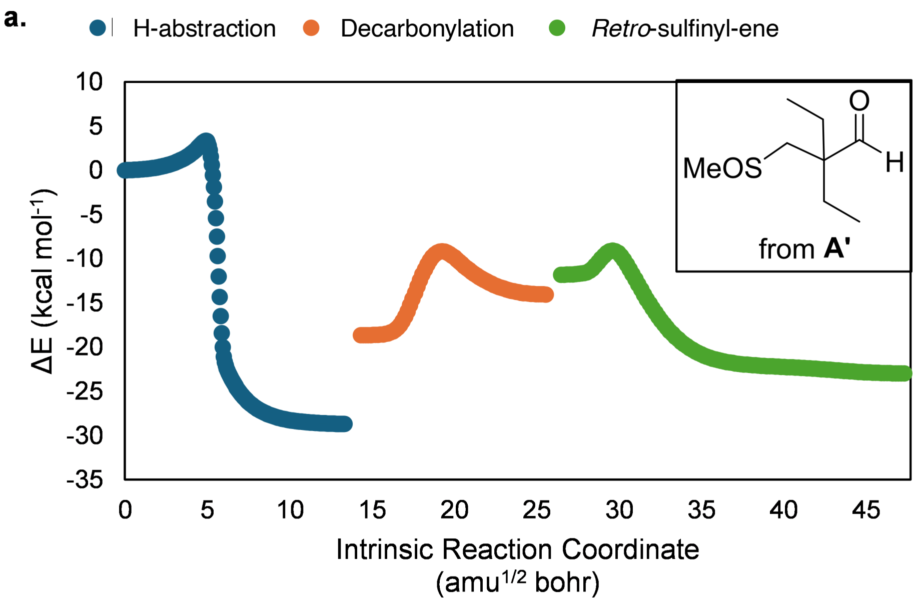

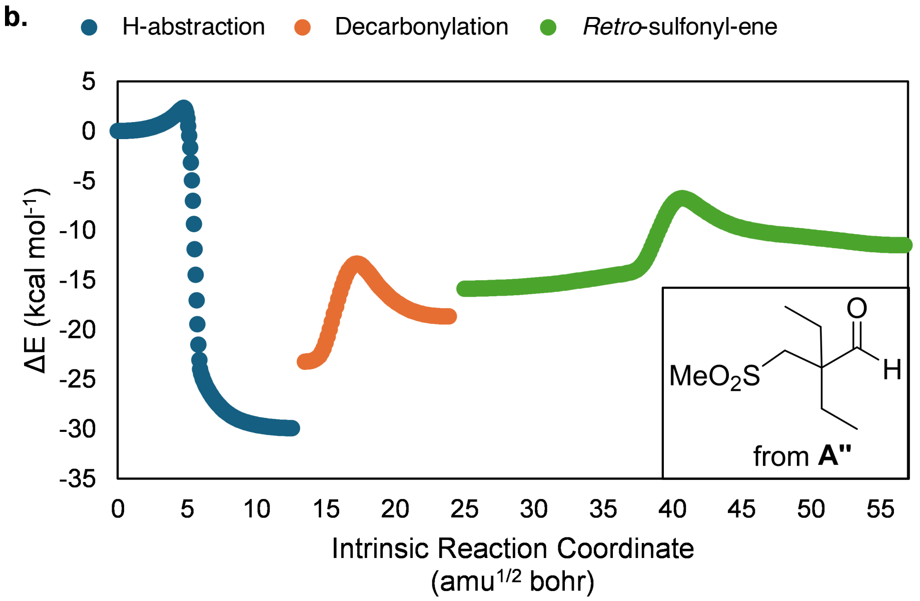

*Figure S- 25 Intrinsic reaction coordinates (IRC) calculated with PCM(H_2O)/M06-2X/6-311+G(2d,p) from the lowest-energy transition structures for the H-abstraction (blue), decarbonylation (orange) and retro-ene (green) reactions of sulfinyl- (**a**) and sulfonyl- (**b**) substituted tertiary aldehydes. The electronic energy of the first point for each IRC was arbitrarily set to 0 kcal mol^{-1}.*

Figure S26 QM study: instability of α-S-alkyl radicals

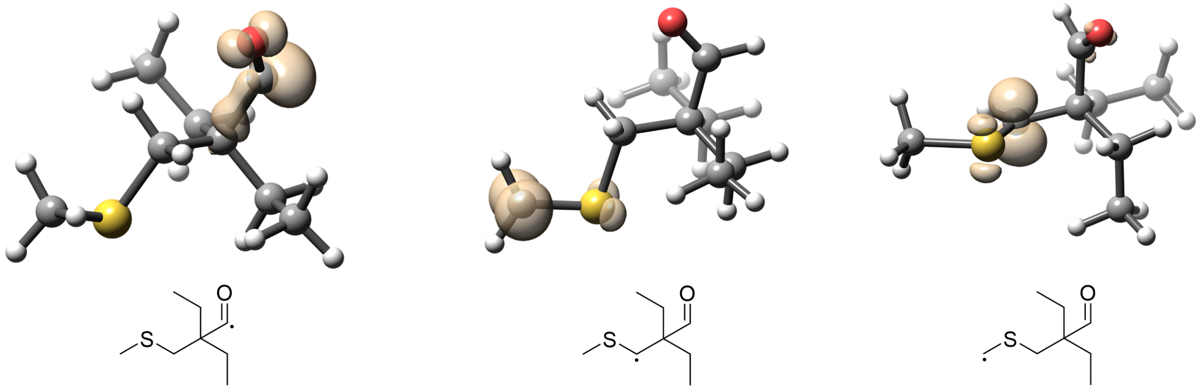

	Aldehyde radical	Secondary α-thio radical	Primary α-thio radical
ΔΔG (kcal mol^{-1})	0.0	+2.1	+5.1

Figure S- 26 a. Lowest-energy structures, relative free energies and spin densities of aldehyde (most stable) vs. secondary α-thio and primary (least stable) α-thio radicals calculated with PCM(H_2O)/M06-2X/6-311+G(2d,p).

Figure S27 Alternative building blocks synthesised

a. Synthesis of Se containing building block

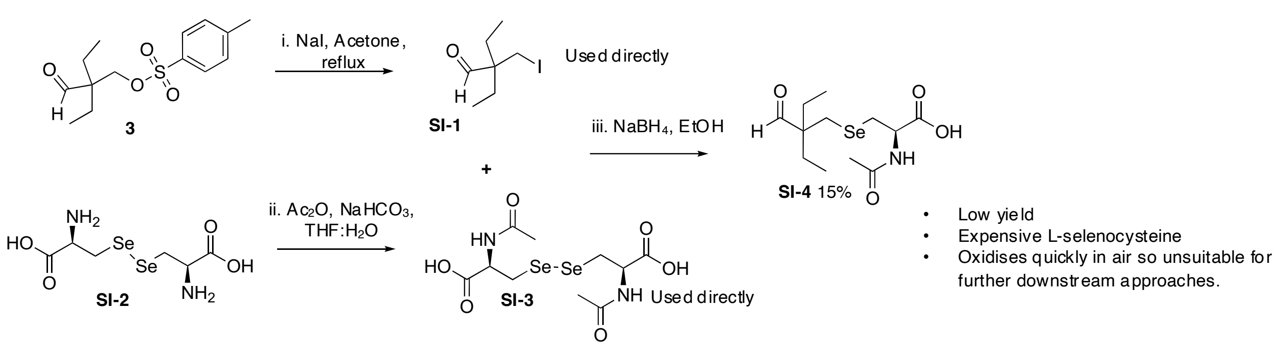

b. i. Synthesis of –Me containing building block

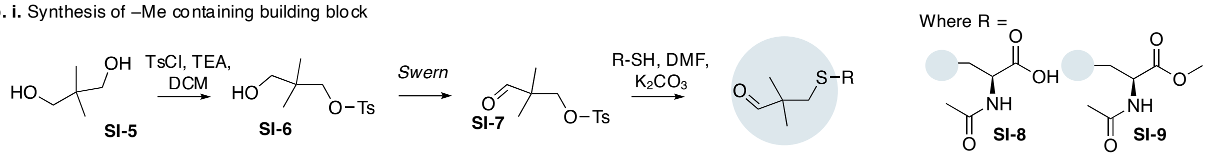

ii. Cell viability of **SI-9** vs **4b** in RAW264.7

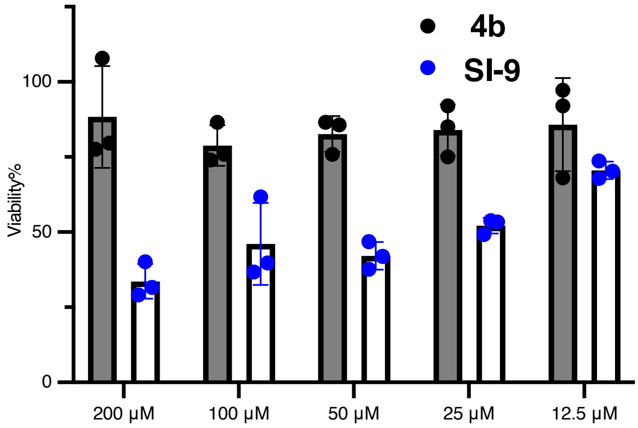

iii. Brightfield at viability assay endpoint (t = 48h) [**SI-9**] or [**4b**]= 200 µM
Scale bar = 200 µM

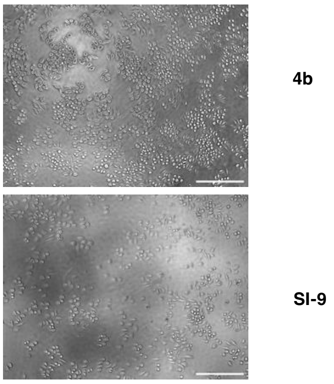

c. i. Synthesis of –OMe containing building block

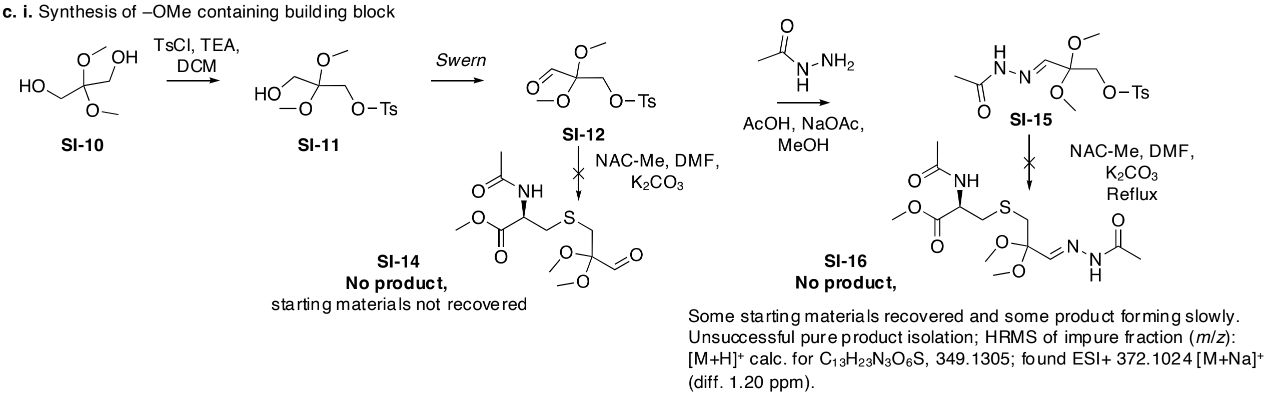

Figure S- 27 Alternative CO-releasing prodrugs considered, attempted development and discontinued. ***a.*** *Synthesis of selenium bearing compound* ***SI-4*** *as an alternative to* ***4a.*** *Hypothesis: selenium will be a better leaving group; result: low yield, unstable to selenium oxidation, no therapeutic improvement over* ***4a*** *(data not shown).* ***b.ii.*** *synthesis of methyl-bearing* ***SI-8*** *and* ***SI-9.*** *Hypothesis: reduced steric bulk may enhance aqueous solubility, hence low aqueous solubility thiols could be used as carriers. Result:* ***ii.*** *compounds were more toxic to RAW264.7 (experiment performed once) while* ***iii.*** *brightfield images suggested that it rather inhibited cell proliferation (lower confluency at end point rather than apoptotic cells observed) suggesting that aldehyde group was reactive and cross-linked proteins having formaldehyde-like effect. We hypothesise that methyl groups may not provide enough steric bulk and allow for aldehydes to be attacked by biological nucleophiles.* ***c.*** *Synthesis of methoxy bearing compound* ***SI-14*** *as an alternative to* ***4a.*** *Hypothesis: lowest activation barrier for CO release by quantum mechanical calculations. Result:* ***SI-12*** *is unstable in -20 °C over long periods (CO released, data not shown), while any attempts of* ***SI-14*** *synthesis resulted in full product decomposition. We hence tried to protect aldehyde, i.e. as hydrazide* ***SI-15*** *for potential tumour specific (lower pH) aldehyde release, however, the molecule was too sterically bulky for any kind of* S_N2 *reaction to take place. Methoxy derivatives may still be of interest, but it is out of scope for this publication.*

Figure S28 Aldehyde stability towards intramolecular biological nucleophiles – intramolecular reaction

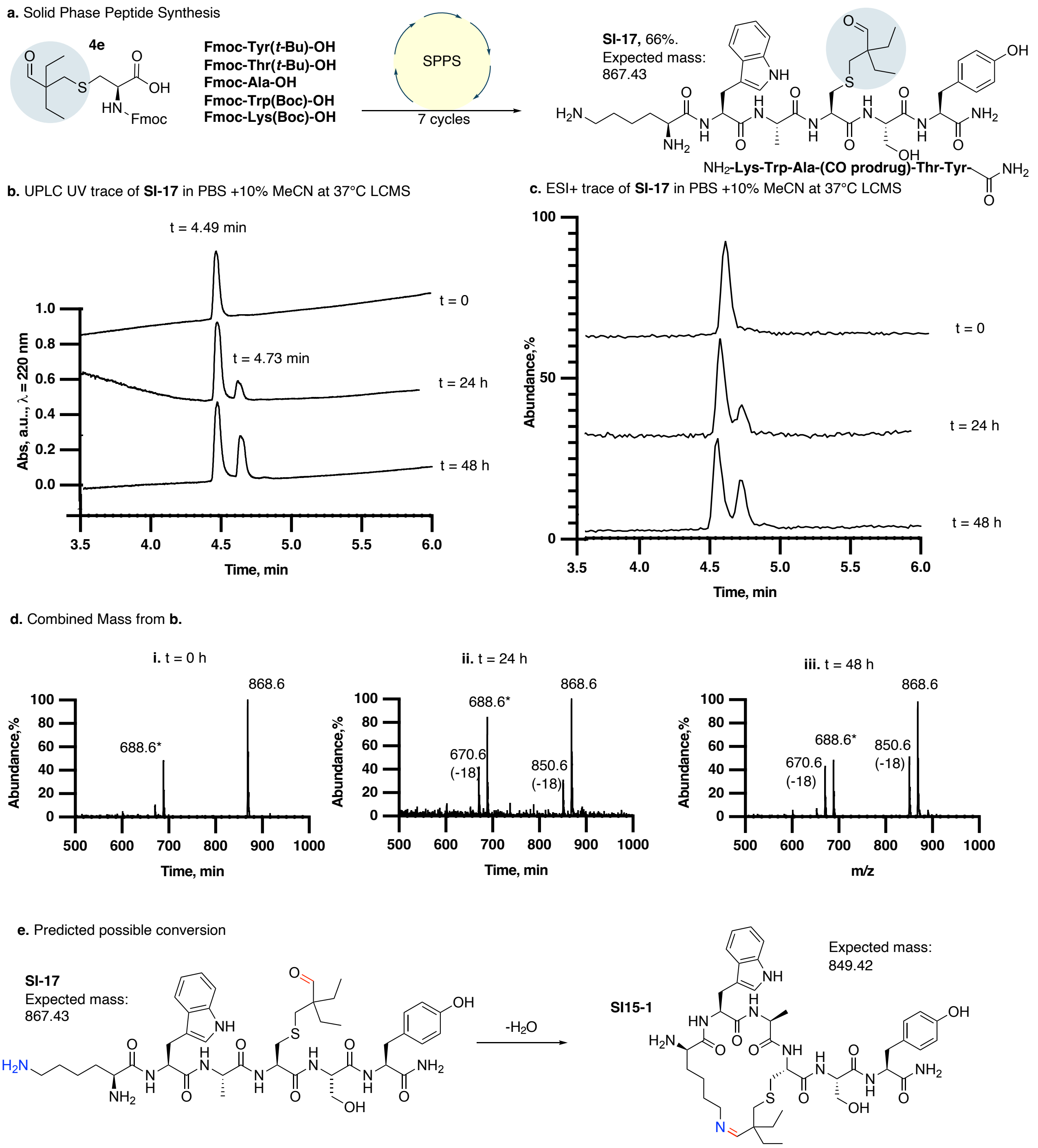

Figure S- 28 ***a.*** *Using* ***4e****, peptide* ***SI-17*** *containing reactive groups (such as lysine, non-protected N-terminus, tyrosine, threonine) was synthesised in high yield by standard SPPS procedure and this did not require aldehyde protection.* ***b.*** *Forced (****intramolecular****) conditions to detect aldehyde-amine condensation. After porlonged incubation in slighly basic pH (phospate-buffered saline, PBS, pH 7.4) some formation of similar polarity product of mass -18 Da was observed (****b.-d.****). We hypothesise that in this context, the intramolecular proximity of free the lysine allowed the condensation to occur. The formation of imine was not confirmed by other methods. Any conditions attempted (pH 3.0, 7.4, 10.0) of condensation of* ***4a/4b*** *and lysine or guanosine (****intermolecular*** *reaction) did not result in any detectable adducts (data not shown); this effect was not observed in stability of protein-CO-prodrug conjugates. *We propose that mass 688.6 appears due to the partial fragmentation of* ***SI-17****, since the mas diference of 180 Da does not correspond to loss of 1-2 amino acids and/or partial decomposition of CO-releasing prodrug .*

Figure S29 Peptide Synthesis and Bioconjugation to **Trastuzumab**

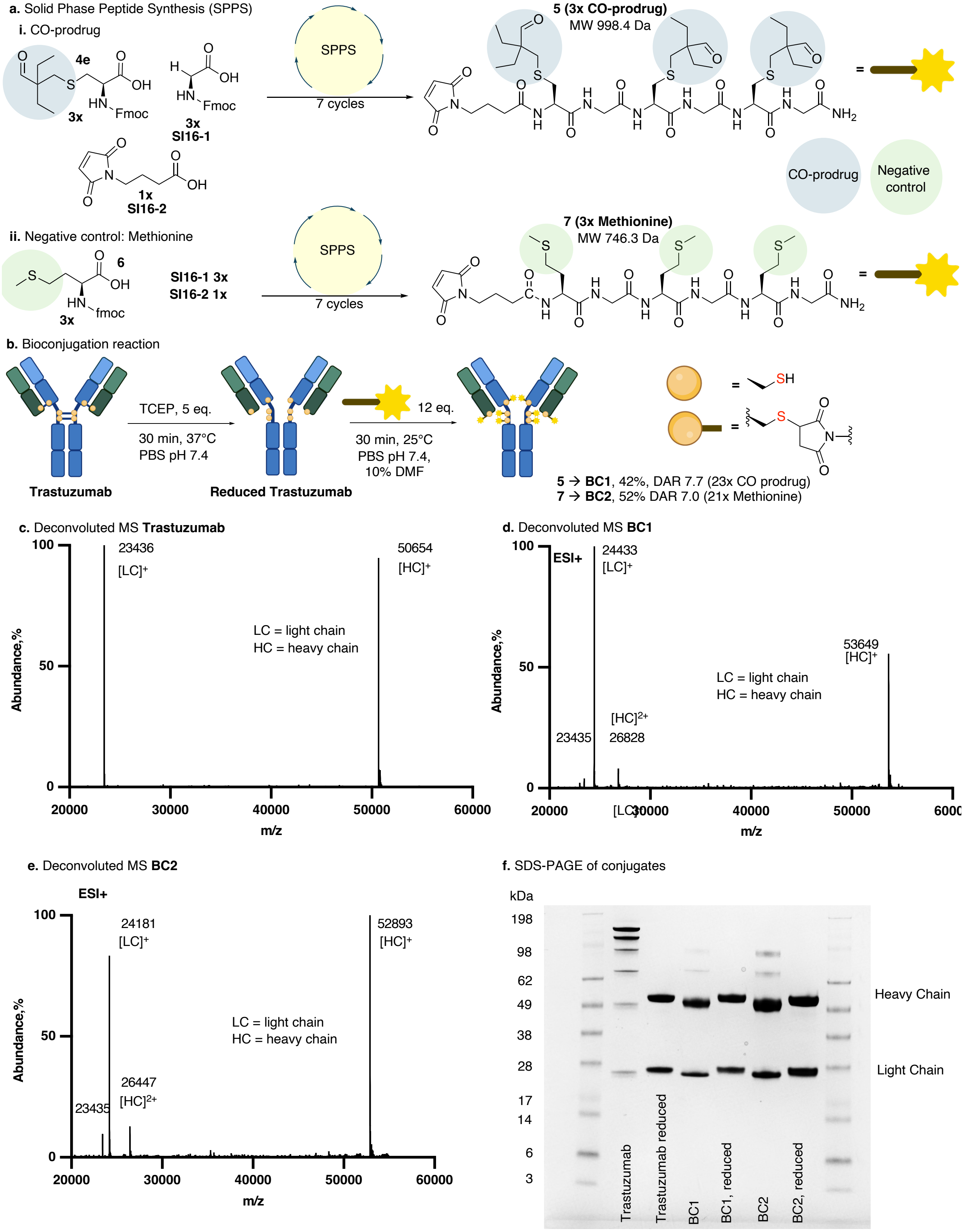

Figure S- 29 Figure S- 28 ***a.*** *SPPS synthesis scheme of* ***5*** *and* ***7*** *that carry CO-prodrugs, or negative control (Methionine, Met), respectively. 4-maleidobutyric acid was used as a final coupling step serving as a bioconjugation handle.* ***b.*** *Schematic of the reaction of interchain-cysteine tagged antibodies with reagents* ***5*** *and* ***7***, ***c.-e.*** *deconvoluted mass spectra (MS) ESI+ for trastuzumab itself and bioconjugates,* ***BC1*** *(calcd. For LC 24,434 Da, HC 53,648 Da, found LC 24,433 Da, HC 53,649 Da) and* ***BC2*** *(calcd. For LC 24,182 Da, HC 52,892 Da, found LC 24,181 Da, HC 52,893 Da).*

f. *SDS-PAGE of protein conjugates. Both SDS-PAGE and MS suggest that drug to antibody ratio (DAR) achieved was close to 8 due to low higher mass intensity bands in non-reduced gel conditions and lack of unconjugated proteins in MS. DAR 7.7 was calculated by mass intensity (peak area) in deconvoluted spectra. DMF – dimethylformamide, TCEP – tris(2-carboxylethyl)phosphine, PBS – phosphate-buffered saline, HC – heavy chain. LC – light chain.*

Figure S30 BC1 binding to the receptor

a. Biolayer Interferometry of BC1

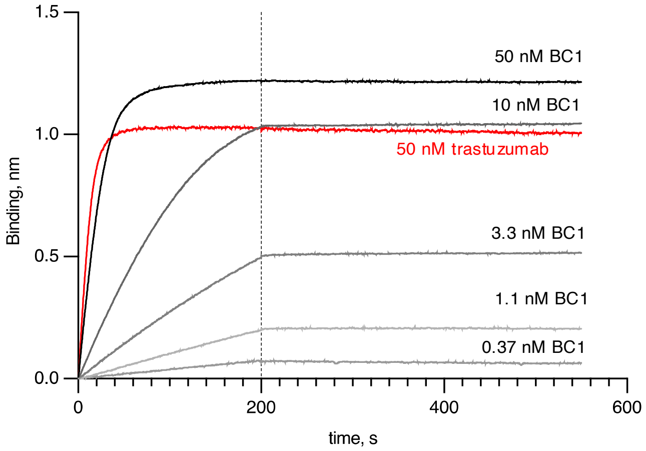

b. Specifications by SARTORIUS (BLI product manufacturer) versus obtained values*

	K_D (nM)	k_a (Ms^{-1})	k_d (s^{-1})
Trastuzumab*	0.487	2.60E+5	1.27E-4
Enhertu*	0.218	2.83E+5	6.18E-4
Trastuzumab**	0.233 ± 0.140	(5.10 ± 1.00) E+5	(1.19 ± 0.673) E-4
BC1**	0.440 ± 0.147	(5.84 ± 0.11) E+5	not determined**

c. Flow cytometry data in SKBR3 (HER2+) **d.** Flow cytometry data in MCF7 (HER2 low)

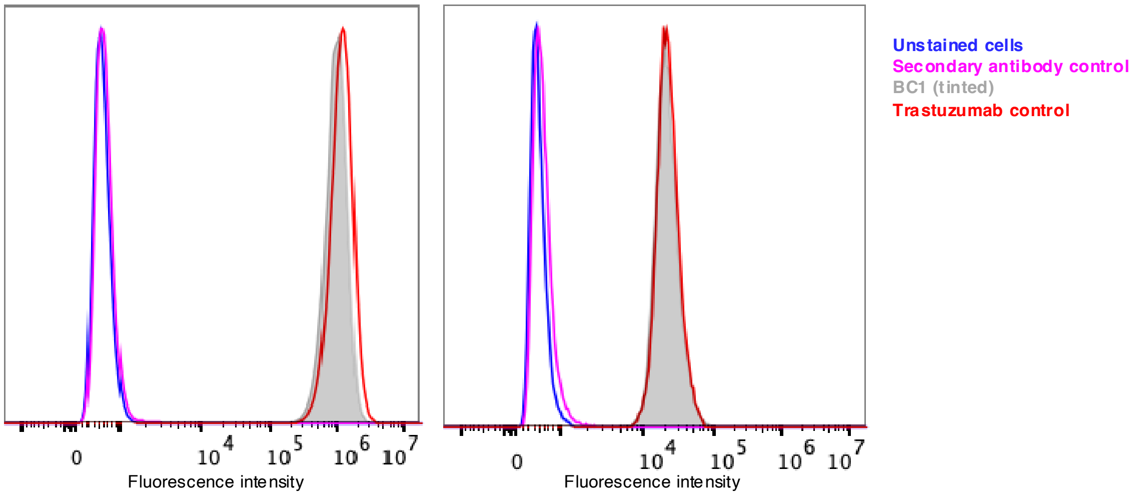

Figure S- 30 ***a.*** *Biolayer Interferometry of BC1 with against Human HER2 receptor protein in concentrations 50 nM and 10 nM – 0.37 nM (3 – fold dilutions) and 50 nM of trastuzumab was used as control.* ***b.*** **K_D, k_a and k_d values of Trastuzumab and Enhertu (antibody drug conjugate (ADC) with DAR 8 (Drug to antibody ratio) of trastuzumab, used in clinic) provided by the equipment manufacturer versus obtained values. The response curves for BC1 exhibit slightly higher response than is observed for trastuzumab and this expected since BC1 have slightly higher molecular weight than trastuzumab. Similarly as found to manufacturer, in this assay format, trastuzumab exhibits a faster on-rate (k_a) than the ADCs as can be observed from a steeper association slope and the off-rate (k_d) was observed to be too slow to generate meaningful data and as a result, K_D, kd values may not be accurate. *Presented in* ***b.*** *are values obtaied from different assay format by the manufacturer (ADCs were immobilised and HER2 receptor was used in dilution series) versus ours. **The values extracted by us, where possible, where in the same order of magintude and followed the same trends, suggesting that BC1 binds the receptor similartly or as well as truastuzumab or FDA-aproved ADC Enhertu. Source: www.sartorius.com Mcbai et. Al Characterization of Trastuzumab Antibody-Drug Conjugates Using Bio-layer Interferometry and Advanced Flow Cytometry, accessed on 2025.02.05.*
c.d. *Flow cytometry analysis of specificity of BC1 versus trastuzumab in HER2 positive cell line SKBR3 (**c.**) and HER2 low cell line MCF7 (**d.**) tinted grey represent BC1 binding, blue line represents unstained cells, pink correspond to secondary antibody control and red corresponds to trastuzumab binding (positive control).*

Figure S31 Stability of **BC1** in PBS

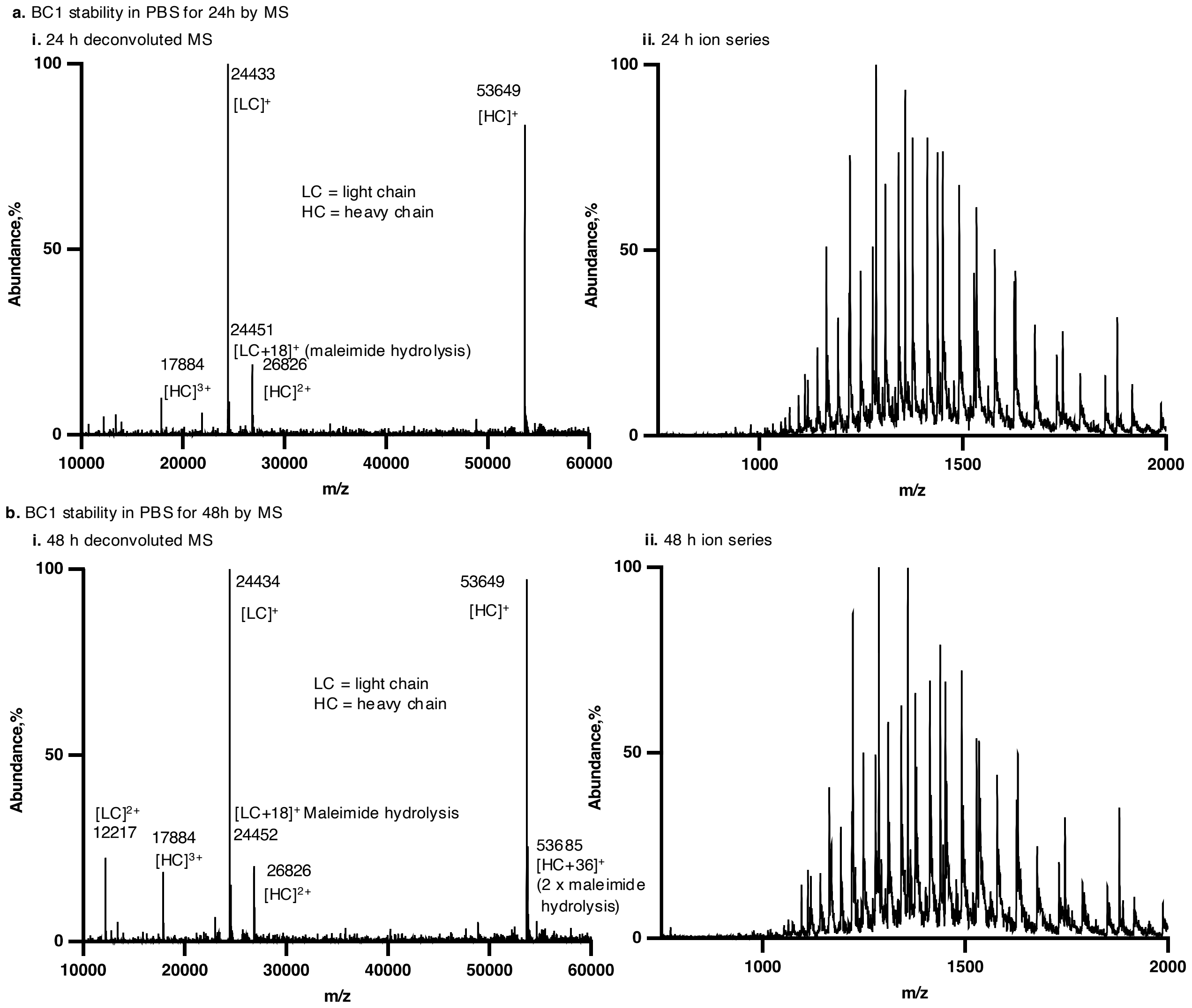

Figure S- 31 Conjugate BC1 was stable in PBS 37°C for at least 48h by mass spectrometry (MS), since no change in mass is observed. Spontaneous CO release would result in the mass reduction in both heavy and light chains. Only maleimide hydrolysis is observed (mass +18 (1x H_2O) or mass +36 (2 x H_2O)) which is normal for maleimide–based conjugates. Aldehyde is stable towards free lysine since no reduction of mass (by factor of -18 for H_2O) is observed at any timepoint. The stability of the conjugate was not tested in human serum, since the stability of CO-prodrugs ***4a/4b*** *was shown (towards aldehyde/sulfur oxidation or CO release).* ***a. i.*** *deconvoluted mass spectra (MS) ESI+ for BC1 after 24 h,* ***ii.*** *ion series of the mass spectrum,* ***b. i.*** *deconvoluted mass spectra (MS) ESI+ for BC1 after 48 h* ***ii.*** *ion series of the mass spectrum. PBS – phosphate-buffered saline.*

Figure S32 Bioconjugate control – Alexa Fluor™ 488 C_5 maleimide

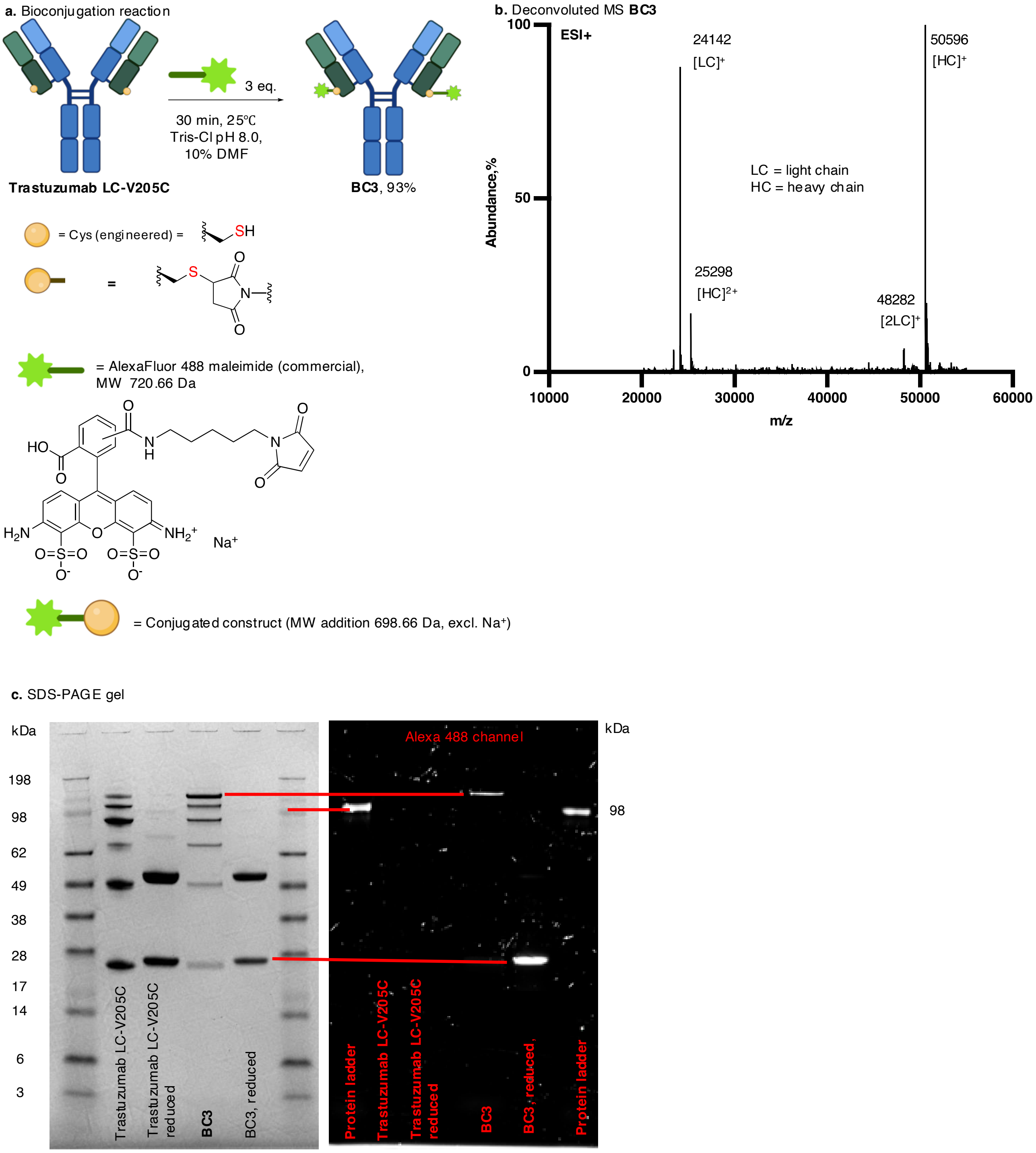

Figure S- 32 ***a.*** *Schematic of the reaction of engineered cysteine (LC V205) tagged antibody with commercial reagent Alexa Fluor 488 nm maleimide* ***b.*** *deconvoluted mass spectra (MS) ESI+ for the bioconjugate,* ***BC3*** *(calcd. For LC 24,142 Da, HC 50,596 Da, found LC 24,142 Da, HC 50,596 Da)* ***c.*** *SDS-PAGE of protein conjugate* ***BC3*** *exposing with visible light or 488 nm laser beam in both non-reducing and reducing conditions. Both SDS-PAGE and MS suggest that engineered cysteine tagging with Alexa Fluor 288 nm went to full conversion, adding to the light chain (LC).*

Figure S33 Receptor saturation

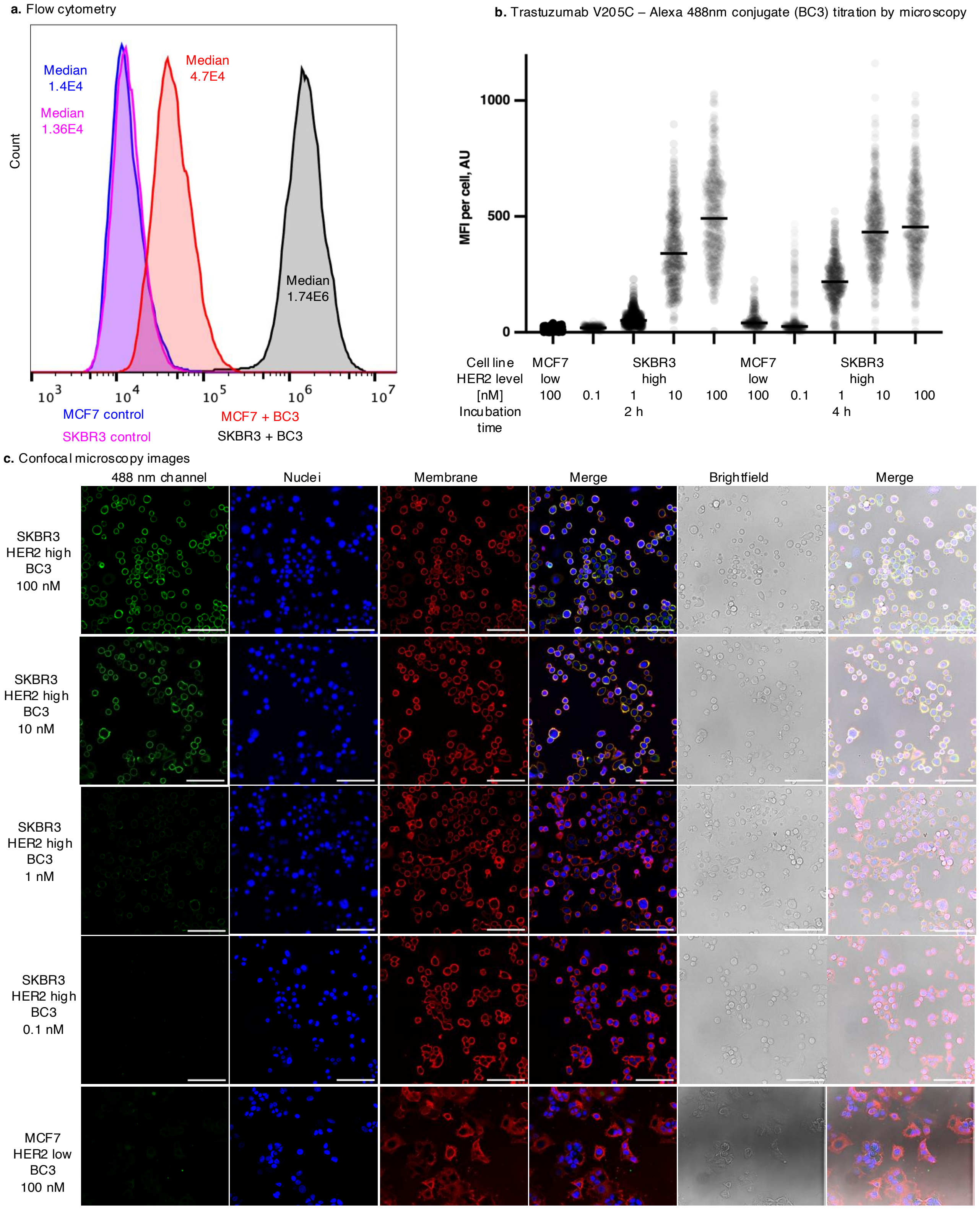

Figure S- 33. ***a.*** *Flow cytometry analysis of HER2-high SKBR3 and HER2-low MCF7 surface receptor levels using BC3-tagged trastuzumab as a stain for flow cytometry.* ***b.*** *Fluorescence quantification (mean fluorescence intensity per cell) in as close as possible conditions to turn on 1-Ac CO fluorescence imaging, suggested that HER2 receptors get close to saturation over 2h at 10 nM, while over 4h the receptors are fully saturated at 10 nM (100 nM suggested receptor oversaturation at both timepoints). This experiment has suggested the concentration range to be tested for turn-on fluorescence of 1-Ac CO-probe for CO-release experiments from CO- releasing bioconjugate BC1.*

c. *confocal microscopy images (used for analysis in* ***b.****) of SKBR3 and MCF7 (breast cancer, HER2 high and low, respectively) treated with a HER2 binder trastuzumab labelled with Alexa Fluor 488 for 2 h (****BC3****) in different concentration. Green – Alexa Fluor 488 labelled binder (****BC3****) Red - Wheat Germ Agglutinin – Alexa Fluor 647 conjugate stain for cell membrane (binding to sugar moieties on the membrane glycoproteins), blue – DAPI nuclear stain. A clear membrane localisation after multiple washing steps was observed with BC3, observed by colocalization of Alexa Fluor 488 (green) and Alexa Fluor 647 channels (red) resulting in yellow colour in merged images.*

Figure S34 CO release from Bioconjugates – extended figure

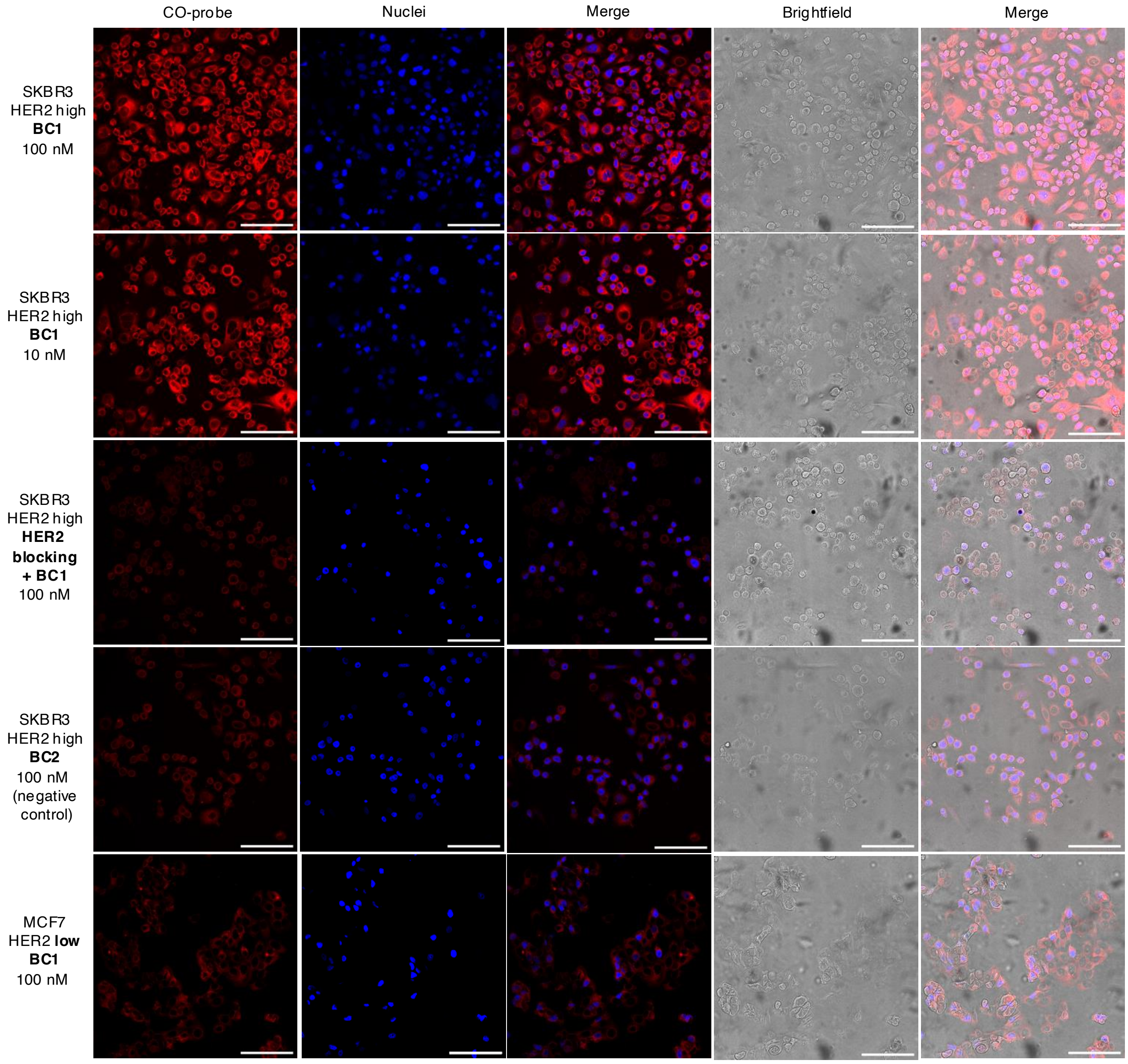

Figure S- 34 Confocal microscopy images of SKBR3 and MCF7 (breast cancer, HER2 high and low, respectively) cells for cellular CO release in cells treated with CO-releasing bioconjugate ***BC1*** *(100 nM, 10 nM) or non-releasing control (**BC2**, 100 nM) using 1-Ac CO probe. After an initial 30 min of cell pre-treatment with CO probe (1-Ac, 5 μM) the cells were washed and bioconjugates or controls were added for 2 hours. After the incubation, cells were washed, fixed and imaged by confocal microscope using DAPI for nulei (blue) and 561 nm channel (red, λ_{ex} = 561 nm, λ_{em} = 570 – 620 nm) for endogenous CO (CO probe turn-on response). White bar represents 100 μm. Turn-on 1-Ac CO probe fluorescence was significant when HER2-high SKBR3 cells were treated with 100 nM and 10 nM of BC1 (n.s. at 1 nM, data not shown), while no turn-on fluorescence was observed in HER2-low MCF7 cells. When HER2-high SKBR3 cells were pre-treated with non-fluorescent trastuzumab (i.e. receptors were blocked), no significant turn-on fluorescence on BC1 100 nM was observed, suggesting that CO is released following CO-carrier is binding to HER2 receptors on the membrane and partial/transient internalisation.*

2 Experimental procedures

2.1 General experimental

All non-aqueous reactions were performed in oven-dried or heat gun-dried glassware under nitrogen atmosphere unless otherwise stated. Nitrogen gas was pre-dried via passage through calcium chloride. Reaction vessels were heated using thermostatically controlled DrySyn blocks filled with sand. Reaction temperatures refer to the thermostat set point. A reaction temperature of 0 °C refers to an external ice/water slurry cooling bath, -78 °C refers to acetone/dry ice bath.

Solvents and Reagents. The chemicals were purchased from commercial sources and used without further purification unless stated otherwise. Solvents were distilled on site under an inert atmosphere: DCM, MeOH and MeCN were distilled from calcium hydride; THF and Et2O were pre-dried over sodium wire then distilled from calcium hydride and lithium aluminium hydride. EtOAc was distilled on site. Anhydrous DMF was bought for Sigma Aldrich and used as supplied. Water used experimentally was deionised and prepared on site.

Compound purification. Flash column chromatography was performed using silica gel (Silica gel 60 Å,40 – 63 μm, Merck). Analytical thin layer chromatography was performed using Merck Silica gel 60 F254 1 mm glass plates and visualised by UV (254 nm) and/or by staining with $KMnO_4$ stain or other suitable stain using known procedures.

Preparative High Performance-Liquid Chromatography (Prep-HPLC) was performed on a HPLC Agilent Infinity 1260 II system with a semi-preparative Column ZORBAX 5 Eclipse Plus C18 21.2 x 250 mm (ID x L) P. N. 595250-902 or ZOBRAX 5 Eclipse Plus C8 21.2 x 150 mm (ID x L) P. N. 595150-906. Before purification, the crude product was dissolved in 1:1 MeCN/H_2O and sterile filtered at 0.22 μm with EMD Millipore™ polyether sulfone syringe filters. Crude product aliquots were injected into the column and flow was maintained at 20.0 mL min^{-1} with solvent mixtures of MeCN/H_2O + 0.1% TFA, unless otherwise noted. UV-vis absorbance was monitored at 210 and 254 nm to indicate the collection of the fractions. MeCN was evaporated from the fractions under a nitrogen flow, and the remaining H_2O/TFA solution was flash frozen and lyophilised.

2.2 Characterisation

NMR: ^{1}H and ^{13}C NMR spectra were recorded on Bruker 400-Neo Prodigy cryoprobe, Bruker 500-AVIII Dual ^{13}C 1H cryoprobe, Bruker 500- AVIII or Bruker 700-AVII+ TCO cryoprobe NMR machines. Chemical shifts are reported in parts per million (ppm) with spectra calibrated to the residual solvent peaks (^{1}H NMR: $CDCl_3$ δH 7.26 ppm, DMSO-d6 δH 2.50 ppm, CD_3OD δH 3.31 ppm, Acteone-d6 δH 2.05 ppm, ^{13}C NMR: $CDCl_3$ δC 77.2 ppm, DMSO-d6 δC 39.5 ppm, CD_3OD δC 49.0 ppm, Acteone-d6 δC 29.8 ppm). All NMR measurements were acquired at 298 K, unless otherwise stated. MestReNova software (v. 15.0.0) was used for spectral processing. Multiplicities are described as s (singlet), d (doublet), t (triplet), q (quartet), m (multiplet), dd (double doublet) etc. Coupling constants (J) are reported in hertz (Hz). The centre of each peak is reported except for multiplet signals where a range of ppm values are given. ^{1}H NMR DOSY was performed by NMR-service team at the Yusuf Hamied Department of Chemistry, University of Cambridge.

Mass spectrometry of protein and peptide samples. LC–MS analysis of protein samples was carried out using a Waters SQD2 mass spectrometer using inlet method A for protein and B for peptides, in combination with an Acquity UPLC system with an Acquity UPLC BEH300 C4 column (130 Å1.7 μm, 2.1 × 50 mm) for proteins or an Acquity UPLC BEH C18 column (130 Å1.7 μm, 2.1 × 50 mm) for peptides and small molecules. The SQD2 mass spectrometer mobile phase consisted of solvent A (99.9% water, 0.1% formic acid), solvent B (99.9% CAN, 0.1% formic acid. Gradient methods were as follows: inlet method A: 5% to 72% B in 6 min, then 72% B for 1.5 min followed by a gradient from 72% to 5% B over 0.25 mins and finally, 95% A for 1.25 mins. Inlet method B: 95% A for 0.5 mins, followed by a gradient for 5% to 90% A over 5.5 mins, then 90% B for 2.5 min followed a gradient from 90% to 5% B over 0.25 min and finally, 95% A for 3.25 min. The capillary voltage of the electrospray source for the Waters SQD2 mass spectrometer was 3.0 kV with a cone voltage of 30 V and the desolvation gas used was nitrogen, with a flow rate of 800 L h−1. The ion series was obtained through integration of the major peaks of the chromatogram. Following this, the total mass spectra were reconstructed using the MaxEnt1 algorithm on the MassLynx software (v. 4.1), according to manufacturer's guidelines.

High resolution mass spectrometry (HRMS) of small molecules. Accurate mass measurements were performed with an Agilent 1260 Infinity LC System coupled to an Agilent 6230 time-of-flight (TOF) LC/MS System (Agilent Technologies, Santa Clara, CA). The Agilent 1260 LC module was equipped with a variable wavelength (VWD) detector along with a binary solvent pump and an autosampler. Chromatographic separations were performed with an Poroshell 120 EC-C18 reverse phase C18 column 3.0 mm × 50 mm; 2.7 µm (Agilent Technologies, Santa Clara, CA). The pump was connected to a gradient binary solvent system: A, H_2O and B, MeCN, and 0.1 % formic acid (v/v) or 10 mM ammonium formate modifiers added after column separation for positive and negative polarity runs respectively. The mobile phase was programmed to run as follows: 0–1.5 min, 95% A and 5% B to 0% A and 100% B; 3-6 min, 100% B; and return to starting mobile phase composition with re-equilibration. The ionization source was Agilent Jet Stream, with electrospray ionization (ESI) in either positive or negative modes for acquisition of mass spectra. The elution was run at a flow rate of 0.4 mL/min. UV spectra were monitored 254 nm. Injection volume was 1 μL for each of the sample solutions, followed by needle wash. The column temperature was maintained at 40°C. Nitrogen was supplied from a centralised reservoir and used as the drying and nebulizer gas. Other MS instrumental conditions are as follows: drying gas temperature and flow rate were 325°C and 10.0 L/min, respectively; nebulizer pressure was 40 psi; sheath gas temperature and flow rate were 400°C and 12.0 L/min, respectively; capillary, nozzle and fragmentor were set to 4000 V, 500V and

200 V respectively; skimmer was 65.0; Oct 1 RF was 750. The instrument state was set to extended dynamic range mode (2 GHz). Data collection and integration were performed using MassHunter WalkUp Console software (version 4.1 Build 4.1.313). The data was collected in the range of 100 and 3000 m/z. Data were stored in both centroid and profile formats during acquisition. Two independent reference lock-mass ions, (purine ($C_5H_4N_4$ at *m/z* 121.0509 and HP-921 (*hexakis-(1H,1H,3H-tetrafluoro-pentoxy)phosphazene*) ($C_{18}H_{18}O_6N_3P_3F_{24}$) at *m/z* 922.0098, were employed using a low flow of the calibrants solution (calibrant solution A, Agilent Technologies) to ensure mass accuracy and reproducibility.

GC-MS experiment was run on Agilent 5977C GCMS by Mass spectrometry service at Yusuf Hamied Department of Chemistry, University of Cambridge. Data collection and integration were performed using MassHunter WalkUp Console software (version 4.1 Build 4.1.313).

SDS-PAGE was done with NuPAGE™ Bis-Tris Mini Protein Gels, 4–12%, 1 .0–1.5 mm and NuPAGE™ MES SDS Running Buffer (20X) diluted in MQ, at a constant voltage of 200 V and using Invitrogen™ SeeBlue™ Plus2 Pre-stained Protein Standard and stained with Instant Blue™ Coomassie protein stain. Gels were imaged using Bio-Rad ChemiDoc. and quantified, when required, using Fiji ImageJ2 (version 2.14.0/1.54f).

Data analysis. GraphPad Prism v10.14.1 was used to generate graphs and for statistical student t-test and ANOVA analysis. Data sets with a Gaussian distribution were analysed by parametric unpaired t test and data sets that did not pass the normality tests were analysed by nonparametric unpaired Mann–Whitney test. Clearance data sets were analysed using Fisher's exact test. All were two-sided tests with a confidence interval of 95%.

Cartoon representations were created with *BioRender.*

2.3 Chemical synthesis

2.3.1 Synthesis of the main compounds

<u>2-ethyl-2-(hydroxymethyl)butyl 4-methylbenzenesulfonate **2**</u>

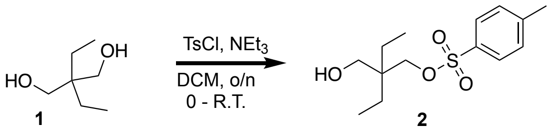

*Scheme S 1 Synthesis of **2***

2,2-Diethyl-1,3-propanediol **1** (1.35 g, 10.3 mmol. 1 eq.) was dissolved in DCM (40 mL) at 0 °C and *p*-toluenesulfonyl acid chloride (1.95 g, 10.2 mmol, 1 eq.) was added, following by a catalytic amount of 4-dimethylaminopyridine (67 mg, 0.55 mmol, 0.05 eq.). The reaction was allowed to reach room temperature and triethylamine (2.14 mL, 15.4 mmol, 1.5 eq.) was added dropwise and the reaction mixture was stirred for 18 hours. The reaction mixture was washed with aqueous HCl (10%, w/v, 3x), brine (2x). The aqueous layer was extracted with DCM (2x 30 mL). Combined organic layers were dried over $MgSO_4$ and the solvent was removed under reduced pressure. The residue was purified by flash chromatography (ethyl acetate/petroleum ether, EA/PET 20-70%) to yield product **2** as a colourless oil (1.95 g, 6.81 mmol, 67%) in over 95% purity (characterised by NMR).
^{1}H NMR (500 MHz, $CDCl_3$) δ 7.80 (d, *J* = 8.1 Hz, 2H), 7.35 (d, *J* = 8.1 Hz, 2H), 3.85 (s, 2H), 3.41 (s, 2H), 2.45 (s, 3H), 1.59 (s, 1H), 1.33 – 1.15 (m,4H), 0.75 (t, *J* = 7.5 Hz, 6H).
^{13}C NMR (126 MHz, $CDCl_3$) δ 144.9, 132.9, 129.9, 127.9, 71.7, 63.6, 41.6, 21.7, 21.6, 6.8.
R_f (3:7 EA/PET) =0.35
HRMS-ESI (*m/z*): calc. for $C_{14}H_{22}O_4S$, 286.1238; found ESI+: 287.1323 $[M+H]^+$ (diff. 3.7 ppm).

<u>2-ethyl-2-formylbutyl 4-methylbenzenesulfonate **3**</u>

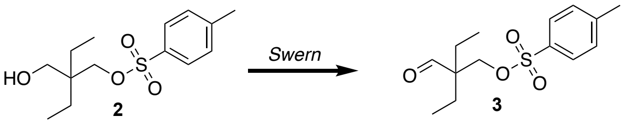

*Scheme S 2. Synthesis of **3***

The aldehyde **3** was synthesised from **2** following *Swern* oxidation procedure. Briefly, oxalyl chloride ($(COCl)_2$, 1.80 mL, 20.4 mmol, 3 eq.) was dissolved in DCM (18 mL) and the solution was cooled to -78 °C. Then, anhydrous DMSO (2.9 mL, 41 mmol, 6 eq.) was added dropwise, and the mixture was stirred for 10 min, following by the dropwise addition of the alcohol **2** (1 eq. 1.95 g, 6.80 mmol in 5 mL DCM) and the reaction mixture was stirred for another 20 min. Lastly, NEt_3 (9.50 mL, 68.0 mmol, 10 eq.) was added dropwise, and the reaction was allowed to reach room temperature over the period of 3 hours. Then, the mixture was diluted with DCM, washed with aqueous HCl (5% w/v, 2x), dried over $MgSO_4$ and the solvent was removed under reduced pressure to yield the product. The crude was purified by flash column chromatography on silica gel to remove baseline impurities (1:9 EA/PET) and yield the product **3** as a colourless to yellowish oil (1.78 g, 6.3 mmol, 92%) in over 95% purity (characterised by NMR).
^{1}H NMR (500 MHz, $CDCl_3$) δ 9.35 (s, 1H), 7.79 (d, *J* = 8.1 Hz, 2H), 7.36 (d, *J* = 8.1 Hz, 2H), 4.06 (s, 2H), 2.46 (s, 3H), 1.68 – 1.52 (m, 4H), 0.74 (t, *J* = 7.6 Hz, 6H).
^{13}C NMR (126 MHz, $CDCl_3$) δ 203.8, 145.2, 132.5, 130.1, 128.2, 68.4, 53.3, 23.3, 21.8, 7.8.
HRMS-ESI (*m/z*): calc. for $C_{14}H_{20}O_4S$, 284.1082; found ESI+ 285.1147 $[M+H]^+$ (diff. 2.7 ppm).

R_f (1:9 EA/PET) = 0.45

General tosyl-thiol S_{N2} reaction procedure.

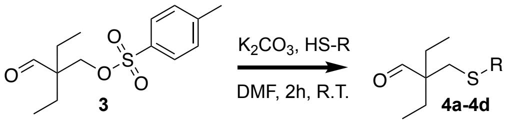

Scheme S 3 General synthetic procedure for compounds ***4a-4d***

Aldehyde (1eq.) was dissolved in DMF (*ca.* 2 mL) under inert conditions and the thiol (1.5 eq.) was added. This was followed by anhydrous K_2CO_3 (3eq.). The mixture was stirred at room temperature for 3 hours before the reaction progress was checked by LC-MS or TLC. The reaction mixture was poured into deionised water or 10% HCl (in case of carboxylic acid-containing compounds) and extracted with ethyl acetate (3x), then the organic layer was washed with water, brine, dried over $MgSO_4$ and the solvent was removed under reduced pressure. The crude was then purified as described in relevant section.
N.B. Strict oxygen-free conditions are required for this reaction as it leads to oxidation of thiols to disulfide. If the reaction goes sluggishly, additional step of tosyl- exchange to iodide (similar as described in synthesis of **4e**) or the use of tosyl can be swapped to mesyl- to reduce steric bulk since the very start of the synthetic scheme.

N-acetyl-*S*-(2-ethyl-2-formylbutyl)-*L*-cysteine **4a**

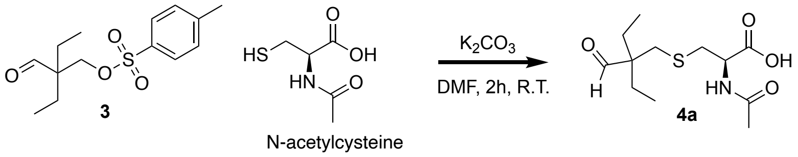

Scheme S 4 Synthesis of 4a

CO-prodrug **4a** was synthesised from **3** (470 mg, 1.65 mmol) and N-acetylcysteine (NAC) according to **General tosyl-thiol S_{N2} reaction procedure** and purified by prep-HPLC to yield the product **4a** as colourless oil (270 mg, 0.98 mmol, 59%) in over 95% purity (characterised by NMR).
^{1}H NMR (400 MHz, $CDCl_3$) δ 9.42 (s, 1H), 6.60 (d, *J* = 7.2 Hz, 1H), 4.81 (dt, *J* = 7.2, 5.1 Hz, 1H), 3.05 (d, *J* = 5.1 Hz, 2H), 2.78 (s, 2H), 2.11 (s, 3H), 1.70 – 1.55 (m, 4H), 0.85-0.79 (m, 6H).
^{13}C NMR (101 MHz, $CDCl_3$) δ 205.9, 173.0, 171.6, 53.6, 52.3, 35.3, 34.8, 25.0, 24.9, 23.1, 8.1, 8.0.
HRMS-ESI (*m/z*): $[M+H]^+$ calc. for $C_{12}H_{21}O_4S$, 275.1199; found 276.1271 $[M+H]^+$ (diff. 2.39 ppm).

methyl N-acetyl-S-(2-ethyl-2-formylbutyl)-L-cysteinate **4b**

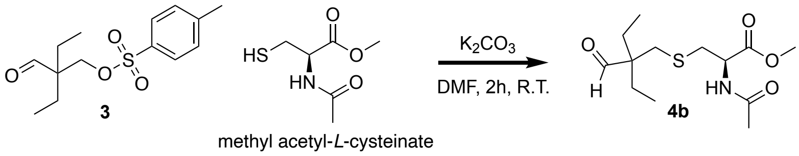

Scheme S 5 Synthesis of 4b

CO-prodrug **4b** was synthesised from **3** (332 mg, 1.17 mmol) and methyl acetyl-*L*-cysteinate (NAC-Me) according to **General tosyl-thiol S_{N2} reaction procedure** and purified by prep-HPLC to yield the product as colourless oil **4b** (175 mg, 0.61 mmol, 52%) in over 95% purity (characterised by NMR).

^{1}H NMR (400 MHz, MeOD) δ 9.41 (s, 1H), 4.65 (dd, *J* = 8.2, 5.3 Hz, 1H), 3.74 (s, 3H), 3.00 (dd, *J* = 13.9, 5.3 Hz, 1H), 2.89 – 2.81 (m, 1H), 2.83 – 2.73 (m, 2H), 2.00 (s, 3H), 1.62 (q, *J* = 7.5 Hz, 4H), 0.81 (t, *J* = 7.5 Hz, 6H).
^{13}C NMR (101 MHz, MeOD) δ 207.0, 173.3, 172.6, 54.5, 53.7, 52.9, 35.8, 35.3, 25.4, 25.4, 22.3, 8.2.
HRMS-ESI (*m/z*): $[M+H]^+$ calc. for $C_{13}H_{23}O_4S$, 289.1352; found ESI+ 323.1241 $[M+Na]^+$ (diff. 0.25 ppm).

2-ethyl-2-(((4-methyl-2-oxo-2*H*-chromen-7-yl)thio)methyl)butanal **4c**

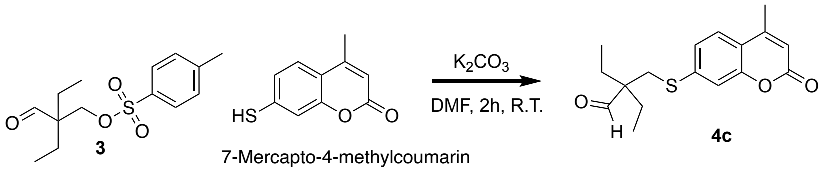

Scheme S 6 Synthesis of 4c

CO-prodrug **4c** was synthesised from **3** (103 mg, 0.36 mmol) and 7-Mercapto-4-methylcoumarin (105 mg, 0.55 mmol, 1.5 eq.) according to **General tosyl-thiol S_{N2} reaction procedure** and purified by prep-HPLC to yield the product as white amorphous solid **4c** (70 mg, 0.23 mmol, 63%) in over 95% purity (characterised by NMR).
^{1}H NMR (400 MHz, Acetone) δ 9.53 (s, 1H), 7.68 (d, *J* = 8.9 Hz, 1H), 7.32 (m, 2H), 6.24 (d, *J* = 1.3 Hz, 1H), 3.35 (s, 2H), 2.46 (d, *J* = 1.3 Hz, 3H), 1.75 (q, *J* = 7.5 Hz, 4H), 0.84 (t, *J* = 7.5 Hz, 6H).
^{13}C NMR (101 MHz, Acetone) δ 205.1, 160.3, 154.9, 153.4, 143.8, 126.2, 124.2, 118.3, 115.29, 114.6, 53.9, 34.9, 25.2, 18.4, 8.2.
HRMS-ESI (*m/z*): $[M+H]^+$ calc. for $C_{17}H_{20}O_3S$ 304.1127 found ESI+ 305.1202 $[M+H]^+$ (diff. 1.24 ppm).

2-(((9*H*-purin-6-yl)thio)methyl)-2-ethylbutanal **4d**

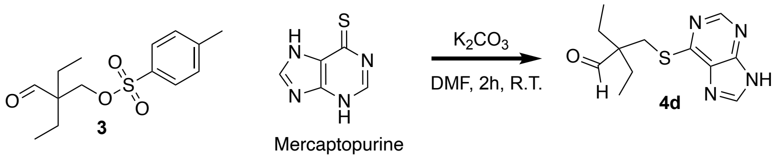

Scheme S 7 Synthesis of 4d

CO-prodrug **4d** was synthesised from **3** (10.3 mg, 0.036 mmol) and Mercaptopurine (8.3 mg, 0.054 mmol, 1.5 eq.) according to **General tosyl-thiol S_{N2} reaction procedure** and purified by prep-HPLC to yield the product as white amorphous solid **4d** (4.0 mg, 0.036 mmol,42%) in over 95% purity (characterised by UPLC).
^{1}H NMR (400 MHz, Acetone) δ 9.55 (s, 1H), 8.66 (s, 1H), 8.34 (s, 1H), 3.74 (s, 1H), 1.75 (q, *J* = 7.6 Hz, 4H), 0.87 (t, *J* = 7.6 Hz, 6H).
^{1}H NMR (400 MHz, $CDCl_3$) δ 9.31 (s, 1H), 8.40 (s, 1H), 3.37 (s, 2H), 1.52 (br, 4H), 0.95 (t, *J* = 7.4 Hz, 6H).
Due to poor solubility of the compound in common NMR solvents, only poor-quality spectra was acquired (product was precipitating), *i.e.,* attempts to obtain ^{13}C NMR spectrum were not fruitful. UPLC-MS data presented in Figure S35.
HRMS-ESI (*m/z*): $[M+H]^+$ calc. for $C_{12}H_{16}N_4OS$ 264.1056 found ESI+ 265.1129 $[M+H]^+$ (diff. 4.32 ppm).

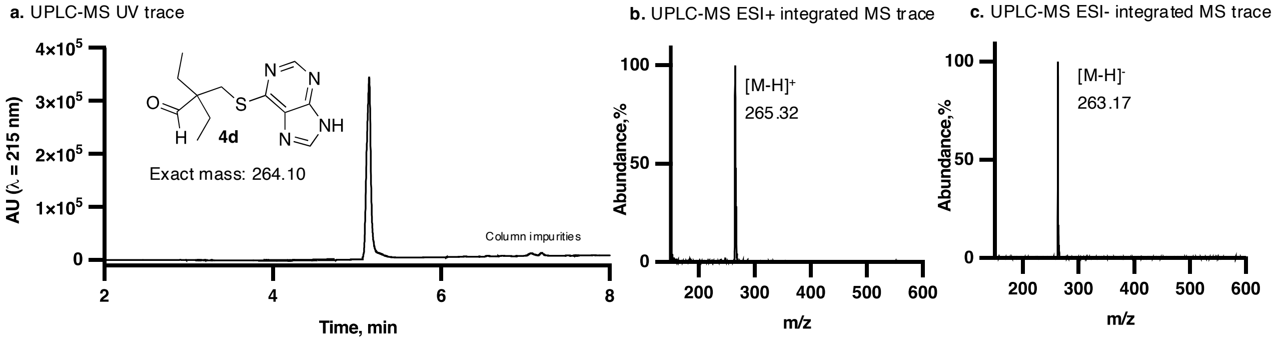

*Figure S- 35 UPLC-MS trace and mass spectrum of purified **4d***

N-(((9*H*-fluoren-9-yl)methoxy)carbonyl)-*S*-(2-ethyl-2-formylbutyl)-*L*-cysteine **4e**

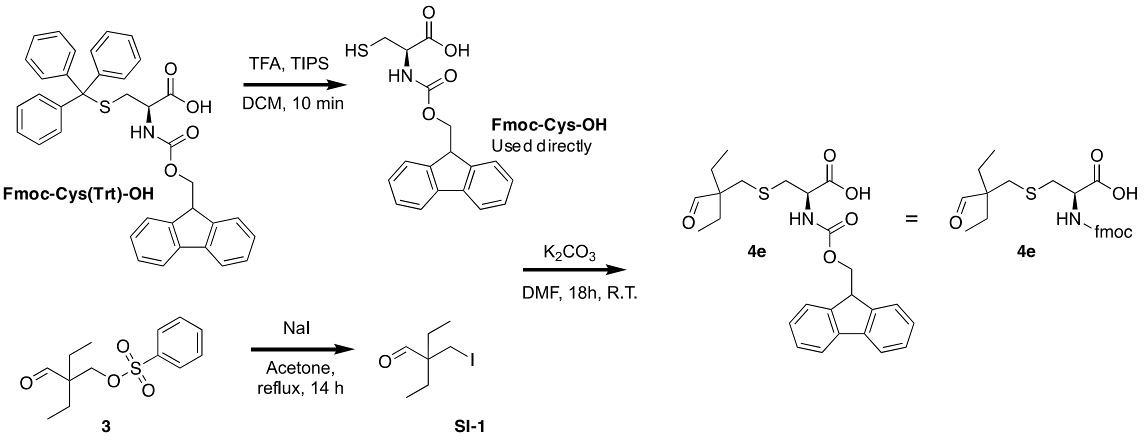

Scheme S 8 Synthesis of 4e

i) **Fmoc-Cys-OH** was prepared from **Fmoc-Cys(Trt)-OH**. Briefly, **Fmoc-Cys(Trt)-OH** (2.85 g, 4.87 mmol) was resuspended in DCM (20 mL) and triisopropyl silane (1.1 mL, 850 mg, 5.35 mmol, 1.1 eq.) was added following by TFA (3.75 mL, 5.55 g, 48.7 mmol, 10 eq.). The reaction mixture was stirred at room temperature until the bright orange solution turned colourless (10 min). The reaction mixture was concentrated under reduced pressure and co-evaporated with Et_2O to remove remaining TFA (3x). The residue was resuspended in hexane and centrifuged after which supernatant was discarded. The cycle was repeated 5 times, and the pellet was further dried under reduced pressure to yield the product as a white amorphous solid (1.67 g, 4.87 mmol, 100%). The solid was used without further purification.

ii) Next, since the **General S_{N2} reaction procedure** resulted in no reaction, compound **3** (131 mg, 0.48 mmol) was dissolved in anhydrous acetone (4 mL) and NaI was added (108 mg, 0.72 mmol, 1.5 eq.). The mixture was refluxed overnight (14 h) under inert atmosphere and then cooled down. The precipitate was removed by filtration and further washed with acetone, and the solvent from the filtrate was removed under reduced pressure. The resulting oil was used in the next step without further purification. Then, the oil was dissolved in DMF (2 mL) under strict inert conditions (argon atmosphere) and **Fmoc-Cys-OH** (247 mg, 0.72 mmol, 1.5 eq.) was added, following by K_2CO_3 (133 mg, 0.98 mmol, 2 eq.) and the reaction was stirred under room temperature for 18 hours. The reaction mixture was poured into deionised water and extracted with ethylacetate (x3), then the organic layer was washed with water, brine, dried over $MgSO_4$ and the solvent was removed under reduced pressure. The crude was dissolved in 1:1 MQ-water/acetonitrile mixture and TCEP (68 mg, 0.24 mmol 0.5 eq.) was added and stirred for 30 minutes at room temperature. TCEP reduction was necessary since the disulfide byproduct coeluted with the product. Then, the mixture was filtered (0.22 µm with EMD Millipore™ polyethersulfone syringe filter) and loaded directly onto prep-HPLC, pure fractions were lyophilised to yield the product as viscous colourless oil (120 mg, 0.26 mmol, 27%) in over 95% purity (characterised by NMR).

^{1}H NMR (700 MHz, $CDCl_3$) δ 9.42 (s, 1H), 9.00 (s, 1H), 7.76 (d, *J* = 7.5 Hz, 2H), 7.61 (t, *J* = 7.5 Hz, 2H), 7.40 (t, *J* = 7.5 Hz, 2H), 7.31 (t, *J* = 7.5 Hz, 2H), 5.76 (d, *J* = 8.0 Hz, 1H), 4.69 – 4.64 (m, 1H), 4.41 (d, *J* = 7.3 Hz, 2H), 4.24 (t, *J* = 7.3 Hz, 1H), 3.11 – 3.00 (m, 2H), 2.86 – 2.72 (m, 2H), 1.65 - 1.57 (m, 4H), 0.80 (t, *J* = 7.6 Hz, 6H).

^{13}C NMR (176 MHz, $CDCl_3$) δ 205.8, 175.0, 156.2, 143.8, 143.8, 141.4, 127.9, 127.2, 125.2, 120.2, 67.7, 53.7, 53.6, 47.3, 35.8, 34.8, 24.9, 8.1

HRMS-ESI (*m/z*): calc. for $C_{25}H_{29}NO_5S$ 455.1794; found ESI+ 478.1679 $[M-Na]^+$ (diff. 4.2 ppm).

2.3.2 Synthesis of the supporting compounds

N-acetyl-*S*-(2-ethyl-2-formylbutyl)-*L*-selenocysteine **SI-4**

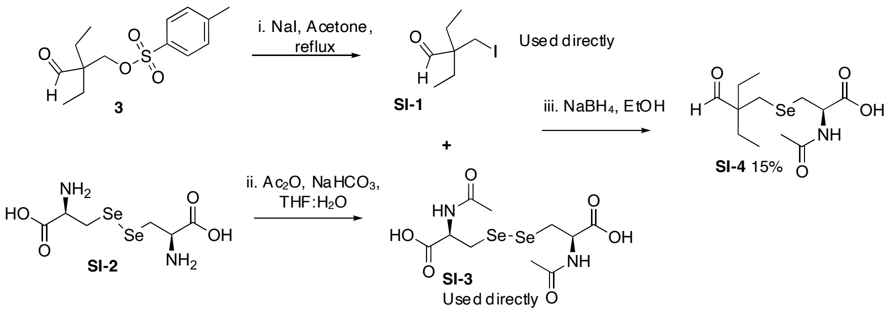

Scheme S 9 Synthesis of SI-4

The procedures were similar as reported by Nucci et al [1]

i) Similarly as for compound **4e**, compound **3** (60 mg, 0.21 mmol) was dissolved in anhydrous acetone (2 mL) and NaI was added (47 mg, 0.32 mmol, 1.5 eq.). The mixture was refluxed overnight (14 h) under inert atmosphere and then cooled down. The precipitate was removed by filtration and further washed with acetone, and the solvent from the filtrate was removed under reduced pressure. The resulting oil **SI-1** was dissolved in ethanol (2 mL) under strict inert conditions (argon atmosphere).

ii) Separately, L-Selenocystine **SI-2** (35 mg, 0.21 mmol, 1 eq.) and sodium bicarbonate (35 mg, 0.42 mmol, 2 eq.) was dissolved in a degassed THF: water (90:10 v/v, 2 mL) and then was stirred at room temperature for 20 min under nitrogen. The reaction was cooled to 0 °C and acetic anhydride (22 mg, 20 µL, 0.21 mmol, 1eq.) was added. The reaction was stirred for 2 h at room temperature under nitrogen. Then, the clear solution was cooled and acidified to pH 1 with 10% HCl and extracted with ethyl acetate (3 x 10 mL). The organic layer was dried under $MgSO_4$ and solvent was evaporated under reduced pressure and further dried in under vacuum overnight to yield **SI-3.**

Then, the residue was suspended in anhydrous EtOH (2 ml) under nitrogen atmosphere. Solid $NaBH_4$ (35 mg, 0.42 mmol., 2eq.) was added in one portion and the yellow mixture was stirred for 15 min, until clear and colourless. To this solution, an iodide solution made in step i) was added dropwise and the mixture was stirred for 2 hours. The reaction was quenched by addition of aq 10 % NH_4Cl (10 mL), and the mixture was shaken with ethyl acetate and brine until neutral. The organic layer was dried under $MgSO_4$, the solvent was removed under reduced pressure and then purified following prep-HPLC procedure to yield the product as colourless oil (10 mg, 0.031 mmol, 15%) in over 90% purity (by ^{1}H NMR).

^{1}H NMR (400 MHz, $CDCl_3$) δ 9.43 (s, 1H), 6.73 (d, *J* = 7.4 Hz, 1H), 4.90 (dt, *J* = 7.4, 5.0 Hz, 1H), 4.41 (s, br, 1H), 3.14 – 3.00 (m, 2H), 2.82 (s, 2H), 2.14 (s, 3H), 1.69 – 1.58 (m, 4H), 0.84 (dt, *J* = 9.8, 7.6 Hz, 6H).

^{13}C NMR (101 MHz, $CDCl_3$) δ 206.1, 172.8, 172.0, 53.5, 52.5, 27.7, 26.5, 25.8, 25.7, 22.9, 8.2, 8.1.

HRMS-ESI (*m/z*): $[M+H]^+$ calc. for $C_{12}H_{21}NO_4Se$, 317.0700; found ESI+ 318.0759 $[M+H]^+$ (diff. 2.87 ppm).

Compounds **SI-6** to **SI-9** were synthesised according to the reaction procedures of compounds **1** - **4,** using 2,2-dimethyl-1,3-propanediol **SI-5** as a starting material instead of **1,** and further following the same steps:

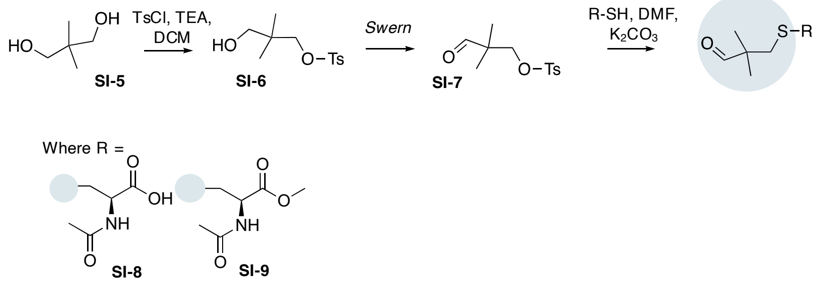

Scheme S 10 Synthesis of SI-6 to SI-9

<u>3-hydroxy-2,2-dimethylpropyl 4-methylbenzenesulfonate **SI-6**</u> was synthesised using 2,2-dimethyl-1,3-propanediol **SI-5** (4.83 g, 46.4 mmol, 1 eq.) and further purified by flash column chromatography 1:99 MeOH:DCM to yield the product **SI-6** as white amorphous solid (4.33 g, 16.8 mmol, 36%).
^{1}H NMR (500 MHz, $CDCl_3$) δ 7.78 (d, J = 8.0 Hz, 2H), 7.35 (d, J = 8.0 Hz, 2H), 3.81 (s, 2H), 3.37 (d, J = 4.2 Hz, 2H), 2.44 (s, 3H), 1.81 (s, 1H), 0.87 (s, 6H).
^{13}C NMR (126 MHz, $CDCl_3$) δ 144.9, 132.9, 129.9, 127.9, 75.1, 67.6, 36.6, 21.7, 21.0.
HRMS-ESI (*m/z*): calc. for $C_{12}H_{18}NO_4S$, 258.0929; found 259.1001[M-H]$^+$ (diff. 1.00 ppm).
<u>2,2-dimethyl-3-oxopropyl 4-methylbenzenesulfonate **SI-7**</u> was synthesised from **SI-6** (3.33 g, 12.9 mmol, 1 eq.) using the same conditions as synthesis of **3** to yield the product **SI-7** as a white amorphous solid (3.01 g, 11.7 mmol, 91%) in 90% purity (by ^{1}H NMR) and used without further purification.
^{1}H NMR (400 MHz, $CDCl_3$) δ 9.42 (s, 1H), 7.81 – 7.73 (m, 2H), 7.36 (d, J = 7.9 Hz, 1H), 4.00 (s, 2H), 2.46 (s, 3H), 1.09 (s, 6H).
^{13}C NMR (101 MHz, $CDCl_3$) δ 202.5, 145.2, 132.6, 130.1, 128.1, 73.2, 46.5, 21.7, 19.0.
HRMS-ESI (*m/z*): calc. for $C_{12}H_{16}NO_4S$, 256.0769; found ESI+ 257.0839 [M-H]$^+$ (diff. 1.13 ppm).
<u>*N*-acetyl-*S*-(2,2-dimethyl-3-oxopropyl)-*L*-cysteine CO-prodrug **SI-8**</u> was synthesised from **SI-7** (233 mg, 0.87 mmol) and N-acetylcysteine (NAC) according to **General tosyl-thiol S_{N2} reaction procedure** and purified by prep-HPLC to yield the product **SI-8** as colourless oil (59 mg, 0.24 mmol, 27%) in over 95% purity (characterised by NMR).
^{1}H NMR (500 MHz, $CDCl_3$) δ 9.46 (s, 1H), 6.80 (d, *J* = 7.4 Hz, 1H), 6.61 (s, 1H), 4.81 (dt, *J* = 7.4, 5.1 Hz, 1H), 3.06 (d, *J* = 5.2 Hz, 2H), 2.77 (d, *J* = 2.2 Hz, 2H), 2.14 (s, 3H), 1.15 (d, *J* = 7.4 Hz, 6H).
^{13}C NMR (126 MHz, $CDCl_3$) δ 205.1, 173.1, 172.6, 52.4, 47.3, 40.5, 35.5, 22.8, 21.8, 21.5.
HRMS-ESI (*m/z*): calc. for $C_{10}H_{17}NO_4S$, 247.1035; found 248.0950 [M-H]$^+$ (diff. 0.25 ppm).
<u>methyl *N*-acetyl-*S*-(2,2-dimethyl-3-oxopropyl)-*L*-cysteinate CO-prodrug **SI-9**</u> was synthesised from **SI-7** (180 mg, 0.70 mmol) and N-acetylcysteine methyl ester (NAC-Me) according to **General tosyl-thiol S_{N2} reaction procedure** and purified by prep-HPLC to yield the product **SI-9** as colourless oil (26 mg, 0.01 mmol, 14%) in over 95% purity (characterised by NMR).
^{1}H NMR (500 MHz, $CDCl_3$) δ 9.47 (s, 1H), 6.36 (d, *J* = 7.2 Hz, 1H), 4.84 (dt, *J* = 7.2, 5.0 Hz, 1H), 3.78 (s, 3H), 3.07 – 2.95 (m, 2H), 2.77 – 2.66 (m, 2H), 2.07 (s, 3H), 1.14 (d, *J* = 6.5 Hz, 6H).
^{13}C NMR (126 MHz, $CDCl_3$) δ 204.5, 171.3, 170.2, 52.9, 52.2, 47.2, 40.6, 36.4, 23.3, 21.7, 21.5.
HRMS-ESI (*m/z*): calc. for $C_{11}H_{19}NO_4S$, 261.1035; found 262.111 [M-H]$^+$ (diff. 1.5 ppm), 284.0927 [M-Na]$^+$ (diff. 0.01 ppm)

Compounds **SI-11** to **SI-12** were synthesised according to the reaction procedures of compounds **1** - **4,** only using 2,2-dimethoxy-1,3-propanediol **SI-10** instead of 2,2-diethyl-1,3-propanediol **1,** and further following the same steps.

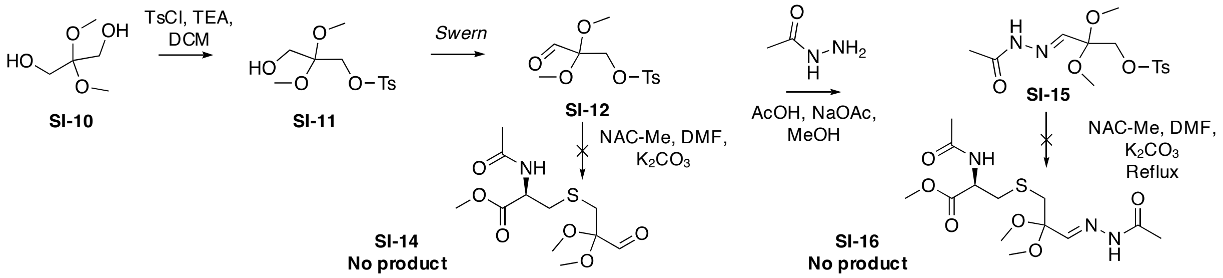

Scheme S 11 Synthesis of SI-11 tp SI-15

<u>3-hydroxy-2,2-dimethoxypropyl 4-methylbenzenesulfonate **SI-11**</u> was synthesised using 2,2-dimethoxyl-1,3-propanediol **SI-10** (935 mg, 6.87 mmol, 1 eq.) and further purified by flash column chromatography 20-60% ethyl acetate : petroleum ether to yield the product as **SI-11** colourless oil (511 mg, 1.76 mmol, 26%).

^{1}H NMR (400 MHz, $CDCl_3$) δ 7.84 – 7.76 (m, 2H), 7.35 (d, J = 8.5 Hz, 2H), 4.01 (s, 2H), 3.60 (s, 2H), 3.21 (s, 6H), 2.45 (s, 3H).

^{13}C NMR (101 MHz, $CDCl_3$) δ 145.2, 132.4, 130.0, 128.0, 99.5, 64.6, 59.5, 48.6, 21.7.

HRMS-ESI (*m/z*): calc. for $C_{12}H_{18}NO_6S$, 290.0836; found ESI+ 313.0724 $[M\text{-}Na]^+$ (diff. 2.30 ppm).

<u>2,2-dimethoxy-3-oxopropyl 4-methylbenzenesulfonate **SI-12**</u> was synthesis from **SI-11** (480 mg, 1.65 mmol, 1 eq.) using the same conditions as synthesis of **3** to yield the product **SI-12** as colourless oil (477 mg, 1.39 mmol, 64%) in 90% purity (by ^{1}H NMR) and used without further purification.

^{1}H NMR (500 MHz, $CDCl_3$) δ 9.43 (s, 1H), 7.76 - 7.74 (m, 2H), 7.36 - 7.43 (m, 2H), 4.09 (s, 2H), 3.28 (s, 6H), 2.45 (s, 3H).

^{13}C NMR (126 MHz, $CDCl_3$) δ 198.8, 145.6, 132.0, 130.1, 128.2, 98.5, 65.4, 50.0, 21.8.

HRMS-ESI (*m/z*): calc. for $C_{12}H_{16}NO_6S$, 288.0668; found ESI+ 311.0560 $[M\text{-}Na]^+$ (diff. 0.04 ppm).

<u>(*E*)-3-(2-acetylhydrazineylidene)-2,2-dimethoxypropyl 4-methylbenzenesulfonate **SI-15.**</u> **S-12** (43 mg, 0.15 mmol, 1 eq.) was dissolved in dry methanol (1 mL) to which acetohydrazide (12.2 mg, 0.16 mmol, 1.1 eq.), sodium acetate (9.0 mg, 0.15 mmol, 1 eq.) an d a drop of glacial acetic acid was added. The mixture was stirred for 1 hour, the solvent was removed under reduced pressure and directly purified by acid-free prep-HPLC to yield the product **SI-13** as amorphous white solid (46 mg, 0.13 mmol, 90%).

^{1}H NMR (400 MHz, $CDCl_3$) δ 9.12 (s, 1H), 7.80 – 7.73 (m, 2H), 7.34 (d, *J* = 7.9 Hz, 2H), 6.93 (s, 1H), 4.18 (s, 2H), 3.23 (s, 6H), 2.44 (s, 3H), 2.10 (s, 3H).

^{13}C NMR (101 MHz, $CDCl_3$) δ 173.5, 145.3, 142.3, 132.4, 129.9, 128.0, 93.3, 67.2, 49.1, 21.6, 20.1.

HRMS-ESI (*m/z*): calc. for $C_{14}H_{20}N_2O_6S$ 344.1054 found ESI+ 345.1112 $[M\text{-}H]^+$ (diff. 0.85 ppm).

2.4 Peptide synthesis

2.4.1 General peptide synthesis protocol

The following Fmoc-AA-OH with side-chain protecting groups were used for the synthesis of standard all L-AA-peptides: Fmoc-Cys(Trt)-OH, Fmoc-Gly-OH, Fmoc-Lys(Boc)-OH, Fmoc-Met-OH, Fmoc-Phe-OH, Fmoc-Ser(*t*-Bu)-OH, Fmoc-Thr(*t*-Bu)-OH, Fmoc-Trp(Boc)-OH, Fmoc-Tyr(*t*-Bu)-OH, the use of other amino acids or building blocks is stated in the given experiment.

Manual SPPS was performed in polypropylene syringes equipped with fitted disks. TentaGel™ S RAM (10 μmol, 43 mg, cat. No. 86407) resin was swelled in DMF (2.0 mL) for 30 min, washed with DMF (5 × 1 min) and peptides were synthesized beginning from the C-terminal by the iteration of the following steps:

Fmoc deprotection. The resin was treated with piperidine in DMF (1:4, v/v) (2 × 3 min) and then washed with DMF (5 × 1 min).

Standard coupling of an amino acid residue. Fmoc-AA-OH (5.0 eq. to the resin loading), HATU (4.9 eq.), and *i*-Pr_2NEt (10.0 eq. in NMP, 2.0 M) were pre-incubated in DMF (final concentration = 0.2 M for fmoc-AA-OH) for 1 min and then added to the resin. After 20 min at 45°C under slight shaking, the coupling mixture was removed by suction and the resin was washed with DMF (3 × 1 min).

After completion of the synthesis, the resins were washed with DCM (3x), dried under vacuum and stored at -20 C until the final cleavage of the peptides from the resin.

Then the peptides were cleaved from the resin and deprotected by treating resins with a deprotection/cleavage cocktail (1.0 mL per 25 mg of resin, TFA–*i*-Pr_3SiH–water (95:2.5:2.5, v/v/v)) for 2 h; where **4e** was introduced to the peptide, no *i*-Pr_3SiH was used in the cleavage cocktail. Then, the peptide containing cleavage solutions were collected in and the resins rinsed with neat TFA (1.0 mL per 25 mg of resin). The combined TFA cleavage solutions were concentrated under the stream of nitrogen and dry-ice cooled diethyl ether (10 mL) was added to precipitate the crude peptides. The crude peptides were pelleted by centrifuging (5000x*g*, 5 min) and the diethyl ether was decanted. The crude peptide pellets were further dried under stream of N_2 and purified by prep-HPLC.

2.4.2 Peptide synthesis and characterisation

5 was synthesised according to the procedure provided in section **2.4.1** on resin (10 μmol), using amino acids: Fmoc-Gly-OH and compound **4e.** In case of the use of molecule **4e** was used for a coupling step, 2.5 eq. of *fmoc*- builiding block **4e** was used, while other reagents for this step were also used in ½ quantities, *i.e.*, 2.45 eq. HATU and 5 eq. *i*-Pr_2Net and the reaction was left for 1 h at 45°C under Ar balloon and slight shaking. As a last coupling step, 4-Maleimidobutyric acid (10.0 eq.) was added together with HATU (9.8 eq.) and *i*-Pr_2NEt (20.0 eq.) and shaked at room temperature for 30 minutes. No *i*-Pr_3SiH was used in the cleavage cocktails where **4e** was introduced. The crude peptide was purified by prep-HPLC and lyophilised to yield the product **5** (5.1 mg, 5.1 μmol, 51% yield) in over 95% purity (by UPLC, Figure S-36).

HRMS (*m/z*): calc. for $C_{44}H_{70}N_8O_{12}S_3$, 998.4297; found ESI+ 999.4368 $[M-H]^+$ (diff. 2.0 ppm)

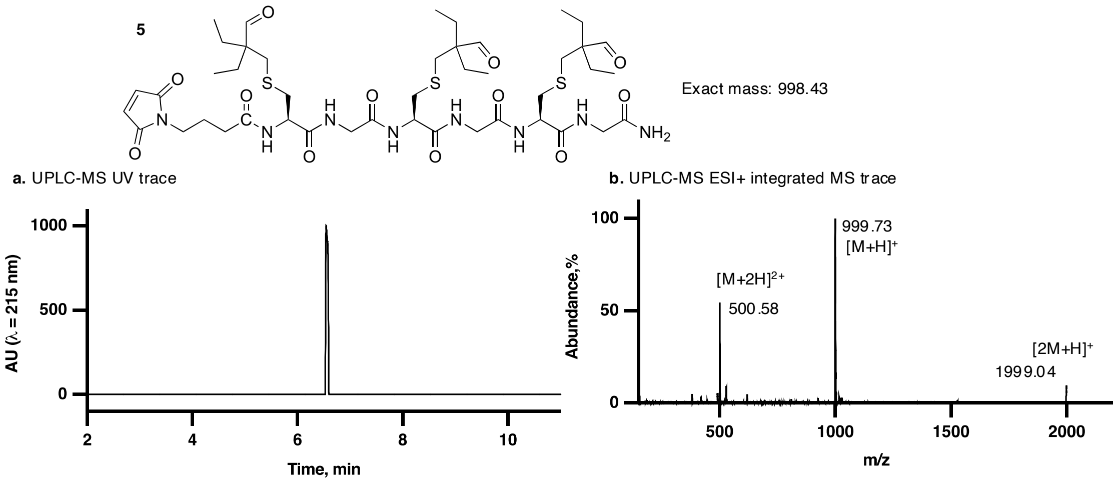

*Figure S- 36 UPLC-MS trace and mass spectrum of purified **5***

7 was synthesised in parallel to **5** according to the procedure provided in section **2.4.1** using Fmoc-Gly-OH, Fmoc-Met-OH and 4-Maleimidobutyric acid (10.0 eq.) as the last coupling step. 3.8 mg, 5.0 μmol, 50% yield) in over 95% purity (by UPLC, Figure S-37).
HRMS (*m/z*): calc. for $C_{29}H_{46}N_8O_9S_3$, 746.2604; found ESI+ 747.2640 $[M-H]^+$ (diff. 1.19 ppm).

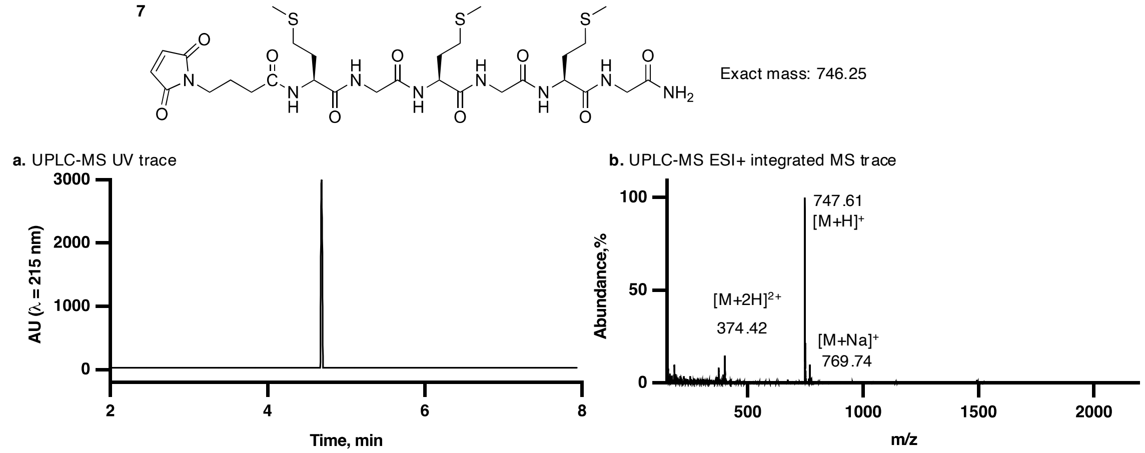

*Figure S- 37 UPLC trace and mass spectrum of purified **7***

SI-17 was synthesised according to the procedure provided in section **2.4.1** on resin (10 μmol), using amino acids: Fmoc-Tyr(*t*-Bu)-OH, Fmoc-Thr(*t*-Bu)-OH, Fmoc-Ala-OH, Fmoc-Trp(Boc)-OH, Fmoc-Cys(Trt)-OH, Fmoc-Lys(Boc)-OH and **4e.** In case of the use of molecule **4e** was used for a coupling step, 2.5 eq. of *fmoc*- builiding block **4e** was used, while other reagents for this step were also used in ½ quantities, *i.e.*, 2.45 eq. HATU and 5 eq. *i*-Pr_2Net and the reaction was left for 1 h at 45°C under Ar balloon and slight shaking. No *i*-Pr_3SiH was used in the cleavage cocktails where **4e** was introduced. The crude peptide was purified by prep-HPLC and further lyophilised to yield the product (5.7 mg, 6.6 μmol, 66% yield) in over 95% purity (by UPLC, Figure S-38).
HRMS (*m/z*): calc. for $C_{42}H_{61}N_9O_9S$, 867.4306; found ESI+ 867.4307 $[M]^+$ (diff. 0.17 ppm).

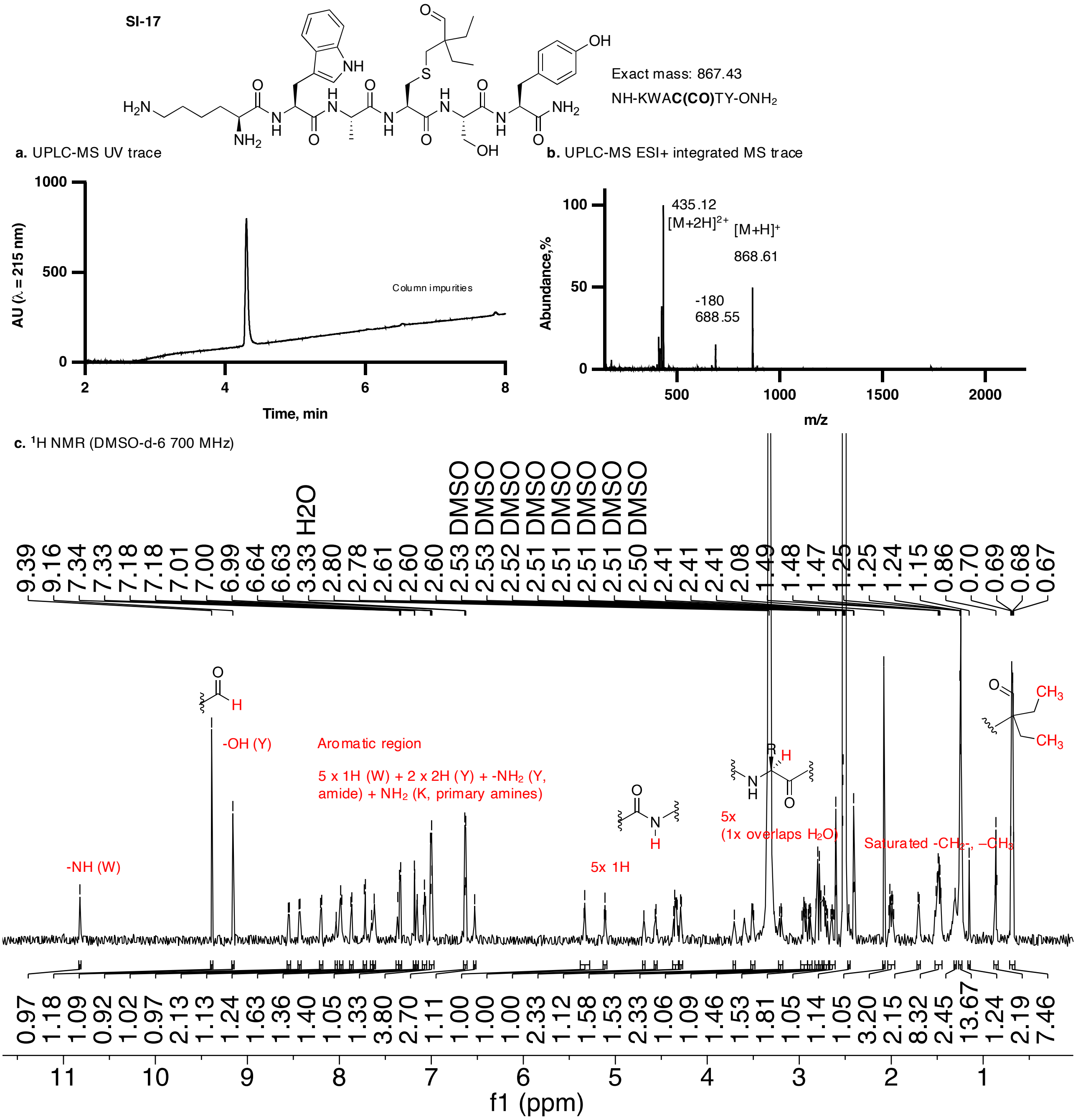

Figure S- 38 UPLC trace and mass spectrum of purified ***SI-17*** *(**a.b.**) and* ***c.*** *low-resolution ^{1}H NMR (700 MHz, DMSO-d6) spectrum. It is difficult to properly assign peptide – ^{1}H NMR spectra without help of 2D spectrum, however, some key properties of the spectrum were observed suggesting intact CO-prodrug moiety present.*

2.5 Verification of CO release vitro, product detection and stability

2.5.1 House-hold CO-meter assay

Two necked-flask equipped with CO meter and magnetic stirrer. All reactive oxygen species were made as described in the literature[2], CO prodrug **4a** or **4b** (2 mM, from 20 mM DMSO solution, 10% DMSO) was added to the flask via syringe which was followed by each freshly prepared ROS solution (100 mM, final concentration 2 mM) and topped up with PBS (pH 7.4) to 1 mL. The CO-monitor (testo 317-3 CO monitor) reading was taken at 30 min time point, each experiment was repeated 3 times. KO_2 was dissolved in degassed DMSO with 1 eq. with 18-crown-6 ether. The Fenton reaction (OH radical) was repeated at fully degassed conditions (freeze-thaw under vacuum, 3 times).

2.5.2 Myoglobin assay

Since the main radical trigger to release carbon monoxide are radicals, which are incompatible with assays like myoglobin (Mb) and it would lead to a fast oxidation of myoglobin to **met-myoglobin**, we have used two compartment myoglobin assay, similar that was described previously by Wang group.[3] As shown in Figure S-2a, in the inner vial equipped with magnetic stirrer is the solution of CO prodrug in MeCN/PBS and ROS, while the bigger (outer) vials contain Mb-deoxy solution and everything is covered by rubber septum. All required stock solutions (Figure S-2b) were prepared and degassed by freeze-thaw on Schlenk line, Myoglobin solution (from equine heart, Sigma Aldrich Cat. M1882) was degassed by bubbling N_2 for 30 min. Then, required volumes of each solution were added sequentially by micro syringe keeping the inert atmosphere and the mixture was stirred for 10 minutes before a sample of myoglobin solution was taken for UV spectroscopy measurement.

2.5.3 CO release in Fenton-like conditions

Stock solutions were prepared as follows and mixed in volumes sequentially as presented in Figure S-39a. The reaction starts at t = 0 is considered the addition of H_2O_2 solution. For a negative controls instead of $MnSO_4$ or H_2O_2 the corresponding volume of MQ water was added. Deuterated trifluoro acetic acid was added to a constant amount when doing replicates. The amount was chosen by measuring the pH in alternative experiment with pH paper to be 8.0 (this would give pD of around 8.4, as pD = pH + 0.4.[4] Reactions were performed in ambient temperature (T = 298K at the day of measurement).

a) For NMR experiment, the reaction was run in open atmosphere fume hood atmosphere and at each time point, 0.7 mL of the reaction mixture was withdrawn and mixed with excess (1 mg) of $Na_2S_2O_3$ and ^1H NMR spectrum was run. The experiment was repeated three times.
b) For CO release experiment, the experiment was repeated in a closed chamber which contained described CO-releasing solution in a beaker that was equipped with a magnetic stirrer, CO-monitor (Figure S-39b). The chamber was placed on a magnetic stirrer and CO-monitor reading was taken at timepoints noted. Experiment was repeated three times.

a.

Addition order	Reagent	[Stock]	Amount	[Final]
1	$NaHCO_3$ in D_2O	800 mM	5.00 mL	400 mM
2	D_2O	-	4.385 mL	-
3	CO-prodrug (i.e. **4a**) in CD_3CN	100 mM	500 μL	5 mM
4	CF_3COOD	neat	100 μL	-
5	$MnSO_4$ in D_2O	250 mM	0.4 μL	1 μM
6	H_2O_2 50%w/w	17.5 M	14.3 μL	25 mM
Quench	$Na_2S_2O_3$ anhydrous	Solid powder	4 mg	25 mM

b.

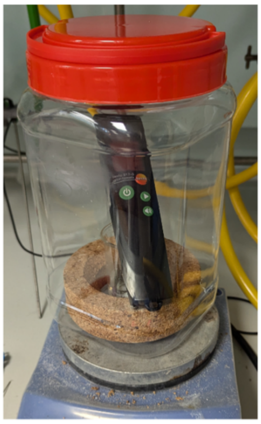

Figure S- 39 Experimental conditions and set up for Mn^{2+} catalysed Fenton-like reaction

2.5.4 CO release in mild oxidising conditions

Stock solutions were prepared as follows and mixed in volumes sequentially as presented in Table S-1. For a negative controls equal volume of MeOH/D was added. Reactions were performed in ambient temperature (T = 298K at the day of measurement). AIBN refers to Azobis(isobutyronitrile). The UV light source was a closed dark chamber equipped with 4 × 9 W bulbs (λ_{max} = 360 nm).

a) For NMR experiment, the reagents in MeOD as per table were added to a directly to NMR tube that was equipped with an evacuation manifold. t = 0 ^{1}H NMR spectrum was run at the beginning of the experiment and then the tube was degassed by three cycles of freeze-thaw under vacuum with last cycle exposing the solution to Argon gas. Then the tube was exposed to UV light for 30 minutes and both ^{1}H NMR and ^{1}H NMR DOSY was run (by the NMR service). Decomposition products of AIBN were assigned by comparison with the previous literature.[5] A sample from this experiment was also used for GC-MS analysi for 2-ethyl-1-butene detection.
b) For UPLC-MS experiment, the reagents as per table in MeOH were added to a 5 mL round bottom flask and then the flask was degassed by three cycles of freeze-thaw under vacuum with last cycle exposing the solution to Argon gas. Then the tube was exposed to UV light for 30 minutes as in a) and UPLC-MS standard method was ran. A sample **SI6-1** was prepared by stirring N-acetylcysteine methyl ester **SI4-2** in MQ-water with 10%DMSO over 24 h and analysed directly.

Table S- 1. CO release in non-oxidising conditions, experimental conditions and set p. AIBN - Azobisisobutyronitrile

Addition order	Reagent	[Stock]	Amount	[Final]
1	CO-prodrug in the solvent	250 mM	100 μL	25 mM
2	AIBN in the solvent	250 mM	100 μL	25 mM
3	Solvent (MeOH or MeOD)	-	800 μL	-
Quench	Exposure to air	-	-	-

2.5.5. Stability assays

Compounds of interest were dissolved in Acetonitrile (10x concentration, usually 10 mM) and then added to a stability media of interest (MQ-water, PBS pH 7.4 (Gibco™ 10010023) DMEM-media (Gibco™ 10566016), human serum (cat. no. Sigma H4522), etc.) to make up for 10% acetonitrile and 1 mM of the compound of interest and then incubated at 37°C for a desired time.

An HPLC standard (N-acetyl L-Tyrosine, CAS 537-55-3) was added to the same concentration to all stability measurements prior the analysis to which peak are was normalised to adjust for the machine injection error. All the experiments were triplicated, and the results are reported as mean ± SD (n = 3). Sample HPLC traces are reported.

Standard HPLC method for stability analysis: instrument: ThermoFisher U3000; column equipped with MSQ+ mass detector, Phenomenex Kinetex C18, 5*μ*m, 50×4.6 mm column, 100 Å; solvent system A = 99.9% H_2O, 0.1% formic acid , B = 99.9% acetonitrile; 0.1% formic acid, flow = 1.20 mL / min; gradient: t = 0–0.50 min 0% B, t = 1–5 min 0–100% B, t = 5–7 min 100% B, t = 7.1-7.8 min 5% B. For mass spectrometry, the capillary voltage of the electrospray source was 3.0 kV with a cone voltage of 75 V and the desolvation gas used was nitrogen, with a flow rate of 800 L h−1.

A. In the case of stability in water, PBS, DMEM the samples were run directly without further downstream refinement using standard HPLC methods.
B. For human serum stability assay, at a given timepoint, equal amount of HPLC-grade acetone (i.e. 100 μL sample plus 100 μL acetone) was added to the sample in an Eppendorf 1.5 mL tube, and the sample was mixed using vortex for 2 minutes. Then, the sample was centrifuged (2 min, 16700x*g*) and then the supernatant was carefully aspirated with an automatic pipette and the solvent was removed under the stream of nitrogen. Then, another portion of HPLC-grade acetone (i.e. 200 μL) was added, and the same procedure was repeated. Lastly, the sample was dissolved in 1:1 mixture water-acetonitrile equal to the initial sample volume (i.e. 100 μL) and the sample was analysed with HPLC.

2.6 Quantum mechanical calculations.

Full geometry optimizations and transition structure (TS) searches were carried out with Gaussian 16[6] using the M06-2X hybrid functional[7] and 6-311+G(2d,p) basis set with ultrafine integration grids. Bulk solvent effects in water were considered implicitly through the IEF-PCM polarizable continuum model.[8] The possibility of different conformations was taken into account for all structures. All stationary points were characterized by a frequency analysis performed at the same level used in the geometry optimizations from which thermal corrections were obtained at 298.15 K. The quasiharmonic approximation reported by Truhlar *et al.* was used to replace the harmonic oscillator approximation for the calculation of the vibrational contribution to enthalpy and entropy.[9] Scaled frequencies were not considered. Mass-weighted intrinsic reaction coordinate (IRC) calculations were carried out using the Hessian-based predictor-corrector integrator scheme by Hratchian and Schlegel[10, 11] in order to ensure that the TSs indeed connected the appropriate reactants and products. Gibbs free energies (ΔG) were used for the discussion on the relative stabilities of the considered structures. The lowest energy conformer for each calculated stationary point (Figures S-16-19) was considered in the discussion; all the computed structures can be obtained from authors upon request. Cartesian coordinates, electronic energies, entropies, enthalpies, Gibbs free energies, and lowest frequencies of the calculated structures are summarized in Supplementary Table S-2.

Table S- 2 Energies, entropies, and lowest frequencies of the lowest energy calculated structures.[a]

Structure	E_{elec} (Hartree)	E_{elec} + ZPE (Hartree)	H (Hartree)	S (cal mol^{-1} K^{-1})	G (Hartree)	Lowest freq. (cm^{-1})	# of imag freq.
**A**	–787.833	–787.604	–787.59	113.7	–787.643	27.6	0
**TS1**	–863.568	–863.331	–863.315	123.6	–863.372	–864.8	1
**B**	–787.182	–786.966	–786.952	118.6	–787.005	11.8	0
**TS2**	–787.164	–786.952	–786.937	119.1	–786.991	–355.6	1
**C**	–673.853	–673.648	–673.636	106.5	–673.685	50.1	0
**TS3**	–673.841	–673.636	–673.624	106	–673.673	–213.6	1
**D**	–235.795	–235.629	–235.621	82.5	–235.66	88.5	0
**A_{OMe}**	–859.64	–859.46	–859.446	112	–859.498	38.6	0
**$TS1_{OMe}$**	–935.375	–935.187	–935.171	121.2	–935.228	–821.6	1
**B_{OMe}**	–858.99	–858.822	–858.808	113.1	–858.861	47.7	0
**$TS2_{OMe}$**	–858.983	–858.817	–858.803	116.9	–858.856	–187.1	1
**C_{OMe}**	–745.669	–745.51	–745.499	104.9	–745.547	25.3	0
**$TS3_{OMe}$**	–745.662	–745.504	–745.492	103.7	–745.54	–271.8	1
**D_{OMe}**	–307.615	–307.496	–307.488	78.9	–307.526	100.7	0
**HO·**	–75.7325	–75.724	–75.7207	42.6	–75.7409	3765.8	0
**H_2O**	–76.4297	–76.4082	–76.4044	45.1	–76.4258	1617.3	0
**CO**	–113.313	–113.308	–113.305	47.2	–113.327	2272.3	0
**MeS·**	–438.041	–438.005	–438.001	59.9	–438.029	526.3	0
**A'**	–863.026963	–862.794364	–862.779711	114.4	–862.833196	50.6	0
**TS1'**	–938.767632	–938.526530	–938.510195	124.4	–938.567788	–995.1	1
**B'**	–862.376425	–862.156133	–862.141535	115.2	–862.195718	68.2	0
**TS2'**	–862.357887	–862.141582	–862.126234	121.1	–862.181879	–374.1	1
**C'**	–749.048954	–748.840035	–748.826780	111.3	–748.877802	39.1	0
**TS3'**	–749.044456	–748.836785	–748.823709	111.2	–748.874471	–449.4	1
**A''**	–938.252917	–938.014595	–937.999402	116.2	–938.054022	56.2	0
**TS1''**	–1013.990038	–1013.743360	–1013.726223	127.7	–1013.785418	–789.7	1
**B''**	–937.602482	–937.376776	–937.361500	118.5	–937.416938	46.7	0
**TS2''**	–937.585326	–937.363358	–937.347408	125.7	–937.404148	–387.9	1
**C''**	–824.276771	–824.062339	–824.048424	114.0	–824.100985	42.1	0
**TS3''**	–824.261545	–824.049387	–824.035234	117.6	–824.088458	–288.4	1
**MeOS·**	–513.266023	–513.225590	–513.220503	67.4	–513.252515	133.4	0
**MeO_2S·**	–588.465985	–588.420450	–588.414802	71.1	–588.448588	184.0	0
**Sec α-S rad**	–787.177187	–786.962710	–786.948401	115.1	–787.001840	41.9	0
**Prim α-S rad**	–787.171984	–786.957948	–786.943579	116.0	–786.997048	30.5	0

[a]Energy values calculated at the PCM(H_2O)/M06-2X/6-311+G(2d,p) level. 1 Hartree = 627.51 kcal mol^{-1}. Thermal corrections at 298.15 K

2.7 Bioconjugation reactions to IgG (immunoglobulin G) antibodies

All buffers for bioconjugation were prepared on site. TCEP was prepared in MQ-water and the pH adjusted as required. Trastuzumab was obtained from MedChemExpress (HY-P9907) and reconstituted in MQ-water to the concentration of 7.2 mg mL^{-1}

The concertation of proteins or their conjugates was measured using Nanodrop™One microvolume UV spectrometer and the concentration was calculated using extinction coefficient $\varepsilon_{(trastuzumab)}$= 225000 M^{-1} cm^{-1}.[12]

To analyse the IgG antibodies by LC–MS, samples had to be fully reduced into heavy chain (HC) and light chain (LC). 1 μL of the reaction mixture was diluted with 8 μL of PBS and DTT (1 μL, 100 mM stock) was added, and the sample was kept 37°C for 30 min.

Similarly, to analyse IgG antibodies by SDS-PAGE, samples were diluted into PBS to around 2 μM reaching 9 μL volume and DTT (1 μL, 100 mM stock) or PBS (1 μL, 100 mM stock) was added for reduced and non-reduced conditions, respectively, before adding stock of LDS (4x, 3.3 μL) and heating the sample to 95°C for 5 minutes.

2.7.1 **BC1** - Trastuzumab CO conjugate

To 100 μL of Trastuzumab in PBS (49.5 μM, 7.2 mg/mL), TCEP (5 eq., 2.5 μL, 10 mM in water) was added and the solution was incubated at 37°C for 30 min. Then desired maleimide **5** (or **7** for negative control) stock solution (12 eq. (1.5 eq. per cysteine), 7.9 μL, 10 mM in DMF) was added and the mixture was kept 25°C while reaction progress was monitored by reducing LC-MS. After the reaction completion, excess of maleimide was removed by UF/DF (Amicon^R 3 kDa MWCO spin filter) buffer exchange to PBS (pH 7.4) three times, followed by a further desalting twice into PBS (pH 7.4) using 7 kDa MWCO Zeba™ spin desalting columns to yield stock solution in PBS (7.0 μM, 300 μL, 42% yield for BC1).

2.7.2 **BC3** - Trastuzumab LC-V205C- Alexa Fluor 488

Trastuzumab LC-V205C[13] modification with Alexa Fluor 488 maleimide was done according to the literature procedures.[14] Briefly, Trastuzumab LC-V205C in storage buffer (42.7 μL, 11.7 mg/mL) and Tris-HCl buffer (1 M, pH 8.0, 3.3 μL) was added to give a final Tris-HCl concentration of 75 mM. Then, Alexa Fluor 488 C5 maleimide (Invitrogen, catalogue number: A10254, 3 eq., 2 μL, 5 mM stock in DMF) was added with additional DMF (3 μL, 10% final). The reaction was incubated at 30 min at room temperature, after which the reaction progress was assessed by reducing LC–MS analysis and shown the reaction completion. Free Alexa Fluor 488 was removed by UF/DF (Amicon^R 3 kDa MWCO spin filter) buffer exchange to PBS (pH 7.4) five times, followed by a further desalting twice into PBS (pH 7.4) using 7 kDa MWCO Zeba™ spin desalting columns to yield stock solution in PBS (24.8 μM, 150 μL, 93% yield).

2.7.3 Bioconjugate stability

Bioconjugate **BC1** was diluted to the final concentration of 5 μM and then the solution was incubated at 37°C for a desired time. Then the sample was reduced with DTT using regular methods and the LC-MS was run.

2.7.4 Biolayer interferometry (BLI)

BLI analyses were performed using an Octet-BLI K2 instrument (ForteBio). Assays were carried out at a constant temperature of 25°C. Consumables used: 96 streptavidin coated biosensors from Sartorius (Cat. 18-5019) and black flat-bottomed 96-well plates. For all assays, two biosensors (sample and reference) were used for each concentration analysed to control for non-specific binding of the IgGs to the sensor surface, the reference sensor with no antigen loaded was run in parallel and subtracted at each IgG concentration. A sensor loaded with antigen and the association step containing only assay buffer was subtracted from all concentrations to account for any drift observed from loaded sensors in the buffer. Analysis of the data were carried out to derive the k_a, k_d and K_D values for BC1 vs trastuzumab using a kinetic association then dissociation model. KDs determined for are apparent values since IgGs are multivalent binders and avidity effect takes place.

Assay buffer (10 mM HEPES, 150 mM NaCl, 3.0 mM EDTA, 0.05% Tween-20, 10 mM BSA) and glycine buffer (10 mM, pH 2) were made on site.

Briefly, Octet SA Biosensors (Sartorius) were pre-hydrated during 3 x cycles of regeneration 5 s in glycine buffer followed by 5 s of neutralisation in assay buffer, followed by 60 s wash in assay buffer, 150 s of loading using 1 ng mL^{-1} biotinylated Human HER2 receptor (ACROBiosystems, Cat. HE2-H82E2) for the sample sensor and buffer for the reference sensor, which was followed by 60 s wash in the assay buffer, then 60 s baseline in the buffer, 200 s association in 50 nM, 10 nM, 3.3 nM, 1.1 nM, 0.37 nM of BC1 and 50 nM trastuzumab itself, lastly following by 350 s dissociation in the assay buffer.

2.8 Cell experiments.

General conditions. Cells (HeLa, RAW264.7, SKBR3, MCF7, HEK293T) were acquired from ATCC unless otherwise stated and were grown in a humidified incubator at 37 °C under 5% CO_2 with 90% humidity and split at approximately 80% confluence using Gibco™ TrypLE™ Express (cat. no 12604013) in order to keep them in the exponential growth phase.
All cell cultures were grown in high glucose Gibco™ DMEM (+ pyruvate, GlutaMax™, cat. no 61965026) supplemented with 10% heat inactivated FBS (F9665, Sigma-Aldrich). Cells were counted using Countess™ 2 automated cell counter using Cell Counting Chamber Slides Invitrogen™ (C10315) and staining dead cells with trypan blue. Each experiment was repeated three times, in case of viability assays, each condition had three technical replicates.

2.8.1 Cell viability assays

Cells were seeded in Corning Costar 96-well clear flat-bottom plates at 5,000 (10,000 for RAW264.7) cells per well in 100 μL of media on day before the experiment. The following day, compounds of interest were diluted to 4x of desired concentrations in 50 μL and added to the seeded cells, following by 50 μL of media. Cells were incubated for 48 h hours before the viability reagent was added (CellTiter-Blue® Cell Viability Assay G8080) and the fluorescence readout was measured after another 2 hours incubation at 37 °C (λ_{ex} = 540 nm, λ_{em} = 590 nm) with Molecular Devices SpectraMax MiniMax 300 Imaging cytometer i3x. Cell viability% was calculated as 100% x F_{cells} / $F_{vehicle\_control}$. F refers to the fluorescence of a given well. %0 cell viability refers to control of 100 μM digitonin. Viability assays from dilution series using doxorubicin (8-0.1 μM) was run in parallel to ensure metabolism and growth in cells are not inhibited by external factors and viability assays of non-toxic compounds are valid.

2.8.2 LPS stimulation of RAW264.7 cells and nitrite quantification by Greiss assay

The assay has been done similarly as reported by Seixas *et al.*[15] Murine macrophages RAW264.7 cells were seeded in 96-well plate 40,000 cells/well in 100 μL and allowed to attach for 7 hours. Phenol-red free complete media (Gibco™ DMEM cat. no. 31053028 supplemented with Glutamax™ cat. no 35050061 and Pyruvate 11360070) was used for this experiment. Then, the cells were treated with Lipopolysaccharide (LPS, Invitrogen eBioscience Lipopolysaccharide (LPS) Solution, (2.5 mg/mL)) to the final concentration of 0.5 μg/mL or vehicle control in 50 μL of the complete media. Then, another 50 μL were added containing compounds of interest of vehicle control which also were pre-diluted in the complete medium to the desired concentration. LPS-induced media only wells used for control. Cells were incubated for 16 hours, after which 200 μL of Greiss reagent (Sigma Aldrich, cat. no. G4410, prepared according to the manufacturer instructions) and the absorbance reading was taken at 540 nm after 15 minutes.
Dilution series of $NaNO_2$ in the complete media same conditions was used to obtain a standard curve for [NO] for each experiment. 100% of [NO] refers to LPS-treated control only since vehicle DMSO also raise some anti-inflammatory effects.

2.8.3 LPS stimulation of RAW264.7 cells and TNF-α quantification by Western blot

Murine macrophages RAW264.7 cells were seeded in 6-well plate 300,000 cells/well in 1 mL and allowed to attach for 6 hours. Then, the cells were treated with Lipopolysaccharide (LPS, Invitrogen eBioscience Lipopolysaccharide (LPS) Solution, (2.5 mg/mL)) to the final concentration of 0.1 μg/mL together with compounds of interest in PBS (10 mM stock solution) to the final concentration of 200

uM, or a vehicle control. LPS-untreated control was also included. Cells were incubated for 16 hours. The supernatant was discarded, and the cells were washed twice with warm PBS (2 mL). N-PER Neuronal Protein Extraction Reagent (Thermo Scientific 87792) supplemented with Halt Protease Inhibitor (Thermo Scientific 78429) (100 μL) was added directly to each well before the cells were scraped and transferred to the respective Eppendorf tube. The lysate was centrifuged (4°C, 10000g, 10 mins), and the supernatant was transferred to a fresh Eppendorf tube. The protein concentration was determined using the Pierce™ BCA Protein Assay mentioned below. Subsequently, the samples (25 μg of protein) were denatured with NuPAGE LDS sample buffer supplemented with DTT (25 mM) at 95°C for 5 mins before they were run on Invitrogen NuPAGE Bis-Tris protein gel. The separated protein bands were then transferred to a PVDF membrane using the iBlot 2 Gel Transfer Device. The membrane was incubated in blocking buffer (1x TBS, 5%w/v skimmed milk, 0.05% Tween 20) for an hour before it was probed with the respective primary antibodies (anti-TNF-α: Abcam 183218, 1:1000 dilution; anti-iNOS: Abcam 178945, 1:1000 dilution; anti-β-actin: Cell Signaling Technology 13E5, 1:1000 dilution) at 4°C overnight. The membrane was then washed twice with phosphate-buffered saline with Tween-20 detergent buffer (PBS-T), and further probed with HRP-conjugated secondary antibody (anti-rabbit IgG, Cell Signaling Technology, 1:1000 dilution) for an hour at room temperature. The membrane was washed twice with PBS-T before the ECL reagent (Pierce 32106) was added. The protein bands were imaged for chemiluminescence with Bio-Rad ChemiDoc MP.

Images were quantified by ImageJ using gel analysis tool. TNF-α/β-actin ratio was used to determine the change in overall TNF-α expression, normalising to minimum (untreated control) and maximum values (LPS-induced vehicle). Merged/gels with ladder are presented in section 5 (Uncropped gels).

Protein quantification in cell lysates

Pierce 660-nm protein assay reagent (150 μL, Thermo Scientific 22662) was added to each well in a 96 well plate. The standard and samples of BSA (10 μL, 125 μg - 2000 μg, Thermo Scientific 23208) were subsequently added to respective wells and incubated for 5 mins. The protein concentration was acquired using the SpectraMax MiniMax 300 Imaging Cytometer for its absorbance at 660nm and a standard curve was plotted with GraphPad Prism.

2.8.4 Cell imaging

General. Cells of interest were routinely passaged according to the recommendations and seeded one day before the experiment at a density of 120,000 cells/well, on 13 mm round cover slips (# 1.5, 0.16–0.19 mm) that were placed in 24-well plates and coated with poly-D lysine (Gibco™ cat. no A3890401, 30 min coating, washing with PBS (x3) and air drying).

Fixed cells were then imaged using a Leica DMi8 confocal microscope with a 40× oil immersion objective. Live cells were imaged with the same microscope equipped OKOlab system for microscopy with 37 ºC temperature control and 5% CO_2 supply . For each sample, at least 3 images were taken at randomly assigned fields, at least three independent biological replicates were performed in each experiment unless stated otherwise. For visualisation and analysis, raw images were imported using "default" colour mode into Fiji ImageJ2 (version 2.14.0/1.54f). The fluorescence per cell were measured by creating binary image from cell membrane or CO-probe stain and selecting regions of interest (ROI) above 100 μm^2 and then using *measure* function.

2.8.3.1 Cell experiments (fixed sample) for imaging exogenous CO using 1-Ac probe

For CO release in cells experiment 1-Ac probe was synthesised according to the instructions in previous literature,[16] and 10 mM (2000x) solution in DMSO was made. Before the experiment, the stock solution was diluted into DMSO 10-fold (200x) and then filtered through sterile filter (0.22 μm). Before imaging, chosen cells (HeLa, SKBR3 or MCF7) were seeded and used when they have reached around 70% confluence, usually following day. On the day of the experiment, cells that were about 70% confluent, were washed with PBS (pH 7.4) once and 1-Ac solution (5 μM, 0.5% DMSO) was added in fresh serum-free DMEM media for 30 min incubation. Then, cells were washed twice and treated with a required concentration of CO-prodrug or a vehicle control and incubated 30 minutes at 50 μM for small molecules like **4a** or **4b**, and 2 hours for bioconjugates **BC1** or **BC2** (in PBS). 100 μM of a positive control CORM3[17] was used to measure maximum possible turn-on fluorescence by the probe so laser intensity could be regulated accordingly. Lastly, cells were rinsed with PBS (3x) and prepared for imaging were fixed with a 4% paraformaldehyde solution in PBS (300 μL / well) for 20 min, protected from light. The cover slips then were mounted onto glass slide with Mounting Medium with DAPI (abcam Fluoroshield cat. no AB104139). Images were acquired using channels: nucleus (λ_{ex} 405 nm, λ_{em} 460-490 nm) and CO-probe fluorescence (λ_{ex} 561 nm, λ_{em} 570-620 nm).

For *1-Ac* mean fluorescence per cell quantification, the binary image was created from 561 nm channel. For comparative purposes and pictorial analysis, the maximum colour for CO-channel was manually set to a value of 100 for every image using the colour balance tool, while nuclei channel was adjusted automatically.

N.B. For experiment where HER2 receptors were blocked with Trastuzumab first, cells were firstly incubated with Trastuzumab (100 nM, 250 μL in complete media, 30 min), then washed 3 times with PBS and the experiment was proceeded as usual.

2.8.3.2 Live cell experiments for imaging exogenous CO using COP-1

COP-1 was synthesised according to the literature and 5 mM (5000x) solution in DMSO was made.[18] Before the experiment, the stock solution was diluted into DMSO 50-fold (100x final stock) and then filtered through sterile filter (0.22 μm).

Briefly, one day before the experiment, murine Macrophages Raw264.7 were seeded in 35 mm dishes (Ibidi μ-dish cat. no 81156). On the day of the experiment, cells that were about 70% confluent, were washed with PBS (pH 7.4) once and 500 μL DMEM medium (phenol red-free, serum free) was added that contained LPS or vehicle control (0.5 μg/mL final) and the macrophages were incubated for 30 minutes. Then, 500 μL DMEM (phenol red-free, serum-free) solution containing COP-1 (final 5 μM, 0.5% DMSO), Hoechst 33342 (final 0.1 μg/mL, Invitrogen, 10 mg/mL solution in water, cat. no H3570) and LPS (adjusted to 0.5 μg/mL final) were added and cells were incubated for another 30 minutes. Then, cells were washed twice and treated with a required concentration of CO-prodrug or a vehicle control (30 minutes at 50 μM) and imaged live directly. Images were acquired using channels: nucleus (λ_{ex} 405 nm, λ_{em} 460-490 nm) and CO-probe 1 fluorescence (λ_{ex} 488 nm, λ_{em} 500-650 nm).

For *COP-1* mean fluorescence per cell quantification, the binary image was created from 488 nm channel. For comparative purposes and pictorial analysis, the maximum colour for CO-channel was manually set to a value of 30 for every image using the colour balance tool, while nuclei channel was adjusted automatically.

2.8.3.3 HER2 receptor saturation experiment

Briefly, all the conditions were kept as similarly as possible as in **2.8.3.1**. SKBR3 and MCF-7 cells were seeded day before the experiment. On the second day, the cells were washed with PBS (pH 7.4) once and the BC3 (HER2 antibody Trastuzumab V205C – AlexaFluor 488 nm conujagate) in PBS was added (250 μL/well) in desired concentrations. After 2 h or 4 h of incubation, the cells were fixed with a 4% paraformaldehyde solution in PBS (300 μL / well) for 20 min, protected from light. Then, the cell walls were stained by treating with Wheat Germ Agglutinin – Alexa Fluor 647 conjugate (Invitrogen™ catalogue number W32466) according to manufacturer instructions and washed 3 times. The cover slips then were mounted onto glass slide with Mounting Medium with DAPI. Imaging was done using standard DAPI, Alexa Fluor 488, Alexa Fluor 647 filter cubes. For comparative purposes and pictorial analysis, the maximum colour for CO-channel was manually set to a value of 30 for every image using the colour balance tool, while nuclei channel was adjusted automatically.

2.8.5 Flow cytometry

BC1 binding to the receptor using secondary antibody control

Cells were routinely cultured in complete DMEM medium, and split every other day when the cells reached 80% confluency in order to keep them in the exponential growth phase. On the day of the experiment cells were harvested for staining of the HER2 receptor. Cells were resuspended to concentration of mln mL^{-1} in Flow cytometry buffer (Invitrogen™ eBioscience™ cat. no 00-4222-26) to eliminate non-specific binding and kept on ice for 1 hour. Then, cells were then stained with 100 nM of **BC1** or **trastuzumab** control for 1 h at 4 °C. The non-specifically bound primary antibody (**BC1** or **trastuzumab**) was removed by repeated washing in PBS (3x) and a secondary goat anti-human IgG-Alexa 647 (Invitrogen A21445) was added for another 30 min at 4 °C in 1:200 dilution. Non stained control and secondary antibody-only controls were also included. Cells were washed three times, resuspended in Flow cytometry buffer and filtered through cell strainer (40 μM) to remove cell clusters. Samples were analysed by flow cytometer straight away counting at least 40,000 events per sample. The samples were acquired using CytoFLEX (Beckman Coulter) using routine methods. The data was analysed by FlowJo software and only single cell data is shown. Washing refers to centrifugation of the sample at 200g, 5 min, at 4°C.

BC3 surface binding (primary antibody binding)

Cells were routinely cultured in complete DMEM medium, and split every other day when the cells reached 80% confluency in order to keep them in the exponential growth phase. On the day of the experiment, cells were harvested and collected for surface receptor staining, washed with PBS (pH 7.4. 3 x), fixed in 2% PFA buffer for 20 min on ice, washed (3 x), and resuspended to a concentration on 1 mln mL^{-1} in Flow cytometry buffer (Invitrogen™ eBioscience™ cat. no 00-4222-26). Then, sample of cells was divided into two parts (Control and Membrane receptor staining). Cells were treated with **BC3** (100 nM) for 30 min on ice or vehicle control in flow cytometry buffer (Invitrogen™ eBioscience™ Flow Cytometry Staining Buffer 00-4222-26) to allow for surface receptor binding, washed (PBS, 3 x) and resuspended in flow cytometry buffer. Samples were analysed by flow cytometer straight away counting at least 40,000 events per sample. The samples were acquired using CytoFLEX (Beckman Coulter) using routine methods. The data was analysed by FlowJo software and only single cell data is shown. Washing refers to centrifugation of the sample at 200g, 5 min, at 4°C.

2. 9 Animal studies

8- to 12-weeks old C57BL/6 mice were obtained from Charles River. On day 0, 1×10^6 MC-38 cells (Kerafast) were injected subcutaneously (s.c.) into the flanks of C57BL/6 mice with 100 µL of a 1:1 mixture of Dulbecco's modified Eagle medium (Gibco) with Matrigel (Corning). 10 mg/Kg of **4a** were administered weekly, once tumor volumes reached between 100-200 mm^3, by intratumoral (i.t.) injection in 50 µL PBS (Gibco). Equimolar doses of N-acetylcysteine (**NAC**) (Sigma) in PBS and the same volume of PBS were also administered as control treatments. Mice were euthanized when tumor volume reached 1 cm^3. All animals were maintained according to protocols approved by the Direção Geral de Veterinária and GIMM Lisboa ethical committee.

Tumour and weight measurement

Tumors were inoculated as aforementioned. Once palpable, they were measured with a caliper and volume was calculated as $\boldsymbol{V} = \frac{(\boldsymbol{length} \times \boldsymbol{width^2})}{\mathbf{2}}$. Toxicity was assessed by monitoring mice weight every day using a scale.

Statistical analysis

Statistical analysis was performed using GraphPad Prism 8. Tumor growth values and weight variation were analyzed by two-way ANOVA (95% confidence interval). Comparisons of the survival of tumour-bearing mice were performed using the log-rank Mantel–Cox test (95% confidence interval). Two-tailed student t-tests were used to assess significance in the remaining data. For statistical tests, a P value of less than 0.05 considered significant.

3 ^{1}H and ^{13}C NMR spectra

3.1 Spectra of the main molecules

2 ^{1}H NMR (500 MHz, $CDCl_3$) and ^{13}C NMR (126 MHz, $CDCl_3$)

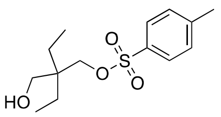

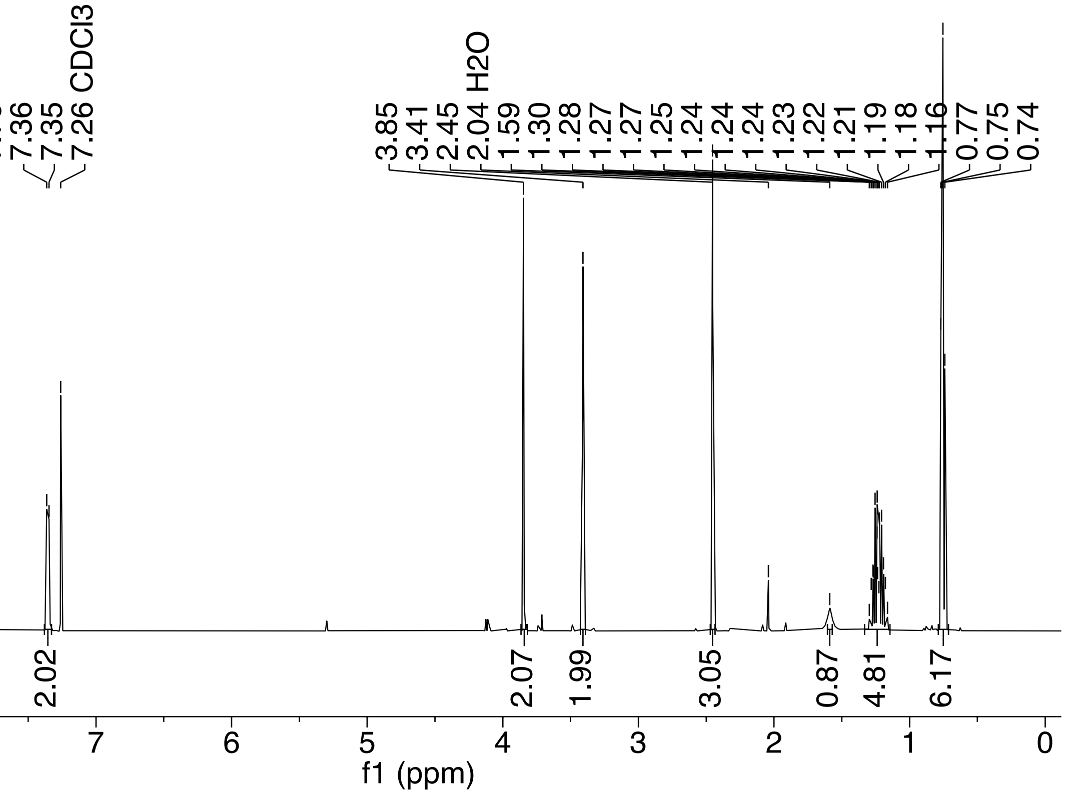

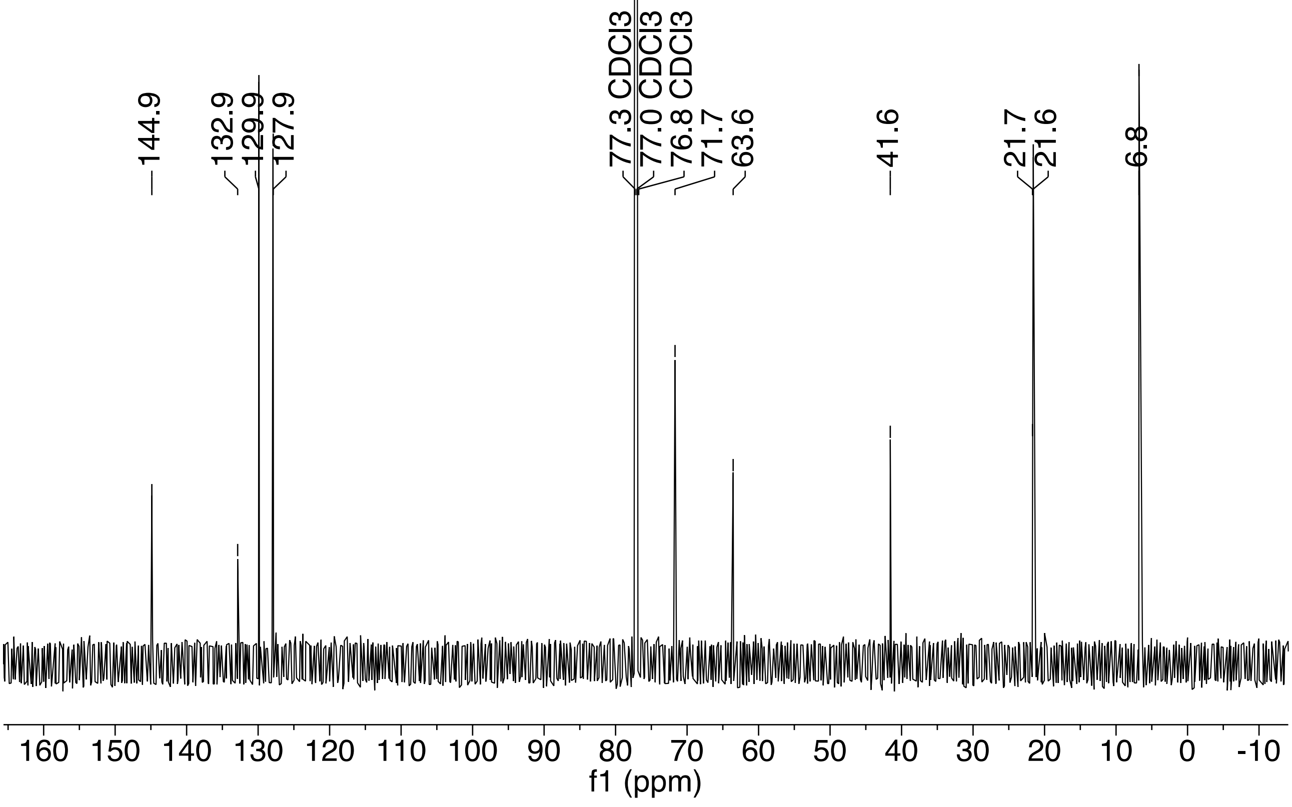

3 ^{1}H NMR (500 MHz, $CDCl_3$) and ^{13}C NMR (126 MHz, $CDCl_3$)

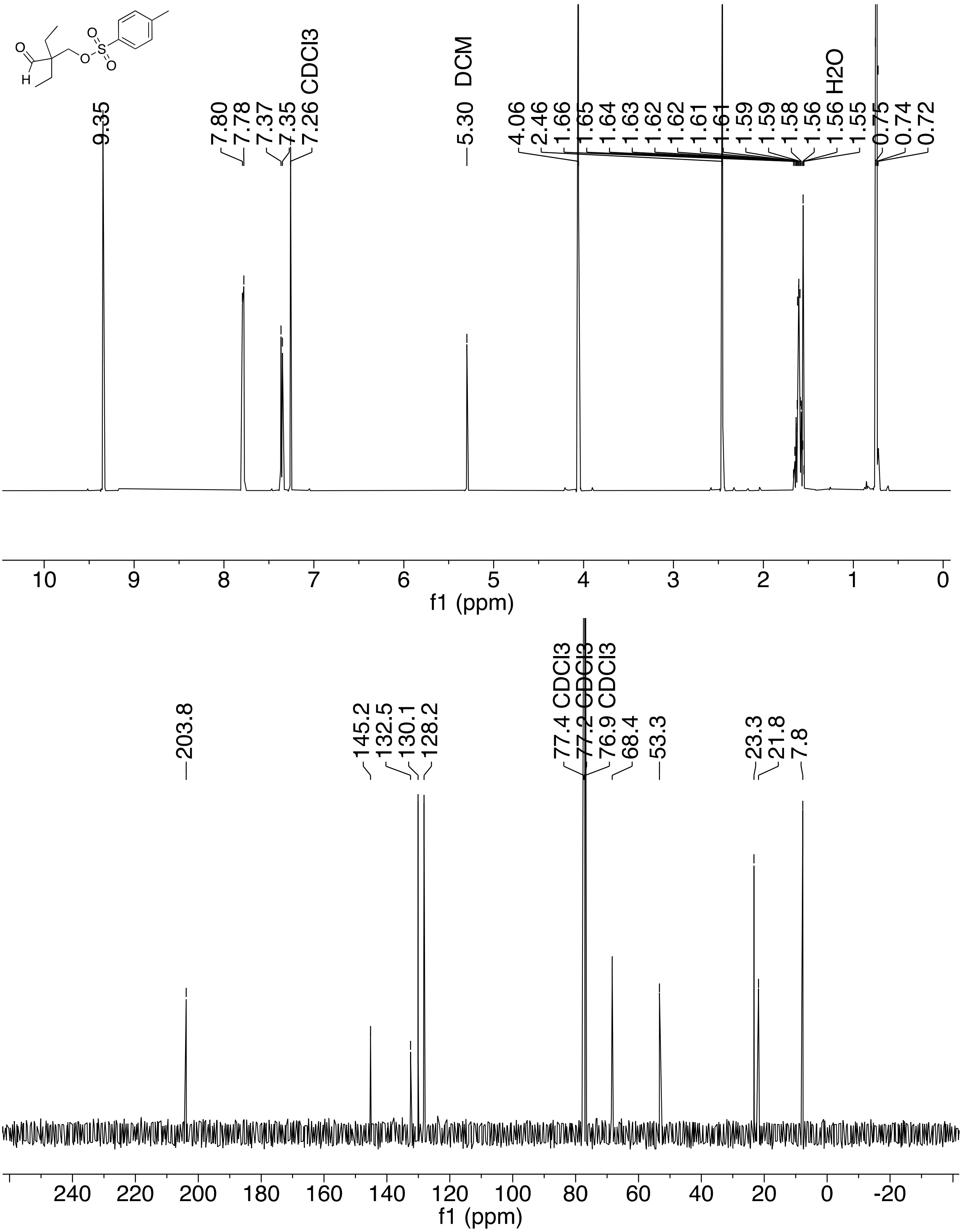

4a ^{1}H NMR (400 MHz, $CDCl_3$) and ^{13}C NMR (101 MHz, $CDCl_3$)

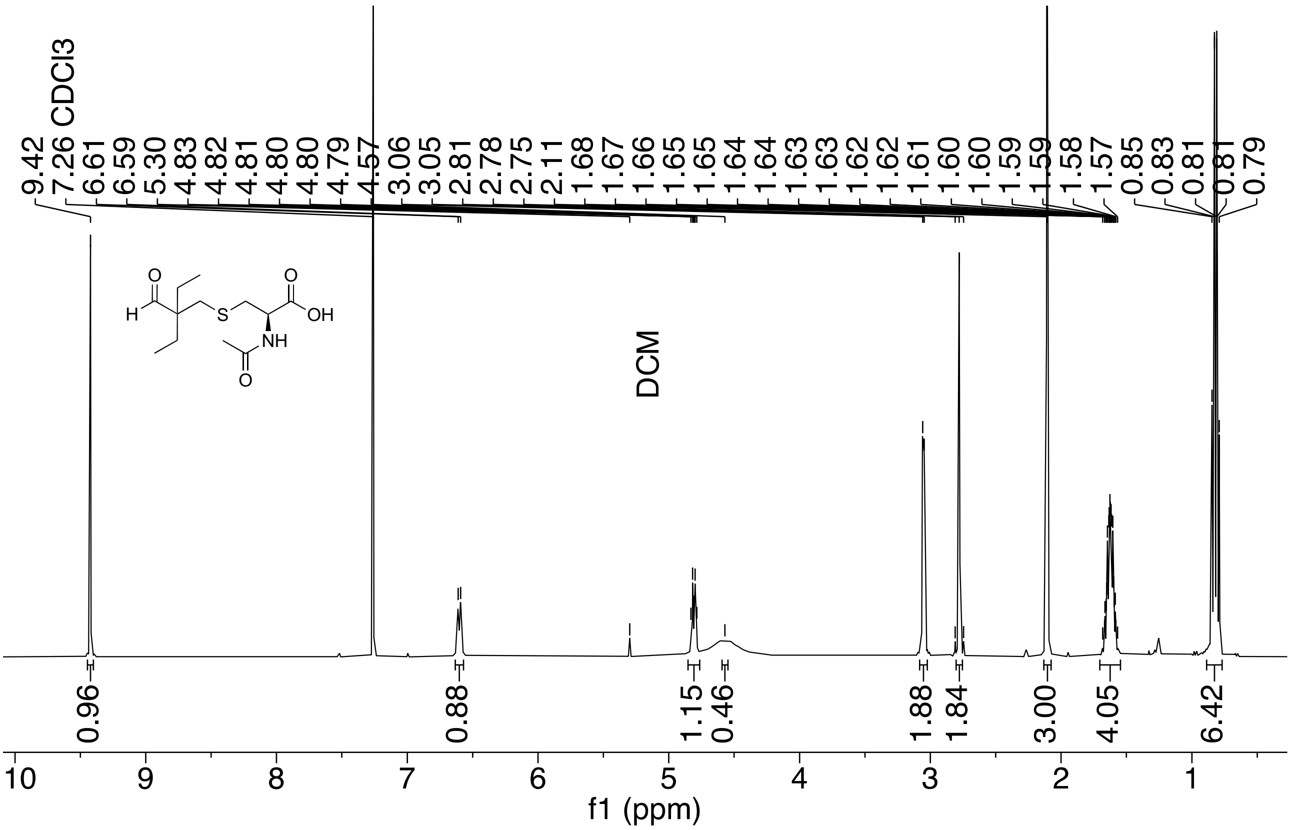

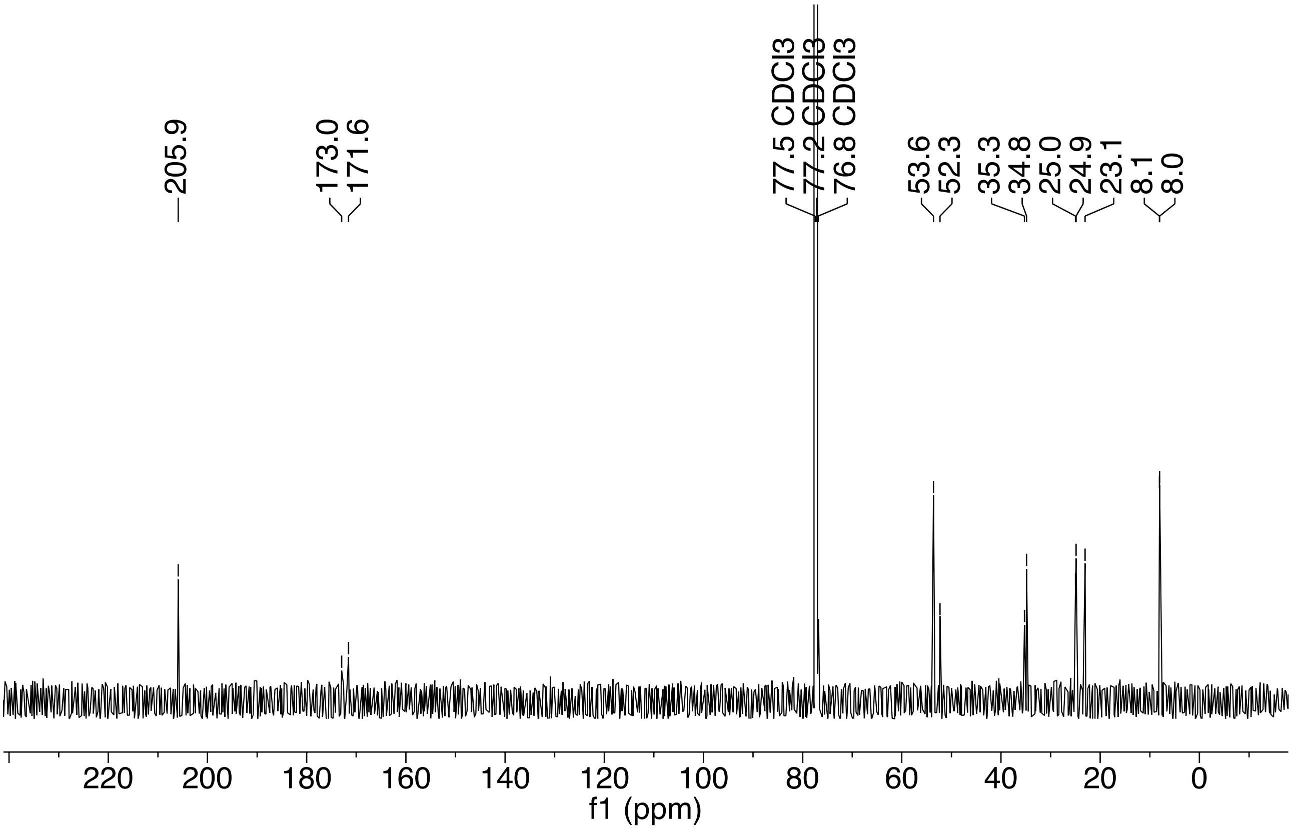

4b ^{1}H NMR (400 MHz, $CDCl_3$) and ^{13}C NMR (101 MHz, $CDCl_3$)

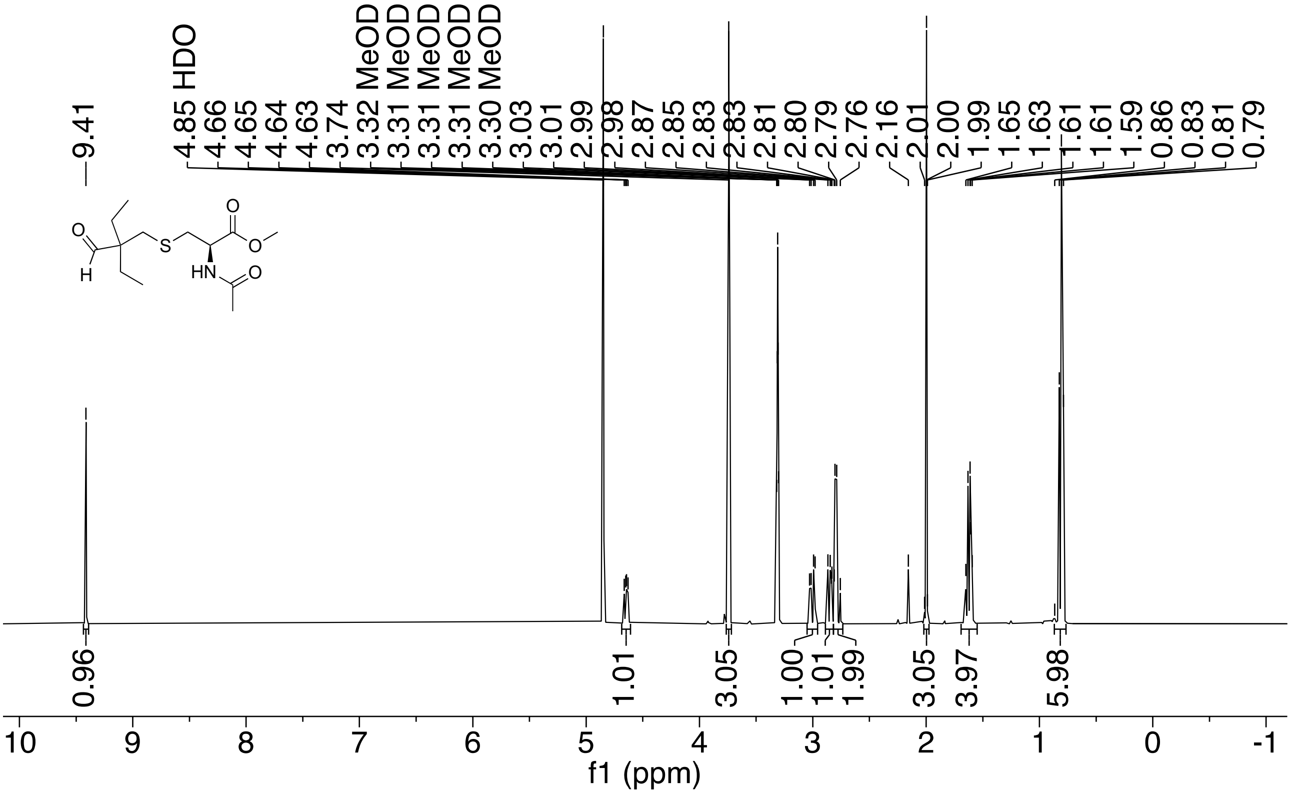

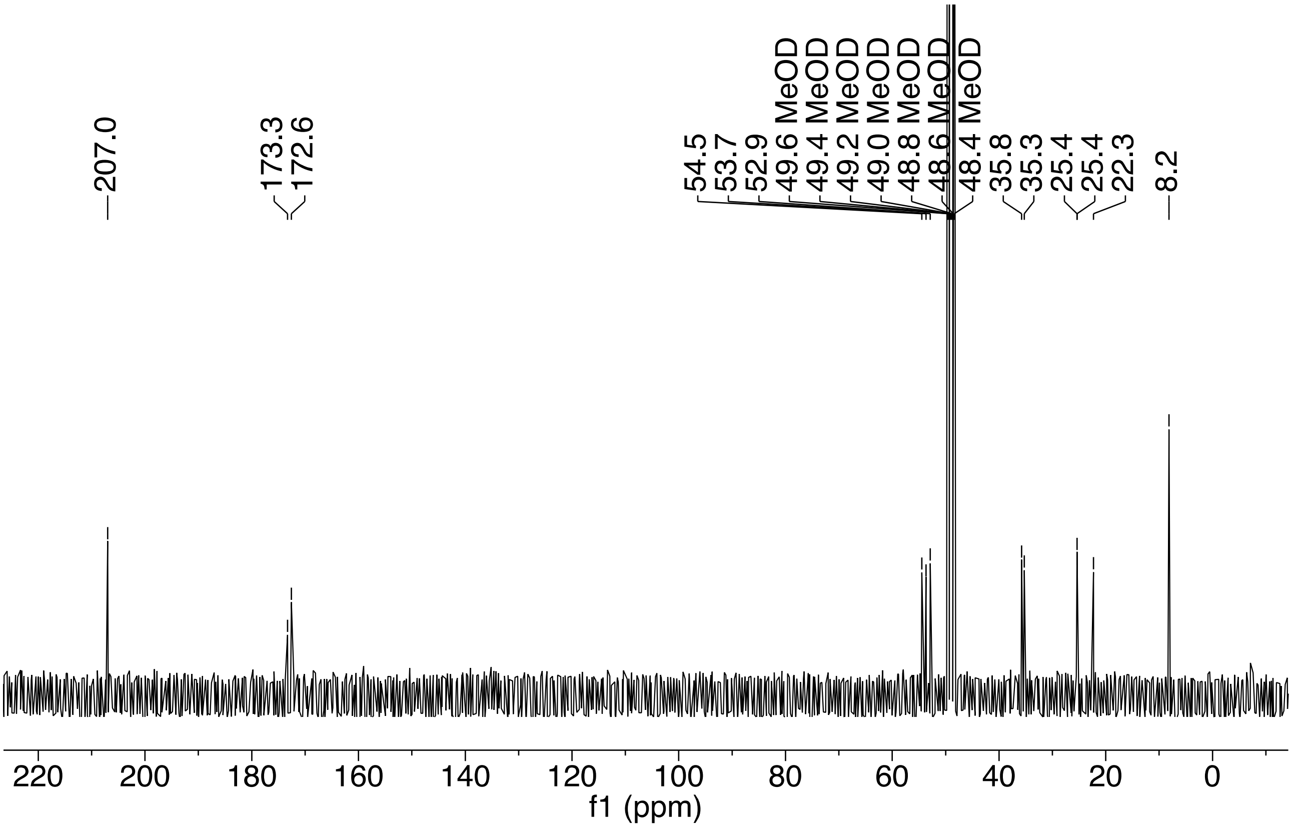

4c ¹H NMR (400 MHz, Acetone-d6) and ¹³C NMR (101 MHz, Acetone-d6)

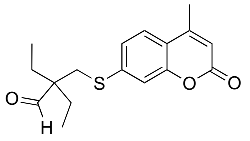

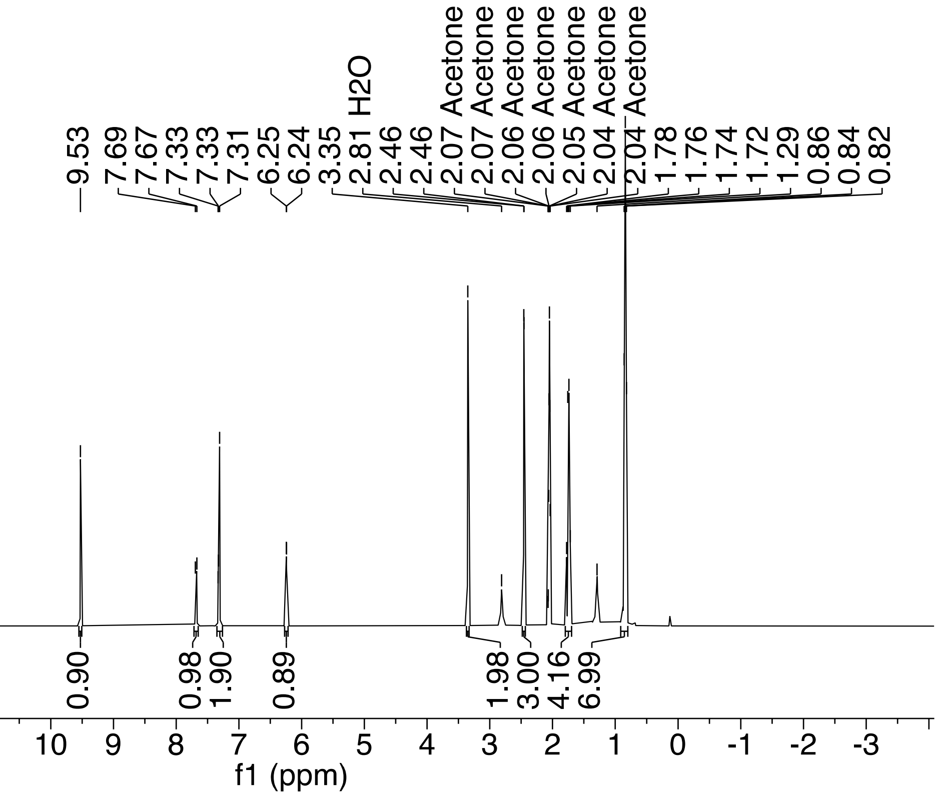

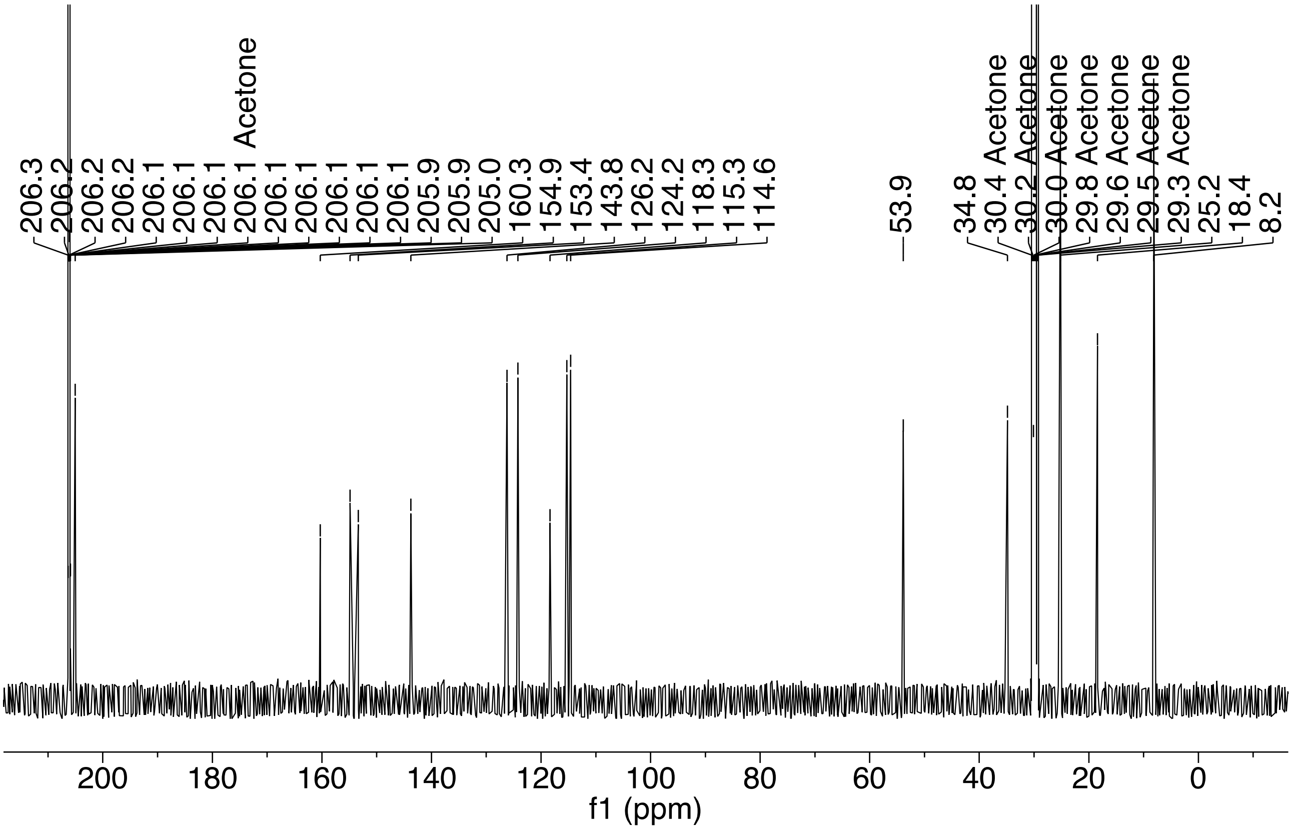

4d ^{1}H NMR (400 MHz, $CDCl_3$) and ^{1}H NMR (400 MHz, Acetone-d6)

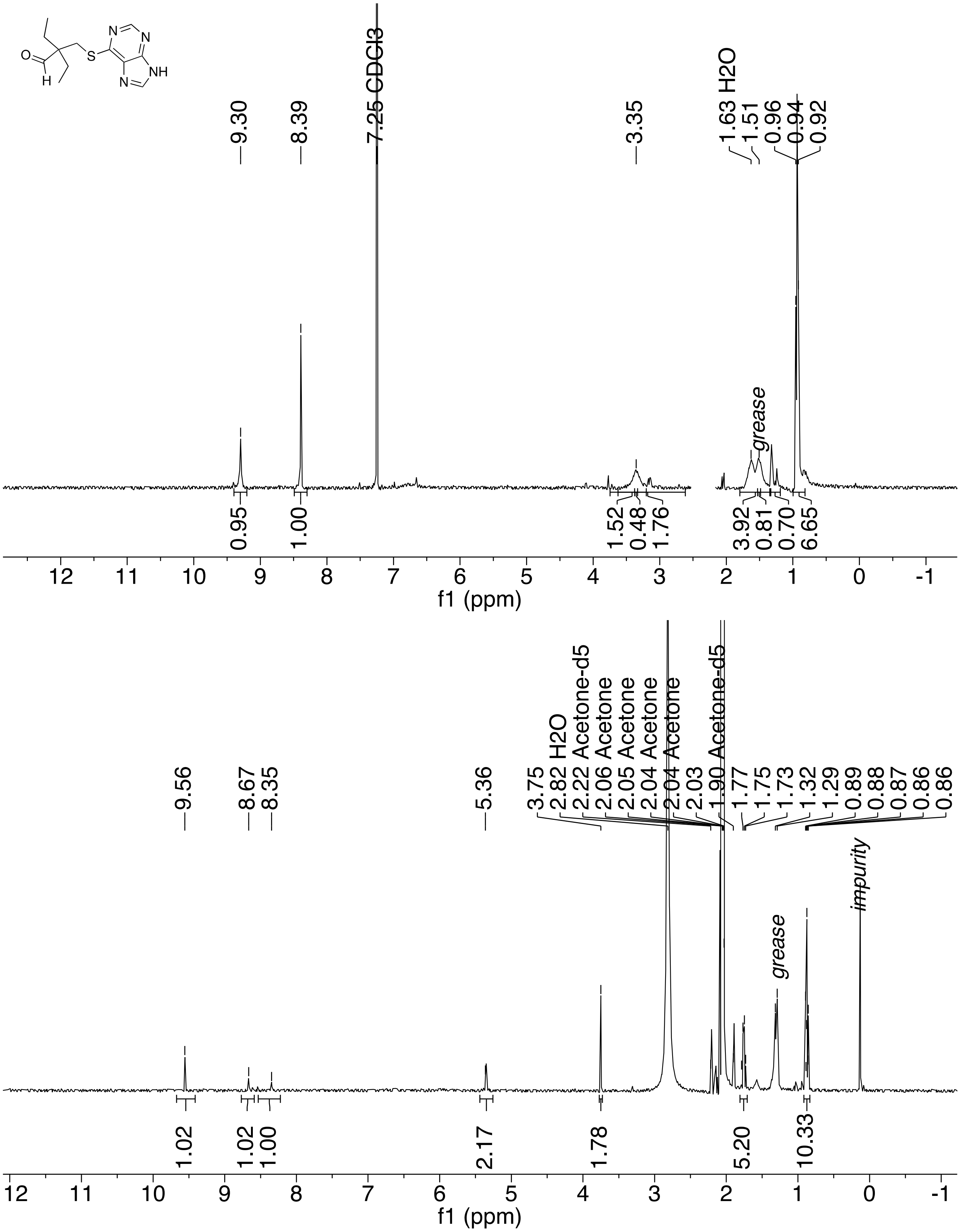

4e ^{1}H NMR (700 MHz, $CDCl_3$) and ^{13}C NMR (176 MHz, $CDCl_3$)

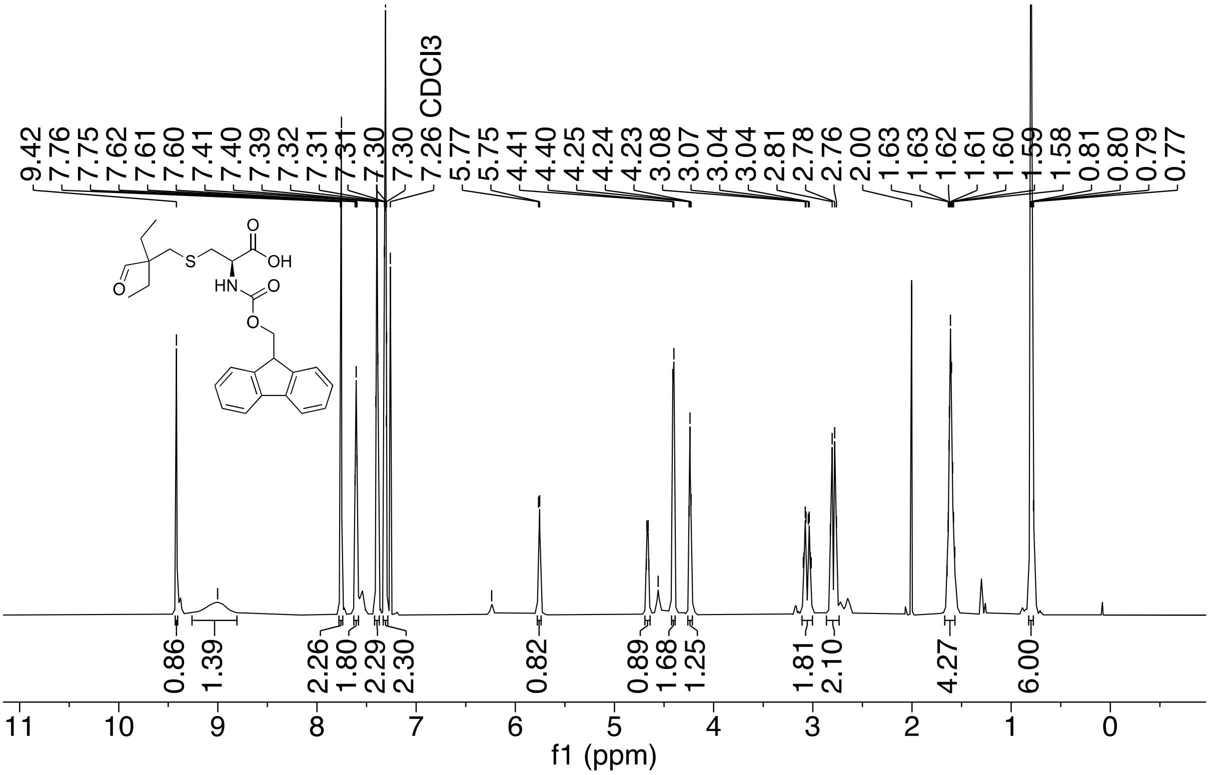

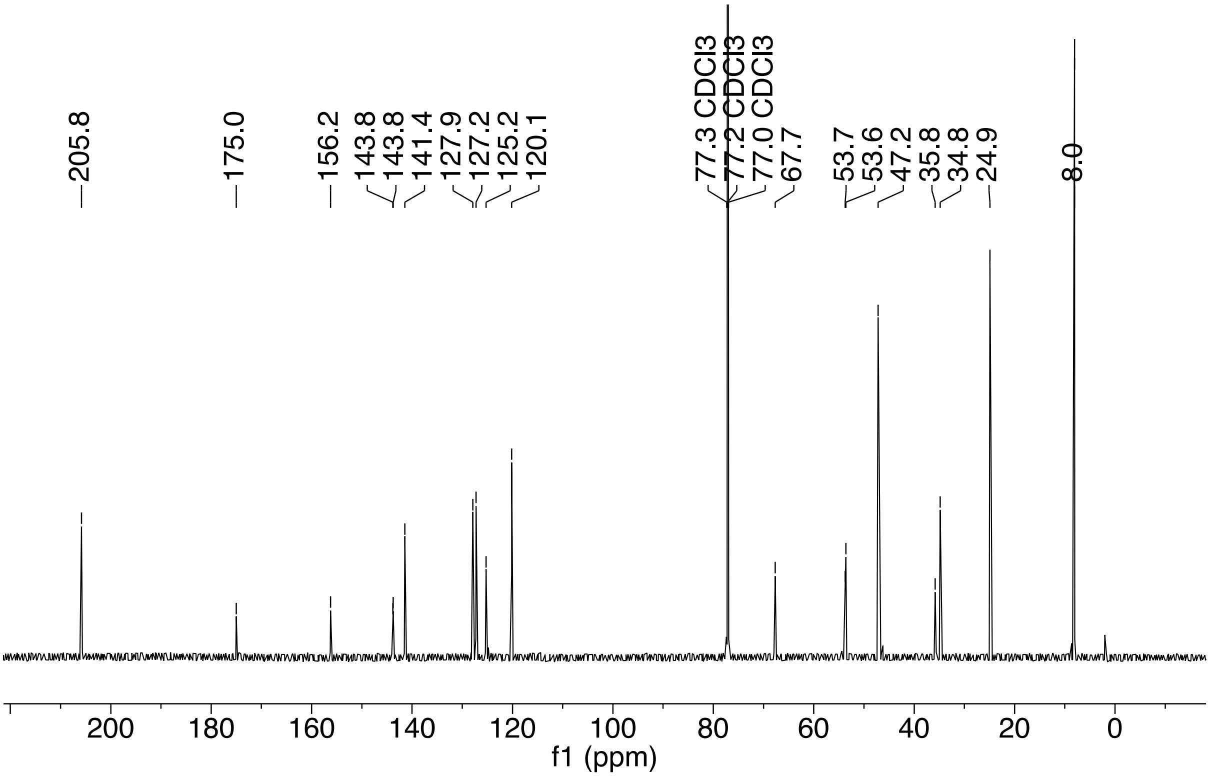

2.2 Spectra of the Supporting Information (SI) molecules

SI-4 ^{1}H NMR (400 MHz, $CDCl_3$) and ^{13}C NMR (101 MHz, $CDCl_3$)

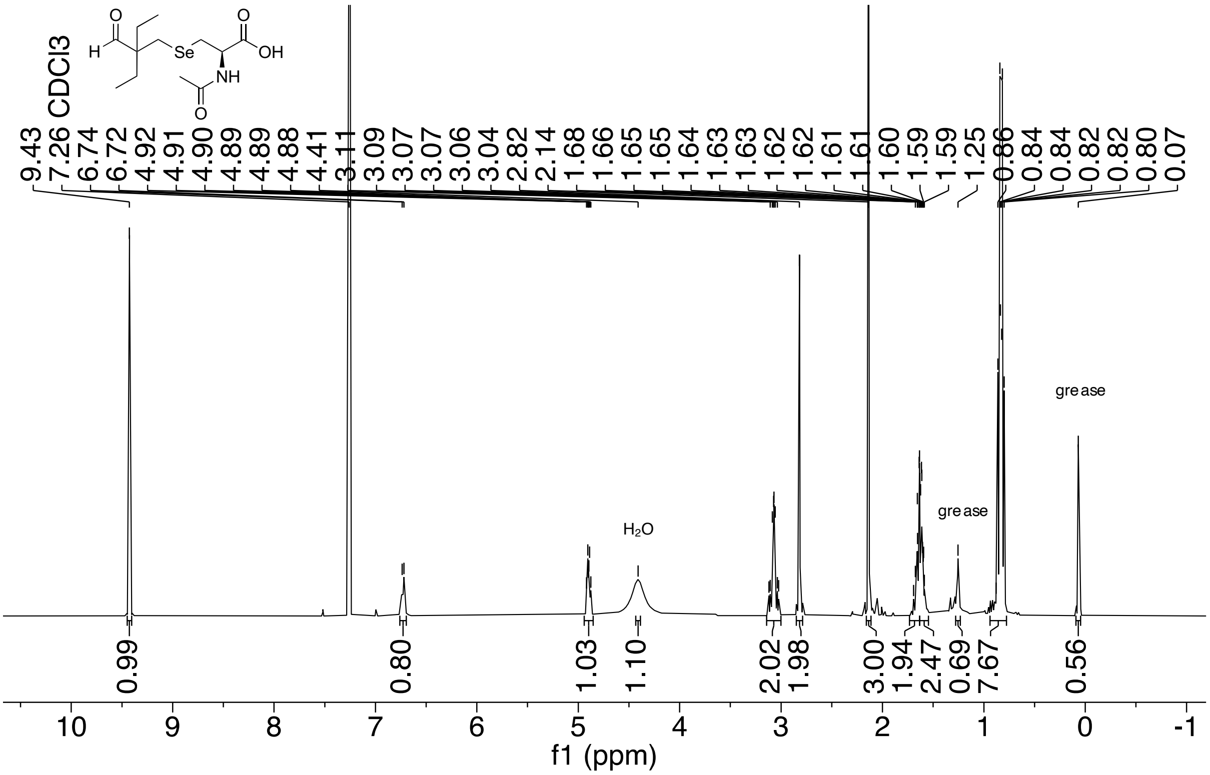

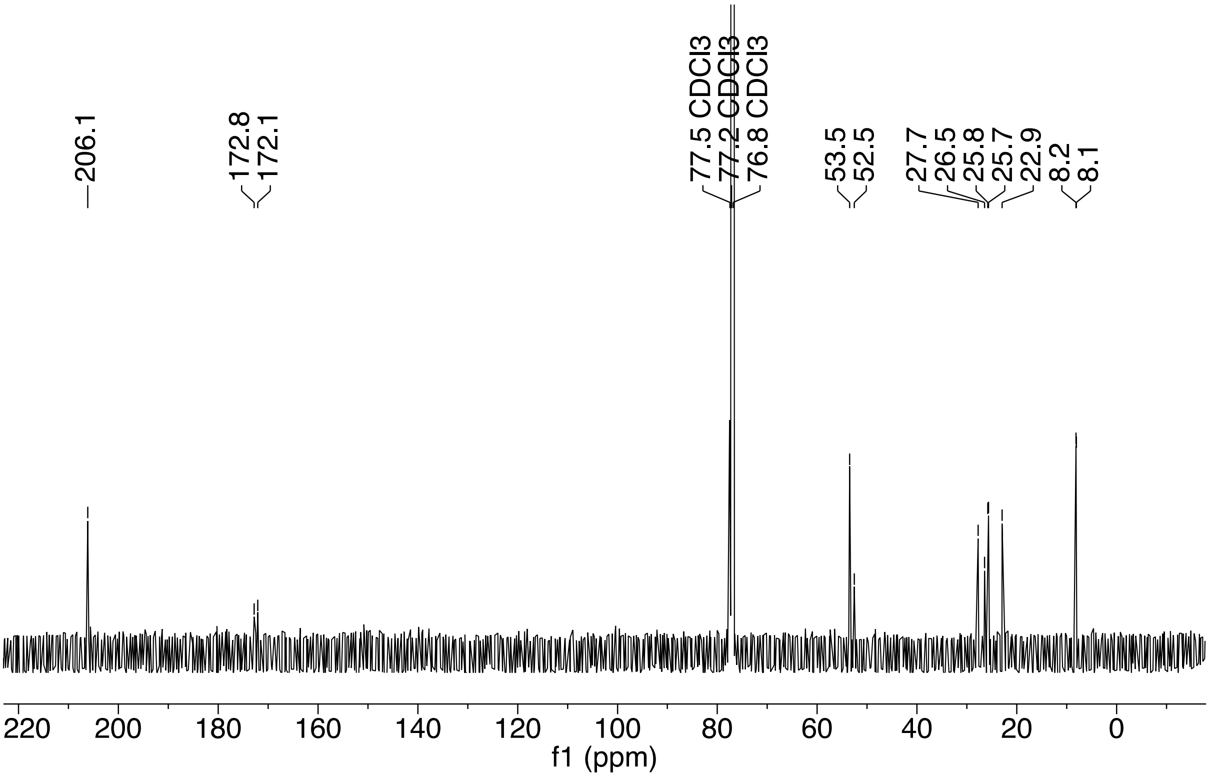

SI-6 ^{1}H NMR (500 MHz, $CDCl_3$) and ^{13}C NMR (126 MHz, $CDCl_3$)

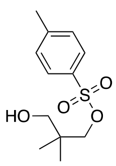

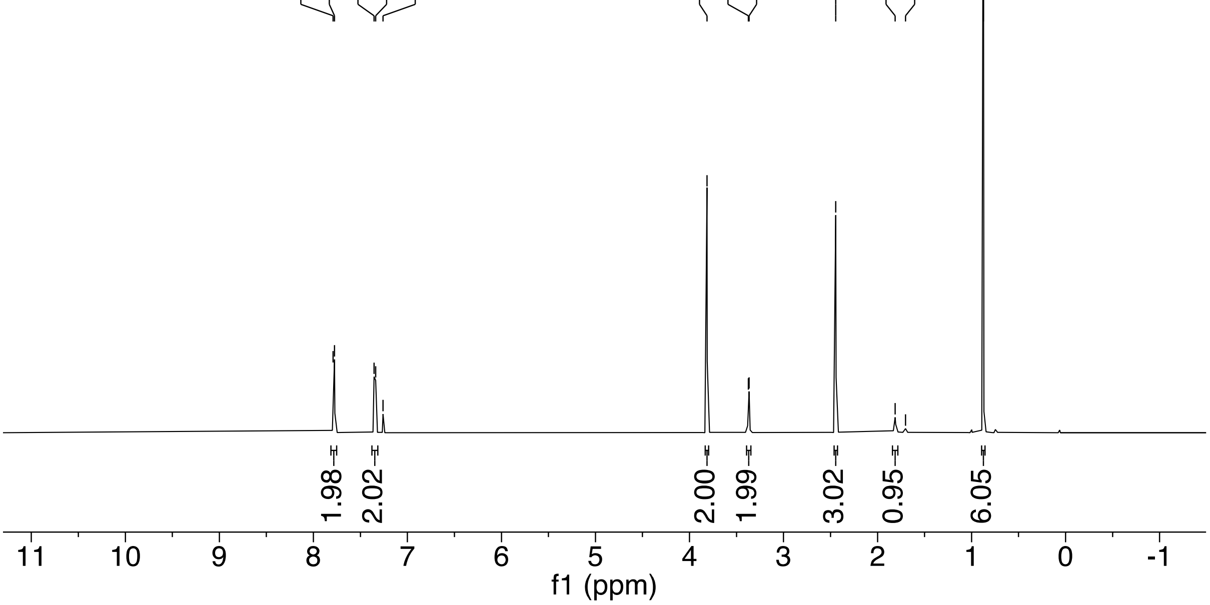

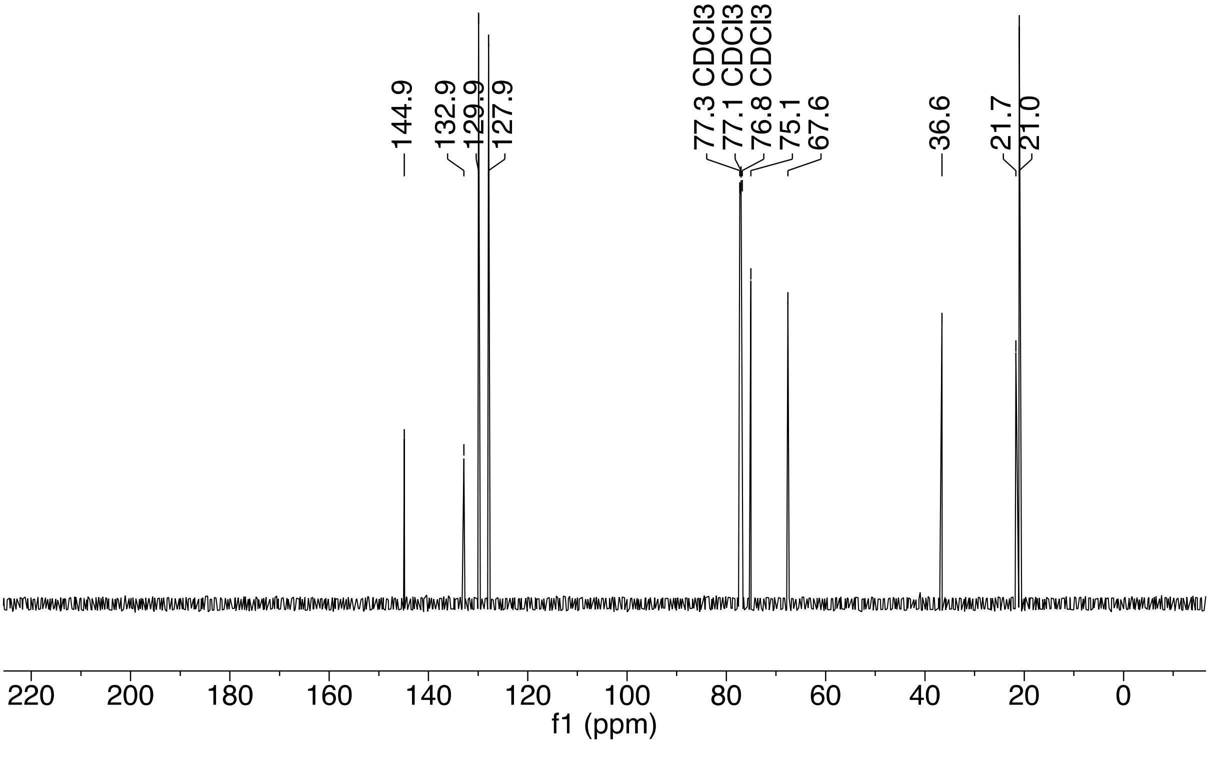

SI-7 ^{1}H NMR (400 MHz, $CDCl_3$) and ^{13}C NMR (101 MHz, $CDCl_3$)

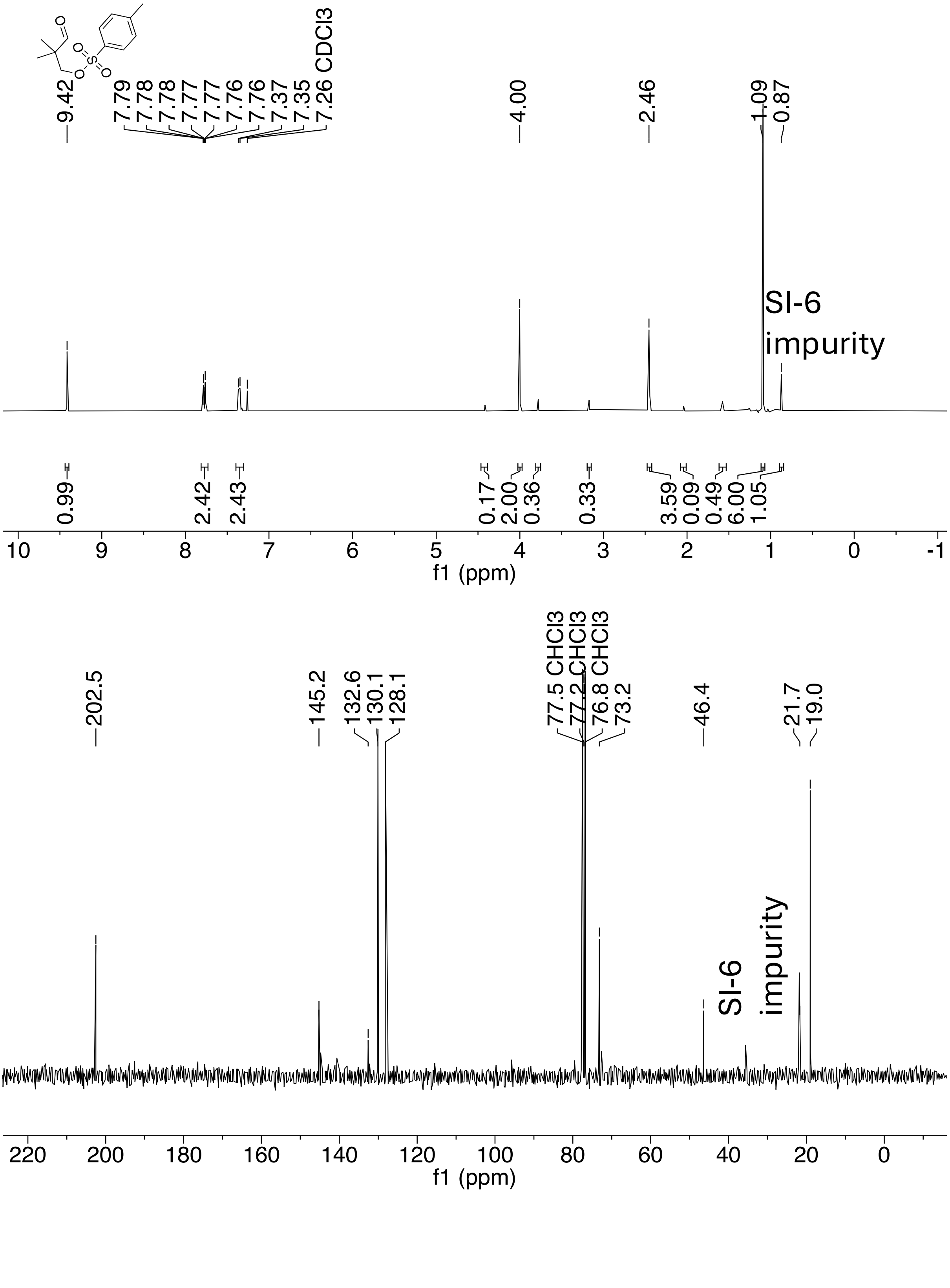

SI-8 ^{1}H NMR (500 MHz, $CDCl_3$) and ^{13}C NMR (126 MHz, $CDCl_3$)

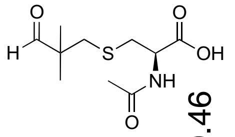

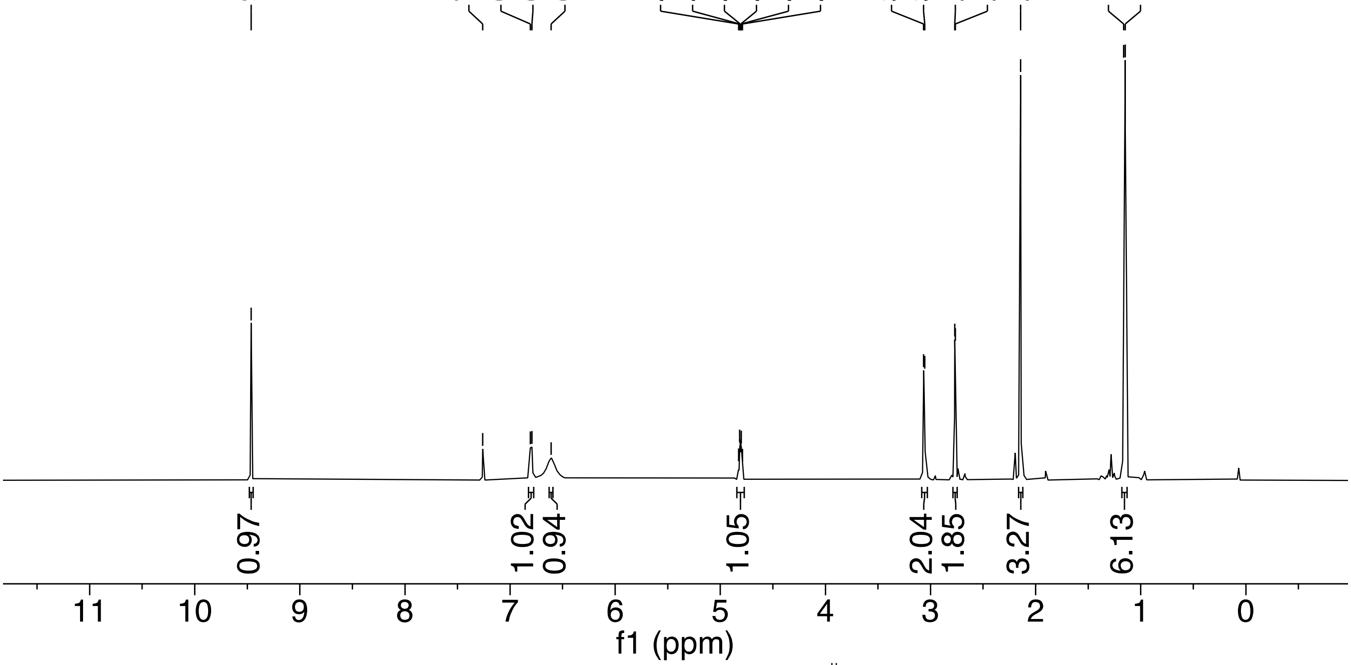

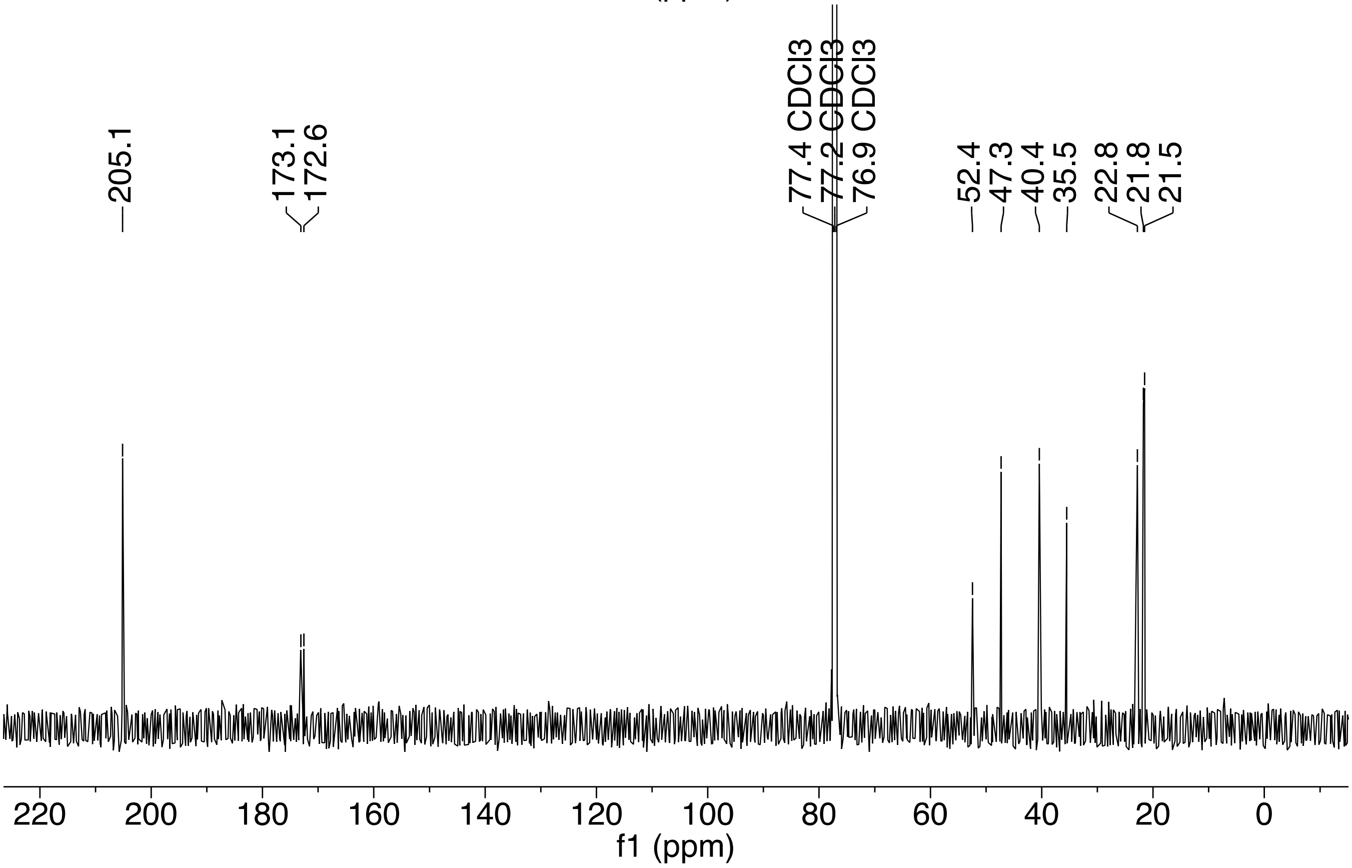

SI-9 ^{1}H NMR (500 MHz, $CDCl_3$) and ^{13}C NMR (126 MHz, $CDCl_3$)

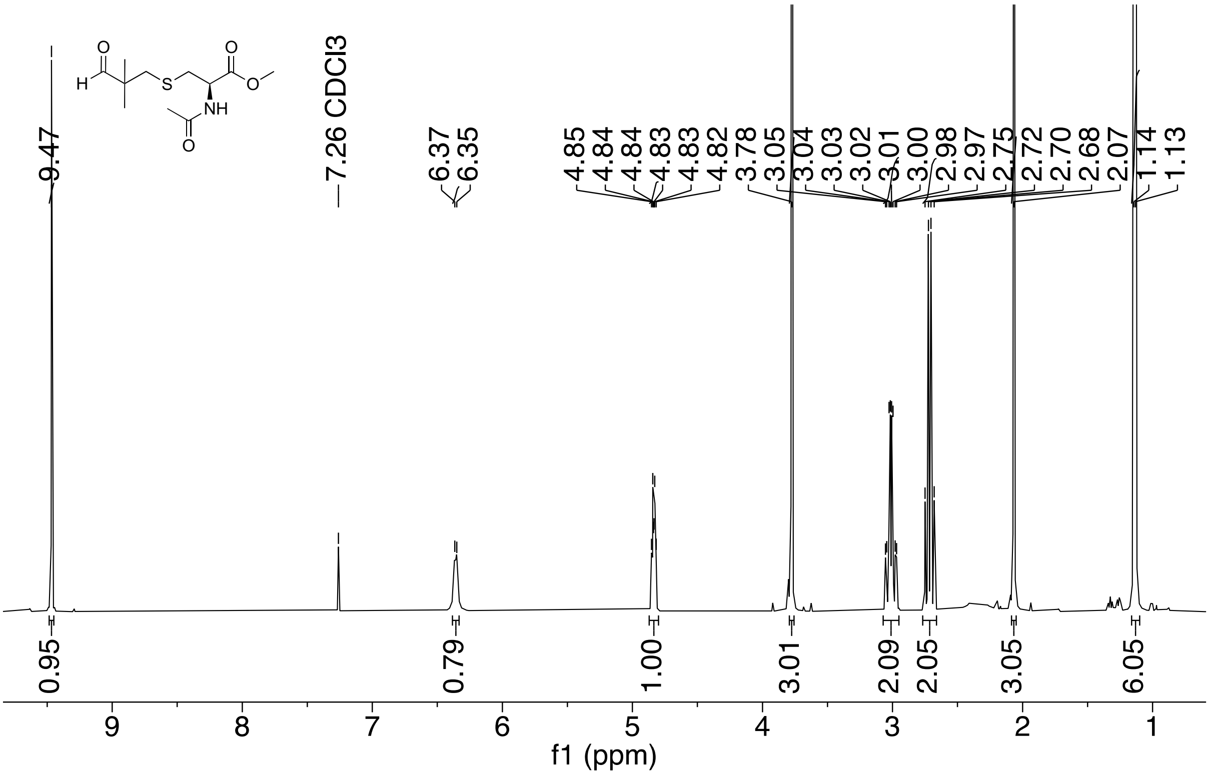

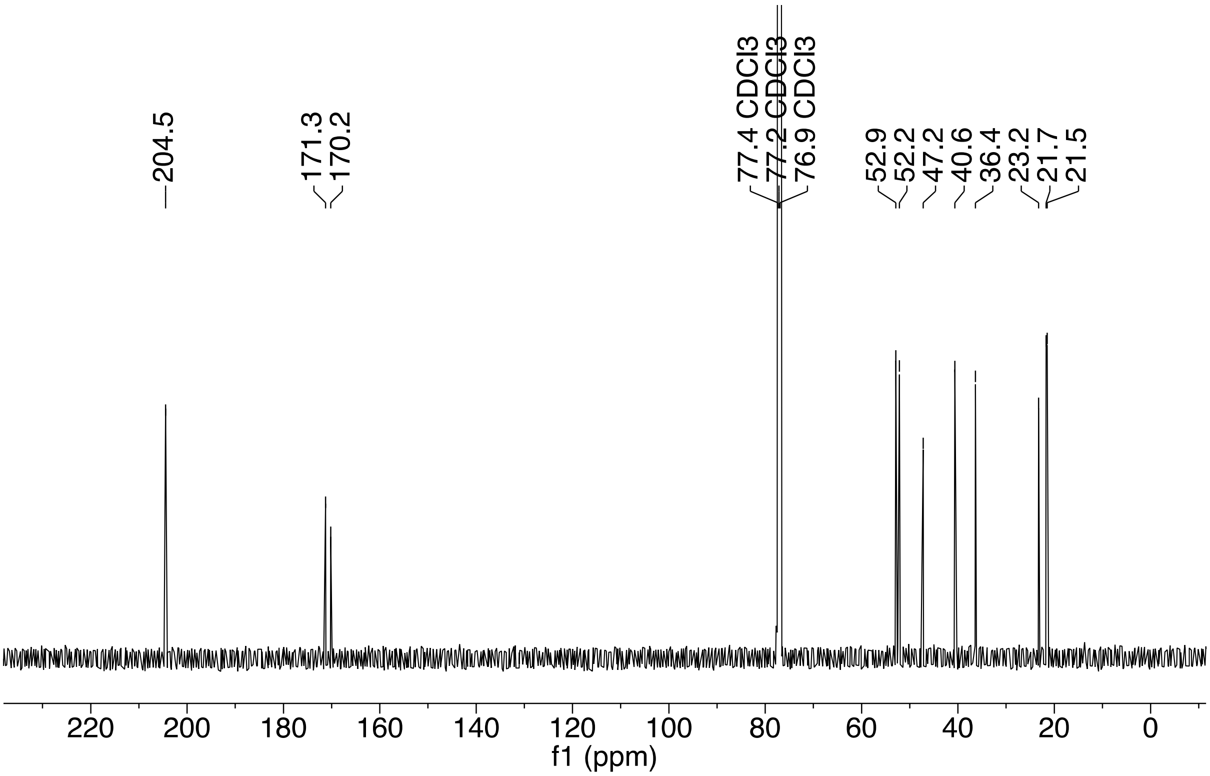

SI-11 ^{1}H NMR (400 MHz, $CDCl_3$) and ^{13}C NMR (101 MHz, $CDCl_3$)

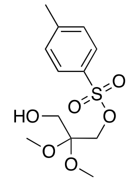

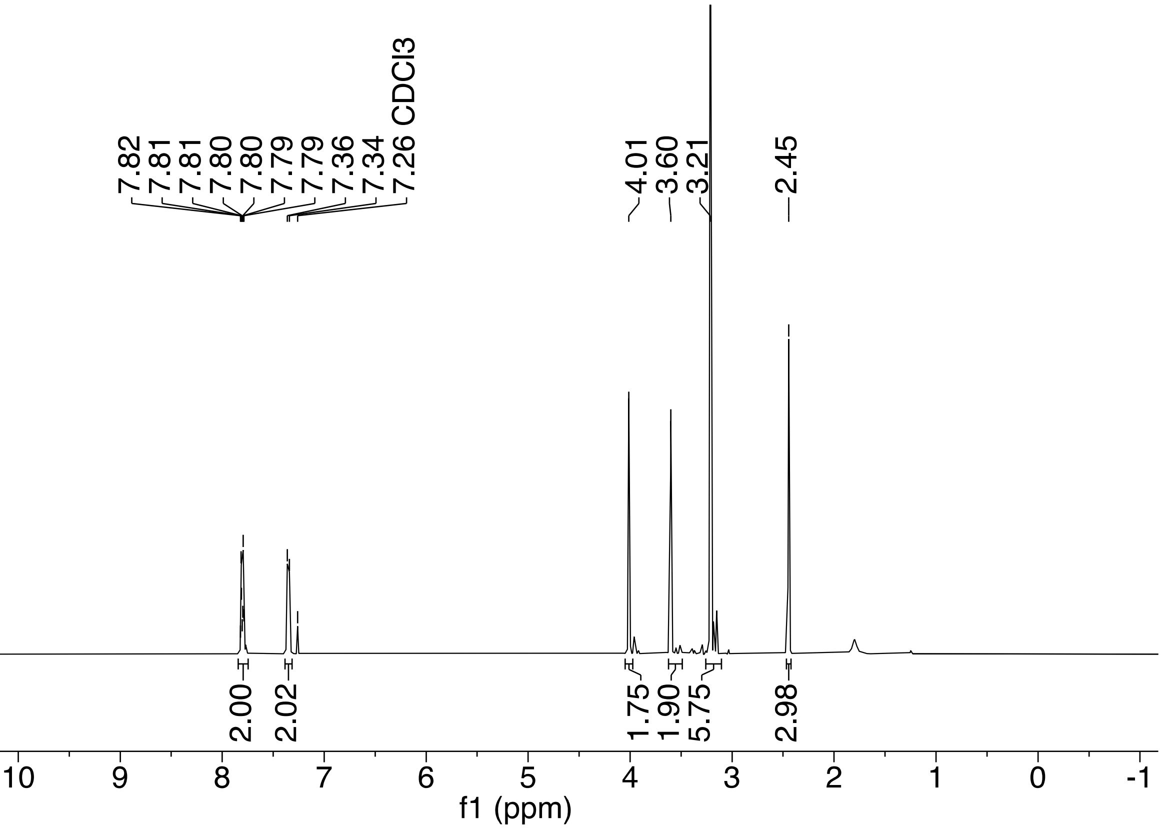

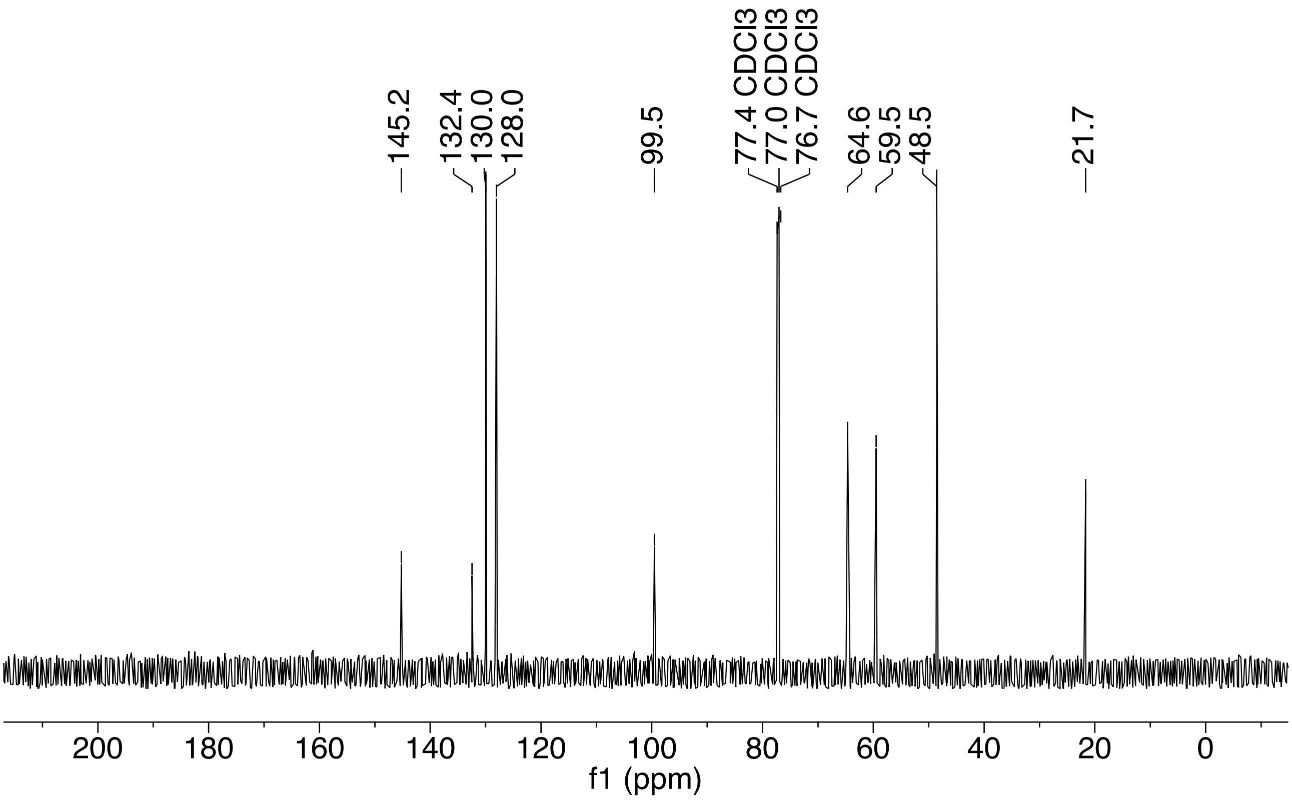

SI-12 ^{1}H NMR (500 MHz, $CDCl_3$) and ^{13}C NMR (126 MHz, $CDCl_3$)

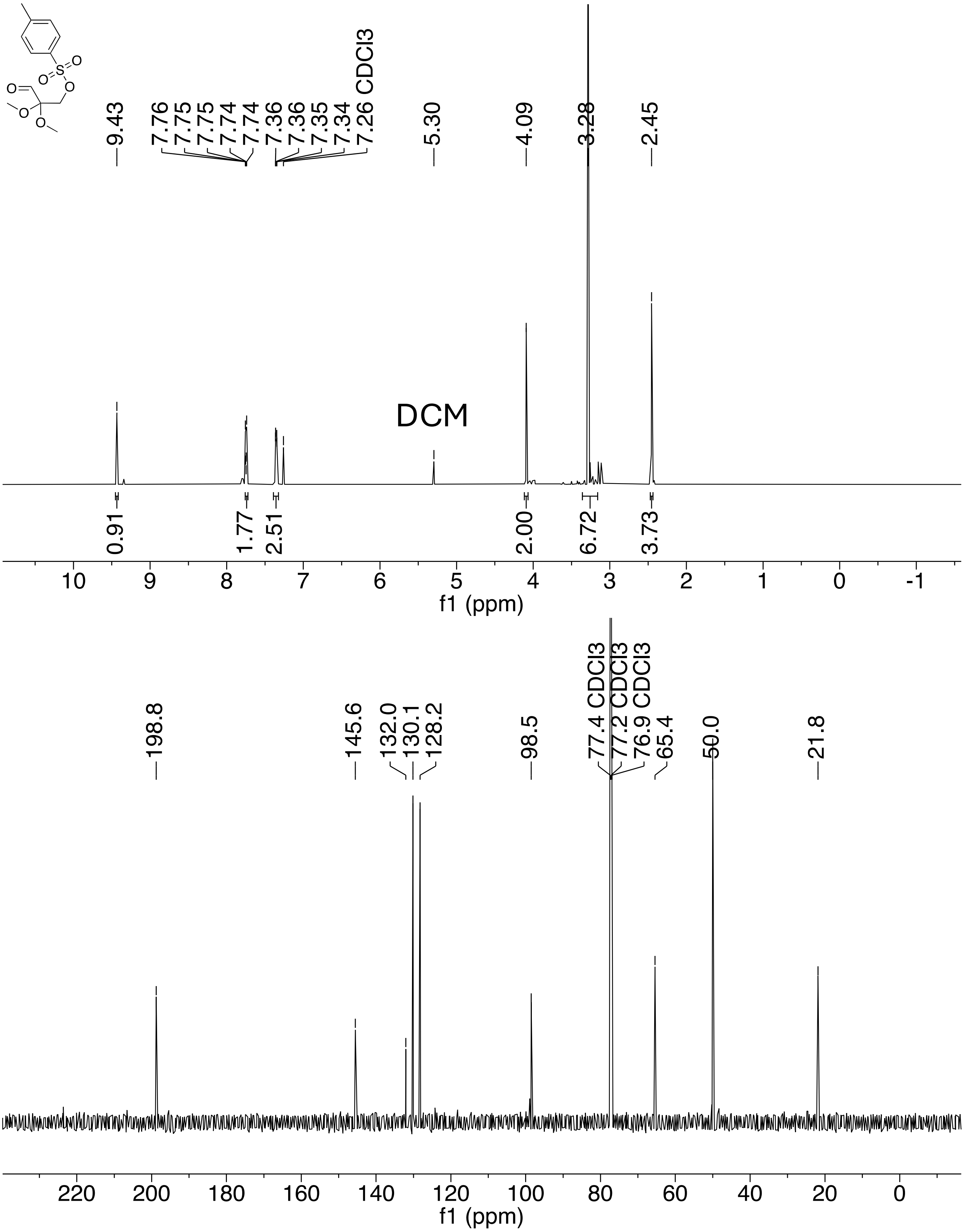

SI-15 ^{1}H NMR (400 MHz, $CDCl_3$) and ^{13}C NMR (101 MHz, $CDCl_3$)

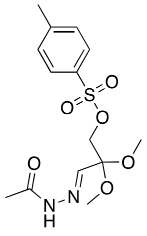

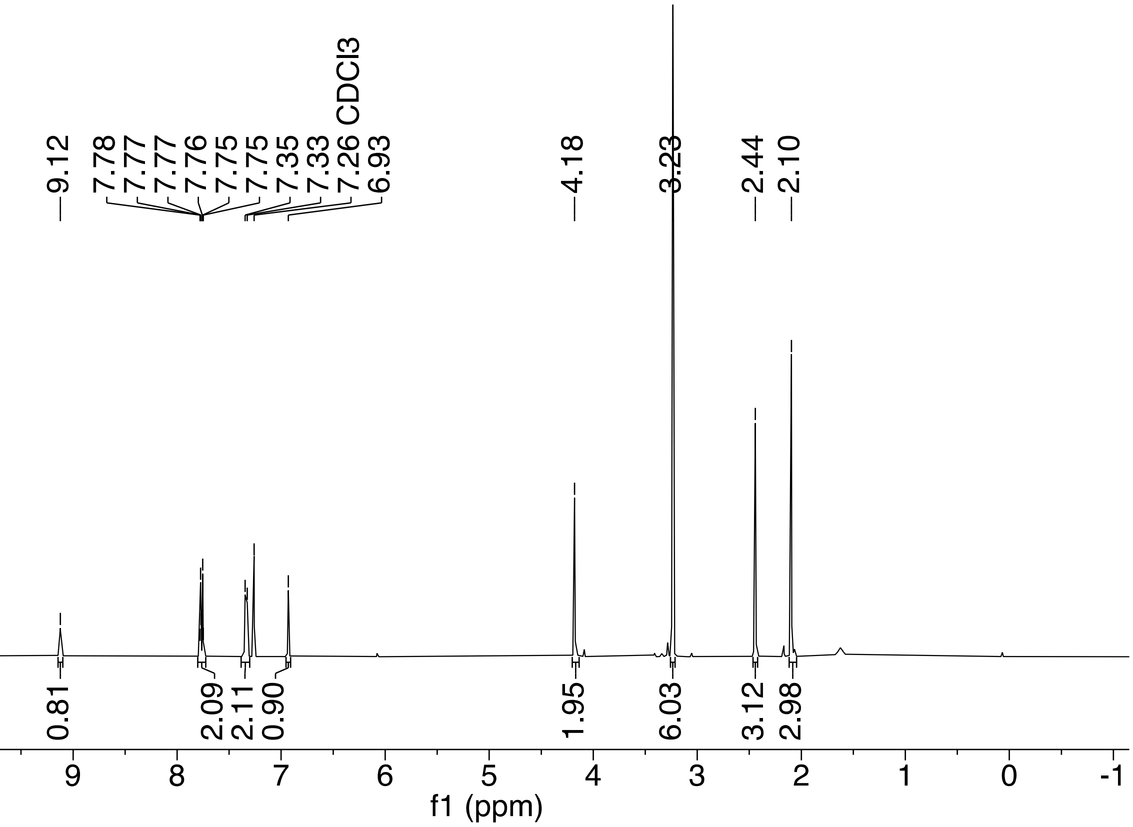

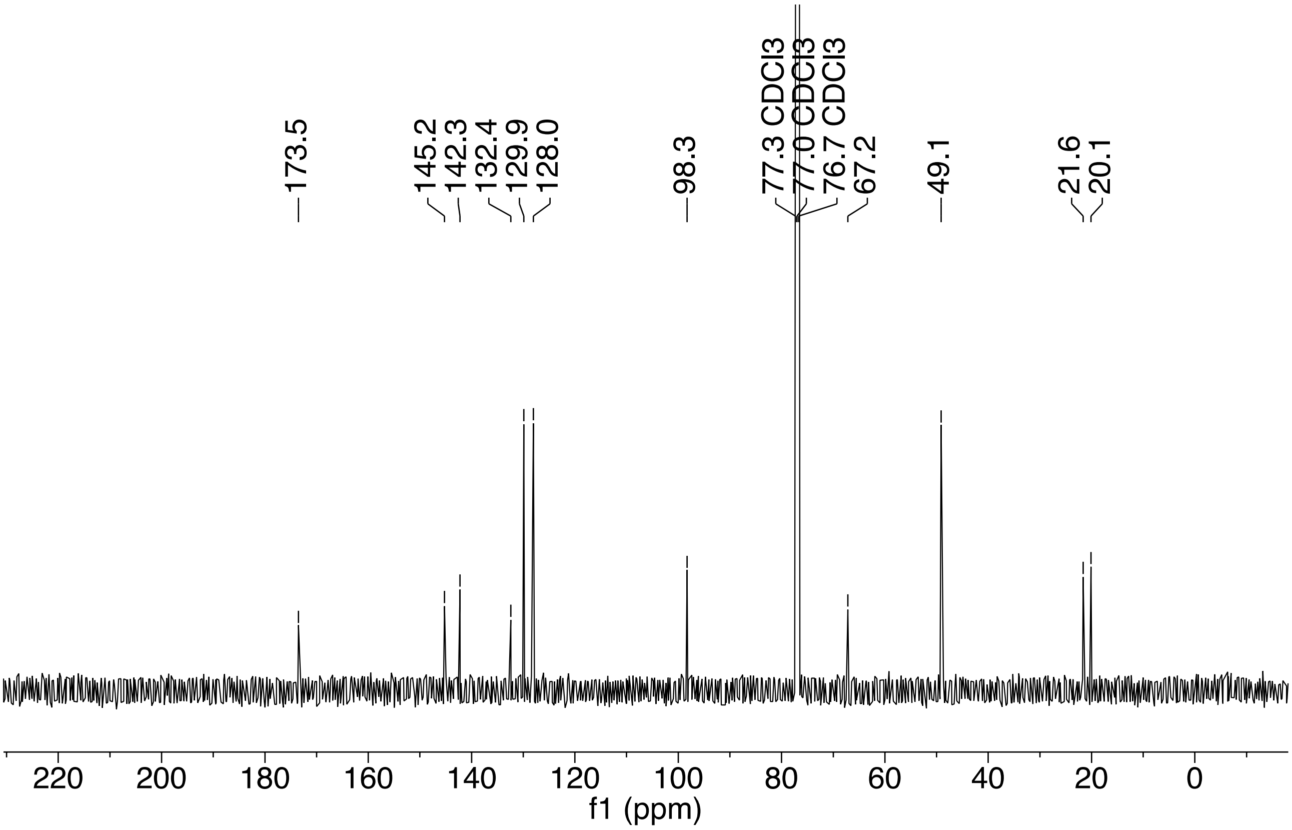

4 Protein LC-MS spectra

Unconjugated Trastuzumab

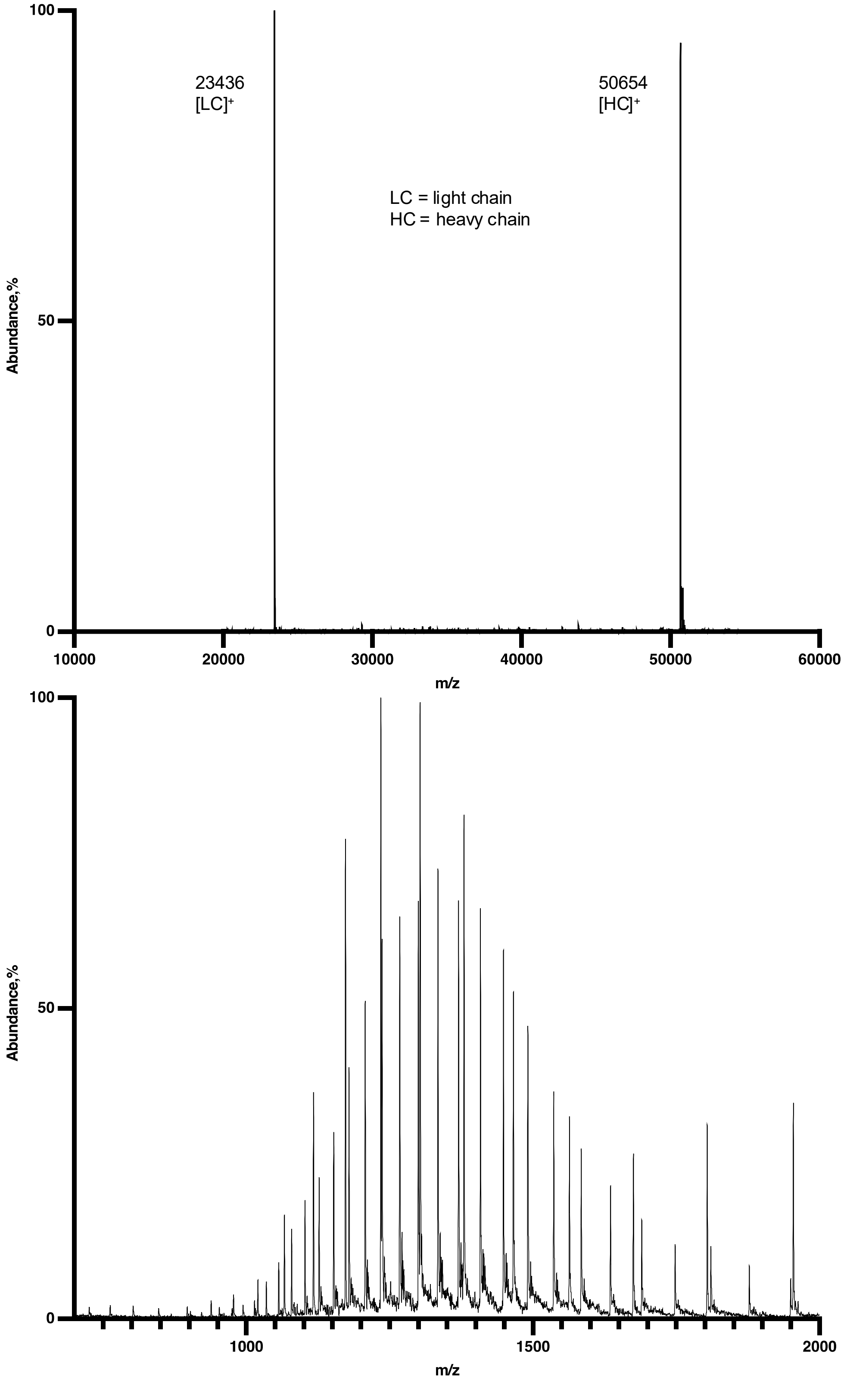

Figure S- 40 LC-MS Data for pure commercially sourced reduced Trastuzumab antibody.

BC1 Trastuzumab-**5** conjugate

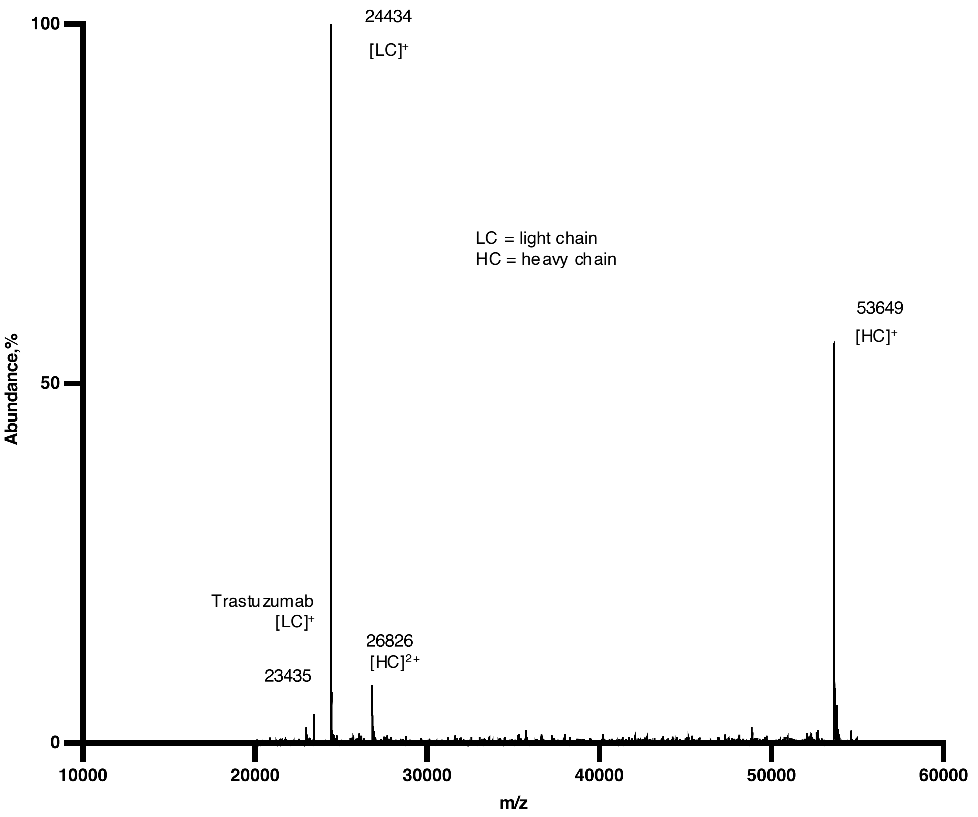

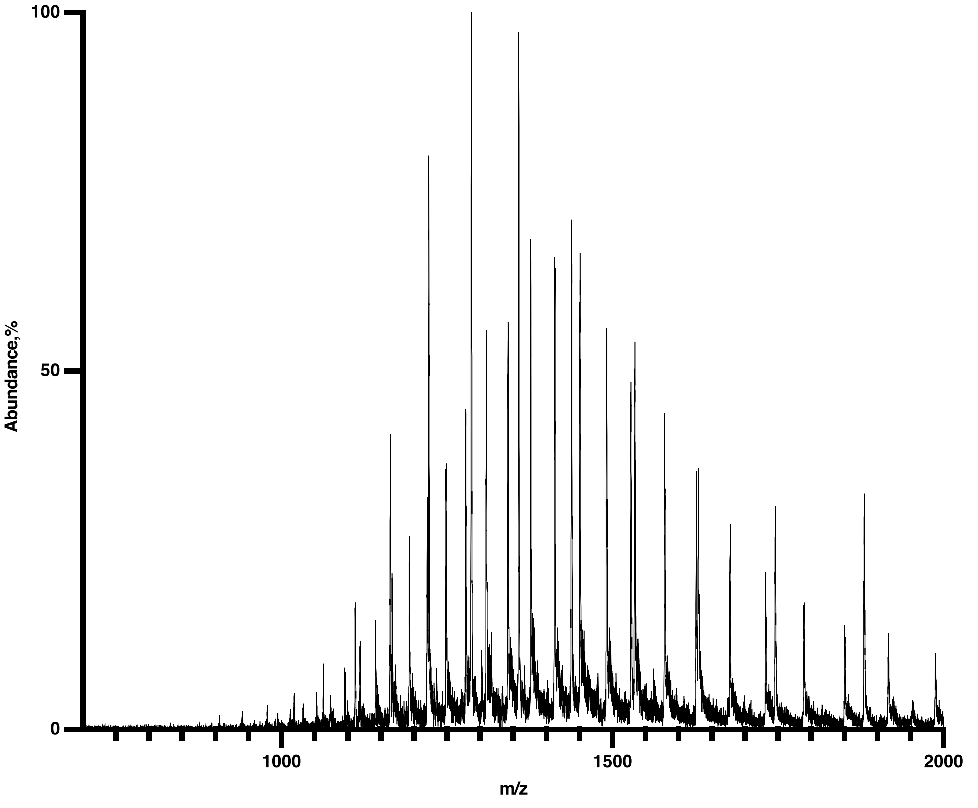

*Figure S- 41 LC-MS spectra of **BC1**, ion series and deconvoluted spectra (calcd. For LC 24,434 Da, HC 53,648 Da)*

BC2 Trastuzumab-**7** conjugate

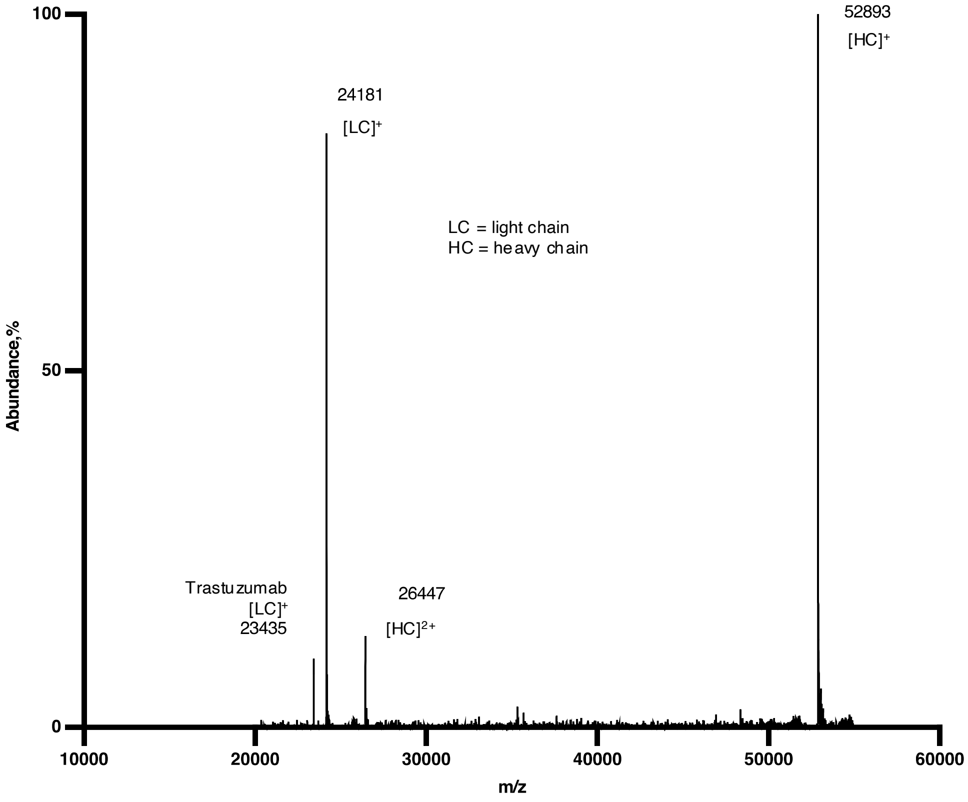

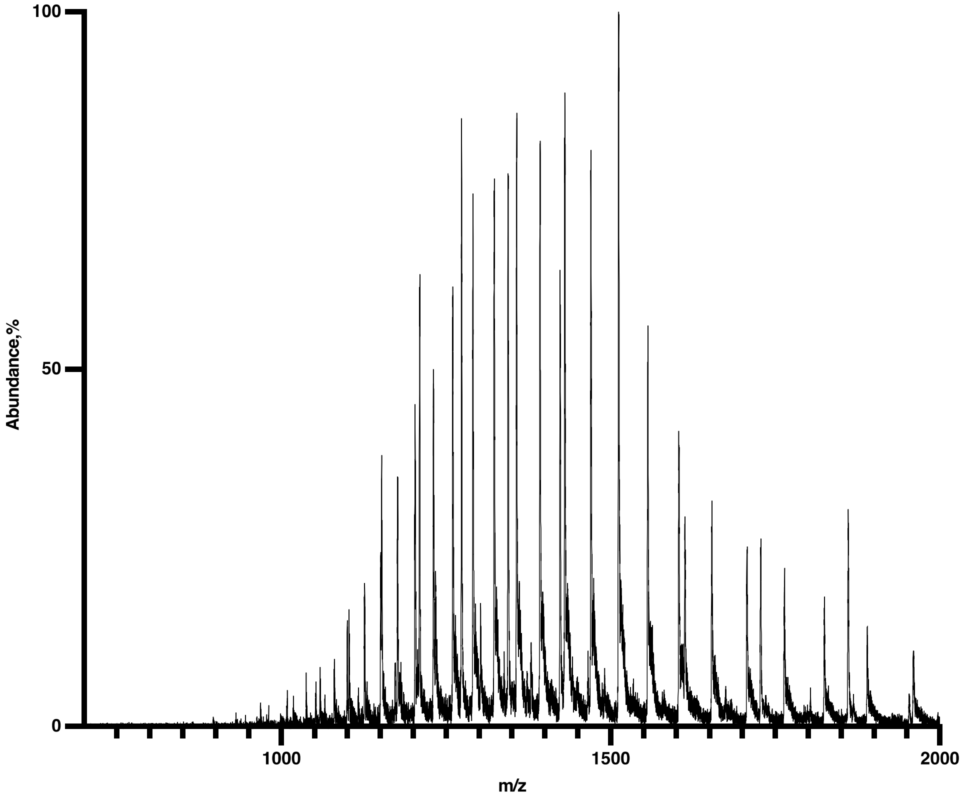

*Figure S- 42 LC-MS spectra of **BC2**, ion series and deconvoluted spectra (calcd. For LC 24,182 Da, HC 52,892 Da)*

Unconjugated Trastuzumab-LC-V205C

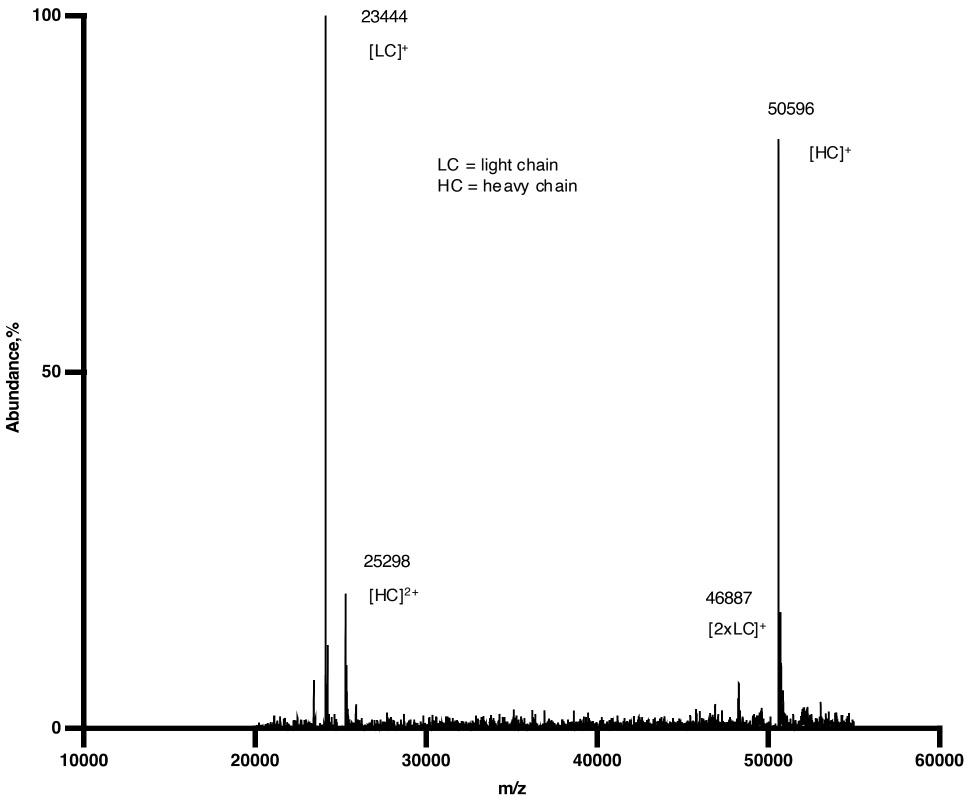

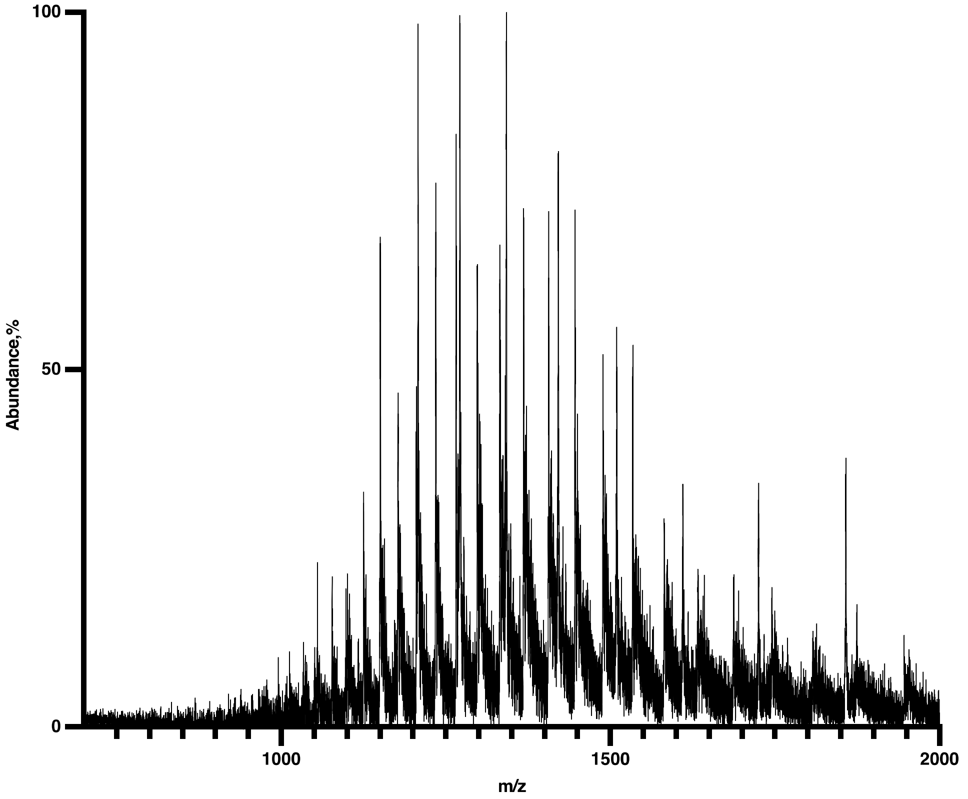

Figure S- 43 LC–MS spectra of reduced, commercially sourced Thiomab (Trastuzumab V205C (LC)); ion series and deconvoluted spectra. (The antibody was supplied by Genentech). Site-selective conjugation is expected to occur at the engineered cysteine residues in each light chain of thiomab.

BC3 Trastuzumab-LC-V205C AlexaFluor 488 nm

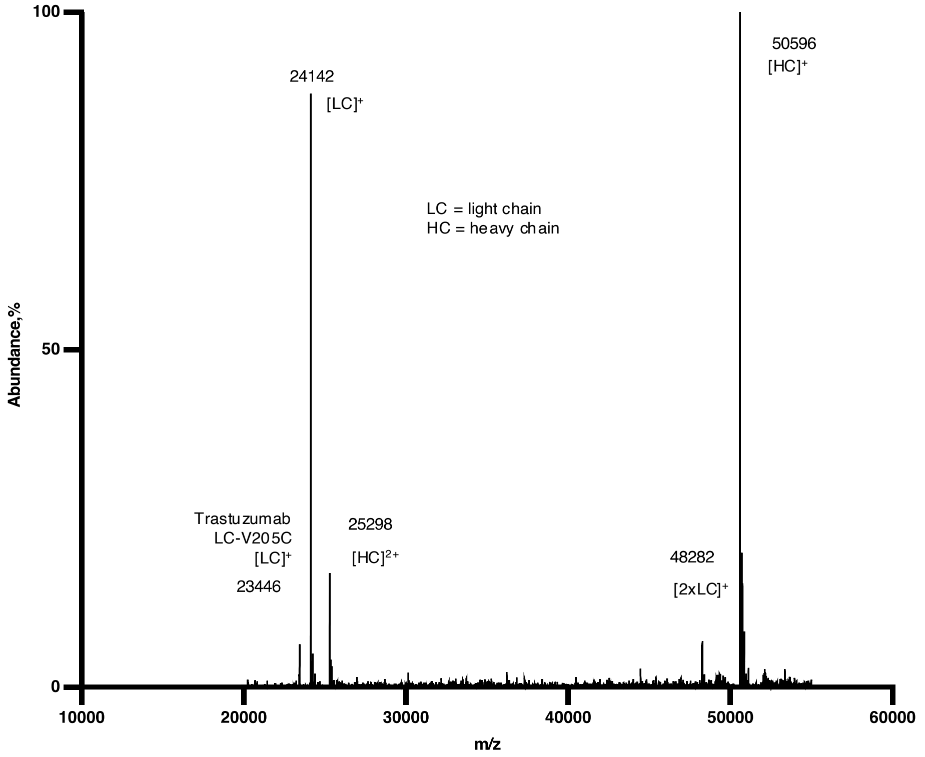

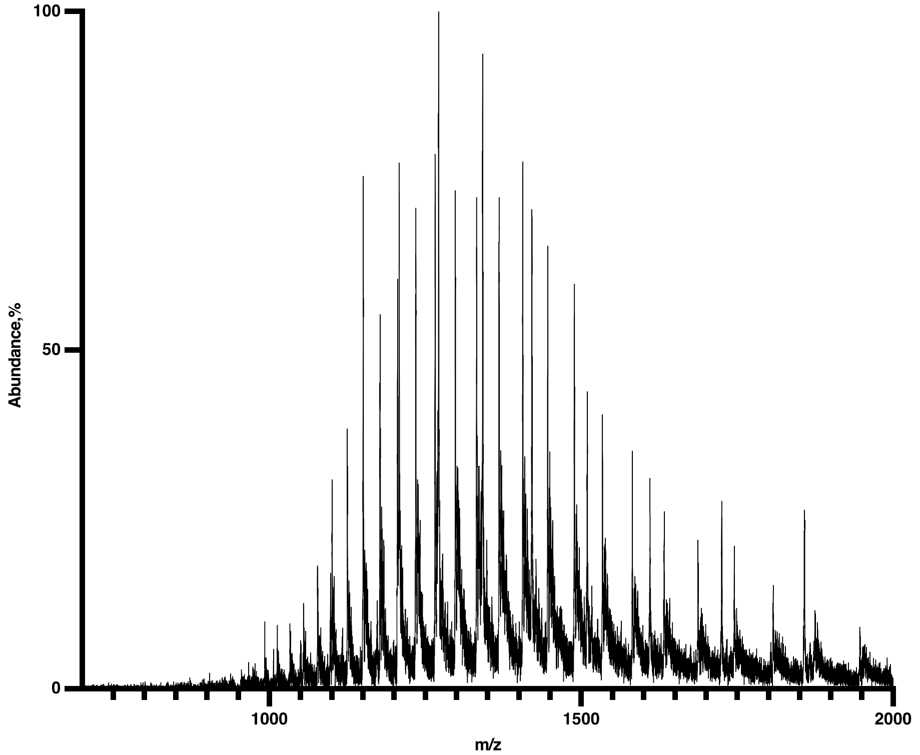

Figure S- 44 LC-MS spectra of trastuzumab LC V205C labelled with Alexa Fluor 488 maleimide after UF/DF ion series and deconvoluted spectra (Calculated masses: LC: 24142 Da, HC: 50596 Da).

5 Uncropped gels

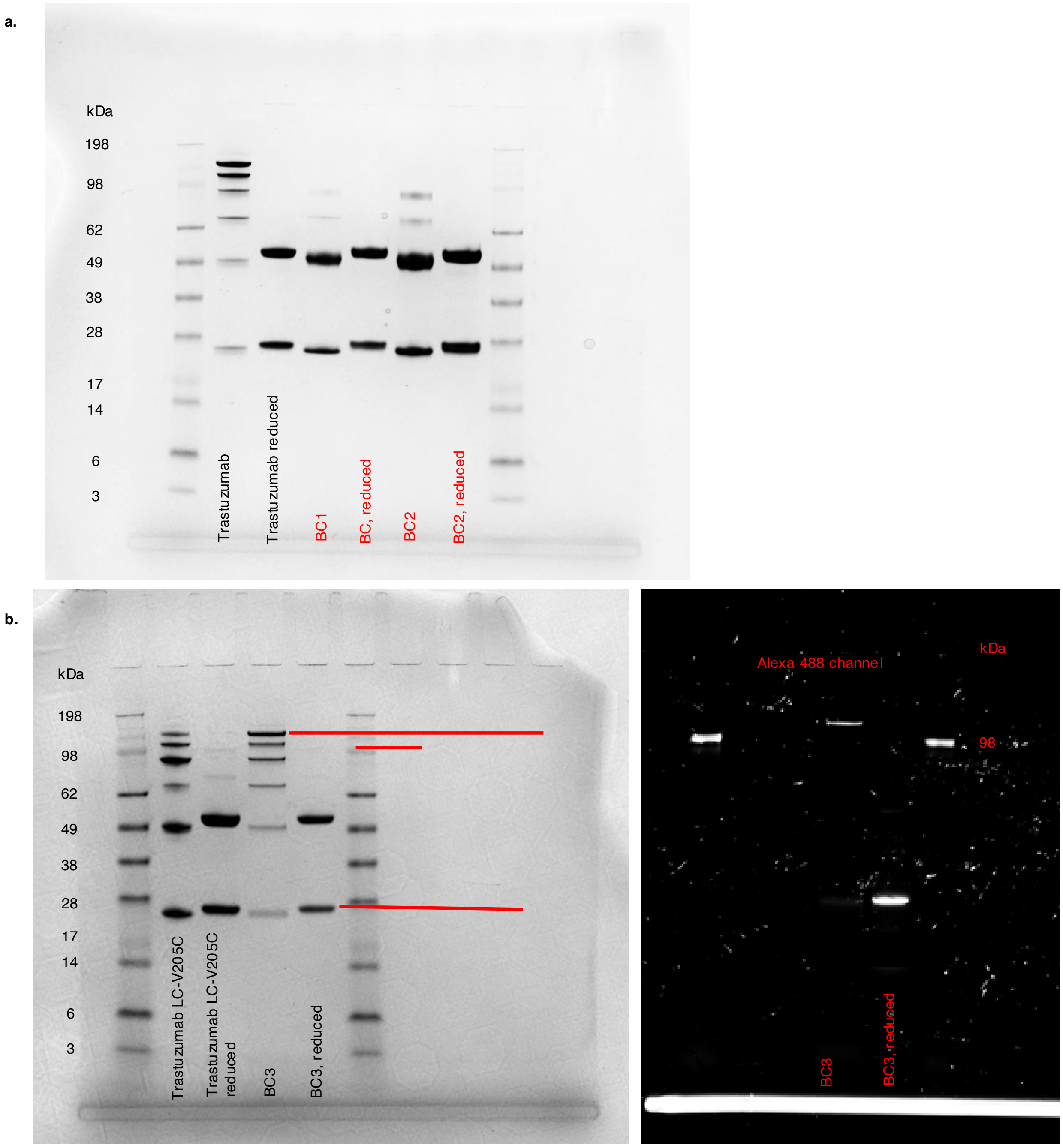

Figure S- 45 Uncropped SDS-PAGE gels (Figures S-29 and S-32)

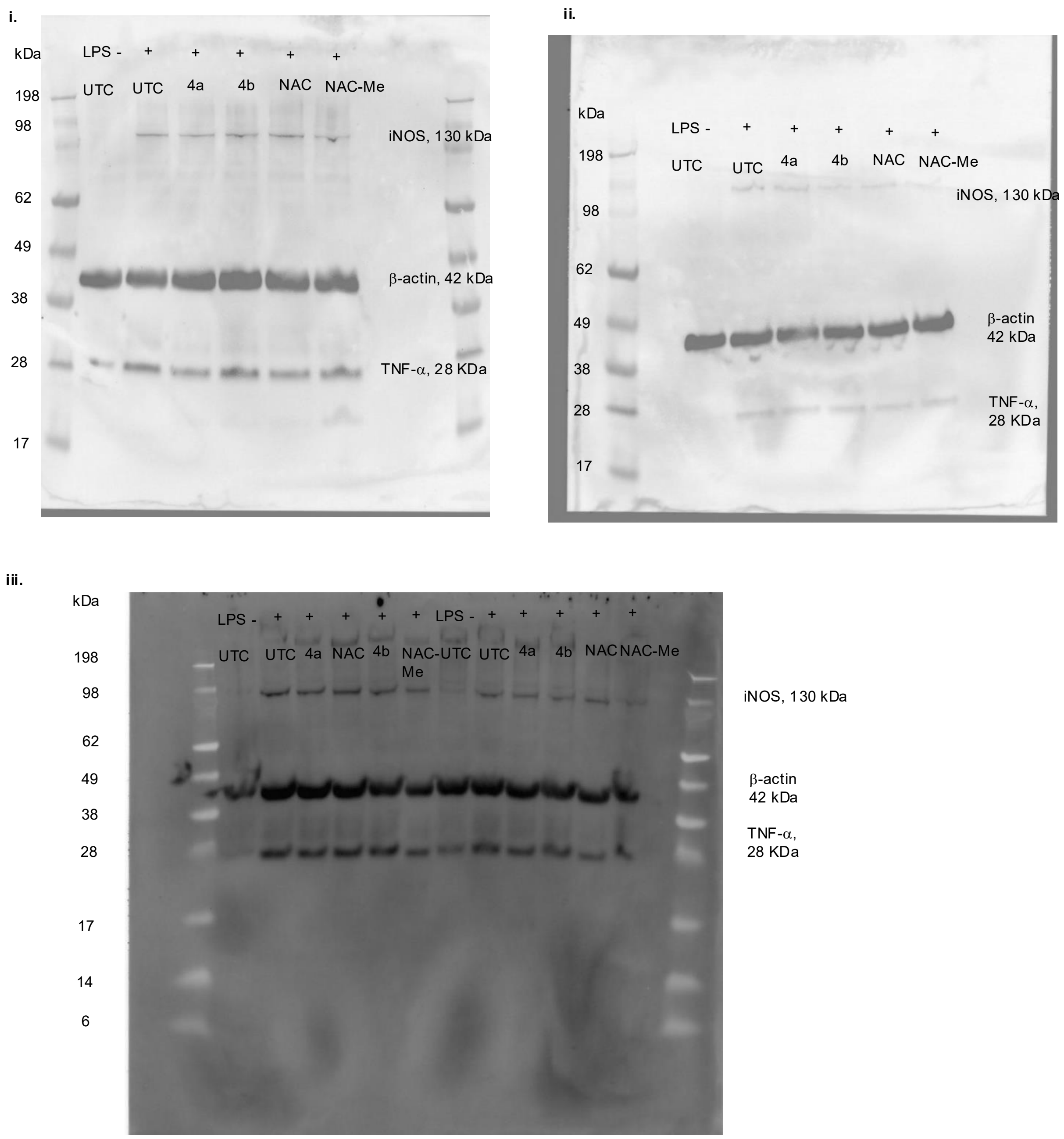

Figure S- 46 Uncropped Western blot membranes used for quantification in Figure S-10.

6 Additional Computational Data

Cartesian coordinates of the lowest energy structures calculated with PCM(H_2O)/M06-2X/6-311+G(2d,p)

Structure **A**

C	0.765416	-2.579625	-0.374327
C	0.972501	-1.253290	-1.098407
H	1.006839	-3.412500	-1.034936
H	1.402618	-2.660454	0.509919
H	-0.271563	-2.698188	-0.056126
C	0.816323	-0.000002	-0.208463
H	1.971299	-1.224760	-1.545030
H	0.265229	-1.169469	-1.927160
C	0.972519	1.253292	-1.098396
H	1.971308	1.224739	-1.545036
H	0.265231	1.169499	-1.927139
C	0.765483	2.579624	-0.374297
C	-0.478148	0.000003	0.600591
H	-0.519981	0.879414	1.244018
H	1.006911	3.412500	-1.034903
H	-0.271485	2.698209	-0.056070
H	1.402708	2.660428	0.509935
S	-1.947082	0.000024	-0.472983
C	-3.210237	0.000007	0.824895
H	-3.118301	0.892650	1.441265
H	-4.179679	0.000021	0.331215
H	-3.118309	-0.892658	1.441234
H	-0.519997	-0.879419	1.244002
C	1.989623	-0.000018	0.748296
O	1.914947	-0.000041	1.950325
H	2.980034	-0.000009	0.250586

Structure **TS1**

C	-1.519782	-2.422446	-0.367676
C	-1.254668	-1.027439	-0.923557
H	-1.939322	-3.060163	-1.146015
H	-0.601875	-2.895420	-0.014286
H	-2.230619	-2.399126	0.460546
C	-0.722788	-0.033513	0.118390
H	-0.537376	-1.090477	-1.746420
H	-2.167516	-0.614735	-1.354969
C	-1.721496	0.226440	1.277601
H	-1.709143	-0.652872	1.926356
H	-1.334589	1.052636	1.882065
C	-3.149186	0.530303	0.837499
C	0.603200	-0.465659	0.748987
H	0.476447	-1.403807	1.288922
H	-3.750147	0.816199	1.700736
H	-3.182355	1.351528	0.118679
H	-3.620056	-0.339187	0.377531
S	1.915597	-0.665559	-0.501578
C	3.328307	-0.810091	0.624980
H	3.206293	-1.682732	1.263544
H	4.217848	-0.930598	0.010890
H	3.421773	0.089432	1.231726
H	0.937550	0.295802	1.457424
C	-0.506302	1.319863	-0.541468
O	-1.026754	1.716531	-1.538604
H	0.224685	2.006091	0.026623
O	1.708499	2.576817	0.300033
H	2.141164	1.854847	-0.198076

Structure **B**

C	0.824884	-2.577747	-0.361161
C	1.001428	-1.255353	-1.098875
H	1.066627	-3.411552	-1.020225
H	1.481496	-2.641587	0.510350
H	-0.204034	-2.710302	-0.022987
C	0.835027	0.000024	-0.222075
H	1.994207	-1.209458	-1.553741
H	0.277765	-1.186252	-1.914780
C	1.001409	1.255438	-1.098819
H	1.994182	1.209568	-1.553700
H	0.277734	1.186377	-1.914717
C	0.824874	2.577795	-0.361035
C	-0.459231	-0.000005	0.597223
H	-0.498258	0.881164	1.238495
H	1.066606	3.411635	-1.020058
H	-0.204039	2.710330	-0.022838
H	1.481500	2.641589	0.510470
S	-1.931442	0.000023	-0.473983
C	-3.189966	-0.000060	0.829296
H	-3.096258	0.892917	1.444682
H	-4.160163	-0.000048	0.337063
H	-3.096234	-0.893098	1.444590
H	-0.498253	-0.881216	1.238438
C	2.010051	-0.000003	0.770024
O	1.991167	-0.000121	1.944401

Structure **TS2**

C	0.793866	-2.563100	-0.409447
C	0.994023	-1.265056	-1.184745
H	1.104020	-3.415820	-1.013289
H	1.384068	-2.571388	0.509932
H	-0.254169	-2.708723	-0.142284
C	0.706826	0.000000	-0.420396
H	2.019847	-1.206093	-1.560692
H	0.349344	-1.272480	-2.074867
C	0.994010	1.265054	-1.184754
H	2.019836	1.206100	-1.560698
H	0.349334	1.272464	-2.074878
C	0.793836	2.563102	-0.409467
C	-0.450867	-0.000002	0.521444
H	-0.449505	0.884989	1.158518
H	1.103982	3.415820	-1.013315
H	-0.254202	2.708715	-0.142309
H	1.384035	2.571404	0.509914
S	-2.024572	-0.000018	-0.430584
C	-3.179169	0.000006	0.966151
H	-3.037887	0.892959	1.572478
H	-4.185164	-0.000001	0.551471
H	-3.037887	-0.892925	1.572509
H	-0.449493	-0.884987	1.158529
C	2.333779	0.000013	0.951099

O	2.053644	0.000019	2.057376

Structure **C**

C	-2.831851	-1.259201	0.050388
C	-1.588783	-0.621666	0.693995
H	-3.476622	-1.711366	0.806078
H	-3.411521	-0.508207	-0.489471
H	-2.539861	-2.035233	-0.659615
C	-0.699622	0.020891	-0.322372
H	-1.912694	0.120159	1.430525
H	-1.039664	-1.394230	1.241394
C	-0.865178	1.472281	-0.637944
H	-0.368227	1.704617	-1.583949
H	-1.930554	1.695365	-0.773707
C	-0.312412	2.397597	0.459264
C	0.343750	-0.776310	-0.996866
H	0.368693	-0.605130	-2.076176
H	-0.502979	3.444310	0.215850
H	-0.782402	2.185341	1.421473
H	0.764331	2.258293	0.567059
S	2.070681	-0.327524	-0.475969
C	1.916595	-0.591561	1.308811
H	1.642912	-1.625004	1.518200
H	2.888839	-0.380041	1.749532
H	1.173965	0.084411	1.731868
H	0.219901	-1.845088	-0.815213

Structure **TS3**

C	2.788078	-1.158763	-0.165915
C	1.260950	-1.093923	-0.284269
H	3.182378	-1.950605	-0.802697
H	3.250483	-0.219620	-0.473119
H	3.088337	-1.364287	0.862960
C	0.652288	-0.070877	0.629867
H	1.001812	-0.854243	-1.321799
H	0.829026	-2.072998	-0.069935
C	1.129260	1.348463	0.520606
H	0.546494	1.973299	1.199630
H	2.171254	1.401515	0.854953
C	1.034957	1.902181	-0.905340
C	-0.345675	-0.404022	1.495685
H	-0.655240	0.294702	2.263267
H	1.333498	2.950441	-0.924831
H	1.685473	1.356496	-1.590606
H	0.010071	1.830606	-1.275200
S	-2.340812	0.176890	0.242534
C	-1.961457	-0.807487	-1.230625
H	-1.610058	-1.798951	-0.948889
H	-2.888193	-0.913775	-1.795302
H	-1.221868	-0.314453	-1.858641
H	-0.620891	-1.441799	1.639613

Structure **D**

C	0.000000	2.574570	0.028943
C	0.000251	1.264076	-0.745352
H	0.000073	3.420641	-0.658645
H	-0.882784	2.658689	0.665061
H	0.882522	2.658844	0.665402
C	-0.000000	-0.000000	0.079900
H	-0.869323	1.229885	-1.411160
H	0.870277	1.229886	-1.410573
C	-0.000251	-1.264076	-0.745352
H	-0.870277	-1.229886	-1.410573
H	0.869323	-1.229885	-1.411160
C	-0.000000	-2.574570	0.028943
C	-0.000000	-0.000000	1.410180
H	0.000121	0.921592	1.978127
H	-0.000121	-0.921592	1.978127
H	-0.000073	-3.420641	-0.658645
H	0.882784	-2.658689	0.665061
H	-0.882522	-2.658844	0.665402

Structure $\mathbf{A_{OMe}}$

C	0.773842	2.315903	-0.273949
H	1.129468	3.014534	-1.026793
H	-0.254276	2.567293	-0.004854
H	1.412639	2.373539	0.608980
C	0.653210	-0.042532	0.022987
C	2.969804	-0.126851	0.539109
C	-0.682026	-0.003726	0.760207
H	-0.681988	0.810473	1.485432
H	3.606040	-0.188308	1.417922
H	3.144221	-1.000602	-0.092706
H	3.203554	0.780033	-0.020113
S	-2.036100	0.193670	-0.436064
C	-3.412370	-0.274224	0.644907
H	-3.464887	0.398129	1.499065
H	-4.322443	-0.181282	0.056285
H	-3.298753	-1.303333	0.979569
H	-0.802606	-0.949842	1.288241
C	0.687058	-1.293484	-0.877394
O	0.833480	-2.393053	-0.423228
O	0.838912	1.022154	-0.864367
O	1.628980	-0.098868	1.018628
H	0.558549	-1.111733	-1.957472

Structure $\mathbf{TS1_{OMe}}$

C	-3.011290	0.441648	-0.301410
H	-3.825557	0.447367	-1.020558
H	-3.251422	-0.230348	0.522888
H	-2.862138	1.453398	0.081860
C	-0.684523	-0.046087	-0.268408
C	-1.104271	-2.135070	0.796278
C	0.376000	-0.611005	-1.222103
H	0.487673	0.072866	-2.063654
H	-1.425056	-2.476912	1.776582
H	-1.905427	-2.293096	0.072849
H	-0.213208	-2.687369	0.492958
S	1.991914	-0.961445	-0.488713
C	2.757546	0.683837	-0.473033
H	2.379007	1.294430	0.343899
H	3.822757	0.518381	-0.323473
H	2.604150	1.176214	-1.431831
H	0.000336	-1.557550	-1.611534
C	-0.266097	1.377844	0.167633
O	-0.383885	2.340374	-0.517449
O	-1.860311	-0.013238	-1.009773
O	-0.814060	-0.745356	0.932091

H	0.210341	1.427911	1.215187
O	0.939753	0.915141	2.498779
H	0.651767	-0.004547	2.341323

Structure **B$_{OMe}$**

C	2.202495	-0.595597	1.595061
H	3.272375	-0.482390	1.748803
H	1.676846	-0.376022	2.525436
H	1.983821	-1.616622	1.279178
C	0.587842	0.139303	0.010187
C	1.489200	-1.366458	-1.571983
C	-0.558517	0.282392	1.003054
H	-0.403203	1.197341	1.574062
H	1.194470	-2.284033	-2.073647
H	1.569112	-0.557790	-2.302955
H	2.451687	-1.506376	-1.078604
S	-2.192672	0.416886	0.227360
C	-2.595696	-1.327930	-0.057536
H	-2.566371	-1.872971	0.884220
H	-3.610769	-1.342633	-0.449857
H	-1.911787	-1.769467	-0.775740
H	-0.559255	-0.568737	1.685085
C	0.480660	1.326611	-1.034296
O	0.481797	2.460125	-0.744015
O	1.845936	0.341569	0.577388
O	0.466007	-1.081745	-0.623456

Structure **TS2$_{OMe}$**

C	0.476491	2.292073	0.883806
H	0.766038	2.781277	1.809209
H	1.155266	2.583536	0.082052
H	-0.548558	2.566126	0.629912
C	0.457149	0.088802	0.045138
C	1.477949	-1.562061	1.386351
C	-0.582733	0.365105	-0.990460
H	-0.502796	-0.388584	-1.773635
H	1.560870	-2.644674	1.364247
H	2.450594	-1.109804	1.186783
H	1.112958	-1.232132	2.357304
S	-2.300616	0.393706	-0.356468
C	-2.545749	-1.358551	0.039932
H	-1.930145	-1.657471	0.883478
H	-3.598430	-1.469192	0.293386
H	-2.317335	-1.972799	-0.829358
H	-0.423364	1.345239	-1.438991
C	2.236989	0.456913	-1.093032
O	2.623380	-0.521583	-1.545547
O	0.572078	0.891497	1.128409
O	0.550064	-1.219225	0.355725

Structure **C$_{OMe}$**

C	-2.313612	-1.573824	-0.133616
H	-2.405254	-2.652552	-0.040321
H	-3.057601	-1.080027	0.494686
H	-2.456591	-1.276997	-1.171436
C	-0.762135	0.075900	0.496710
C	-1.131327	2.236129	-0.351522
C	0.555340	0.369529	1.114087
H	0.752605	-0.362455	1.895446
H	-1.683963	2.692525	-1.168100
H	-1.584743	2.514905	0.602052
H	-0.093578	2.573306	-0.382515
S	1.981162	0.380330	-0.060916
C	1.978188	-1.350965	-0.598921
H	1.085920	-1.574933	-1.177349
H	2.862632	-1.484693	-1.219074
H	2.038692	-2.011940	0.264134
H	0.569634	1.363461	1.559124
O	-0.999791	-1.245301	0.314630
O	-1.210841	0.829490	-0.544932

Structure **TS3$_{OMe}$**

C	0.539629	2.234215	-0.599585
H	1.036202	2.685841	-1.452438
H	-0.518133	2.078552	-0.817322
H	0.655035	2.871887	0.277720
C	0.710218	0.210094	0.594729
C	2.311862	-1.322799	-0.257751
C	-0.492950	0.412932	1.288887
H	-0.779621	1.440537	1.463057
H	2.647568	-2.318512	0.015164
H	1.871906	-1.334289	-1.255115
H	3.146861	-0.624586	-0.232039
S	-2.187878	-0.209624	0.140453
C	-1.278521	-1.103609	-1.153436
H	-0.712904	-1.932020	-0.730664
H	-2.013754	-1.496246	-1.853870
H	-0.607566	-0.434146	-1.691840
H	-0.590906	-0.226951	2.155376
O	1.188528	0.980970	-0.388210
O	1.327712	-0.973604	0.717919

Structure **D$_{OMe}$**

C	-0.000089	2.348290	-0.101817
H	-0.000128	3.082638	-0.901594
H	-0.892611	2.470260	0.514202
H	0.892408	2.470377	0.514218
C	0.000000	0.000000	0.067220
C	0.000089	-2.348290	-0.101817
C	0.000000	0.000000	1.404532
H	0.000008	0.924727	1.956388
H	-0.000008	-0.924727	1.956388
H	0.000128	-3.082638	-0.901594
H	0.892611	-2.470260	0.514202
H	-0.892408	-2.470377	0.514218
O	0.000000	1.078245	-0.735946
O	-0.000000	-1.078245	-0.735946

Structure **HO·**

O	0.000000	0.000000	0.108290
H	0.000000	0.000000	-0.866317

Structure **H_2O**

O	-0.000000	0.000000	0.117677
H	0.000000	0.760986	-0.470709
H	-0.000000	-0.760986	-0.470709

Structure **CO**

C	0.000000	0.000000	-0.640406
O	0.000000	0.000000	0.480304

Structure **MeS·**

C	1.105594	0.000066	-0.007984
H	1.497026	0.898940	-0.478121
H	1.496800	-0.895660	-0.484343
H	1.414603	-0.003844	1.040974
S	-0.690125	0.000010	-0.001913

Structure **A'**

C	1.093619	-2.156918	1.108136
C	1.024597	-0.633902	1.150451
H	1.334353	-2.548708	2.096567
H	0.142963	-2.599533	0.804327
H	1.863771	-2.505775	0.417345
C	0.743220	0.025218	-0.204042
H	0.255986	-0.315915	1.861143
H	1.960581	-0.228217	1.535500
C	1.853575	-0.261096	-1.255319
H	1.754662	-1.301025	-1.575208
H	1.649746	0.348449	-2.141511
C	3.275244	-0.004016	-0.767467
C	-0.575162	-0.427856	-0.839891
H	-0.542403	-1.483034	-1.115040
H	3.975931	-0.110666	-1.595708
H	3.385724	1.002605	-0.360049
H	3.565796	-0.714591	0.007267
S	-1.971391	-0.211822	0.299877
C	-3.284762	-0.443331	-0.908667
H	-3.213123	-1.452314	-1.312708
H	-4.227266	-0.317579	-0.380597
H	-3.173594	0.307787	-1.688998
H	-0.799192	0.162785	-1.732013
C	0.726799	1.537954	-0.061233
O	1.228307	2.142796	0.851043
H	0.285081	2.080248	-0.918602
O	-1.955751	1.263245	0.645013

Structure **TS1'**

C	-1.593230	-2.444267	-0.435216
C	-1.315561	-1.032217	-0.939563
H	-1.955983	-3.066076	-1.253676
H	-0.694413	-2.919104	-0.037608
H	-2.353693	-2.449823	0.347802
C	-0.855950	-0.055364	0.151067
H	-0.556981	-1.060760	-1.727460
H	-2.208874	-0.618472	-1.408147
C	-1.921627	0.164518	1.262589
H	-1.944203	-0.735497	1.881274
H	-1.572182	0.971261	1.914403
C	-3.323510	0.477924	0.752213
C	0.426996	-0.490700	0.863961
H	0.269397	-1.411545	1.427096
H	-3.972641	0.727046	1.591542
H	-3.323772	1.325231	0.064212
H	-3.761334	-0.376915	0.235666
S	1.774543	-0.822552	-0.308461
C	3.111016	-0.869905	0.892759
H	2.927977	-1.695009	1.579444
H	4.030569	-1.038783	0.337393
H	3.139458	0.084980	1.415779
H	0.775754	0.288528	1.545232
C	-0.647336	1.324708	-0.460430
O	-1.146339	1.728794	-1.464977
H	0.013153	2.029388	0.175331
O	1.407366	2.624066	0.590592
H	1.854119	1.997283	-0.024385
O	1.945995	0.479754	-1.082214

Structure **B'**

C	-1.067402	-2.120965	-1.152906
C	-1.005129	-0.596726	-1.159280
H	-1.272268	-2.487791	-2.158948
H	-0.124846	-2.565053	-0.827722
H	-1.859596	-2.488018	-0.498068
C	-0.747579	0.023687	0.223457
H	-0.225973	-0.258063	-1.847641
H	-1.939313	-0.185974	-1.544735
C	-1.870064	-0.281236	1.248430
H	-1.785693	-1.335818	1.520412
H	-1.660626	0.290191	2.156742
C	-3.283971	0.013897	0.760742
C	0.572162	-0.412002	0.860976
H	0.545907	-1.466878	1.138289
H	-3.992206	-0.132092	1.576269
H	-3.384029	1.042799	0.410245
H	-3.574055	-0.651093	-0.053577
S	1.967854	-0.204263	-0.284645
C	3.278376	-0.382764	0.935498
H	3.213316	-1.378041	1.373207
H	4.221698	-0.266908	0.406721
H	3.157290	0.393324	1.689396
H	0.783154	0.191724	1.745965
C	-0.742460	1.556792	0.095188
O	-1.237086	2.214405	-0.744118
O	1.938085	1.255822	-0.681490

Structure **TS2'**

C	-0.782009	2.551976	0.064887
C	-1.040039	1.338835	-0.822901
H	-1.108257	3.461979	-0.438761
H	-1.326719	2.478682	1.009455
H	0.280427	2.653369	0.286156
C	-0.789924	-0.004286	-0.187037
H	-2.071578	1.349754	-1.188507
H	-0.401975	1.404776	-1.714241
C	-1.156144	-1.179996	-1.058070
H	-2.181640	-1.034927	-1.410614
H	-0.528331	-1.135367	-1.959626
C	-1.012934	-2.555040	-0.411959
C	0.391866	-0.157764	0.711329
H	0.332642	-1.072103	1.304317
H	-1.399279	-3.325664	-1.078491
H	0.030540	-2.795437	-0.201470
H	-1.568732	-2.612796	0.526777
S	1.943636	-0.288874	-0.257511
C	3.051401	-0.477886	1.151290

H	2.823590	-1.416586	1.654138
H	4.063339	-0.500735	0.752709
H	2.917218	0.374245	1.816038
H	0.527100	0.694932	1.377885
C	-2.348251	-0.070817	1.212943
O	-3.377693	0.088435	0.745238
O	2.206403	1.090032	-0.817550

Structure **C'**

C	2.773042	-1.696836	-0.002581
C	1.586928	-1.022277	-0.711694
H	3.311285	-2.353438	-0.687240
H	3.474537	-0.950490	0.374987
H	2.426103	-2.292365	0.844031
C	0.818637	-0.129132	0.210033
H	1.965039	-0.452439	-1.566123
H	0.923184	-1.790054	-1.116837
C	1.453985	1.108779	0.764746
H	0.682541	1.746014	1.205563
H	2.115950	0.832879	1.599574
C	2.264465	1.910616	-0.254838
C	-0.459106	-0.608574	0.771737
H	-0.755883	-0.115400	1.699681
H	2.653657	2.821977	0.199405
H	3.113055	1.335981	-0.629301
H	1.647058	2.191388	-1.111129
S	-1.871147	-0.314623	-0.393140
C	-1.862386	1.485297	-0.314667
H	-0.922743	1.852920	-0.725203
H	-2.696214	1.829043	-0.922830
H	-1.991937	1.788127	0.724005
H	-0.491271	-1.692733	0.908402
O	-3.121175	-0.750585	0.339604

Structure **TS3'**

C	-3.212480	-1.013971	-0.198799
C	-1.765253	-1.111291	0.307001
H	-3.802841	-1.857876	0.160431
H	-3.688478	-0.095453	0.147688
H	-3.234959	-1.017379	-1.289566
C	-0.945039	0.066763	-0.129917
H	-1.774941	-1.169306	1.401289
H	-1.321509	-2.038126	-0.063091
C	-1.067663	1.316395	0.688126
H	-2.114817	1.432502	0.987657
H	-0.518426	1.155896	1.628188
C	-0.566390	2.595909	0.028337
C	-0.035790	-0.049797	-1.183856
H	0.271141	0.851929	-1.704579
H	-0.742629	3.451847	0.680312
H	0.504367	2.533802	-0.168372
H	-1.083837	2.777523	-0.916109
S	1.961435	-0.423885	-0.429120
C	1.503116	-1.660649	0.794970
H	1.108700	-2.533782	0.276025
H	2.406242	-1.923947	1.342638
H	0.759749	-1.233952	1.467000
H	-0.125401	-0.921951	-1.827017
O	2.313960	0.813784	0.363532

Structure **A''**

C	1.148761	2.316466	-0.617877
C	1.118029	0.835247	-0.979312
H	1.315352	2.915020	-1.513866
H	0.204796	2.641688	-0.178229
H	1.952449	2.544442	0.084989
C	0.927789	-0.103658	0.217244
H	0.320573	0.647813	-1.702108
H	2.045803	0.551951	-1.477443
C	2.066973	0.027781	1.269808
H	1.926919	0.971214	1.802545
H	1.937492	-0.763322	2.015200
C	3.475542	-0.034871	0.689959
C	-0.376903	0.131914	0.990935
H	-0.407905	1.122503	1.444025
H	4.206013	-0.055424	1.498743
H	3.621186	-0.929058	0.081303
H	3.691228	0.834937	0.068648
S	-1.839570	-0.001968	-0.034847
C	-3.166120	-0.210227	1.122237
H	-3.179399	0.648575	1.789383
H	-4.075475	-0.247235	0.524552
H	-3.017556	-1.141112	1.663016
H	-0.496580	-0.616702	1.778032
C	0.979771	-1.555369	-0.230391
O	1.478949	-1.932853	-1.257513
H	0.588045	-2.284818	0.504685
O	-1.707070	-1.220514	-0.821001
O	-2.052238	1.246781	-0.746678

Structure **TS1''**

C	-1.490495	-2.513573	-0.155047
C	-1.318266	-1.148488	-0.812392
H	-1.756299	-3.254974	-0.908828
H	-0.568445	-2.847897	0.322319
H	-2.284197	-2.505077	0.594242
C	-0.994153	-0.015797	0.171356
H	-0.526530	-1.197305	-1.563744
H	-2.226637	-0.871738	-1.348389
C	-2.111290	0.207704	1.231658
H	-2.055740	-0.614217	1.948974
H	-1.873345	1.116491	1.793123
C	-3.521048	0.303655	0.660353
C	0.300100	-0.228735	0.965187
H	0.241401	-1.109000	1.604541
H	-4.219381	0.569875	1.453597
H	-3.590922	1.064984	-0.118458
H	-3.846446	-0.646311	0.235435
S	1.739933	-0.458642	-0.076066
C	3.105823	-0.126927	1.001174
H	3.070274	-0.833500	1.827153
H	4.002186	-0.276278	0.401454
H	3.031429	0.902112	1.345761
H	0.520311	0.646061	1.581360
C	-0.905216	1.306742	-0.579937
O	-1.425578	1.555547	-1.622680
H	-0.327310	2.127425	-0.017394
O	0.990296	2.938418	0.424295

H	1.539663	2.486752	-0.245351
O	1.815589	-1.838184	-0.518494
O	1.699485	0.578392	-1.105978

Structure **B''**

C	1.133175	2.293470	-0.643628
C	1.100467	0.807707	-0.988194
H	1.259425	2.879503	-1.554484
H	0.204107	2.615718	-0.172023
H	1.962859	2.534824	0.023001
C	0.931597	-0.110811	0.233549
H	0.291364	0.608694	-1.693936
H	2.023462	0.519982	-1.493756
C	2.080177	0.034143	1.266307
H	1.947540	0.995344	1.767953
H	1.947381	-0.735605	2.031251
C	3.483500	-0.052369	0.677756
C	-0.374541	0.099272	1.004485
H	-0.412696	1.082291	1.473266
H	4.218786	-0.036658	1.482073
H	3.627324	-0.973195	0.109859
H	3.694665	0.789731	0.017942
S	-1.834361	-0.013461	-0.032225
C	-3.154066	-0.302080	1.114763
H	-3.173644	0.517729	1.829280
H	-4.065363	-0.315444	0.519073
H	-2.991928	-1.260340	1.601159
H	-0.485379	-0.670297	1.770712
C	0.996065	-1.580612	-0.217326
O	1.515655	-2.019470	-1.174612
O	-1.686502	-1.183394	-0.884909
O	-2.063701	1.269211	-0.675734

Structure **TS2''**

C	1.095277	-0.494212	2.565102
C	1.095277	-1.297262	1.268317
H	1.301537	-1.149800	3.410956
H	1.862985	0.283359	2.552734
H	0.127601	-0.022815	2.739055
C	0.963026	-0.494385	0.000000
H	2.017022	-1.880783	1.189368
H	0.273124	-2.023626	1.291930
C	1.095277	-1.297262	-1.268317
H	2.017022	-1.880783	-1.189368
H	0.273124	-2.023626	-1.291930
C	1.095277	-0.494212	-2.565102
C	0.036383	0.682363	0.000000
H	0.154738	1.303690	-0.889076
H	1.301537	-1.149800	-3.410956
H	0.127601	-0.022815	-2.739055
H	1.862985	0.283359	-2.552734
S	-1.699477	0.184811	-0.000000
C	-2.580318	1.725652	-0.000000
H	-2.317786	2.274749	-0.900919
H	-3.637039	1.462933	-0.000000
H	-2.317786	2.274749	0.900919
H	0.154738	1.303690	0.889076
C	2.795594	0.503809	0.000000
O	2.771106	1.645038	0.000000
O	-1.984586	-0.509793	1.244537
O	-1.984586	-0.509793	-1.244537

Structure **C''**

C	-3.020478	-1.673677	-0.151568
C	-2.015477	-0.821881	0.642713
H	-3.677122	-2.225767	0.522180
H	-3.638487	-1.041076	-0.790726
H	-2.500532	-2.392909	-0.786903
C	-1.115984	-0.042158	-0.262509
H	-2.565038	-0.139732	1.296343
H	-1.429362	-1.480590	1.291105
C	-1.505186	1.335900	-0.691627
H	-0.899457	1.645557	-1.545734
H	-2.550151	1.321665	-1.027238
C	-1.368389	2.378318	0.428730
C	0.071283	-0.709734	-0.839293
H	0.234902	-0.483547	-1.897916
H	-1.711843	3.355663	0.086428
H	-1.964952	2.098986	1.298750
H	-0.326155	2.465356	0.737017
S	1.632618	-0.148550	-0.102848
C	1.442565	-0.439023	1.637918
H	1.287090	-1.503686	1.797142
H	2.372478	-0.110065	2.098683
H	0.601619	0.151667	1.995310
H	0.073856	-1.792292	-0.699835
O	1.748192	1.286925	-0.312065
O	2.706966	-0.994287	-0.602337

Structure **TS3''**

C	-1.810040	-2.407180	-0.261344
C	-2.137467	-1.061559	0.372769
H	-2.339011	-3.204846	0.259755
H	-2.110298	-2.434037	-1.310378
H	-0.741073	-2.621231	-0.206033
C	-1.482672	0.140243	-0.240878
H	-3.220302	-0.895110	0.371813
H	-1.854207	-1.075239	1.433840
C	-1.864612	1.455950	0.367255
H	-2.951389	1.566471	0.274756
H	-1.675101	1.380250	1.445236
C	-1.164200	2.687442	-0.189100
C	-0.494878	0.027796	-1.185747
H	-0.128682	0.911529	-1.695704
H	-1.515510	3.582442	0.323901
H	-0.084005	2.623629	-0.044291
H	-1.361203	2.813495	-1.255049
S	1.426561	-0.230748	0.136247
C	2.760987	0.725755	-0.597486
H	2.444220	1.765059	-0.631349
H	3.626007	0.595423	0.051781
H	2.946273	0.327732	-1.591675
H	-0.330939	-0.912954	-1.696956
O	1.837999	-1.638287	0.127260
O	1.115444	0.370623	1.434938

Structure **MeOS·**

S	-0.218858	-0.503637	0.000013

C	1.386724	0.307080	0.000017
H	1.470095	0.920469	-0.895545
H	1.471366	0.919166	0.896320
H	2.144967	-0.474732	-0.001100
O	-1.238130	0.606352	0.000001

Structure **MeO_2S·**

S	0.191643	-0.000009	-0.274504
C	-1.575672	0.000062	0.102675
H	-1.645539	-0.000105	1.189106
H	-1.993594	0.902463	-0.332440
H	-1.993698	-0.902207	-0.332645
O	0.751343	1.262366	0.203251
O	0.751229	-1.262414	0.203248

Structure **Sec α-S rad**

C	0.915236	2.235995	-0.746738
C	0.903367	0.746910	-1.074726
H	1.014940	2.825692	-1.658451
H	-0.009804	2.534971	-0.249581
H	1.748369	2.495736	-0.091150
C	0.644011	-0.146919	0.144744
H	0.144074	0.536394	-1.831423
H	1.854063	0.450842	-1.524223
C	1.645901	0.071623	1.291601
H	1.468751	1.058975	1.723781
H	1.418564	-0.652476	2.080411
C	3.104526	-0.070971	0.868432
C	-0.750007	-0.007711	0.691286
H	3.760048	-0.014867	1.737555
H	3.285114	-1.030685	0.377133
H	3.400592	0.718565	0.176277
S	-2.104335	0.189946	-0.349006
C	-3.460979	-0.036808	0.828547
H	-3.391413	0.707428	1.618380
H	-4.384919	0.103405	0.272831
H	-3.427403	-1.041650	1.242459
H	-0.949324	-0.235732	1.731280
C	0.689566	-1.616468	-0.256268
O	0.605518	-2.028681	-1.387415
H	0.743832	-2.320192	0.596875

Structure **Prim α-S rad**

C	-0.743406	2.578260	-0.359676
C	-0.907387	1.255494	-1.101431
H	-0.974282	3.412729	-1.021813
H	-1.411322	2.642700	0.502864
H	0.280107	2.711042	-0.005305
C	-0.770087	-0.004061	-0.217187
H	-1.888777	1.218496	-1.584159
H	-0.170875	1.187447	-1.906070
C	-0.876448	-1.252567	-1.121240
H	-1.860000	-1.233904	-1.600678
H	-0.144334	-1.152394	-1.926456
C	-0.675260	-2.582172	-0.401259
C	0.489453	0.005816	0.644244
H	0.529779	-0.881066	1.275898
H	-0.887169	-3.411774	-1.075746
H	0.352908	-2.693844	-0.053108
H	-1.337691	-2.677664	0.462648
S	1.995591	0.036479	-0.377132
C	3.193677	0.015941	0.843348
H	4.223178	0.030410	0.522099
H	2.927789	-0.220700	1.862555
H	0.504820	0.880291	1.293496
C	-1.976535	-0.026619	0.698123
O	-1.941162	-0.037876	1.901549
H	-2.948330	-0.032975	0.165961

7 References

(1) Nucci, A.; Marino-Merlo, F.; De Nisco, M.; Pedatella, S.; Rossi, F.; Jacob, C.; Caputo, R.; Mastino, A. Se-(2-aminoalkyl)selenocysteines as biochemical redox agents. A tool to contrast cell injury induced by aflatoxin B1 in HepG2 cells. *Amino Acids* **2014**, *46* (2), 459–470. DOI: 10.1007/s00726-013-1639-1.

(2) Zhao, Y.; Pluth, M. D. Hydrogen Sulfide Donors Activated by Reactive Oxygen Species. *Angew. Chem. Int. Ed.* **2016**, *55* (47), 14638–14642. DOI: 10.1002/anie.201608052.

(3) Ji, X.; Zhou, C.; Ji, K.; Aghoghovbia, R. E.; Pan, Z.; Chittavong, V.; Ke, B.; Wang, B. Click and Release: A Chemical Strategy toward Developing Gasotransmitter Prodrugs by Using an Intramolecular Diels-Alder Reaction. *Angew. Chem. Int. Ed.* **2016**, *55* (51), 15846–15851. DOI: 10.1002/anie.201608732.

(4) Bender, M. L.; Clement, G. E.; Kezdy, F. J.; Heck, H. D. A. The Correlation of the pH (pD) Dependence and the Stepwise Mechanism of α-Chymotrypsin-Catalyzed Reactions. *J. Am. Chem. Soc.* **1964**, *86* (18), 3680–3690. DOI: 10.1021/ja01072a017.

(5) Liu, Z.; Zhang, G.; Lu, W.; Huang, Y.; Zhang, J.; Chen, T. UV light-initiated RAFT polymerization induced self-assembly. *Polym. Chem.* **2015**, *6* (34), 6129–6132. DOI: 10.1039/c5py00907c.

(6) Frisch, M. J. T., G. W.; Schlegel, H. B.; Scuseria, G. E.; Robb, M. A.; Cheeseman, J. R.; Scalmani, G.; Barone, V.; Petersson, G. A.; Nakatsuji, H.; Li, X.; Caricato, M.; Marenich, A. V.; Bloino, J.; Janesko, B. G.; Gomperts, R.; Mennucci, B.; Hratchian, H. P.; Ortiz, J. V.; Izmaylov, A. F.; Sonnenberg, J. L.; Williams-Young, D.; Ding, F.; Lipparini, F.; Egidi, F.; Goings, J.; Peng, B.; Petrone, A.; Henderson, T.; Ranasinghe, D.; Zakrzewski, V. G.; Gao, J.; Rega, N.; Zheng, G.; Liang, W.; Hada, M.; Ehara, M.; Toyota, K.; Fukuda, R.; Hasegawa, J.; Ishida, M.; Nakajima, T.; Honda, Y.; Kitao, O.; Nakai, H.; Vreven, T.; Throssell, K.; Montgomery, J. A., Jr.; Peralta, J. E.; Ogliaro, F.; Bearpark, M. J.; Heyd, J. J.; Brothers, E. N.; Kudin, K. N.; Staroverov, V. N.; Keith, T. A.; Kobayashi, R.; Normand, J.; Raghavachari, K.; Rendell, A. P.; Burant, J. C.; Iyengar, S. S.; Tomasi, J.; Cossi, M.; Millam, J. M.; Klene, M.; Adamo, C.; Cammi, R.; Ochterski, J. W.; Martin, R. L.; Morokuma, K.; Farkas, O.; Foresman, J. B.; Fox, D. J. Gaussian, Inc.,. Gaussian 16, Revision C.01,. Gaussian, Inc., Wallingford CT, 2016.

(7) Zhao, Y.; Truhlar, D. G. The M06 suite of density functionals for main group thermochemistry, thermochemical kinetics, noncovalent interactions, excited states, and transition elements: two new functionals and systematic testing of four M06-class functionals and 12 other functionals. *Theor. Chem. Acc.* **2008**, *120* (1-3), 215–241. DOI: 10.1007/s00214-007-0310-x.

(8) Scalmani, G.; Frisch, M. J. Continuous surface charge polarizable continuum models of solvation. I. General formalism. *J. Chem. Phys.* **2010**, *132* (11), 114110. DOI: 10.1063/1.3359469.

(9) Ribeiro, R. F.; Marenich, A. V.; Cramer, C. J.; Truhlar, D. G. Use of solution-phase vibrational frequencies in continuum models for the free energy of solvation. *J. Phys. Chem. B* **2011**, *115* (49), 14556–14562. DOI: 10.1021/jp205508z.

(10) Hratchian, H. P.; Schlegel, H. B. Accurate reaction paths using a Hessian based predictor-corrector integrator. *J. Chem. Phys.* **2004**, *120* (21), 9918–9924. DOI: 10.1063/1.1724823.

(11) Hratchian, H. P.; Schlegel, H. B. Using Hessian Updating To Increase the Efficiency of a Hessian Based Predictor-Corrector Reaction Path Following Method. *J. Chem. Theory Comput.* **2005**, *1* (1), 61–69. DOI: 10.1021/ct0499783.

(12) Maeda, A.; Bu, J.; Chen, J.; Zheng, G.; DaCosta, R. S. Dual in vivo photoacoustic and fluorescence imaging of HER2 expression in breast tumors for diagnosis, margin assessment, and surgical guidance. *Mol. Imaging* **2014**, *13*. DOI: 10.2310/7290.2014.00043.

(13) Junutula, J. R.; Raab, H.; Clark, S.; Bhakta, S.; Leipold, D. D.; Weir, S.; Chen, Y.; Simpson, M.; Tsai, S. P.; Dennis, M. S.; et al. Site-specific conjugation of a cytotoxic drug to an antibody improves the therapeutic index. *Nat. Biotechnol.* **2008**, *26* (8), 925–932. DOI: 10.1038/nbt.1480.

(14) Vasco, A. V.; Taylor, R. J.; Mendez, Y.; Bernardes, G. J. L. On-Demand Thio-Succinimide Hydrolysis for the Assembly of Stable Protein-Protein Conjugates. *J. Am. Chem. Soc.* **2024**, *146* (30), 20709–20719. DOI: 10.1021/jacs.4c03721.

(15) Seixas, J. D.; Chaves-Ferreira, M.; Montes-Grajales, D.; Goncalves, A. M.; Marques, A. R.; Saraiva, L. M.; Olivero-Verbel, J.; Romao, C. C.; Bernardes, G. J. An N-Acetyl Cysteine Ruthenium Tricarbonyl Conjugate Enables Simultaneous Release of CO and Ablation of Reactive Oxygen Species. *Chem. Eur. J.* **2015**, *21* (42), 14708–14712. DOI: 10.1002/chem.201502474.

(16) Liu, K.; Kong, X.; Ma, Y.; Lin, W. Rational Design of a Robust Fluorescent Probe for the Detection of Endogenous Carbon Monoxide in Living Zebrafish Embryos and Mouse Tissue. *Angew. Chem. Int. Ed.* **2017**, *56*, 13489–13492.

(17) Clark, J. E.; Naughton, P.; Shurey, S.; Green, C. J.; Johnson, T. R.; Mann, B. E.; Foresti, R.; Motterlini, R. Cardioprotective actions by a water-soluble carbon monoxide-releasing molecule. *Circ. Res.* **2003**, *93* (2), e2–8. DOI: 10.1161/01.RES.0000084381.86567.08.

(18) Michel, B. W.; Lippert, A. R.; Chang, C. J. A reaction-based fluorescent probe for selective imaging of carbon monoxide in living cells using a palladium-mediated carbonylation. *J. Am. Chem. Soc.* **2012**, *134* (38), 15668–15671. DOI: 10.1021/ja307017b.
